# Optimization of Cyclophilin B-Targeted Tri-vector Inhibitors for Novel MASH Treatments

**DOI:** 10.1021/acs.jmedchem.5c00301

**Published:** 2025-03-12

**Authors:** Maria-Eleni Kouridaki, Jonathan Gillespie, John Robinson, Tanya Mathie, Laura Bain, Duncan McArthur, Angus Morrison, Daniel B. Greenslade, Michail Papadourakis, Kasia Maj, Kate Cameron, Darryl Turner, Scott P. Webster, Martin A. Wear, Dahlia Doughty-Shenton, Alison N. Hulme, Julien Michel

**Affiliations:** a EaStCHEM School of Chemistry, University of Edinburgh, David Brewster Road, Edinburgh, Scotland EH9 3FJ, U.K.; b BioAscent Discovery Ltd., Newhouse, Scotland Lanarkshire ML1 5UH, U.K.; c Cytochroma Ltd., Roslin Innovation Centre, Easter Bush Estate, Edinburgh, Scotland EH25 9RG, U.K.; d Concept Life Sciences Ltd., Nine, 9 Little France Road, Edinburgh Bioquarter, Edinburgh, Scotland EH16 4UX, U.K.; e Centre for Cardiovascular Science, Queen’s Medical Research Institute, University of Edinburgh, 47 Little France Crescent, Edinburgh, Scotland EH16 4TJ, U.K.; f The Edinburgh Protein Production Facility (EPPF), University of Edinburgh, Level 3 Michael Swann Building, King’s Buildings, Max Born Crescent, Edinburgh, Scotland EH9 3FF, U.K.; g Centre for Reproductive Health, Institute for Regeneration and Repair, University of Edinburgh, 4-5 Little France Drive, Edinburgh Bioquarter, Edinburgh, Scotland EH16 4UU, U.K.

## Abstract

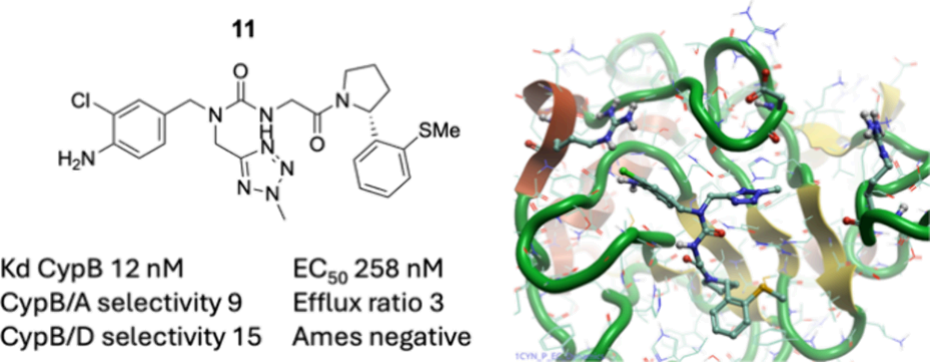

Cyclophilins have been implicated in the pathophysiology of metabolic dysfunction-associated steatohepatitis (MASH). Pharmacological inhibition of the cyclophilin B isoform has the potential to attenuate liver fibrosis in MASH, but current cyclophilin inhibitors in clinical trials lack isoform selectivity. We previously reported the novel tri-vector small-molecule inhibitor **1** that exhibited improved subtype selectivity by simultaneously engaging three pockets on the surface of cyclophilins. Here, we present structure–activity relationships that address genotoxicity concerns, enhance subtype selectivity, improve pharmaceutical properties, and demonstrate strong efficacy in a MASH cellular model. Lead compound **11** is a potent cyclophilin B inhibitor with an encouraging pharmacokinetic profile suitable for further development.

## Introduction

Metabolic dysfunction associated steaotic liver disease (MASLD, formerly known as NAFLD^1^) is an increasingly prevalent condition characterized by liver steatosis in the absence of hereditary disorders. Histological examination enables characterization of MASLD as a benign condition or a more concerning metabolic dysfunction associated steatohepatitis (MASH).^1,2^ Left untreated MASH can progress to cirrhosis, liver failure and hepatocellular carcinoma (HCC).^3^ MASH is a complex metabolic syndrome that is challenging to treat owing to a lack of understanding of the mechanisms of the disease. To date, over 50 investigational new drugs have been developed for various metabolic, inflammation and fibrosis MASH targets.^4^ In March 2024 the FDA approved the oral thyroid hormone receptor-beta (THRβ) agonist Resmetirom as the first MASH treatment. In a phase III trial Resmetirom improved liver pathology in ca. 25–30% of patients.^5^ Future improvements in standard of care may require combination therapies, necessitating approval of other MASH therapeutics.

Cyclophilins are a family of proteins that play a crucial role in human biology by assisting protein folding and trafficking, contributing to the regulation of a multitude of cellular processes.^6,7^ Cyclophilin inhibitors have been proposed as attractive MASH therapeutics owing to the role played by the cyclophilin A isoform (Cyp A) in mediating inflammation, and the role of the cyclophilin D isoform (Cyp D) in necrotic cell death.^8,9^ Pharmacological inhibition of the cyclophilin B isoform (Cyp B) in MASH may be particularly relevant owing to the documented role of this isoform in collagen production.^10^ Previous studies have shown that Cyp B is significantly upregulated in fibrotic tissues compared to normal tissues. Fibrosis is the only histological marker that has been significantly associated with liver failure in MASH patients.^11^ Nonimmunosuppressive cyclophilin inhibitors derived from the natural products Cyclosporine (**CsA**) or Sanglifehrin (**SfA**),^12^ have been shown to have antifibrotic effects in the CCL4 model of liver fibrosis and in a mouse model of MASH.^13,14^ These findings have motivated Phase 2 MASH clinical trials of the nonimmunosuppressive cyclophilin inhibitor Rencofilstat.^15^

**Figure 1:**
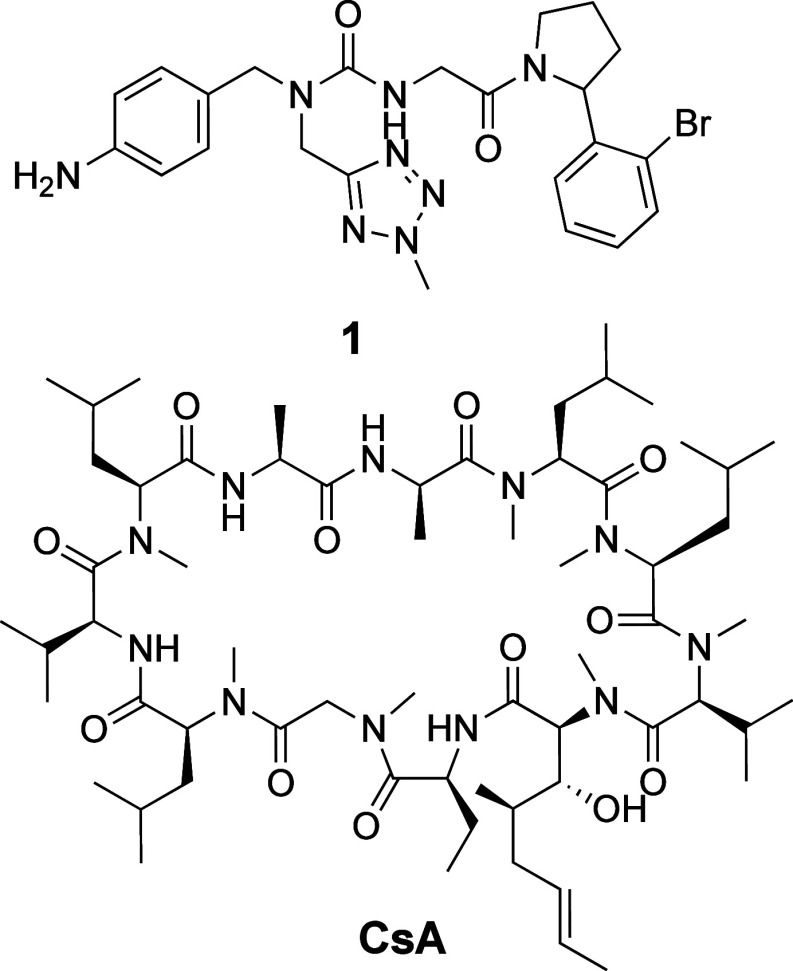
Chemical structures of tri-vector cyclophilin inhibitor **1** and cyclosporine A (**CsA**).

Cyclophilin inhibitors that have entered clinical studies are pan-selective inhibitors, which may limit engagement of cyclophilin isoforms B or D owing to the large relative abundance of cyclophilin A.^16,17^ It is thus desirable to develop new classes of cyclophilin inhibitors that exhibit greater subtype selectivity and ease of development, as cyclophilin inhibitors derived from the natural products **CsA** or **Sfa** are difficult to modify to optimize their pharmaceutical properties. Several groups have reported various classes of small molecule inhibitors.^18^ Achieving subtype selectivity has generally proven challenging owing to the high degree of conservation of the two major pockets occupied by most known inhibitors. Peterson et al. recently reported Cyp D selective inhibitors based on a macrocycle scaffold but had to rely on a pro-drug strategy to achieve cell permeability.^16^ We previously reported **1**, a small molecule tri-vector cyclophilin inhibitor that simultaneously engages three pockets on the surface of cyclophilins (Figure 1). **1** demonstrated efficacy comparable to **CsA** in an antiproliferative cell assay, as well as reduced cytotoxicity, suggesting potential for differentiation from macrocyclic cyclophilin inhibitors.^19^ Nevertheless **1** showed only a modest selectivity profile for Cyp B over Cyp A, and featured a primary aromatic amine moiety that was deemed a development concern as primary aromatic amines may undergo metabolic activation into reactive nitrenium ions that form covalent adducts with DNA.^20^ We thus set out to design improved tri-vector cyclophilin inhibitors to address mutagenicity concerns, optimize subtype selectivity, and pharmaceutical properties. These efforts lead to the development of **11**, a potent Cyp B inhibitor with encouraging pharmacokinetic profile for further development.

## Results and Discussion

### Molecular Modeling

**Figure 2:**
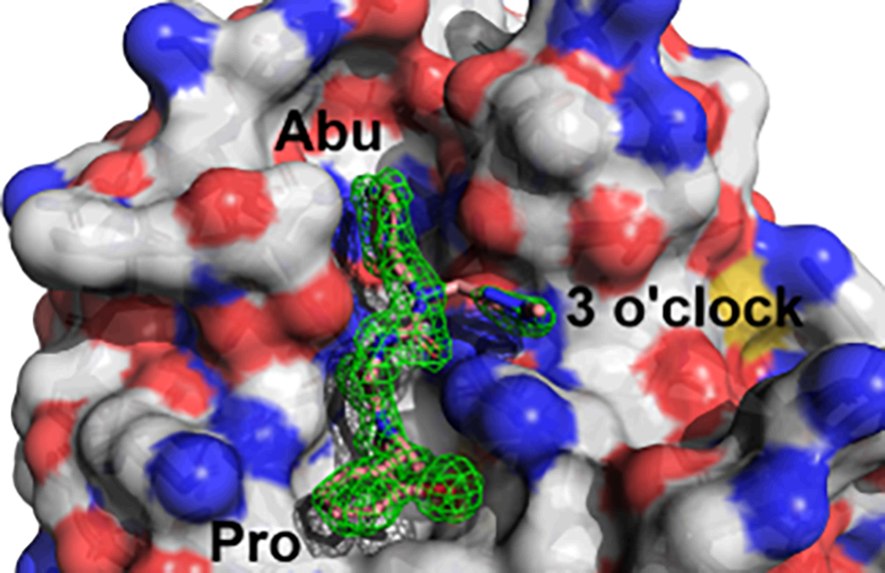
X-ray diffraction structure of Cyp A/**1** (PDB ID 6GJN) used for affinity calculations. **1** occupies the active site pockets Abu and Pro, and the accessory 3 o’clock pocket. Fo – Fc electron density omit maps are shown as a green mesh at 1.5σ contour.

An *in silico* affinity model derived from an X-ray diffraction crystal structure of Cyp A in complex with **1** (Figure 2) was first benchmarked against a series of previously reported phenyl-pyrrolidine urea ligands for cyclophilin A.^19,21^ by computing binding free energies with the software Flare FEP v5.^22^ The resulting FEP affinity model showed encouraging ranking ability (r^2^ 0.56, MUE 0.4 kcal.mol^–1^, Kendall τ 0.67, Figure S1), and was adopted to guide design of structurally novel improved inhibitors.

Thus, starting from **1** we performed FEP scans in the Abu, Pro and 3 o’clock pockets respectively (Figure 2). Pro pocket modifications focused on replacement of the *ortho*-bromo phenyl pyrrolidine substituent by alternative moieties to improve solubility and affinity. Replacement by chloride or linear, branched or cycloalkyl groups led to loss of affinity, and only thiomethylation was predicted to enhance potency (Table S1). FEP scans in the Abu pocket focused on overcoming genotoxicity liabilities associated with the aniline moiety. Replacement of the amino group by hydroxyl or amide groups, and methylation or cyclization were predicted to significantly worsen affinity. Detailed inspection of the Abu pocket indicated that the amino group donates two hydrogen bonds to a Cyp A residue and a buried water molecule. Since alternatives to this group could not be identified, the strategy switched to destabilizing the nitrenium ion derivative of **1** through *ortho*-halogenation or replacement of the phenyl group by nitrogen rich heterocycles.^23,24^ The approach was supported by our previous discovery of chlorinated pyridine fragments bound to the Abu pocket of Cyp A.^25^ Monohalogenation was predicted to provide a modest improvement in affinity, but dihalogenation decreased affinity. Pyridine or pyrimidine replacement was also predicted to enhance affinity. However, the predicted p*K*_a_ values for the aminopyridine variants suggested a significant fraction would be ionized in physiological conditions, which was expected to be detrimental for binding to the Abu pocket (Table S2). The FEP scan in the 3 o’clock pocket focused on exploration of alternative 5-membered rings. Replacement of the tetrazole moiety by thiadiazole, thiazole and oxadiazole rings was well tolerated. By contrast a moderate loss of affinity was predicted for furan, oxazole, isoxazole, triazole and thiophene variants. These modifications were associated with large changes in predicted LogP values, offering scope to strike a balance between cell permeability and solubility (Table S3). The most promising design features identified through the separate FEP scans were combined, to give a series of compounds predicted to have improved binding properties together with cLogP values ranging between 2 and 5 (Table S4).

### Compound Synthesis

Switching the *ortho*-bromophenyl pyrrolidine substituent to its thiomethyl equivalent as found in compound **2** was predicted to give improved binding in the Pro-pocket by FEP (Table S1). However, synthesis of this analogue was not readily achieved following our established route.^19^

**Scheme 1:**
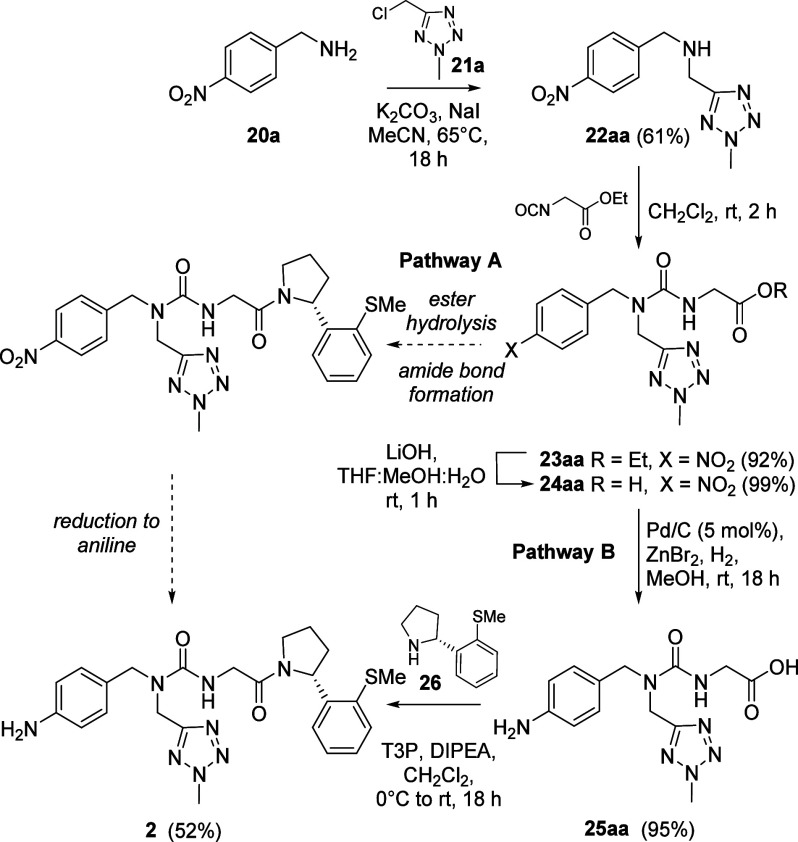
Synthesis of Thiomethyl Pro-pocket Analogue **2**

Nevertheless, optimization of the synthetic pathway gave valuable insight which was translated into the synthesis of further analogues (**3**-**14**), as well as skeletal variants (**15**-**19**). Alkylation of 4-nitrobenzylamine **20a** with 5-chloromethyl-2-methyl-tetrazole **21a** gave secondary amine **22aa** (Scheme 1) and subsequent coupling to ethyl isocyanoacetate gave urea **23aa**. Conversion to the desired thiomethyl-substituted analogue **2** via ester hydrolysis, pyrrolidine coupling and nitro group reduction failed due to catalyst poisoning in the final step (Pathway A, Scheme 1). The successful synthesis of **2** instead relied upon switching the order of nitro group reduction and amide coupling steps (Pathway B, Scheme 1), allowing a clean conversion of nitroaryl acid **24aa** into aniline acid **25aa** before T3P-mediated coupling to the (*R*)-*ortho*-thiomethylphenyl pyrrolidine (*R*)-**26** to give the desired tri-vector cyclophilin inhibitor **2**. Critical to this protecting-group free approach to coupling **25aa** without competing dimerization through amide formation on the aniline, was the strategic use of T3P and meticulous optimization of the coupling conditions.

**Scheme 2:**
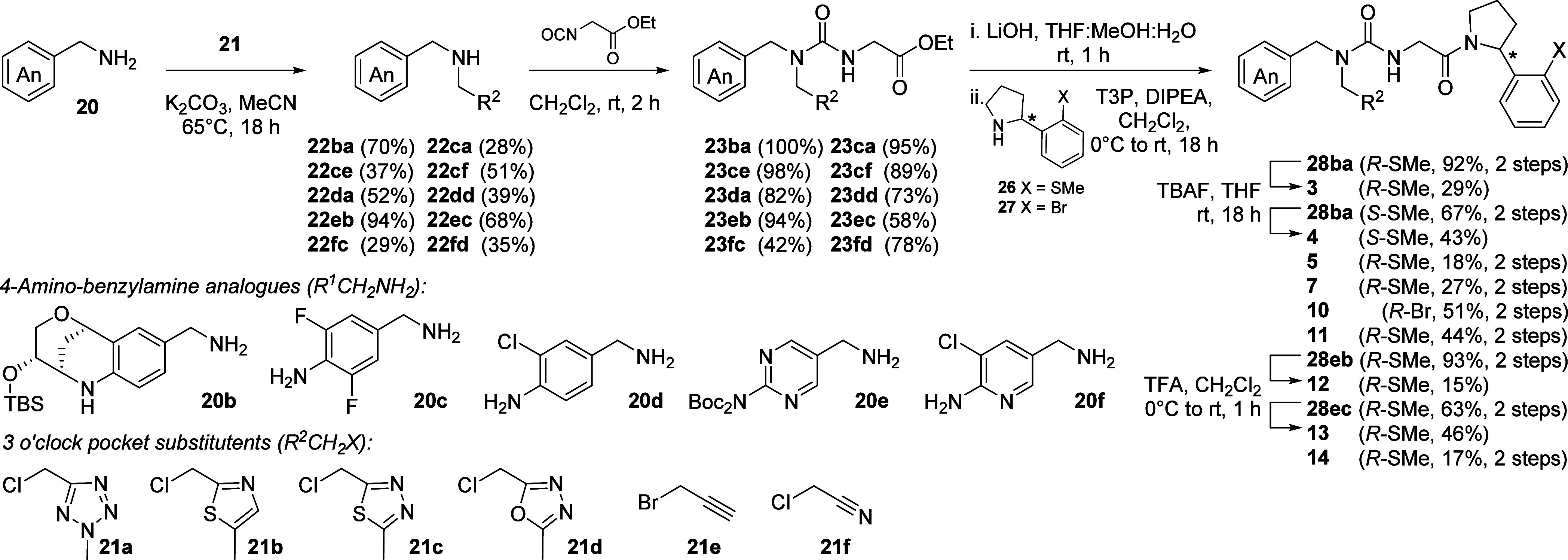
Urea Synthesis from 4-Amino-benzylamine Analogues (**20b**–**f**) and Pre-formed Methyl-substituted Chloromethyl-heteroaryls (**21a**–**d**) Propargyl Bromide **21e** and Chloroacetonitrile **21f**, to Give Tri-vector Inhibitors 3-5, 7 and 10-14

A series of 4-amino-benzylamine analogues (**20b**–**f**, Scheme 2) were used to explore binding in the Abu pocket. Though not modeled by FEP, this series included compounds featuring a hydroxy-12-oxa-8-azatricyclo[7.3.1.02,7]trideca-2,4,6-trien-4-yl] moiety projected into the Abu pocket, since a report from Gräedler et al. demonstrated that this substituent confers significant potency in a series of structurally related urea inhibitors.^36^ The TBS-protected derivative of this substituent (**20b**) was prepared by condensation of (4*S*,5*R*)-5-(Hydroxymethyl)tetrahydrofuran-2,4-diol with *tert*-butyl *N*-[(4-aminophenyl)methyl]carbamate, catalyzed by montmorillonite in acetonitrile,^26^ followed by TBS protection of the free hydroxyl and unmasking of the amino group for coupling (SI Experimental). Other 4-amino-benzylamine analogues (**20c**–**f**) were prepared by reduction of the corresponding benzonitriles;^27^ with the pyrimidinyl analogue bis-Boc protected through subsequent transformations (SI Experimental).

Anilines **20b**–**f** were readily coupled to the corresponding methyl-substituted chloromethyl-hetereoaryl derivatives including tetrazole (**21a**), oxazole (**21b**), thiadiazole (**21c**) and oxadiazole (**21d**) which were most highly ranked in our FEP studies of the 3 o’clock pocket, to give a selection of secondary amines **22**. Propargyl bromide (**21e**) and chloroacetonitrile (**21f**) were also coupled to give secondary amines which could be used for late-stage diversification of the 3 o’clock pocket moiety. Each of these secondary amines **22** could be coupled to ethyl isocyanotoacetate to give ureas **23** in good to excellent yields (generally >70%). Tetrazole (**23ba**, **23ca**, **23da**), thiazole (**23eb**), thiadiazole (**23ec**) and propargyl (**23ce**) *N*-substituted ureas were successfully subjected to hydrolysis and T3P-mediated amide bond formation with (*R*)-**26**. To probe the structural requirements of the Pro-pocket in more depth, coupling with (*S*)-*ortho*-thiomethylphenyl pyrrolidine (*S*)-**26** was used in the synthesis of **4**, and the (*R*)-bromo-analogue (*R*)-**27** was coupled for the synthesis of **10**. Both TBS-deprotection of the alcohol group in **28ba** ((*R*)- or (*S*)-SMe adduct) and Boc-deprotection of the amino group in **28eb** and **28ec**, to give compounds **3** & **4**, and **12** & **13**, respectively, proved somewhat difficult to achieve even under optimized conditions (15–46%).

**Scheme 3:**
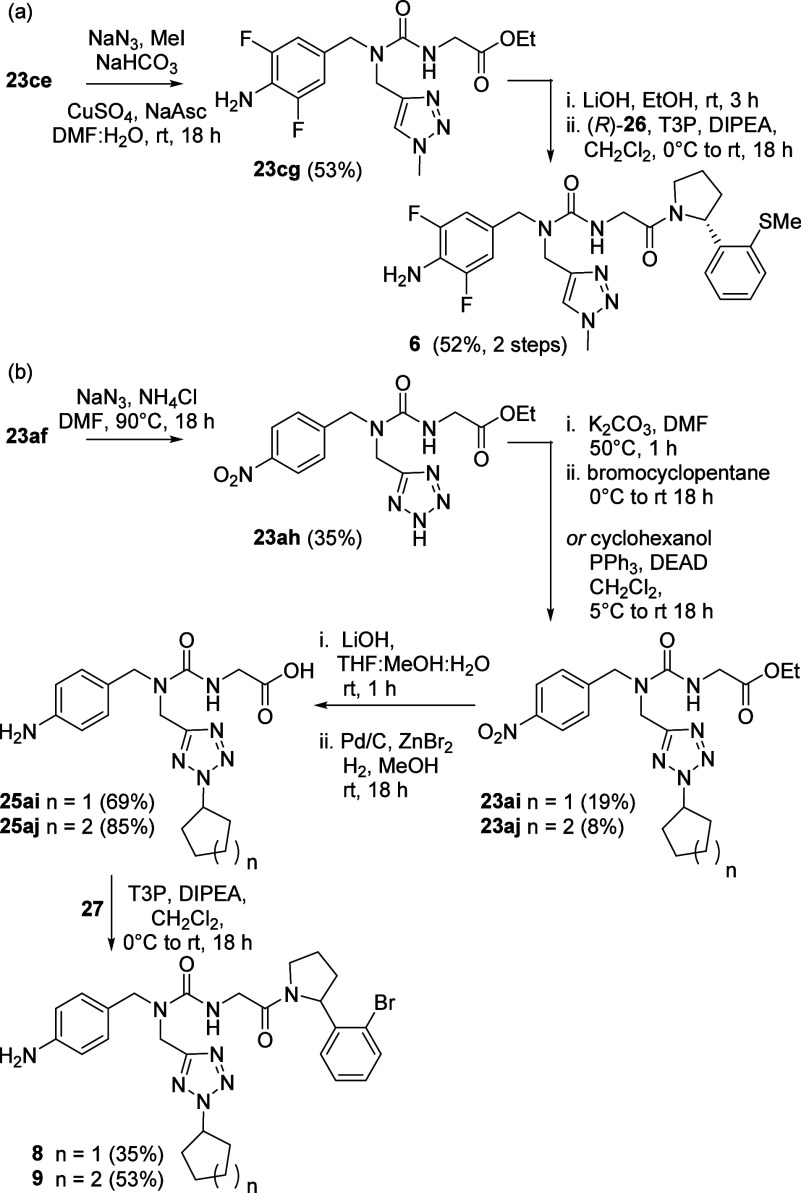
[3 + 2] Cycloaddition Reaction-Based Routes to the Synthesis of (a) Methyl-Substituted Triazole Analogue 6 and (b) Tetrazole Analogues 8-9

To facilitate the study of deeper binding in the 3 o’clock pocket, we explored the synthesis of substituted triazole and tetrazole derivatives from the propargyl and cyanomethyl-substituted urea intermediates (**23ce**) and (**23af**)^19^ via [3 + 2] cycloaddition reactions (Scheme 3). Formation of the methyl-substituted triazole **22cg** was achieved through CuAAC reaction of **22ce** with azidomethane formed *in situ*,^19^ providing a direct and efficient route to compound **6** (Scheme 3a). Correspondingly, tetrazole intermediate **23ah** was formed by the cycloaddition reaction of sodium azide with the carbonitrile function of urea **23af**.^19,28^ Nonselective alkylation of the tetrazole was achieved under either mild basic (*n* = 1, Scheme 3b),^29^ or Mitsunobu-type (*n* = 2, Scheme 3b)^30^ conditions to give modest yields of the nitro intermediates **23ai** and **23aj**, following chromatographic separation of the regioisomeric products. These 3 o’clock extended intermediates were smoothly converted into the desired analogues **8** and **9** following the optimized 3-step hydrolysis, nitro group reduction, amide bond coupling procedure.

**Scheme 4:**
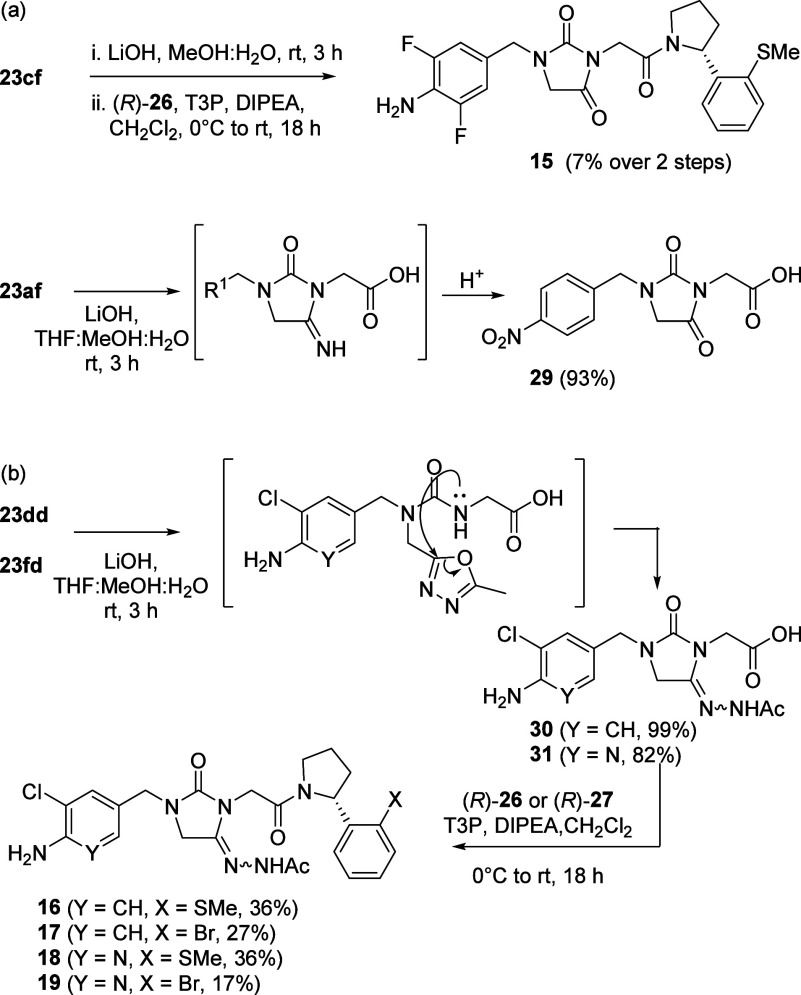
Unexpected Base-Induced Rearrangements of (a) Cyanomethyl Substituted Ureas **23cf** and **23af** and (b) Oxadiazole Substituted Ureas **23dd** and **23fd**

Although the synthetic route to trisubstituted ureas proceeded mostly as expected, when hydrolysis and amide bond formation was attempted on the cyanomethyl functionalized urea **23cf**, an unexpected hydantoin product **15** was observed (Scheme 4a). Detailed analysis of the reaction of related urea **23af** under the basic ester hydrolysis conditions, suggested initial formation of an hydantoin imine as reported for other simpler cyanomethyl substituted ureas.^31,32^ Conversion of this imine to the corresponding hydantoin occurred following prolonged treatment with acid; either deliberately during workup (**29**) or, as in the synthesis of the compound **15**, following acidic chromatography. Isolated intermediate **29** showed a loss of the nitrile/imine carbon peak in the NMR, an *m*/*z* value corresponding to the parent hydantoin and was characterized by extensive 2D NMR analysis. For the oxadiazole functionalized ureas **23dd** or **23fd**, a related rearrangement reaction to give hydantoin acylhydrazone derivatives **30** and **31** is likely to proceed via an intramolecular nucleophilic attack by the urea nitrogen similar to transformations described in the literature (Scheme 4b).^33,34^ These acyl hydrazones are characterized by NH peaks in the δ 9.0–10.0 ppm range, in contrast to their urea precursors with NH peaks in the δ 4.0–6.0 ppm range. Coupling intermediates **30** and **31** to amines (*R*)-(**26**) and (*R*)-(**27**) under previously optimized conditions allowed the isolation of acylhydrazones **16**-**19**.

### Structure–Activity Relationships

**Figure 3:**
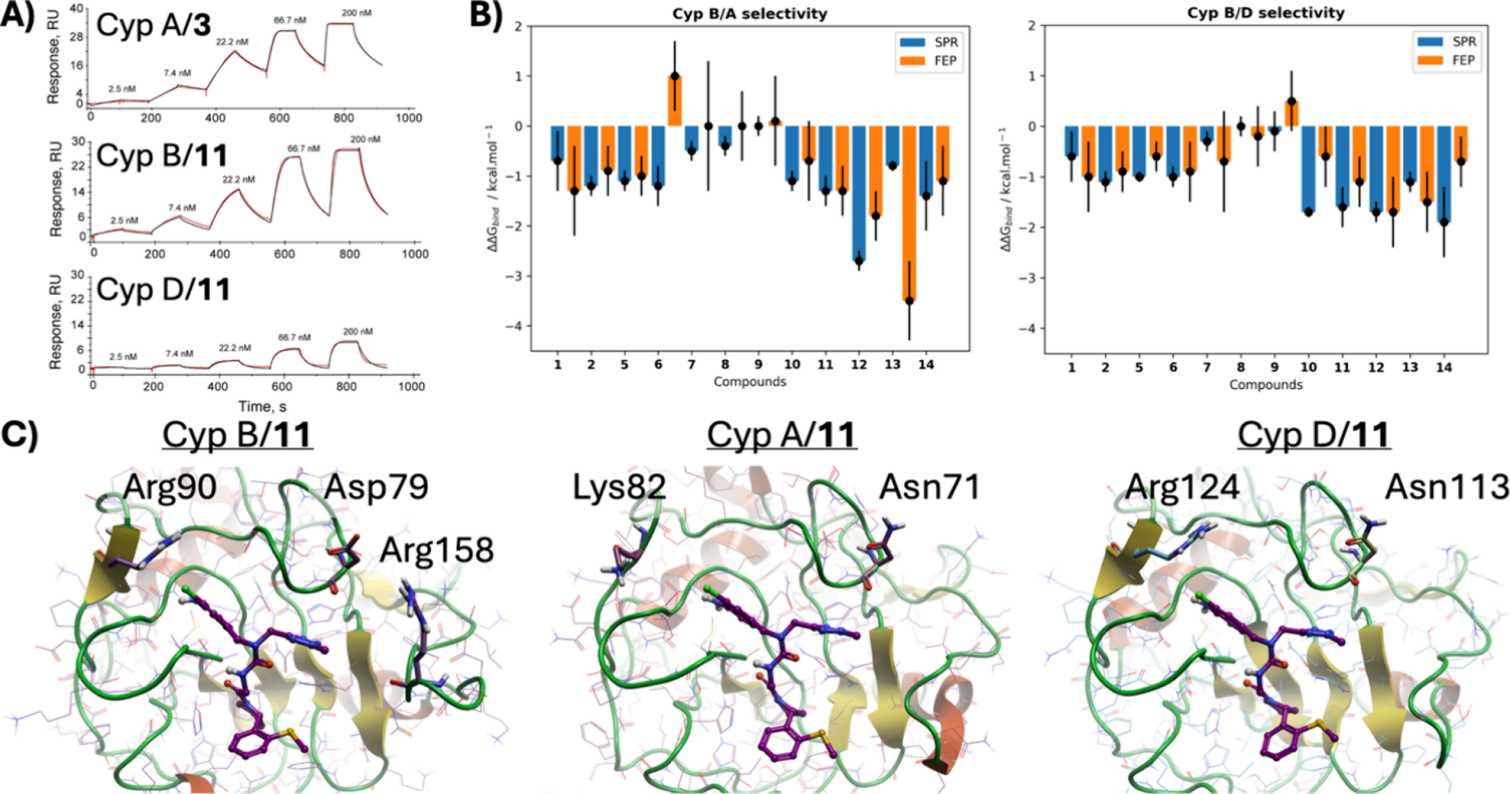
**A**) Representative sensorgrams from SPR kinetic titration experiments. **B**) Comparison of binding selectivity profiles between Cyp B/Cyp A and Cyp B/Cyp D measured by SPR or computed by FEP. Negative values indicate preferential binding to Cyp B. **C**) Representative snapshots from MD simulations-of models of **11** bound to Cyp B, Cyp A, Cyp D. Abu and 3 o’clock pocket residues that are not conserved between the three isoforms are depicted in ball and sticks representation. Models derived from PDB IDs 6GJN, 1CYN, 4J5B for Cyp A, Cyp B, Cyp D, respectively.

The cyclophilin binding properties of the compounds were assessed by Surface Plasmon Resonance (SPR) against Cyp A, B and D isoforms (Table 1, representative sensorgrams are shown in Figure 3A).^35^ Isothermal titration calorimetry experiments were also carried out for compound **11** and **CsA** to provide orthogonal validation of the SPR-derived K_d_ measurements (ESI for details).

**Table 1:**
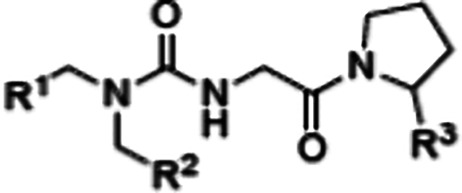

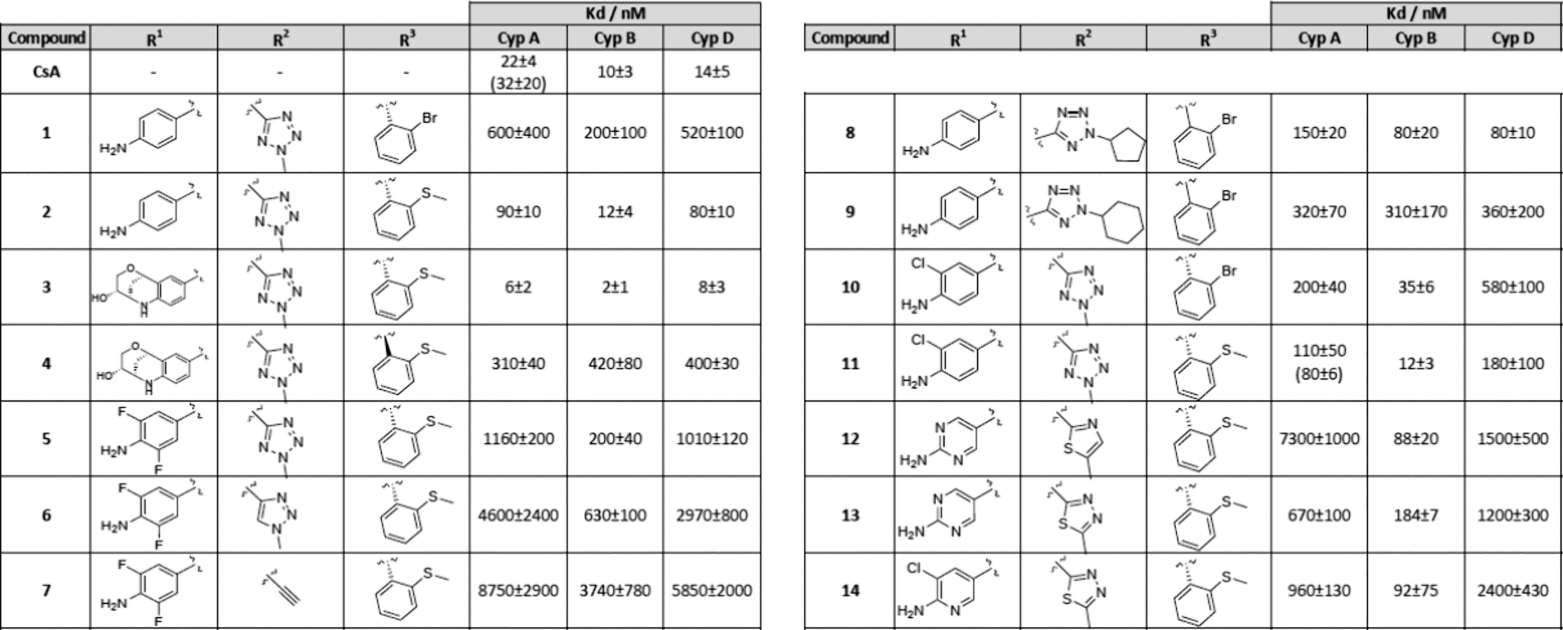
SPR-Derived Dissociation Constants for Compounds **1**-**14**^a^

a Values in parentheses are from ITC assays. Data for **1** from De Simone et al.^19^

Replacement of the bromophenyl Pro pocket moiety by a thiomethylphenyl (**1→2**) led to 10–20 fold improvements in binding affinities across the three Cyp isoforms, in line with the FEP predictions. Further replacement of the Abu pocket aniline moiety by the annulated tetrahydropyran reported by Gräedler et al.^36^ (**2** → **3**) yielded a further 10-fold improvement in affinity. Compound **3** showed an impressively low K_d_ for Cyp B (2 nM) albeit with only a small preference over Cyp A (3-fold). Replacement of the (*R*)-phenylpyrolidine by its (*S*)-enantiomer decreased potency 10–40 fold (**3** → **4**). FEP modeling suggested the difluorination strategy to deactivate the aniline (**2** → **5**) would incur a ca. 3-fold affinity loss, but the SPR measurements revealed more significant (*ca*. 10–15 fold) affinity loss across all three isoforms. Tetrazole to triazole replacement in the 3 o’clock pocket led to further undesirable 3–4 fold affinity loss (**5 → 6**) in line with FEP estimates. Tetrazole replacement by a smaller linear alkyne moiety (**5 → 7**) was not tolerated. These findings confirmed the importance of engaging the 3 o’clock pocket to achieve competitive potency among this compound series. Further expansion deeper in the 3′oclock pocket was assessed by replacement of the methyl substituted tetrazole by cyclopentyl and cyclohexyl substituted tetrazoles (**1 → 8** and **1 → 9**). These modifications did not significantly improve or worsen affinity, at the expense of significantly increased cLogP values.

These efforts compelled us to maintain a minimally decorated 5-membered ring in the 3-o’clock pocket and pursue alternative strategies to move away from the aniline liability. Monochlorination *ortho* to the aniline amino group improved potency 3–5 fold, in line with FEP modeling (**1 → 10**). This modification, combined with the bromo to thiomethyl phenylpyrrolidine replacement led to **11** that showed high Cyp B affinity (K_d_ ca. 10 nM) and 10–20-fold selectivity over Cyp A and Cyp D.

We then sought to replace the aniline by pyrimidine and chloro-pyridine variants, concomitantly with tetrazole replacements to maintain cLogP values within a range of 3–4. These considerations led us to prepare variants featuring oxadiazole, thiazole or thiadiazole heterocycles occupying the 3′oclock pocket. In disagreement with the predicted FEP trends the thiazole and thiadiazole series featuring pyrimidine and chloropyridine Abu pocket variations (**12**, **13**, **14**) showed weaker affinity when compared to **11**. Surprisingly, **12** demonstrated an impressive 80-fold selectivity for Cyp B over Cyp A. All the hydantoin derivatives (**15**, **16**, **17**, **18**, **19**) showed greatly decreased affinity for all isoforms compared to the linear urea-based compounds (Table 2). This was likely due to disruption of hydrogen-bonding interactions involving the urea nitrogen proximal to the pyrrolidine moiety.

**Table 2:**
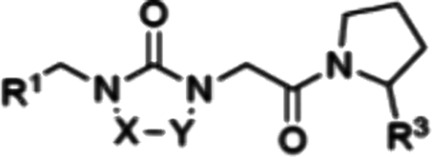

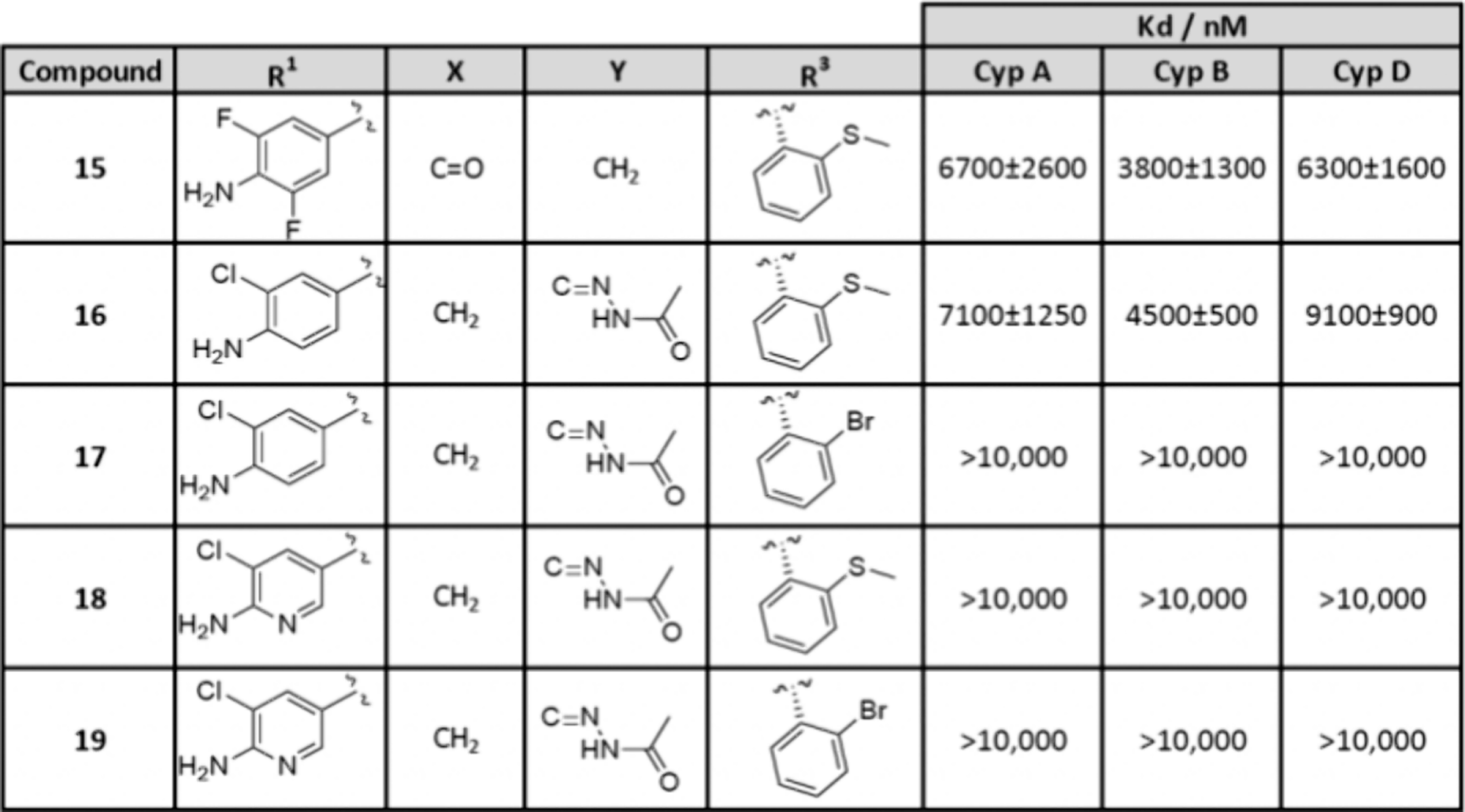
SPR-Derived Dissociation Constants for Compounds **15-19**

To further investigate the observed binding selectivity trends, we carried out another round of FEP modeling with Flare FEP version 8. Thus, compounds **1**, **2**, **5**-**14** were modeled in complex with Cyp A, Cyp B and Cyp D (ESI for details). Compounds **3**, **4** were excluded as handling of alchemical transformations involving the annulated tetrahydropyran ring were technically challenging. Compounds **15**-**19** were excluded owing to their poor affinities (Table 2). Figure 3B shows a comparison of Cyp B/A and Cyp B/D binding selectivity ratios measured by SPR and computed by FEP. Although there is no quantitative agreement between SPR measurements and FEP calculations for all compounds, both methodologies are broadly consistent in indicating preferential binding to Cyp B vs the other two isoforms for this ligand series. The binding selectivity differences may be explained by differences in residues found in the Abu and 3 o’clock pockets (Figure 3C). Arg90 in Cyp B is replaced by Lys82 in Cyp A, leading to a less occluded pocket in Cyp A. Asp79 in Cyp B is replaced by Asn71 in Cyp A and Asn113 in Cyp D. In addition, Arg158 in Cyp B is an insertion in the sequence alignment with Cyp A and Cyp D. These differences introduce variability in the electrostatic interactions of the heterocycles positioned in the 3 o’clock pocket.

### Cellular Assays

**Figure 4:**
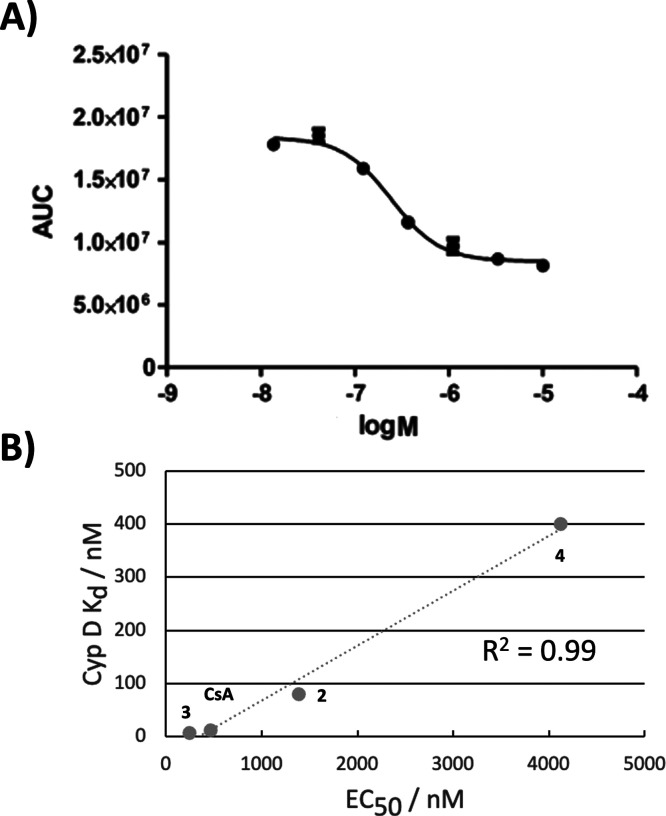
Calcium Retention Capacity assay in HepG2 cells. **A)** Concentration response curve for **3. B)** Correlation between EC_50_ values derived for four compounds and SPR derived Cyp D dissociation constants.

We then sought evidence of engagement of cyclophilins in a cellular context for some of the prepared compounds through use of a Calcium Retention Capacity assay in permeabilised human hepatocyte (HepG2) cells, a commonly used cell line for *in vitro* MAFLD studies.^37^ Notably, compound **3** demonstrated 2-fold greater efficacy over **CsA** in the calcium retention assay (EC_50_ 244 nM vs 463 nM, Figure 4A). The SPR-derived Cyp D dissociation constants of four tested compounds correlated strongly with the measured calcium retention assay EC_50_ values (Figure 4B). These results are consistent with the compounds acting as inhibitor of the Cyp D mediated opening of the mitochondrial permeability transition pore under calcium overload.^38^

**Figure 5:**
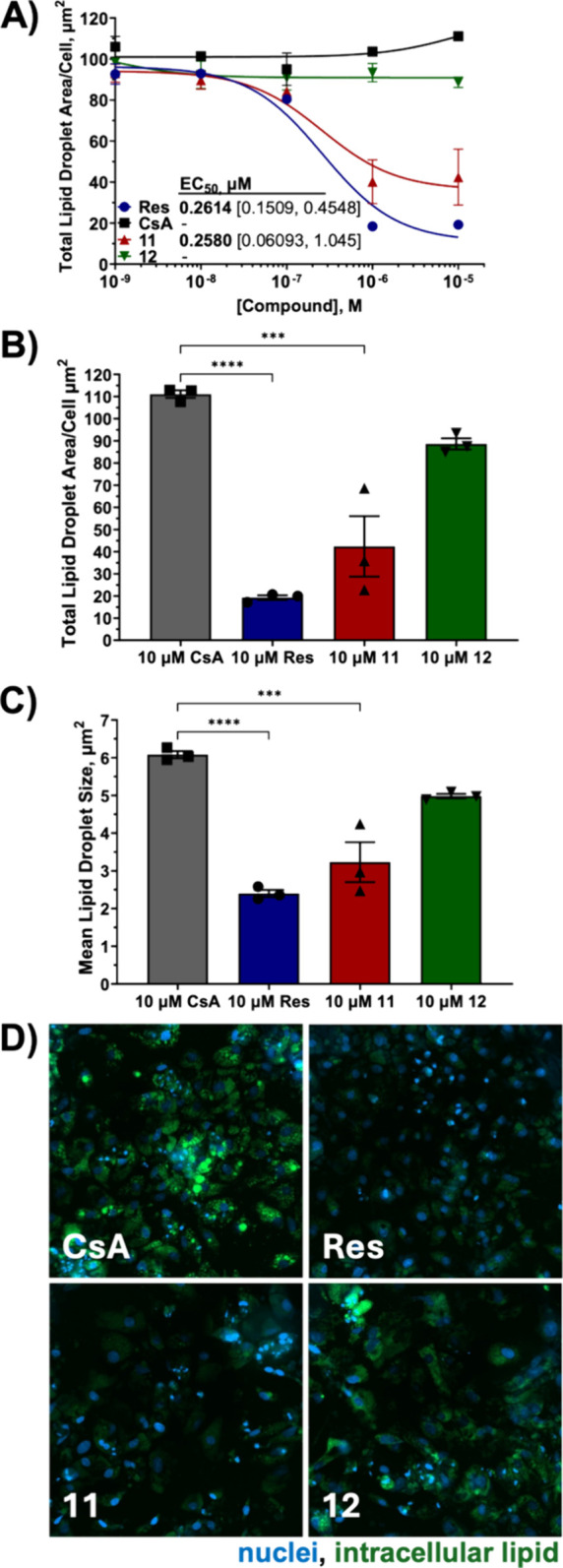
Effect of Compounds on Hepatocyte Steatosis after 48 h. **A)** Concentration response curve for steatosis after treatment with **CsA**, Resmetirom (**Res**), **11** and **12**; EC_50_ with 95% CI [lower limit, upper limit]. **B)** Total area of hepatocyte steatosis per cell. **C)** Mean lipid droplet size. **D)** Representative images 1 mm × 1 mm. Values in panels **A**-**C** are mean ± SEM from 3 independent experiments, each with 6 replicates; **** *p* < 0.0001, *** *p* < 0.001.

We also sought evidence of efficacy of selected compounds for the treatment of fatty liver disease. A recent publication demonstrated that knockout of Cyp B in mice, subjected to combined chemical and diet-induced MASH, significantly protected against lipid accumulation and fibrosis in the liver whereas knock out of Cyp A did not.^39^ Using induced pluripotent stem cell (iPSC)-derived hepatocytes, which demonstrate genotypic and phenotypic signatures consistent with the MASH phenotype when loaded with free fatty acids, we evaluated steatosis following 48 h treatment with the Cyp B-selective compounds **11** and **12** versus that seen with nonselective **CsA**. Compound **11**, which demonstrates ca. 7-fold greater affinity for Cyp B compared to **12**, showed good potency causing significant reduction in hepatocyte overall lipid levels (Figure 5A, B) and lipid droplet size (Figure 5C), whereas **CsA** did not. Intriguingly, this reduction was comparable to that induced by the FDA-approved MASH therapeutic Resmetirom (**Res**) in this assay (Figure 5A, 5D); EC_50_ values were similar (ca. 0.3 μM). However, Resmetirom seemed capable of lowering lipid levels to a greater extent compared to **11**. Resmetirom’s EC_50_ curve bottom was lower (Figure 5A) ca. 11 μm^2^ versus 36 μm^2^ per cell, and overall cellular lipid levels (Figure 5B) as well as mean lipid droplet size (Figure 5C) with 10 μM treatment were lower compared to **11**. Since, to our knowledge, the mechanism of action of tri-vector Cyp B-selective inhibitors and Resmetirom are independent, they could potentially be combined for increased efficacy.

### Drug Metabolism, Pharmacokinetics and Toxicity

The *in vitro* metabolism of compounds **1**, **3**, **10**, **11**, and **12** was assessed to establish the potential for further development of this series (Table 3). The water/octanol distribution coefficient values (Log D) were considered to be in an acceptable range. All compounds were very soluble at pH 7.4 but **3** was significantly less soluble in simulated intestinal fluid (SIF). **3** could not be detected in a simulated gastric fluid (SGF), suggesting that the annulated tetrahydropyran moiety may be chemically unstable in acidic conditions. Compound **1** showed encouraging Caco-2 apparent permeability but high efflux ratio. Compounds **10** and **11** improved on **1** with similar apparent permeability but decreased efflux ratio. By contrast compounds **3** and **12** showed significantly reduced Caco-2 apparent permeability and increased efflux. The low apparent permeability and high efflux of **12** may account for the lack of potency of this compound in the MASH hepatocyte assay. Conversely it is possible that compound **3** showed strong potency in the Calcium Retention Capacity assay because the HepG2 cells were permeabilized with digitonin prior to treatment. All compounds showed high clearance in human and rat liver microsomes. Compound **3** showed low clearance in human and rat hepatocytes which is consistent with its low passive permeability. Compounds **10**, **11**, **12** showed moderate clearance in human and rat hepatocytes. These results indicate that while the annulated tetrahydropyran moiety present in **3** confers excellent cyclophilin binding affinity (Table 1) it also renders the parent compound unable to enter cells. The relatively more rapid clearance of compounds **10**-**12** in liver microsomes than in hepatocytes may be an indication of CYP metabolism.^40^ Gratifyingly the strategies to alleviate genotoxicity of the aniline moiety through halogenation (**10**, **11**) or phenyl substitution by pyrimidine (**12**) were deemed successful as all three compounds were Ames negative in all strains tested, in absence and presence of liver S9 fractions.

**Table 3:** DMPK and Mutagenicity Data for a Panel of Compounds

	**1**	**3**	**10**	**11**	**12**
log D	1.39	0.66	1.74	1.53	1.27
solubility (PBS)/μM	194	>200	>200	>200	>200
solubility (SGF)/μM	>200	n.d	>200	>200	>200
solubility (SIF)/μM	>200	54	>200	>200	>200
Caco-2 A-B/10^–6^ cm/s	4	0.03	6.6	5	0.8
Efflux ratio	6.8	37	1.9	2.8	16
Rat Liver microsomes CL/μM/min/mg	-	137	238	352	449
Rat Liver microsomes *t*_1/2_/ min	-	51	29	20	15
Human Liver microsomes CL/μM/min/mg	-	169	344	558	604
Human Liver microsomes *t*_1/2_/ min	-	41	20	12	11
Rat Hepatocytes CL/μM/min/10^6^ cells	-	<8	<8	13	12
Rat Hepatocytes *t*_1/2_/ min	-	>120	>120	78	84
Human Hepatocytes CL/μM/min/10^6^ cells	-	<8	12	13	11
Human Hepatocytes *t*_1/2_/ min	-	>120	85	74	94
Ames fluctuation test (TA98 – S9/+S9)	-	-	neg/neg	neg/neg	neg/neg
Ames fluctuation test (TA100 – S9/+S9)	-	-	neg/neg	neg/neg	neg/neg
Ames fluctuation test (TA1535 – S9/+S9)	-	-	neg/neg	neg/neg	neg/neg
Ames fluctuation test (TA1537 – S9/+S9)	-	-	neg/neg	neg/neg	neg/neg

Further profiling (Table 4) indicated that compound **11** showed moderate (CYP2C8, CYP2C19, CYP3A) or weak (CYP1A, CYP2B6, CYP2C9, CYP2D6) inhibition of cytochrome P450s, indicating overall an encouraging drug–drug interaction profile. Compound **11** was measured in both Rat and Human plasma protein binding assays and there was no indication of plasma stability issues during the time-course of the study.

**Table 4:** Plasma Protein Binding and Cytochrome P450 Inhibition Measurements for Compound **11**^a^

Rat PPB free %	11	Human PPB free %	2
Rat plasma recovery %	99	Human plasma recovery %	88
CYP1A phenacetin %	25	CYP2B6 bupropion %	11
CYP2C8 amodiaquine %	71	CYP2C9 diclofenac %	45
CYP2C19 omeprazole %	72	CYP2D6 dextrometor. %	18
CYP3A midazolam %	73	CYP3A testoterone %	59

a Single point measurements at 10 μM.

## Conclusions

Cyclophilin inhibitors offer a promising potential therapeutic approach for the treatment of MASH and related liver diseases. The development of inhibitors that selectivity engage Cyp B over Cyp A is particularly interesting owing to the role played by Cyp B in liver fibrosis. To date only natural product derived nonisoform selective cyclophilin inhibitors have entered clinical studies.^12,18^ Earlier reports of potent small molecule Cyp A inhibitors,^41^ could not be subsequently replicated by other groups.^21^ Daum et al. reported arylindanyl ketone inhibitors that selectively inhibited Cyp A over Cyp B in a PPIase enzymatic assay with low micromolar dissociation constants,^42^ but as pointed out here, selective Cyp B or D inhibition over Cyp A is desirable in a MASH context. Potent and selective Cyp D inhibition was achieved by Petersson et al. via biphenyl dicarboxylate macrocyclic compounds, but a prodrug approach was necessary to deliver the compounds into cells and no DMPK data was reported.^16^ Ahmed-Belkacem et al. reported a series of phenyl-pyrrolidine urea compounds that show potent *in vitro* activity against Cyp A, Cyp B and Cyp D but isoform-selective inhibition was not reported.^21^ Shore et al. and Gräedler et al. later reported optimizations of this series into potent Cyp D inhibitors but did not report binding selectivity or DMPK and mutagenicity data to assert development potential for *in vivo* studies.^36,43^ These efforts underscore the challenges associated with developing potent and isoform-selective small molecule cyclophilin inhibitors. Here we used free energy calculations to optimize a class of tri-vector cyclophilin inhibitors that engage a less conserved accessory “3 o’clock” pocket. These studies informed the synthesis of compounds that achieved single digit nM potency for Cyp B, and up to 80-fold Cyp B/A selectivity in a Surface Plasmon Resonance Assay. The cellular activity of the series was confirmed in human HepG2 cells in addition to iPSC-derived hepatocytes which exhibit MASH-like characteristics. A series of four potent analogues was progressed to *in vitro* DMPK assays and mutagenicity assays. These experiments validated rational compound modifications introduced to alleviate genotoxicity risks. Compound **11** emerged overall as a potent Cyp B inhibitor with 10-fold selectivity over Cyp A, encouraging drug safety profile and *in vitro* DMPK properties, as well as efficacy in reducing hepatic steatosis; a key component of MASH disease. Thus, this compound series has the potential to be useful to investigate the therapeutic benefits of selective Cyp B inhibition in the context of MASH and related liver diseases.

## Experimental Section

### General Synthetic and Analytical Methods

Reagents were purchased from Fluorochem, Sigma-Aldrich and Acros and were used as supplied unless otherwise stated. Solvents and chemicals were purified according to standard procedures. Titration of *n*-butyllithium (*n*-BuLi) solutions was carried out to ascertain their exact concentration using 1,10-phenanthroline as an indicator. Tetrahydrofuran (THF), dichloromethane (DCM) and dimethyl sulfoxide (DMSO), Ethyl Acetate (EtOAc), Dimethylformamide (DMF), Diethyl Ether (D_2_O), Ethanol (EtOH) and Methanol (MeOH) were obtained from the *Inert* Solvent Purification System (SPS) or purchased as anhydrous solvents from Sigma-Aldrich. All anhydrous reactions were performed in flame-dried flasks under a positive pressure of dry nitrogen unless otherwise stated. Air- and moisture-sensitive compounds were introduced via syringes using standard inert atmosphere techniques. Temperature control during certain procedures was maintained using a Julabo chiller unit. Reactions were monitored by thin layer chromatography (TLC) using E. Merck silica gel plates, Kieselgel 60 F254 with 0.2 mm thickness. Components were visualized by illumination with short-wavelength ultraviolet light and/or staining.

Flash column chromatography was performed either manually with E. Merck silica gel 60 (230–400 mesh ASTM), or using either a Biotage IsoleraÔ or a Teledyne ISCO Combi*Flash* NextGen 300+ using Biotage Sfär or Teledyne ISCO RediSep silica gel flash columns. Fraction collection was based on UV detection. Analytical reverse phase HPLC analysis was performed using a Waters 600E (100 μL) gradient pump using a 717plus autosampler and a Waters 996 PDA equipped with a Phenomenex Luna C18(2), 5 μm, 250 × 4.6 mm column at a flow rate of 1 mL min^–1^. Semipreparative reverse phase HPLC was performed using a Waters 600 (225 μL) system using a Waters 486 tunable absorbance detector recording at 254 nm equipped with a Phenomonex Luna C18(2), 5 μm, 250 × 21.2 mm column at a flow rate of 21.2 mL min^–1^. Preparative HPLC was carried using a Waters HPLC comprising of a Waters 2767 Sample Manager, Waters 2545 Binary Gradient Module, Waters Systems Fluidics Organizer, Waters 515 ACD pump, Waters 2998 Photodiode Array Detector, using a Waters XBridge Prep OBD C18, 5 μm, 19 mm × 50 mm i.d. column and a flow rate of 20 mL/min. Compounds were purified using acidic reverse phase HPLC (water/acetonitrile/0.1% trifluoroacetic acid) using a standard gradient of 5% acetonitrile/95% water to 100% acetonitrile, or basic reverse phase HPLC (water/acetonitrile/0.01 M ammonia solution) using a standard gradient of 10% acetonitrile/90% water to 100% acetonitrile. Fraction collection was based on UV detection.

SFC separations were carried out using a Waters system comprising of a fluid delivery model, thermocube (set to 4 °C), autosampler, column oven (set to 40 °C), 2489 UV/visible detector (254 nm), fraction collection module, heat exchanger, and back pressure regulator. Semipreparative separations were achieved using Chiralpak (AS-H or AD-H) or Chiralcel (OD-H or OJ-H) columns (all 10 ′ 250 mm, 5 μm). Liquid CO_2_ was used as the bulk mobile phase.

^1^H and ^13^C NMR nuclear magnetic resonance spectra were recorded in deuteriochloroform (CDCl_3_) or deuterated DMSO (DMSO-*d*_6_) at ambient temperature (unless otherwise specified) on a Bruker Avance III 400 spectrometer, Bruker Avance III 500 (optimized for ^1^H/^13^C), Bruker Avance III 500 (optimized for ^1^H) or Bruker Avance III 600 operating at 400 MHz, 500 MHz, 500 or 600 MHz respectively for ^1^H, and at 75 MHz, 125 MHz, 125 or 150 MHz respectively for ^13^C. All spectra were calibrated at δ 7.26 ppm (residual CHCl_3_) or δ 2.50 ppm (residual DMSO) for ^1^H spectra, and δ 77.16 ppm (CHCl_3_) or δ 39.52 ppm (DMSO) for ^13^C spectra. Splitting patterns were designated as follows: s = singlet, d = doublet, t = triplet, q = quartet, m = multiplet, br = broad.

IR absorption spectra were recorded neat on a Shimadzu IRAffinity-1 spectrometer from 4000 to 400 cm^–1^. Electrospray (ESI) mass spectra were obtained on a Bruker 12 T SolariX, Bruker microTOF II or Kratos MS50TC mass spectrometer. LCMS samples were analyzed using an Agilent 1200 Series HPLC system and a 6140 single quadrupole mass spectrometer equipped with a multimode (APCI+ESI) source. The reverse phase HPLC was carried out using a Phenomenex Luna C18 (2)-HST column (2.5 μm, 50 ′ 2.0 mm) with monitoring at 254 and 220 nm. The column oven temperature was set to 45 °C. The column flow rate was set to 1.0 mL/min and the solvents used were 0.1% formic acid in water (A) and 0.1% formic acid in acetonitrile (B).

Method A: the method timetable was 0.00–0.50 min (1% B), 0.50–3.50 min (linear gradient from 1% to 100% B), and 3.50–4.25 min (100% B).

Method B: the method timetable was 0.00–0.50 min (1% B), 0.50–2.00 min (linear gradient from 1% to 100% B), and 2.00–3.25 min (100% B).

### General Procedure A: *N*-Alkylation

The benzylamine hydrochloride (1 equiv) was dissolved in acetonitrile (0.3 M) in a two-necked RBF and K_2_CO_3_ (2 equiv) was added to the mixture. The reaction was left to stir for 1 h at rt. After this time, the alkyl halide (1 equiv) was added and the mixture was stirred at 70 °C for 18 h. Upon completion of the reaction, the mixture was evaporated to dryness. The crude material was dissolved in EtOAc and was washed with NaOH (1 M aq), NaHCO_3_ (sat aq) and brine. The combined organic layers were dried over MgSO_4_, filtered and the solvent removed *in vacuo* to give the crude substituted compound. The final product was purified using flash column chromatography (hexane/EtOAc, 10:1 to 1:3).

### General Procedure B: Urea Formation

The *N*-alkylated amine from General Procedure A (1 equiv) was dissolved in DCM (0.12 M). Ethyl isocyanatoacetate (1 equiv) was added and the mixture was stirred at rt for 2–18 h. Upon completion of the reaction, the mixture was evaporated to dryness. The final product was purified by flash column chromatography (hexane/EtOAc, 10:1 to 1:5).

### General Procedure C: Ester Hydrolysis

The ethyl ester (1 equiv) was mixed with LiOH (1 M aq, 1 equiv) in a 2:2:1 ratio mixture of MeOH/THF/H_2_O (0.13 M) and stirred at rt for 1 h, while monitoring the reaction progression by TLC. Upon completion, the organic solvents were removed *in vacuo*, and the basic aqueous solution was washed with EtOAc to remove any impurities. The aqueous solution containing the carboxylic acid product was acidified to ∼ pH 5 using HCl (3 M aq), and the aqueous layer was extracted 3 times with EtOAc. The combined organic layers were dried over MgSO_4_, filtered and evaporated to dryness to give the final hydrolyzed product.

### General Procedure D: Nitro Group Reduction

The Nitrobenzylamine (1 equiv) was placed in a two-necked RBF and dissolved in anhydrous MeOH (0.065 M). Pd/C (10%, 0.05 equiv) and ZnBr_2_ (1 equiv) were added, and the flask was flushed with H_2_ gas 3 times. The reaction was stirred at rt for 18 h. After confirming its completion by TLC, the reaction mixture was passed through a Celite plug and the solvent was removed *in vacuo* and the crude product was used without further purification in the next reaction.

### General Procedure E: Amide Formation

The carboxylic acid (1 equiv) was placed in a two-necked RBF and was dissolved in DCM (0.075 M), followed by the addition of the pyrrolidine analogue (2.5 equiv). DIPEA (3 equiv) was added, and the reaction mixture was stirred for 30 min at rt. The reaction was cooled to 0 °C and propanephosphonic acid anhydride (1.5 equiv) was added dropwise. The reaction was allowed to gradually warm to rt overnight with stirring. Upon completion of the reaction, the solvent was removed *in vacuo* and the crude product was purified using preparative HPLC (H_2_O/MeCN + 0.1%TFA).

Final compounds **2**-**19** were determined to be >95% pure as determined by reverse phase LC/MS (c.f. Supporting Information), with the exception of compounds **8** and **9** which contained 6–8% of the corresponding 1-substituted regioisomer.

### Preparation of Compound 2

#### *N-[(2-methyltetrazol-5-yl)methyl]-1-(4-nitrophenyl)methanamine* (**22aa**)

(4-Nitrophenyl)methanamine (**20aa**) (86.0 mg, 0.570 mmol) and 5-(chloromethyl)-2-methyl-tetrazole (**21a**) (74.9 mg, 0.570 mmol) were dissolved in MeCN (5 mL) and potassium carbonate (156 mg, 1.13 mmol) followed by sodium iodide (0.85 mg, 0.010 mmol) added. The mixture was heated to 65 °C overnight. The reaction was filtered through a Celite pad which was washed with EtOAc and combined organics concentrated to dryness. The crude material was purified by flash column chromatography on the Biotage Isolera (12 g silica, 0 to 2% MeOH in DCM) to provide the amine **22aa** as yellow gum (86 mg, 61%). LCMS (Method B): 1.078 min, 249.2 [M + H]^+^. ^1^H NMR (400 MHz, CDCl_3_) δ 8.23–8.15 (m, 2H), 7.58–7.50 (m, 2H), 4.34 (s, 3H), 4.08 (s, 2H), 3.96 (s, 2H).

#### *Ethyl 2-({[(2-Methyl-2H-1,2,3,4-tetrazol-5-yl)methyl][(4-nitrophenyl)methyl]carbamoyl} amino)acetate* (**23aa**)

Amine **22a** (86 mg, 0.35 mmol) was reacted according to General Procedure B. The crude product was purified by flash column chromatography on the Biotage Isolera (12 g silica, 0 to 3.5% MeOH in DCM) to provide urea **23aa** as a yellow gum (127 mg, 92%). HRMS (ESI) [M + H]^+^ found 378.3695, C_15_H_20_N_7_O_5_ requires 378.3695.

#### *2-({[(2-Methyl-2H-1,2,3,4-tetrazol-5-yl)methyl][(4-aminophenyl)methyl]carbamoyl} nitro)acetic acid* (**24aa**)

Urea ester **23aa** (63 mg, 0.17 mmol) was reacted according to General Procedure C to afford acid **24aa** as a viscous orange oil (58 mg, 0.17 mmol, 99%). HRMS (ESI) [M + H]^+^ found 350.1210, C_13_H_15_N_7_O_5_ requires 350.1210.

#### *2-[[(4-Aminophenyl)methyl-[(2-methyltetrazol-5-yl)methyl]carbamoyl]amino]acetic acid* (**25aa**)

To an argon purged flask containing 10% Pd/C (4.0 mg, 0.040 mmol) was added acid **24aa** (22 mg, 0.060 mmol) in MeOH (1 mL). The vessel was purged with hydrogen and stirred under pressure of a balloon for 2.5 h. The reaction was filtered through a filter tip and concentrated to dryness to give aniline **25aa** as a yellow gum (19 mg, 95%). ^1^H NMR (400 MHz, MeOD) δ 7.15–7.07 (m, 2H), 6.82–6.74 (m, 2H), 4.63 (s, 2H), 4.49 (s, 2H), 4.30 (s, 3H), 3.90 (s, 2H).

#### *1-[(4-Aminophenyl)methyl]-1-[(2-methyltetrazol-5-yl)methyl]-3-[2-oxo-2-[(2R)-2-(2-methylsulfanylphenyl)pyrrolidin-1-yl]ethyl]urea* (**2**)

Aniline acid **25aa** (47 mg, 0.15 mmol) and (2*R*)-2-(2-methylsulfanylphenyl)pyrrolidine (*R*)-(**26**) (0.25 mL, 0.37 mmol) were reacted according to General Procedure E. The crude product was purified by flash column chromatography on the Biotage Isolera (12 g silica, 0 to 5% MeOH/DCM) to provide compound **2** as colorless frothy solid (40 mg, 0.081 mmol, 52%). LCMS (Method B): 1.85 min (100% @ 254 nm), 495.2 [M + H]^+^. ^1^H NMR (400 MHz, DMSO) δ 7.36–7.15 (m, 2.49H), 7.09–6.98 (m, 1.66H), 6.93–6.88 (m, 2H), 6.56 (t, *J* = 5.2 Hz, 0.59H), 6.52–6.45 (m, 2.41H), 5.24 (m, 1H), 4.97 (m, 1.75H), 4.60–4.40 (m, 2H), 4.33–4.20 (m, 5H), 3.97–3.93 (m, 1H), 3.90–3.48 (m, 2.49H), 3.01 (dd, *J* = 16.7, 5.1 Hz, 0.44H), 2.53 (m, 3H), 2.39–2.27 (m, 0.48H), 2.16 (m, 0.6H), 1.97–1.59 (m, 3H).

### Preparation of Compound 3

#### *1-[(1S,9R,10S)-10-[tert-Butyl(dimethyl)silyl]oxy-12-oxa-8-azatricyclo[7.3.1.0,*^27^*]trideca-2,4,6-trien-4-yl]-N-[(2-methyltetrazol-5-yl)methyl]methanamine* (**22ba**)

[(1*S*,9*R*,10*S*)-10-[*tert*-Butyl(dimethyl)silyl]oxy-12-oxa-8-azatricyclo [7.3.1.0,^27^]trideca-2,4,6-trien-4-yl]methanamine (**20b**) (280 mg, 0.84 mmol) and 5-(chloromethyl)-2-methyl-tetrazole (111 mg, 0.840 mmol) were reacted according to General Procedure A. The crude reaction was filtered through Celite which was washed with EtOAc (10 mL). Combined organics were concentrated to dryness and the crude product purified by flash column chromatography on the Biotage Isolera (20 g silica, 0 to 5% 2 M NH_3_-MeOH/DCM) to provide amine **22ba** as a light-colored gum (254 mg, 0.799 mmol, 70%). HRMS (ESI) [M + H]^+^ found 431.6402, C_21_H_34_N_6_O_2_Si requires 431.6402.^1^H NMR (400 MHz, CDCl_3_) δ 7.11 (d, *J* = 7.9 Hz, 2H), 6.53–6.46 (m, 1H), 4.72–4.66 (m, 1H), 4.32 (s, 4H), 4.06 (s, 2H), 3.72 (s, 2H), 3.52–3.43 (m, 3H), 3.38 (dd, *J* = 13.0, 1.9 Hz, 1H), 2.67 (dt, *J* = 12.7, 2.7 Hz, 1H), 1.51–1.42 (m, 1H), 0.93 (s, 9H), 0.08 (d, *J* = 3.8 Hz, 6H).

#### *Ethyl 2-[[[(1S,9R,10S)-10-[tert-Butyl(dimethyl)silyl]oxy-12-oxa-8-azatricyclo[7.3.1.0,*^27^*] trideca-2,4,6-trien-4-yl]methyl-[(2-methyltetrazol-5-yl)methyl]carbamoyl]amino]acetate* (**23ba**)

Amine **22ba** (254 mg, 0.590 mmol) was reacted according to General Procedure B. The crude reaction was concentrated directly on to silica and purified by flash column chromatography on the Biotage Isolera (12 g silica, 0 to 3% EtOAc/Heptane) to provide urea **23ba** as a colorless gum (330 mg, 0.601 mmol, 100%). LCMS (Method B): 1.988 min, 560.2 [M + H]^+^. ^1^H NMR (400 MHz, CDCl_3_) δ 7.03 (dd, *J* = 8.2, 2.2 Hz, 1H), 6.98 (d, *J* = 2.1 Hz, 1H), 6.43 (d, *J* = 8.2 Hz, 1H), 5.36 (s, 1H), 4.60 (s, 3H), 4.39 (q, *J* = 15.9 Hz, 2H), 4.24 (s, 4H), 4.10 (q, *J* = 7.1 Hz, 2H), 3.94 (dd, *J* = 5.2, 2.7 Hz, 2H), 3.44–3.36 (m, 3H), 3.28 (dd, *J* = 13.0, 1.9 Hz, 1H), 2.58 (d, *J* = 12.7 Hz, 1H), 1.38 (d, *J* = 13.1 Hz, 1H), 1.18 (t, *J* = 7.1 Hz, 3H), 0.85 (s, 9H), 0.01 (m, 6H).

#### *1-[[(1S,9R,10S)-10-[tert-Butyl(dimethyl)silyl]oxy-12-oxa-8-azatricyclo[7.3.1.0,*^27^*] trideca-2,4,6-trien-4-yl]methyl]-3-[2-[(2R)-2-(2-methylsulfanylphenyl) pyrrolidin-1-yl]-2-oxo-ethyl]-1-[(2-methyltetrazol-5-yl)methyl]urea* (*R*)-(**28ba**)

Urea ester **23ba** (101 mg, 0.180 mmol) was subjected to the conditions described in General Procedure C. The resultant 2-[[[(1*S*,9*R*,10*S*)-10-[*tert*-butyl(dimethyl)silyl]oxy-12-oxa-8-azatricyclo[7.3.1.0,^27^]trideca-2,4,6-trien-4-yl]methyl-[(2-methyltetrazol-5-yl)methyl] carbamoyl]amino]acetate (**25ba**) was used in its crude form for the subsequent reaction. In a flask, the crude lithium salt of **25ba** (96.8 mg, 0.180 mmol) was reacted with (2*R*)-2-(2-methylsulfanylphenyl)pyrrolidine (*R*)-(**26**) (87 mg, 0.45 mmol). After completion, the reaction mixture was concentrated, and the residue was preabsorbed onto silica. Purification was achieved using flash column chromatography on the Biotage Isolera, employing a gradient of 0 to 4% MeOH/DCM on 12 g of silica. This yielded amide (*R*)-**28ba** as a light-colored gum (121 mg, 0.166 mmol, 92%). LCMS (Method B): 2.109 min, 729.2 [M + Na]^+^. ^1^H NMR (400 MHz, CDCl_3_) δ 7.18–7.09 (m, 2H), 7.08–6.79 (m, 4H), 6.39 (dd, *J* = 10.8, 8.2 Hz, 1H), 5.78 5.64 (m, 1H), 5.41 (dd, *J* = 8.2, 2.6 Hz, 0.35H), 5.21 (m, 1H), 4.68–4.50 (m, 3H), 4.45–4.26 (m, 2H), 4.26–4.17 (m, 4H), 4.11–3.98 (m, 1.4H), 3.79–3.56 (m, 1.8H), 3.55–3.46 (m, 0.39H), 3.42–3.33 (m, 3H), 3.31–3.21 (m, 1.75H), 2.56 (m, 1H), 2.42 (m, 3H), 2.35–2.09 (m, 1H), 1.95–1.70 (m, 2H), 1.40–1.30 (m, 1H), 0.85 (s, 9H), 0.01 (s, 3H), 0.01 (s, 3H).

#### *1-[[(1S,9R,10S)-10-Hydroxy-12-oxa-8-azatricyclo[7.3.1.0,*^27^*]trideca-2,4,6-trien-4-yl]methyl]-3-[2-[(2R)-2-(2-methylsulfanylphenyl)pyrrolidin-1-yl]-2-oxo-ethyl]-1-[(2-methyltetrazol-5-yl)methyl]urea* (**3**)

Amide (*R*)-**28ba** (121 mg, 0.170 mmol) was placed in a vial which was purged with argon and dry THF (6 mL) added. Tetrabutylammonium fluoride (0.34 mL, 0.34 mmol) was added dropwise and the solution stirred overnight at room temperature. An additional 170 μL TBAF was added and stirring continued for 2 h. The reaction was concentrated directly on to silica and purified by flash column chromatography on the Biotage Isolera (12 g silica, 0 to 5% 2 M-NH_3_ MeOH/DCM) then purified by reverse phase column chromatography (Xbridge C18, 10–95% MeCN/H_2_O with NH_4_OH modifier) to provide compound **3** as colorless frothy solid (30 mg, 0.048 mmol, 29%). LCMS (Method B): 2.08 min (100% @ 254 nm), 615.0 [M + Na]^+^. ^1^H NMR (400 MHz, DMSO) δ 7.38–7.14 (m, 2.45H), 7.12–6.98 (m, 1.62H), 6.98–6.92 (m, 1H), 6.92–6.86 (m, 1H), 6.64–6.51 (m, 1H), 6.51–6.43 (m, 1H), 6.39–6.33 (m, 1H), 5.30–5.21 (m, 1H), 4.99–4.94 (m, 1H), 4.59–4.44 (m, 3H), 4.34–4.24 (m, 5H), 4.02–3.94 (m, 1H), 3.91–3.49 (m, 2.43H), 3.41–3.33 (m, 2H), 3.29–3.25 (m, 1H), 3.17–3.09 (m, 1H), 3.06–2.96 (m, 0.44H), 2.56–2.52 (m, 3H), 2.48–2.28 (m, 1.51H), 2.24–2.10 (m, 0.60H), 2.00–1.58 (m, 3H), 1.28–1.20 (m, 1H).

### Preparation of Compound 4

#### *1-[[(1S,9R,10S)-10-Hydroxy-12-oxa-8-azatricyclo[7.3.1.0,*^27^*]trideca-2,4,6-trien-4-yl]methyl]-3-[2-[(2S)-2-(2-methylsulfanylphenyl)pyrrolidin-1-yl]-2-oxo-ethyl]-1-[(2-methyltetrazol-5-yl)methyl]urea* (*S*)-(**28ba**)

Urea ester **23ba** (101 mg, 0.180 mmol) was subjected to the conditions described in General Procedure C. The resultant 2-[[[(1*S*,9*R*,10*S*)-10-[*tert*-butyl(dimethyl)silyl]oxy-12-oxa-8-azatricyclo[7.3.1.0,^27^]trideca-2,4,6-trien-4-yl]methyl-[(2-methyltetrazol-5-yl)methyl] carbamoyl]amino]acetate (**25ba**) was used in its crude form in the subsequent reaction. In a flask, the crude lithium salt of **25ba** (129 mg, 0.240 mmol) was reacted with (2*S*)-2-(2-methylsulfanylphenyl)pyrrolidine (*S*)-(**26**) (116 mg, 0.600 mmol) according to General Procedure E. The crude reaction was concentrated directly onto silica and purified by flash column chromatography on the Biotage Isolera (12 g silica, 0 to 2% MeOH/DCM) to provide the amide (*S*)-**28ba** as a light-colored gum (127 mg, 0.174 mmol, 67%). LCMS (Method B): 2.109 min, 729.2 [M + Na]^+^. ^1^H NMR (400 MHz, MeOD) δ 7.40–7.25 (m, 1.76H), 7.24–7.14 (m, 1H), 7.12–6.98 (m, 2.26H), 6.97–6.91 (m, 1H), 6.55–6.48 (m, 1H), 5.48–5.40 (m, 1H), 4.68–4.52 (m, 3H), 4.50–4.33 (m, 2H), 4.30–4.23 (m, 3H), 4.12–4.06 (m, 1H), 4.00–3.92 (m, 0.53H), 3.92–3.63 (m, 2H), 3.52–3.46 (m, 1H), 3.45–3.37 (m, 2H), 3.37–3.31 (m, 1H), 3.28–3.19 (m, 0.56H), 2.65–2.55 (m, 1H), 2.55–2.48 (m, 3H), 2.46–2.20 (m, 1H), 2.03–1.74 (m, 3H), 1.43–1.31 (m, 1H), 0.98–0.92 (m, 9H), 0.14–0.07 (m, 6H).

#### *1-[[(1S,9R,10S)-10-Hydroxy-12-oxa-8-azatricyclo[7.3.1.0,*^27^*]trideca-2,4,6-trien-4-yl]methyl]-3-[2-[(2S)-2-(2-methylsulfanylphenyl)pyrrolidin-1-yl]-2-oxo-ethyl]-1-[(2-methyltetrazol-5-yl)methyl]urea* (**4**)

Amide (*S*)-(**28ba**) (127 mg, 0.180 mmol) was placed in a vial which was purged with argon and dry THF (4 mL) added. Tetrabutylammonium fluoride (0.36 mL, 0.36 mmol) was added dropwise and the solution stirred overnight at room temperature. The reaction was concentrated directly on to silica and purified by flash column chromatography on the Biotage Isolera (12 g silica, 0 to 5% 2 M-NH_3_ MeOH/DCM) then purified by reverse phase column chromatography (Xbridge C18, 10–95% MeCN/H_2_O with NH_4_OH modifier) to provide compound **4** as colorless gummy solid (47 mg, 0.076 mmol, 43%). LCMS (Method A): 2.09 min (100% @ 254 nm), 615.0 [M + Na]^+^. ^1^H NMR (400 MHz, DMSO) δ 7.37–7.13 (m, 2.45H), 7.10–6.96 (m, 1.65H), 6.96–6.90 (m, 1H), 6.90–6.85 (m, 1H), 6.63–6.51 (m, 1H), 6.49–6.41 (m, 1H), 6.38–6.31 (m, 1H), 5.29–5.20 (m, 1H), 4.98–4.92 (m, 1H), 4.58–4.42 (m, 3H), 4.32–4.19 (m, 5H), 3.98–3.93 (m, 1H), 3.89–3.49 (m, 2.46H), 3.41–3.32 (m, 2H), 3.28–3.23 (m, 1H), 3.16–3.07 (m, 1H), 3.06–2.96 (m, 0.46H), 2.55–2.51 (m, 3H), 2.48–2.28 (m, 1.56H), 2.23–2.09 (m, 0.69H), 1.98–1.59 (m, 3H), 1.27–1.17 (m, 1H).

### Preparation of Compound 5

#### *6-Difluoro-4-[[(2-methyltetrazol-5-yl)methylamino]methyl]aniline* (**22ca**)

4-(Aminomethyl)-2,6-difluoro-aniline (**20c**) (77 mg, 0.49 mmol) and 5-(chloromethyl)-2-methyl-tetrazole (**21a**) (64.5 mg, 0.490 mmol) were reacted according to General Procedure A. After the mixture was stirred at 70 °C for 18 h, an additional 0.5 eq of tetrazole was added and heating continued for a further 8 h then cooled to room temperature and allowed to stand overnight. The reaction was concentrated directly onto silica and purified by flash column chromatography on the Biotage Isolera (12 g silica, 0 to 10% MeOH/DCM) to provide amine **22ca** as yellow gum (35 mg, 0.14 mmol, 28%). HRMS (ESI) [M + H]^+^ found 255.1212, C_10_H_12_F_2_N_6_ requires 255.1212. ^1^H NMR (400 MHz, CDCl_3_) δ 6.88–6.83 (m, 2H), 4.36 (s, 3H), 4.06 (s, 2H), 3.74 (d, *J* = 0.7 Hz, 2H), 3.69 (s, 2H).

#### *Ethyl 2-[[(4-Amino-3,5-difluoro-phenyl)methyl-[(2-methyltetrazol-5-yl)methyl] carbamoyl]amino]acetate* (**23ca**)

Amine **22ca** (66.0 mg, 0.26 mmol) was reacted according to General Procedure B to give the urea **23ca** as a colorless solid (94 mg, 0.25 mmol, 95%). LCMS (Method B): 1.465 min, 384.2 [M + H]^+^. ^1^H NMR (400 MHz, CDCl_3_) δ 6.88–6.77 (m, 2H), 5.55 (s, 1H), 4.63 (s, 2H), 4.48 (s, 2H), 4.34 (s, 3H), 4.20 (q, *J* = 7.1 Hz, 2H), 4.03 (d, *J* = 5.2 Hz, 2H), 3.70 (s, 2H), 1.28 (t, *J* = 7.2 Hz, 4H).

#### *1-[(4-Amino-3,5-difluoro-phenyl)methyl]-1-[(2-methyltetrazol-5-yl)methyl]-3-[2-oxo-2-[(2R)-2-(2-methylsulfanylphenyl)pyrrolidin-1-yl]ethyl]urea* (**5**)

Urea ester **23ca** (61 mg, 0.16 mmol) was reacted according to General Procedure C to give 2-[[(4-amino-3,5-difluoro-phenyl)methyl-[(2-methyltetrazol-5-yl)methyl]carbamoyl] amino]acetic acid (**25ca**) as a viscous yellow oil that was used in the next reaction without further purification. The crude acid **25ca** (32 mg, 0.090 mmol) and (2*R*)-2-(2-Methylsulfanylphenyl) pyrrolidine (*R*)-(**26**) (34.8 mg, 0.180 mmol) were reacted according to General Procedure D to give a yellow gum (40 mg). The crude material was purified by flash column chromatography on the Biotage Isolera (4 g silica, 0 to 2% NH_3_-MeOH/DCM) to provide compound **5** as beige gum (15 mg, 0.028 mmol, 18%). LCMS (Method A): 2.36 min (100% @ 254 nm), 531.2 [M + H]^+^. ^1^H NMR (400 MHz, DMSO) δ 7.38–7.14 (m, 2.48H), 7.11–6.97 (m, 1.60H), 6.89–6.76 (m, 2H), 6.69–6.55 (m, 1H), 5.29–5.21 (m, 1H), 5.13–5.05 (m, 2H), 4.62–4.58 (m, 1.17H), 4.58–4.48 (m, 0.76H), 4.38–4.27 (m, 5H), 3.99–3.93 (m, 1H), 3.89–3.64 (m, 1.45H), 3.64–3.53 (m, 0.44H), 3.06–2.96 (m, 0.44H), 2.56–2.52 (m, 3H), 2.42–2.29 (m, 0.55H), 2.23–2.10 (m, 0.63H), 1.98–1.60 (m, 3H).

### Preparation of Compound 6

#### *2,6-Difluoro-4-[(prop-2-ynylamino)methyl]aniline* (**22ce**)

4-(Aminomethyl)-2,6-difluoro-aniline (**20c**) (442 mg, 2.79 mmol) and propargyl bromide (**21e**) were reacted according to General Procedure A. The crude reaction mixture was filtered through dicalite which was washed with EtOAc. Combined filtrates were concentrated to dryness and purified by flash column chromatography on the Biotage Isolera (40 g silica, 0 to 5% MeOH/DCM) to provide amine **22ce** as yellow gum (203 mg, 1.04 mmol, 37%). HRMS (ESI) [M + H]^+^ found 197.0910, C_10_H_10_F_2_N_2_ requires 197.0910. ^1^H NMR (400 MHz, CDCl_3_) δ 6.90–6.77 (m, 2H), 3.78–3.73 (m, 2H), 3.66 (s, 2H), 3.39 (d, *J* = 2.4 Hz, 2H), 2.25 (t, *J* = 2.4 Hz, 1H).

#### *Ethyl 2-[[(4-Amino-3,5-difluoro-phenyl)methyl-prop-2-ynyl-carbamoyl]amino] acetate* (23ce)

Amine **22ce** (203 mg, 1.03 mmol) was reacted according to General Procedure B. Then an additional 20 μL of isocyanate was added in portions of 5 μL until the reaction was deemed complete by TLC. The crude reaction mixture was concentrated to dryness and urea **23ce** used without further purification (343 mg, 1.05 mmol, 98%). LCMS (Method B): 1.529 min, 326.2 [M + H]^+^. ^1^H NMR (400 MHz, CDCl3) δ 6.86–6.75 (m, 2H), 5.14 (t, *J* = 4.9 Hz, 1H), 4.44 (t, *J* = 0.7 Hz, 2H), 4.20 (q, *J* = 7.1 Hz, 2H), 4.07–3.99 (m, 4H), 3.71 (s, 2H), 2.30 (t, *J* = 2.5 Hz, 1H), 1.27 (t, *J* = 7.1 Hz, 3H).

#### *Ethyl 2-[[(4-Amino-3,5-difluoro-phenyl)methyl-[(1-methyltriazol-4-yl)methyl]carbamoyl] amino]acetate* (**23cg**)

To a flask containing DMF (2 mL) and water (0.5 mL) was added MeI (26 μL, 0.42 mmol) followed by NaN_3_ (31.2 mg, 0.480 mmol), Na_2_CO_3_ (136 mg, 1.28 mmol), l-Ascorbic acid (Sodium salt) (50.7 mg, 0.260 mmol), CuSO_4_·5H_2_ O (31.9 mg, 0.130 mmol) and crude urea **23ce** (104 mg, 0.320 mmol). The reaction was stirred at room temperature overnight. The crude reaction was quenched by addition of NH_4_ Cl (sat aq) and extracted into EtOAc (25 mL x 2). Combined organics were dried over Na_2_SO_4_, filtered and concentrated to give crude product as a yellow gum which was purified by flash column chromatography on the Biotage Isolera (12 g silica, 0 to 5% MeOH/DCM) to provide triazole **23cg** as colorless gum (65 mg, 0.17 mmol, 53%). LCMS (Method B): 1.398 min, 383.2 [M + H]^+^. ^1^H NMR (400 MHz, CDCl3) δ 7.41 (s, 1H), 6.82–6.69 (m, 2H), 5.56 (s, 1H), 5.29 (d, *J* = 0.5 Hz, 1H), 4.47 (s, 2H), 4.42 (s, 2H), 4.18 (q, *J* = 7.2 Hz, 2H), 4.05 (s, 3H), 3.98 (d, *J* = 5.4 Hz, 2H), 3.72 (s, 2H), 1.25 (t, *J* = 7.1 Hz, 3H).

#### *1-[(4-Amino-3,5-difluoro-phenyl)methyl]-1-[(1-methyltriazol-4-yl)methyl]-3-[2-oxo-2-[(2R)-2-(2-methylsulfanylphenyl)pyrrolidin-1-yl]ethyl]urea* (**6**)

Urea ester **22cg** (65 mg, 0.17 mmol) was reacted according to General Procedure C to give crude 2-[[(4-amino-3,5-difluoro-phenyl)methyl-[(1-methyltriazol-4-yl)methyl]carbamoyl] amino]acetate (**25cg**) which was used as obtained without further purification. The crude lithium salt of **25cg** (61 mg, 0.17 mmol) was reacted with (*R*)-2-(2-methylsulfanylphenyl) pyrrolidine (*R*)-(**26**) (39 mg, 0.20 mmol) according to General Procedure E to give a yellow gum (65 mg). The crude product was purified by flash column chromatography on the Biotage Isolera (12 g silica, 0 to 4% MeOH/DCM) to provide compound **6** as an off white frothy solid (47 mg, 0.089 mmol, 52%). LCMS (Method A): 2.25 min (100% @ 254 nm), 530.2 [M + H]^+^. ^1^H NMR (400 MHz, DMSO) δ 7.93–7.85 (m, 1H), 7.38–7.14 (m, 2.46H), 7.11–6.97 (m, 1.62H), 6.86–6.73 (m, 2H), 6.66–6.50 (m, 1H), 5.29–5.22 (m, 1H), 5.11–5.01 (m, 2H), 4.36–4.16 (m, 4H), 4.00–3.94 (m, 4H), 3.90–3.65 (m, 1.47H), 3.58 (m, 1H), 3.02 (m, 0.45H), 2.53 (m, 3H), 2.41–2.29 (m, 0.54H), 2.17 (m, 0.61H), 1.98–1.60 (m, 3H).

### Preparation of Compound 7

#### *1-[(4-Amino-3,5-difluoro-phenyl)methyl]-3-[2-oxo-2-[(2R)-2-(2-methylsulfanyl phenyl)pyrrolidin-1-yl]ethyl]-1-prop-2-ynyl-urea* (**7**)

Crude urea ester **23ce** (68 mg, 0.21 mmol) was reacted according to General Procedure C to give 2-[[(4-amino-3,5-difluoro-phenyl)methyl-prop-2-ynyl-carbamoyl]amino]acetate (63 mg, 0.21 mmol) (**25ce**) which was used without further purification. The crude lithium salt of acid **25ce** (63 mg, 0.21 mmol) and (2*R*)-2-(2-methylsulfanylphenyl)pyrrolidine (*R*)-(**26**) (61 mg, 0.31 mmol) were reacted according to General Procedure E to give a yellow gum (104 mg). The crude product was purified by flash column chromatography on the Biotage Isolera (12 g silica, 0 to 3% MeOH/DCM) then by reverse phase column chromatography (Xbridge C18, 10–95% MeCN/H_2_O with NH_4_OH modifier) to provide compound **7** as colorless froth (26.5 mg, 0.056 mmol, 27%). LCMS (Method A): 2.46 min (100% @ 254 nm), 473.2 [M + H]^+^. ^1^H NMR (400 MHz, DMSO) δ 7.38–7.15 (m, 2.41H), 7.12–6.98 (m, 1.62H), 6.86–6.76 (m, 2H), 6.64–6.49 (m, 1H), 5.30–5.21 (m, 1H), 5.11–5.06 (m, 2H), 4.34 (s, 1.17H), 4.29 (s, 0.77H), 4.00–3.79 (m, 4H), 3.79–3.66 (m, 0.66H), 3.64–3.53 (m, 1H), 3.18–3.11 (m, 1H), 3.05–2.95 (m, 0.46H), 2.54 (s, 3H), 2.41–2.10 (m, 1H), 2.00–1.58 (m, 3H).

### Preparation of Compound 8

#### *2-{[(4-Nitrophenyl)methyl]amino}acetonitrile* (**22af**)

4-Nitrobenzylamine hydrochloride (**20a**) (10.0 g, 53.2 mmol) was reacted with chloroacetonitrile (**21f**) (3.36 mL, 53.2 mmol, 1.00 equiv) according to General Procedure A to afford the amine **22af** as a brown oil (6.71 g, 35.1 mmol, 66%). HRMS (ESI) [M + H]^+^ found 191.1915, C_9_H_10_N_3_O_2_ requires 191.1903. ^1^H NMR (400 MHz, CDCl_3_) δ 8.21 (2H, d, *J* = 8.7 Hz), 7.55 (2H, d, *J* = 8.7 Hz), 4.05 (2H, s), 3.60 (2H, s), 1.71 (1H, br. s). ^13^C NMR (126 MHz, CDCl_3_) δ 147.6, 145.3, 128.9, 123.8, 117.1, 51.5, 36.5.

#### *Ethyl 2-{[(Cyanomethyl)[(4-nitrophenyl)methyl]carbamoyl]amino}acetate* (**23af**)

Amine **22af** (0.90 g, 4.8 mmol) was reacted according to General Procedure B to afford urea **23af** as a brown oil (1.3 g, 4.3 mmol, 90%). HRMS (ESI) [M + H]^+^ found 321.1230, C_14_H_17_N_4_O_5_ requires 321.1225. ^1^H NMR (400 MHz, CDCl_3_) δ 8.25 (2H, d, *J* = 8.7 Hz), 7.50 (2H, d, *J* = 8.7 Hz), 5.19 (1H, t, *J* = 5.1 Hz), 4.68 (2H, s), 4.32 (2H, s), 4.27 (2H, q, *J* = 7.2 Hz), 3.99 (2H, d, *J* = 5.1 Hz), 1.31 (3H, t, *J* = 7.2 Hz). ^13^C NMR (126 MHz, CDCl_3_) δ 170.4, 156.5, 147.9, 142.4, 127.7, 124.4, 115.4, 61.7, 50.8, 42.9, 35.8, 14.1.

#### *Ethyl 2-({[(4-Nitrophenyl)methyl][(1H-1,2,3,4-tetrazol-5-yl)methyl]carbamoyl} amino)acetate* (**23ah**)

To a 100 mL RBF equipped with a stir bar was added urea nitrile **23af** (780 mg, 2.37 mmol), DMF (14.0 mL, 0.18 M), NaN_3_ (278 mg, 4.27 mmol, 1.80 equiv), and NH_4_Cl (620 mg, 4.50 mmol, 1.90 equiv). The reaction vessel was stirred at 90 °C for 18 h. The reaction was cooled to rt and diluted with HCl (50.0 mL, 1 M aq), then extracted with EtOAc (3 × 20 mL). The organic phase was collected, dried over MgSO_4_ and filtered. Then solvent was removed *in vacuo* to give tetrazole **23ah** as a brown oil (300 mg, 0.830 mmol, 35%). HRMS (ESI) [M + H]^+^ found 364.1412, C_14_H_18_N_7_O_5_ requires 364.1419. ^1^H NMR (400 MHz, CDCl_3_) δ 8.07 (2H, d, *J* = 8.5 Hz), 7.42 (2H, d, *J* = 8.5 Hz), 6.10 (1H, t, *J* = 5.4 Hz), 4.82 (2H, s), 4.70 (2H, s), 4.10 (2H, q, *J* = 7.1 Hz), 3.96 (2H, d, *J* = 5.4 Hz), 1.19 (3H, t, *J* = 7.1 Hz).

#### *Ethyl 2-({[(2-Cyclopentyl-2H-1,2,3,4-tetrazol-5-yl)methyl][(4-nitrophenyl)methyl] carbamoyl}amino)acetate* (**23ai**)

Tetrazole **23ah** (2.87 g, 7.90 mmol) was dissolved in DMF (79.0 mL, 0.10 M) in a two-necked RBF and K_2_CO_3_ (2.15 g, 15.8 mmol, 2.00 equiv) was added. The reaction was stirred at 50 °C, for 1 h and was then cooled to 0 °C. Cyclopentyl bromide (1.20 mL, 11.9 mmol, 1.50 equiv) was added dropwise and the mixture was left to reach room temperature and was stirred for 18 h. The solvent was removed *in vacuo* to give a ∼ 50:50 regioisomeric mixture of 1- and 2-alkylated tetrazoles. The crude material was purified using flash chromatography (hexane/EtOAc, 4:1) to afford predominantly the faster-eluting 2-substituted tetrazole **23ai** as an orange oil (634 mg, 1.47 mmol, 19%). HRMS (ESI) [M + H]^+^ found 432.46781, C_19_H_25_N_7_O_5_ requires 432.4679. ^1^H NMR (500 MHz, CDCl_3_) δ 8.19 (2H, d, *J* = 8.7 Hz), 7.49 (2H, d, *J* = 8.7 Hz), 5.89 (1H, t, *J* = 5.2 H), 5.21 (1H, *J* = 7.7, 5.4 Hz), 4.76 (2H, s), 4.65 (2H, s), 4.23 (2H, q, *J* = 7.1 Hz), 4.07 (1H, d, *J* = 5.2 Hz), 2.29–2.21 (2H, m), 2.21–2.12 (2H, m), 1.99–1.86 (2H, m), 1.82–1.72 (2H, m), 1.31 (3H, t, *J* = 7.1 Hz).

#### *2-({[(2-Methyl-2H-1,2,3,4-tetrazol-5-yl)cyclopropyl][(4-anilino)methyl]carbamoyl} amino)acetic acid* (**25ai**)

Urea ester (**23ai**) (634 mg, 1.47 mmol) was hydrolyzed according to General Procedure C, to afford 2-({[(2-Methyl-2*H*-1,2,3,4-tetrazol-5-yl)cyclopropyl][(4-nitro)methyl]carbamoyl}amino)acetic acid (**24ai**) as a thick yellow oil (457 mg, 1.13 mmol). The crude acid **24ai** was reacted according to General Procedure D to afford aniline **25ai** as a viscous, yellow oil (377 mg, 1.01 mmol, 69% over two steps). HRMS (ESI) [M + H]^+^ found 374.1963, C_17_H_24_N_7_O_3_ requires 374.1938, [M + Na]^+^ found 396.1758, C_17_H_23_N_7_O_3_Na requires 396.1755. ^1^H NMR (500 MHz, DMSO) δ 12.35 (1H, br s), 7.19 (2H, d, *J* = 8.0 Hz), 7.03 (2H, d, *J* = 8.0 Hz), 5.26 (1H, tt, *J* = 7.7, 5.3 Hz), 4.62 (2H, s), 4.46 (2H, s), 3.71 (2H, d, *J* = 5.6 Hz), 2.24–2.15 (2H, m), 2.08–1.98 (2H, m), 1.84–1.75 (2H, m), 1.75–1.67 (2H, m).

#### *1-[(4-Aminophenyl)methyl]-3-{2-[2-(2-bromophenyl)pyrrolidin-1-yl]-2-oxoethyl}-1-[(2-methyl-2H-1,2,3,4-tetrazol-5-yl)cyclopentyl]urea hydrochloride* (**8**)

Crude acid **25ai** (377 mg, 1.01 mmol) was reacted with 2-(2-bromophenyl) pyrrolidine (**27**) (457 mg, 2.02 mmol) according to General Procedure E. The crude product was purified with preparative HPLC to afford compound **8** as a yellow fluffy solid (200 mg, 0.35 mmol, 35%). LCMS 3.76 min (92% @ TAC), 581.0, 583.0 [M + H]^+^, halide isotope splitting pattern. HRMS (ESI) [M + H]^+^ found 581.1984, C_27_H_34_O_2_N_8_^79^Br (100.0%) requires 581.1983; [M + H]^+^ found 583.1964, C_27_H_34_O_2_N_8_^81^Br (97.5%) requires 583.1964. mp 222–225 °C. ^1^H NMR (500 MHz, MeOD) δ 7.64–7.56 (1H, ddd, *J* = 36.78, 7.93, 4.84), 7.47 (1H, d, *J* = 8.2 Hz), 7.44–7.38 (1.4H, m), 7.31 (1H, d, *J* = 8.2 Hz), 7.29–7.24 (2H, m), 7.15–7.09 (1.6H, m), 5.40 (1H, m), 5.23 (1H, m), 4.71 (2H, s), 4.52 (2H, s), 4.60 (1H, br s), 4.17- 4.04 (1.2H, td, *J* = 16.9, 10.2 Hz), 4.02–3.93 (0.8H, m), 3.86–3.79 (0.8H, m), 3.78–3.70 (1.2H, m), 2.56–2.34 (1.2H, m), 2.30–2.09 (6H, m), 1.94–1.73 (4.8H, m).

### Preparation of Compound 9

#### *Ethyl 2-({[(2-Cyclohexyl-2H-1,2,3,4-tetrazol-5-yl)methyl][(4-nitrophenyl)methyl] carbamoyl}amino)acetate* (**23aj**)

Tetrazole **23ah** (3.97 g, 10.9 mmol) and cyclohexanol (1.14 mL, 10.9 mmol, 1.00 equiv) were dissolved in dry DCM (55.0 mL, 0.20 M) at 5 °C. Addition of PPh_3_ (2.87 g, 10.9 mmol, 1.00 equiv) in one portion and neat DEAD (1.74 mL, 10.9 mmol, 1.00 equiv) dropwise over 10 min followed. The reaction mixture was stirred briefly and was then allowed to warm to room temperature. After 20 h, the solvent was removed *in vacuo* to give a ∼ 50:50 regioisomeric mixture of 1- and 2-alkylated tetrazoles. The crude material was purified using flash chromatography (hexane/EtOAc, 2:3) to afford predominantly the faster-eluting 2-substitued tetrazole **23aj** as an orange oil (379 mg, 0.85 mmol, 8%). HRMS (ESI) [M + H]^+^ found 446.2158, C_20_H_28_N_7_O_5_ requires 446.2146. ^1^H NMR (500 MHz, CDCl_3_) δ 8.19 (2H, d, *J* = 8.7 Hz), 7.48 (2H, d, *J* = 8.7 Hz), 5.90 (1H, t, *J* = 5.3 Hz), 4.76 (2H, s), 4.71–4.66 (1H, m), 4.65 (2H, s), 4.23 (2H, q, *J* = 7.1 Hz), 4.07 (2H, d, *J* = 5.3 Hz), 2.23–2.15 (2H, m), 1.97–1.86 (4H, m), 1.81–1.73 (1H, m), 1.50–1.43 (2H, m), 1.39–1.33 (1H, m), 1.30 (3H, t, *J* = 7.1 Hz).

#### *2-({[(2-Methyl-2H-1,2,3,4-tetrazol-5-yl)cyclohexyl][(4-aminophenyl)methyl] carbamoyl} amino)acetic acid* (**25aj**)

Urea ester **23aj** (379 mg, 0.850 mmol) was reacted according to the General Procedure C to produce 2-({[(2-Methyl-2*H*-1,2,3,4-tetrazol-5-yl)cyclohexyl][(4-nitrophenyl)methyl] carbamoyl}amino)acetic acid (**24aj**) as a thick, pale brown oil (301 mg, 0.722 mmol). The crude acid **24aj** (301 mg, 0.722 mmol) was reacted according to General Procedure D to afford aniline **25aj** which was concentrated *in vacuo* and reacted without further purification (282 mg, 0.722 mmol 85% over two steps). HRMS (ESI) [M + H]^+^ found 418.1836, C_18_H_24_N_7_O_5_ requires 418.1833. ^1^H NMR (500 MHz, CDCl_3_) δ 8.89(1H, br s, O*H*), 8.19 (2H, d, *J* = 8.7 Hz), 7.48 (2H, d, *J* = 8.7 Hz), 6.10 (1H, br s), 4.76 (2H, s), 4.69 (1H, dd, *J* = 7.0, 4.4 Hz), 4.65 (2H, s), 4.12 (2H, s), 2.24–2.16 (2H, m), 1.97–1.85 (4H, m), 1.81–1.73 (1H, m,), 1.54–1.43 (2H, m), 1.39–1.30 (1H, m).

#### *1-[(4-Aminophenyl)methyl]-3-{2-[2-(2-bromophenyl)pyrrolidin-1-yl]-2-oxoethyl}-1-[(2-methyl-2H-1,2,3,4-tetrazol-5-yl)cyclohexyl]urea hydrochloride* (**9**)

Crude acid **25aj** (282 mg, 0.722 mmol) was reacted with 2-(2-bromophenyl) pyrrolidine (**27**) (327 mg, 1.44 mmol) according to General Procedure E to afford the final product (**9**) as a yellow, fluffy solid (229 mg, 0.380 mmol, 53%). LCMS 3.89 min (92% @ TAC), 595.0, 597.0 [M + H]^+^, halide isotope splitting pattern. HRMS (ESI) [M + H]^+^ found 595.2561, C_28_H_35_N_8_O_2_^79^Br (100.0%) requires 595.2565; found 597.1974, C_27_H_34_O_2_N_8_^81^Br (97.5%) requires 597.1956. mp 225–228 °C. ^1^H NMR (601 MHz, MeOD) δ 7.62 (1H, dd, *J* = 44.1, 7.9 Hz), 7.53–7.34 (3H, m), 7.33–7.22 (3H, m), 7.17 (3H, m), 5.44–5.39 (1H, m), 4.77–4.68 (3H, m), 4.68–4.61 (2H, m), 4.13 (1.2H, q, *J* = 16.9 Hz), 4.01–3.94 (0.8H, m), 3.95–3.82 (0.8H, m), 3.79–3.68 (1.2H, m), 2.57–2.32 (1.2H, m), 2.20–2.12 (2H, m), 2.07–2.02 (0.8H, m), 2.00–1.84 (6H, m), 1.79–1.72 (0.8H, m), 1.57–1.47 (2H, m), 1.37–1.29 (1.2H, m). Rotameric ratio 3:2.

### Preparation of Compound 10

#### *2-Chloro-4-[[(2-methyltetrazol-5-yl)methylamino]methyl]aniline* (**22da**)

4-(Aminomethyl)-2-chloro-aniline (**20d**) (103 mg, 0.56 mmol) and 5-(chloromethyl)-2-methyl-tetrazole (**21a**) (74.1 mg, 0.560 mmol) were reacted according to General Procedure A. Purification by flash column chromatography (silica column, 20% to 100% EtOAc in heptane gradient) followed by concentration *in vacuo* afforded amine **22da** as a yellow gum (70 mg, 0.26 mmol, 52%). LCMS (Method A): 1.04 min, 140.2, 142.2 [M – C_3_H_6_N_5_]^+^, halide isotope splitting pattern. ^1^H NMR (400 MHz, DMSO) δ 7.14 (d, *J* = 1.9 Hz, 1H), 6.96 (dd, *J* = 8.2, 2.0 Hz, 1H), 6.73 (d, *J* = 8.2 Hz, 1H), 5.18 (s, 2H), 4.32 (s, 3H), 3.83 (s, 2H), 3.54 (s, 2H), 2.55 (s, 1H).

#### *Ethyl 2-[[(4-Amino-3-chloro-phenyl)methyl-[(2-methyltetrazol-5-yl)methyl] carbamoyl] amino]acetate* (**23da**)

Amine **22da** (96 mg, 0.34 mmol) was reacted according to General Procedure B. The reaction mixture was concentrated *in vacuo* to afford crude product. Purification by flash column chromatography (silica column, 50% to 100% EtOAc in heptane gradient) followed by evaporation of solvent afforded urea **23da** as a pale yellow solid (119 mg, 0.31 mmol, 82%). LCMS (Method A): 1.91 min, 382.0, 384.2 [M + H]^+^, halide isotope splitting pattern. ^1^H NMR (400 MHz, DMSO) δ 7.12 (t, *J* = 5.8 Hz, 1H), 7.06 (d, *J* = 2.0 Hz, 1H), 6.90 (dd, *J* = 8.3, 2.0 Hz, 1H), 6.71 (d, *J* = 8.2 Hz, 1H), 5.25 (s, 2H), 4.54 (s, 2H), 4.29 (s, 5H), 4.07 (q, *J* = 7.1 Hz, 2H), 3.74 (d, *J* = 5.7 Hz, 2H), 1.17 (t, *J* = 7.1 Hz, 3H).

#### *2-[[(4-Amino-3-chloro-phenyl)methyl-[(2-methyltetrazol-5-yl)methyl] carbamoyl]amino] acetic acid* (**25da**)

Urea ester **23da** (161 mg, 0.380 mmol) was reacted according to General Procedure C. The reaction was concentrated *in vacuo* to afford acid **25da** as an off-white solid (161 mg, 0.380 mmol, 99%). LCMS (Method A) 1.61 min, 140.2, 142.2 [M – C_6_H_9_O_3_N_6_]^+^, fragmentation to benzylic cation. ^1^H NMR (400 MHz, DMSO) δ 7.06 (d, *J* = 2.0 Hz, 1H), 6.91 (dd, *J* = 8.2, 2.0 Hz, 1H), 6.71 (d, *J* = 8.2 Hz, 1H), 6.42 (s, 1H), 5.24 (s, 2H), 4.54 (s, 2H), 4.29 (s, 3H), 4.27 (s, 2H). Exchangeable NH_2_ and COOH protons not observed.

#### *1-[(4-Amino-3-chloro-phenyl)methyl]-1-[(2-methyltetrazol-5-yl)methyl]-3-[2-oxo-2-[(2R)-2-(2-bromophenyl)pyrrolidin-1-yl]ethyl]urea* (**10**)

Crude acid **25da** (83 mg, 0.19 mmol) and (2*R*)-2-(2-bromophenyl)pyrrolidine (*R*)-(**27**) (88 mg, 0.39 mmol) were reacted according to General Procedure E to afford the crude product. Purification by flash column chromatography (silica column, 0% to 5% 2 M methanolic ammonia gradient) followed by prep. HLPC (basic) afforded compound **10** (56 mg, 0.100 mmol, 51%). LCMS (method A) 1.74 min (100% @ 254 nm), 561.0, 563.0 [M + H]^+^, halide isotope splitting pattern. ^1^H NMR (400 MHz, CDCl_3_) δ 7.57–7.51 (m, 1H), 7.37–6.86 (m, 5H), 6.71–6.66 (m, 1H), 5.87–5.74 (m, 1H), 5.43–5.21 (m, 1H), 4.70–4.58 (m, 2H), 4.48–4.34 (m, 2H), 4.30–4.28 (m, 3H), 4.21–4.02 (m, 3H), 3.86–3.33 (m, 3H), 2.45–2.39 (m, 1H), 2.02–1.80 (m, 3H).

### Preparation of Compound 11

#### *1-[(4-Amino-3-chloro-phenyl)methyl]-1-[(2-methyltetrazol-5-yl)methyl]-3-[2-oxo-2-[(2R)-2-(2-methylsulfanylphenyl)pyrrolidin-1-yl]ethyl]urea* (**11**)

Crude acid **25da** (42 mg, 0.10 mmol) and (2*R*)-2-(2-methylsulfanylphenyl) pyrrolidine (*R*)-(**26**) (38 mg, 0.20 mmol) were reacted according to General Procedure E. The organics were combined, filtered through a hydrophobic frit and concentrated *in vacuo* to afford crude product. Purification by flash column chromatography (silica column, 0% to 10% 2 M methanolic ammonia in DCM gradient) followed by evaporation of solvent from the appropriate fractions afforded partially purified product. Further purification by prep. HPLC (basic) followed by evaporation of solvent from the appropriate fractions and drying under vacuum afforded compound **11** as a white solid (24 mg, 0.045 mmol, 45%). LCMS (Method A) 2.44 min (100% @ 254 nm), 529.2, 531.2 [M + H]^+^, halide isotope splitting pattern. ^1^H NMR (400 MHz, DMSO) δ 7.37–7.13 (m, 2.5H), 7.10–6.96 (m, 2.5H), 6.94 (s, 1H), 6.71 (dd, *J* = 8.2, 5.3 Hz, 1H), 6.62 (t, *J* = 5.2 Hz, 0.5H), 6.56 (t, *J* = 5.3 Hz, 0.5H), 5.28–5.20 (m, 3H), 4.57 (s, 1H), 4.54–4.44 (m, 1H), 4.33–4.23 (m, 4H), 3.96 (d, *J* = 5.0 Hz, 1H), 3.89–3.74 (m, 0.5H), 3.74–3.65 (m, 0.5H), 3.63–3.49 (m, 0.5H), 3.29 (d, *J* = 0.9 Hz, 3H), 3.00 (dd, *J* = 16.7, 5.1 Hz, 0.5H), 2.53 (s, 3H), 2.23–2.05 (m, 0.5H), 1.97–1.59 (m, 2.5H).

### Preparation of Compound 12

#### *tert-Butyl N-tert-Butoxycarbonyl-N-[5-[[(5-methylthiazol-2-yl)methylamino] methyl] pyrimidin-2-yl]carbamate* (**22eb**)

*tert*-Butyl *N*-[5-(aminomethyl)pyrimidin-2-yl]-*N*-*tert*-butoxy carbonyl-carbamate (**20e**) (190 mg, 0.290 mmol) and 2-(chloromethyl)-5-methyl-thiazole (**21b**) (48 mg, 0.32 mmol) were reacted according to General Procedure A to give crude amine **22eb** (240 mg, 0.28 mmol, 94%). LCMS (Method B) 1.48 min, 458.2 [M + Na]^+^. ^1^H NMR (400 MHz, Chloroform*-d*) δ 8.77 (s, 2H), 7.37 (q, *J* = 1.3 Hz, 1H), 4.08 (s, 2H), 3.90 (s, 2H), 2.46 (d, *J* = 1.2 Hz, 3H), 1.56–1.42 (m, 18H).

#### *Ethyl 2-[[[2-[Bis(tert-butoxycarbonyl)amino]pyrimidin-5-yl]methyl-[(5-methylthiazol −2-yl)methyl]carbamoyl]amino]acetate* (**23eb**)

Crude amine **22eb** (261 mg, 0.30 mol) was reacted according to General Procedure B. Purification of the crude product by flash column chromatography (silica column, 0% to 8% 2 M methanolic ammonia in DCM gradient) followed by concentration *in vacuo* afforded the urea **23eb** (212 mg, 0.28 mmol, 94%). LCMS (Method B) 1.86 min, 587.2 [M + Na]^+^. ^1^H NMR (400 MHz, CDCl_3_) δ 8.69 (s, 2H), 7.38 (q, *J* = 1.2 Hz, 1H), 6.42 (t, *J* = 5.3 Hz, 1H), 4.64 (s, 2H), 4.54 (s, 2H), 4.20 (q, *J* = 7.0 Hz, 2H), 4.02 (d, *J* = 5.3 Hz, 2H), 2.44 (d, *J* = 1.3 Hz, 3H), 1.48–1.40 (m, 18H), 1.28 (t, *J* = 7.2 Hz, 3H).

#### *2-[[[2-[Bis(tert-butoxycarbonyl)amino]pyrimidin-5-yl]methyl-[(5-methylthiazol-2-yl)methyl]carbamoyl]amino]acetic acid* (**25eb**)

Urea ester **23eb** (240 mg, 0.320 mmol) was reacted according to General Procedure C. The crude acid **25eb** was used without further purification in subsequent reactions (199 mg, 0.30 mmol, 93%). LCMS (Method B) 1.70 min, 559 [M + Na]^+^. ^1^H NMR (400 MHz, CDCl_3_) δ 8.70 (s, 2H), 7.40 (d, *J* = 0.9 Hz, 1H), 6.95–6.91 (m, 1H), 4.64 (s, 2H), 4.52 (s, 2H), 4.05 (d, *J* = 5.5 Hz, 2H), 2.45 (d, *J* = 1.1 Hz, 3H), 1.51–1.42 (m, 18H). Exchangeable COOH proton not observed.

#### *1-[(2-Aminopyrimidin-5-yl)methyl]-1-[(5-methylthiazol-2-yl)methyl]-3-[2-oxo-2-[(2R)-2-(2-methylsulfanylphenyl)pyrrolidin-1-yl]ethyl]urea* (**12**)

Crude acid **25eb** (161 mg, 0.300 mmol) and (2*R*)-2-(2-methylsulfanylphenyl)pyrrolidine (*R*)-(**26**) (116 mg, 0.60 mmol) were reacted according to General Procedure E to give *tert*-butyl *N*-*tert*-butoxycarbonyl-*N*-[5-[[(5-methylthiazol-2-yl)methyl-[[2-oxo-2-[(2*R*)-2-(2-methylsulfanylphenyl)pyrrolidin-1-yl]ethyl]carbamoyl]amino]methyl]pyrimidin-2-yl]carbamate (**28eb**) (214 mg, 0.300 mmol, 100%) which was used in the subsequent step without purification. Crude carbamate **28eb** (214 mg, 0.300 mmol) was dissolved in DCM (5 mL) at room temperature and trifluoroacetic acid (1 mL, 13.0 mmol) was added. The reaction was stirred for 1 h at room temperature and concentrated *in vacuo*. Purification by SCX chromatography (washing with DCM and MeOH and eluting with 2 M methanolic ammonia) followed by concentration *in vacuo* afforded compound **12** (23 mg, 0.05 mmol, 15%). LCMS (Method B) 2.20 min (100% @ 254 nm), 512.0 [M + H]^+^. ^1^H NMR (400 MHz, CDCl_3_) δ 8.22 (s, 2H), 7.38–7.05 (m, 4H), 6.98–6.89 (m, 1H), 6.06–5.99 (m, 1H), 5.56–5.23 (m, 1H), 5.06 (s, 2H), 4.64–4.52 (d, *J* = 18.0 Hz, 2H), 4.45–4.29 (m, 2H), 4.20–4.02 (m, 1H), 3.94–3.31 (m, 3H), 2.71–2.14 (m, 7H), 2.12–1.76 (m, 3H).

### Preparation of Compound 13

#### *Ethyl 2-[[[2-[Bis(tert-butoxycarbonyl)amino]pyrimidin-5-yl]methyl-[(5-methyl-1,3,4-thiadiazol-2-yl)methyl]carbamoyl]amino]acetate* (**23ec**)

*tert*-Butyl *N*-[5-(aminomethyl)pyrimidin-2-yl]-*N*-*tert*-butoxycarbonyl-carbamate (**20e**) (64 mg, 0.20 mmol) and 2-(chloromethyl)-5-methyl-1,3,4-thiadiazole (**21c**) (35 mg, 0.24 mmol) were reacted according to General Procedure A. Purification of the crude product by flash column chromatography (silica column, 0% to 6% 2 M methanolic ammonia in DCM gradient) followed by evaporation of solvent from the appropriate fractions afforded crude *tert*-Butyl *N*-*tert*-butoxycarbonyl-*N*-[5-[[(5-methyl-1,3,4-thiadiazol-2-yl)methylamino]-methyl]pyrimidin-2-yl]carbamate (**22ec)** (59 mg, 0.14 mmol, 68%) which was used without further purification. Crude amine **22ec** (59 mg, 0.14 mmol) was reacted according to General Procedure B. Purification by flash column chromatography (silica column, 0% to 100% EtOAc in heptane gradient) followed by concentration *in vacuo* afforded urea **23ec** as a pale-yellow gum (44 mg, 0.080 mmol, 58%). LCMS (Method A): 2.43 min, 588.2 [M + Na]^+^. ^1^H NMR (400 MHz, DMSO) δ 8.71 (s, 2H), 7.46 (t, *J* = 5.8 Hz, 1H), 4.84 (s, 2H), 4.61 (s, 2H), 4.09 (q, *J* = 7.1 Hz, 2H), 3.79 (d, *J* = 5.8 Hz, 2H), 2.66 (s, 3H), 1.40 (s, 18H), 1.18 (t, *J* = 7.2 Hz, 3H).

#### *1-[(2-Aminopyrimidin-5-yl)methyl]-1-[(5-methyl-1,3,4-thiadiazol-2-yl)methyl]-3-[2-oxo-2-[(2R)-2-(2-methylsulfanylphenyl)pyrrolidin-1-yl]ethyl]urea* (**13**)

Urea ester **23ec** (115 mg, 0.183 mmol) was reacted according to General Procedure C. HCl (1 M aq.) was added to the reaction mixture to adjust to pH 5 and the mixture was concentrated *in vacuo* to afford crude *2-[[[2-[bis(tert-butoxycarbonyl)amino]pyrimidin-5-yl]methyl-[(5-methyl-1,3,4-thiadiazol-2-yl)methyl]carbamoyl]amino]acetic acid* (**25ec**) as a yellow gum (150 mg, 0.167 mmol, 91% yield) which was used directly in the next reaction. Crude acid **25ec** (110 mg, 0.151 mmol) was reacted with (*R*)-2-(2-Methylsulfanylphenyl)pyrrolidine (*R*)-(**26**) (44 mg, 0.226 mmol) according to General Procedure E to afford crude *tert*-butyl *N*-[5-[[(5-methyl-1,3,4-thiadiazol-2-yl)methyl-[[2-oxo-2-[(2*R*)-2-(2-methyl-sulfanylphenyl)pyrrolidin-1-yl]ethyl]carbamoyl] amino]methyl]pyrimidin-2-yl]carbamate (**28ec)** as a yellow gum (120 mg, 0.104 mmol, 69% yield). LCMS (method A): 2.73 min, 735.2 [M + Na]^+^. Crude carbamate **28ec** (120 mg, 0.104 mmol) was dissolved in DCM (2 mL) and trifluoroacetic acid (0.50 mL, 6.5 mmol) added. The reaction was stirred at room temperature for 1 h. The reaction mixture was concentrated *in vacuo* to afford crude product as a yellow oil. Purification by SCX chromatography (washing alternately with MeOH and DCM, eluting product with 2 M methanolic ammonia) followed by evaporation of solvent afforded crude product. Purification by prep. HPLC (basic) and concentration *in vacuo* afforded compound **13** as a white solid (25 mg, 0.05 mmol, 46%). LCMS (Method A): 1.94 min (100% @ 254 nm), 513.2 [M + H]^+^. ^1^H NMR (400 MHz, DMSO) δ 8.16 (d, *J* = 3.5 Hz, 2H), 7.38–7.14 (m, 2.5H), 7.11–6.93 (m, 2H), 6.84 (t, *J* = 5.5 Hz, 0.5H), 6.54 (d, *J* = 8.0 Hz, 2H), 5.30–5.19 (m, 1H), 4.75–4.57 (m, 2H), 4.35–4.18 (m, 2H), 3.98 (d, *J* = 5.3 Hz, 1H), 3.92–3.77 (m, 0.5H), 3.70 (q, *J* = 6.0, 4.2 Hz, 0.5H), 3.64–3.49 (m, 0.5H), 3.02 (dd, *J* = 16.6, 5.2 Hz, 0.5H), 2.63 (s, 3H), 2.54 (s, 1.5H), 2.39–2.30 (m, 0.5H), 2.17 (tt, *J* = 12.0, 7.7 Hz, 0.5H), 2.00–1.59 (m, 4H). *Note - signal due to SMe only integrates to 1.5H, signal is assumed to be split and second peak of 1.5H hidden beneath adjacent DMSO signal.*

### Preparation of Compound 14

#### *3-Chloro-5-[[(5-methyl-1,3,4-thiadiazol-2-yl)methylamino]methyl]pyridin-2-amine* (**22 fc**)

5-(Aminomethyl)-3-chloro-pyridin-2-amine (**20f**) (200 mg, 1.21 mmol) and 2-(chloromethyl)-5-methyl-1,3,4-thiadiazole (**21c**) (179 mg, 1.21 mmol) were reacted according to General Procedure A. Purification by flash column chromatography (silica column, 0% to 5% 2 M methanolic ammonia in DCM gradient) followed concentration *in vacuo* afforded amine **22 fc** (127 mg, 0.350 mmol, 29%). LCMS (Method B) 0.75 min, 270.2, 272.2 [M + H]^+^, halide isotope splitting pattern. ^1^H NMR (400 MHz, CDCl_3_) δ 7.92 (d, *J* = 2.0 Hz, 1H), 7.56 (d, *J* = 2.0 Hz, 1H), 4.91 (s, 2H), 4.18 (s, 2H), 3.74 (s, 2H), 2.77 (s, 3H).

#### *Ethyl 2-[[(6-Amino-5-chloro-3-pyridyl)methyl-[(5-methyl-1,3,4-thiadiazol-2-yl) methyl] carbamoyl]amino]acetate* (**23 fc**)

Amine **22 fc** (126 mg, 0.470 mmol) was reacted to General Procedure B. Purification by flash column chromatography (silica column, 0% to 4% 2 M methanolic ammonia in DCM gradient) followed by concentration *in vacuo* afforded urea **23 fc** (93 mg, 0.20 mmol, 42%). LCMS (Method B) 1.27 min, 399.2, 401.2 [M + H]^+^, halide isotope splitting pattern. ^1^H NMR (400 MHz, CDCl_3_) δ 7.78 (dq, *J* = 5.1, 2.8 Hz, 1H), 7.39 (dt, *J* = 5.7, 2.5 Hz, 1H), 5.95–5.86 (m, 1H), 5.14 (dd, *J* = 11.6, 5.8 Hz, 2H), 4.75–4.68 (m, 2H), 4.39–4.33 (m, 2H), 4.19–4.08 (m, 2H), 3.95 (dt, *J* = 7.1, 3.7 Hz, 2H), 2.69 (dd, *J* = 5.1, 2.8 Hz, 3H), 1.21 (dq, *J* = 9.9, 3.4 Hz, 3H).

#### *1-[(6-Amino-5-chloro-3-pyridyl)methyl]-1-[(5-methyl-1,3,4-thiadiazol-2-yl)methyl]-3-[2-oxo-2-[(2R)-2-(2-methylsulfanylphenyl)pyrrolidin-1-yl]ethyl]urea* (**14**)

Urea ester **23 fc** (84 mg, 0.21 mmol) was reacted according to General Procedure C. The reaction mixture concentrated *in vacuo* to afford the 2-[[(6-amino-5-chloro-3-pyridyl)methyl-[(5-methyl-1,3,4-thiadiazol-2-yl)methyl]-carbamoyl]amino]acetic acid (**25 fc**) as an off-white solid (321 mg) which was used in the next reaction without further purification. Crude acid **25 fc** (39 mg, 0.11 mmol) and (2*R*)-2-(3-methylsulfanylphenyl)pyrrolidine (*R*)-(**26**) (41 mg, 0.21 mmol) were reacted according to General Procedure E. Purification by flash column chromatography (silica column, 0% to 5% 2 M methanolic ammonia in DCM gradient) was followed by prep. HLPC (basic, followed by acidic) and concentration *in vacuo*. The material afforded was stirred in MeCN over K_2_CO_3_, filtered and concentrated *in vacuo* to afford compound **14** (10 mg, 0.018 mmol, 17%). LCMS (Method B) 1.52 min (100% @ 254 nm), 546.2, 548.2 [M + H]^+^, halide isotope splitting pattern. ^1^H NMR (400 MHz, CDCl_3_) δ 7.89–7.84 (m, 1H), 7.43–6.89 (m, 5H), 5.89–5.70 (m, 1.3H), 5.48–5.21 (m, 1H), 4.92 (m, 2H), 4.83–4.66 (m, 2H), 4.38–3.54 (d, *J* = 8.9 Hz, 5H), 3.35–3.30 (d, *J* = 3.4 Hz, 0.7H), 2.72–2.70 (m, 3H), 2.53–2.48 (m, 3H), 2.39–1.81 (m, 4H).

### Preparation of Compound 15

#### *2-[(4-Amino-3,5-difluoro-phenyl)methylamino]acetonitrile* (**22cf**)

4-(Aminomethyl)-2,6-difluoro-aniline (**20c**) (70 mg, 0.44 mmol) was reacted with chloroacetonitrile (**21f**) according to General Procedure A. The crude product was purified by flash column chromatography on the Biotage Isolera (12 g silica, 0 to 2% MeOH/DCM) to provide amine **22cf** as colorless gum (45 mg, 0.23 mmol, 51%). HRMS (ESI) [M + H]^+^ found 198.1920, C_9_H_9_F_2_N_3_ requires 198.1920. ^1^H NMR (400 MHz, CDCl_3_) δ 6.87–6.78 (m, 2H), 3.81 (s, 2H), 3.71 (s, 2H), 3.55 (s, 2H), 1.60 (s, 1H).

#### *Ethyl 2-[[(4-Amino-3,5-difluoro-phenyl)methyl-(cyanomethyl)carbamoyl] amino]acetate* (**23cf**)

Amine **22cf** (45 mg, 0.23 mmol) was reacted according to General Procedure B. The crude product was purified by flash column chromatography on the Biotage Isolera (12 g silica, 0 to 2.5% MeOH/DCM) to afford urea **23cf** as colorless gum which solidified on standing (66 mg, 0.20 mmol, 89%). LCMS (Method B): 1.462 min, 327.2 [M + H]^+^. ^1^H NMR (400 MHz, CDCl_3_) δ 6.80 (dd, *J* = 6.8, 2.0 Hz, 2H), 5.12 (t, *J* = 5.1 Hz, 1H), 4.43 (s, 2H), 4.28 (s, 2H), 4.21 (q, *J* = 7.1 Hz, 2H), 4.01 (d, *J* = 5.1 Hz, 2H), 3.79 (s, 2H), 1.28 (t, *J* = 7.2 Hz, 3H).

#### *1-[(4-Amino-3,5-difluoro-phenyl)methyl]-3-[2-oxo-2-[(2R)-2-(2-methylsulfanylphenyl) pyrrolidine-1-yl]ethyl]imidazolidine-2,4-dione* (**15**)

Urea ester **23cf** (66.0 mg, 0.20 mmol) was dissolved in MeOH (3 mL) and lithium hydroxide monohydrate (17.0 mg, 0.40 mmol) dissolved in water (0.5 mL) was added dropwise. The solution was stirred at room temperature for ∼1.5 h before acidifying to pH 5 with HCl (1 N aq.). The aqueous layer was extracted with EtOAc (′3) giving an insoluble precipitate before being acidified to pH 2. The EtOAc and aqueous layers were mixed for a second time dissolving all solids and the phases separated. The aqueous layer was concentrated to dryness to give ∼95 mg of a colorless gum. The crude material was purified on acidic HPLC to give a hydantoin intermediate as a beige solid (22 mg, 0.074 mmol, 37%). The hydantoin intermediate (22 mg, 0.074 mmol) and (2*R*)-2-(2-methylsulfanyl-phenyl)pyrrolidine (*R*)-(**26**) (14 mg, 0.070 mmol) were reacted according to General Procedure E. The crude product was purified by reverse phase column chromatography (Xbridge C18, 10–95% MeCN/H_2_O with NH_4_OH modifier) to give compound **15** as a beige solid (7.0 mg, 0.015 mmol, 20%). LCMS (Method A): 2.43 min (100% @ 254 nm), 475.2 [M + H]^+^. ^1^H NMR (400 MHz, DMSO) δ 7.39–7.18 (m, 2.4H), 7.15–7.06 (m, 1H), 7.05–6.98 (m, 0.6H), 6.86–6.74 (m, 2H), 5.36–5.20 (m, 1H), 5.19–5.14 (m, 2H), 4.45–4.34 (m, 1.2H), 4.34–4.24 (m, 2H), 4.17–4.08 (m, 0.4H), 4.01–3.84 (m, 2.4H), 3.77–3.63 (m, 1H), 3.60–3.48 (m, 0.4H), 3.42–3.33 (m, 0.4H), 2.56–2.53 (m, 1.2H), 2.49–2.48 (m, 1.6H), 2.46–2.29 (m, 0.4H), 2.29–2.15 (m, 0.6H), 2.03–1.58 (m, 3H).

### Preparation of Compound 16

#### *2-Chloro-4-[[(5-methyl-1,3,4-oxadiazol-2-yl)methylamino]methyl]aniline* (**22dd**)

4-(Aminomethyl)-2-chloro-aniline (**20d**) (341 mg, 1.85 mmol) and 2-(chloromethyl)-5-methyl-1,3,4-oxadiazole (**21d**) (245 mg, 1.85 mmol) were reacted according to General Procedure A. Purification by flash column chromatography (silica column, 20% to 100% EtOAc in heptane gradient) followed by concentration *in vacuo* afforded amine **22dd** as a yellow gum (212 mg, 0.710 mmol, 39%). LCMS (Method A): 1.82 min, 140.2, 142.2 [M-C_3_H_6_ON_3_]^+^, halide isotope splitting pattern. Mass consistent with fragmentation to afford benzylic cation, parent mass ion not observed. ^1^H NMR (400 MHz, DMSO) δ 7.13 (d, *J* = 2.0 Hz, 1H), 6.94 (dd, *J* = 8.2, 2.0 Hz, 1H), 6.73 (dd, *J* = 8.2, 4.0 Hz, 1H), 5.19 (s, 2H), 3.79 (s, 2H), 3.53 (s, 2H), 2.79 (s, 1H), 2.46 (s, 3H).

#### *Ethyl 2-[[(4-Amino-3-chloro-phenyl)methyl-[(5-methyl-1,3,4-oxadiazol-2-yl)methyl] carbamoyl]amino]acetate* (**23dd**)

Amine **22dd** (212 mg, 0.710 mmol) was reacted according to General Procedure B. Purification by flash column chromatography (silica column, 0% to 3% MeOH in DCM gradient) followed by concentration *in vacuo* afforded urea **23dd** (235 mg, 0.520 mmol, 73%). LCMS (Method A) 1.80 min, 382.0, 384.2 [M + H]^+^, halide isotope splitting pattern. ^1^H NMR (400 MHz, DMSO) δ 7.25 (t, *J* = 5.7 Hz, 1H), 7.08 (d, *J* = 2.0 Hz, 1H), 6.92 (dd, *J* = 8.2, 2.1 Hz, 1H), 6.72 (dd, *J* = 8.2, 3.9 Hz, 1H), 5.27 (s, 2H), 4.54 (s, 2H), 4.33 (s, 2H), 4.10 (q, *J* = 7.1 Hz, 2H), 3.78 (d, *J* = 5.7 Hz, 2H), 2.41 (s, 3H), 1.20 (t, *J* = 7.1 Hz, 3H).

#### *N-[[1-[(4-amino-3-chloro-phenyl)methyl]-2-oxo-3-[2-oxo-2-[(2R)-2-(2-methylsulfanyl phenyl)pyrrolidin-1-yl]ethyl]imidazolidin-4-ylidene]amino]acetamide* (**16**)

Urea ester **23dd** (235 mg, 0.520 mmol) was reacted according to General Procedure C to give crude 2-[5-(Acetylhydrazono)-3-[(4-amino-3-chloro-phenyl)methyl]-2-oxo-imidazolidin-1-yl]acetic acid (**30**) as an off-white solid (307 mg, 0.520 mmol, 99%). LCMS (method A) 1.57 min, 354.0, 356.0 [M + H]^+^, halide isotope splitting pattern. Crude acid **30** (60 mg, 0.17 mmol) and (2*R*)-2-(3-methylsulfanylphenyl)pyrrolidine (*R*)-(**26**) (49 mg, 0.25 mmol) were reacted according to General Procedure E. Purification by flash column chromatography (silica column, 0% to 5% 2 M methanolic ammonia in DCM gradient) followed by concentration *in vacuo* afforded partially purified product. Further purification by prep. HPLC (basic) followed by concentration *in vacuo* afforded hydantoin **16** as a white solid (33 mg, 0.062 mmol, 36%). LCMS (Method A) 2.31 min (100% @ 254 nm), 529.2, 531.2 [M + H]^+^, halide isotope splitting pattern. ^1^H NMR (400 MHz, DMSO) δ 9.87–9.76 (m, 1H), 7.38–7.14 (m, 2.5H), 7.14–6.95 (m, 2.5H), 6.91–6.84 (m, 1H), 6.75 (dt, *J* = 8.2, 1.7 Hz, 1H), 5.45–5.28 (m, 2H), 5.22 (d, *J* = 7.0 Hz, 0.5H), 4.44–4.17 (m, 3H), 4.11–3.86 (m, 3H), 3.69 (t, *J* = 8.6 Hz, 1H), 3.59–3.40 (m, 0.5H), 3.29 (s, 3H), 2.53 (d, *J* = 3.8 Hz, 2H), 2.48 (d, *J* = 1.6 Hz, 1H), 2.18 (t, *J* = 9.6 Hz, 0.5H), 2.00–1.88 (m, 0.5H), 1.87–1.69 (m, 3.5H), 1.70–1.58 (m, 0.5H).

### Preparation of Compound 17

#### *N-[[1-[(4-amino-3-chloro-phenyl)methyl]-3-[2-[(2R)-2-(2-bromophenyl)pyrrolidin-1-yl]-2-oxo-ethyl]-2-oxo-imidazolidin-4-ylidene]amino]acetamide* (**17**)

Crude acid **30** (80 mg, 0.22 mmol) and (2*R*)-2-(2-bromophenyl)pyrrolidine (*R*)-(**27**) (61 mg, 0.27 mmol) were reacted according to General Procedure E. Purification by flash column chromatography (silica column, 0% to 5% 2 M methanolic ammonia in DCM gradient) followed by prep. HLPC (basic) afforded hydantoin **17** (33 mg, 0.059 mmol, 27%). LCMS (Method B) 1.68 min (100% @ 254 nm), 561.0, 563.0 [M + H]^+^, halide isotope splitting pattern. ^1^H NMR (400 MHz, CDCl_3_) δ 9.74 (s, 0.4H), 9.51 (s, 0.3H), 8.69 (s, 0.3H), 7.66–6.63 (m, 7H), 5.56–5.11 (m, 1H), 4.62–3.35 (m, 10H), 2.67–2.24 (m, 1H), 2.17–1.53 (m, 6H).

### Preparation of Compound 18

#### *3-Chloro-5-[[(5-methyl-1,3,4-oxadiazol-2-yl)methylamino]methyl]pyridin-2-amine* (**22fd**)

5-(Aminomethyl)-3-chloro-pyridin-2-amine (**20f**) (36 mg, 0.21 mmol) and 2-(chloromethyl)-5-methyl-1,3,4-oxadiazole (**21d**) (27 mg, 0.21 mmol) were reacted according to General Procedure A. Purification by flash column chromatography (silica column, 0% to 10% 2 M methanolic ammonia in DCM gradient) followed by concentration *in vacuo* afforded amine **22fd** as a yellow gum (24 mg, 0.071 mmol, 35%). HRMS (ESI) [M + H]^+^ found 254.7012, C_10_H_12_ClN_5_O requires 254.7011. ^1^H NMR (400 MHz, DMSO) δ 7.80 (d, *J* = 2.0 Hz, 1H), 7.52 (d, *J* = 2.0 Hz, 1H), 6.14 (s, 2H), 3.80 (s, 2H), 3.54 (s, 2H), 2.86 (br. s, 1H), 2.46 (s, 3H).

#### *Ethyl 2-[[(6-Amino-5-chloro-3-pyridyl)methyl-[(5-methyl-1,3,4-oxadiazol-2-yl)methyl] carbamoyl]amino]acetate* (**23fd**)

Amine **22fd** (24 mg, 0.070 mmol) was reacted according to General Procedure B. Purification by flash column chromatography (silica column, 1% to 5% MeOH in DCM gradient) then purification by preparative HPLC (basic) followed by concentration *in vacuo* afforded urea **23fd** (25 mg, 0.055 mmol, 78%). LCMS (Method A) 1.36 min, 383.0, 385.2 [M + H]^+^, halide isotope splitting pattern observed. ^1^H NMR (400 MHz, DMSO) δ 7.80 (dd, *J* = 2.1, 1.1 Hz, 1H), 7.51–7.44 (m, 1H), 7.32 (t, *J* = 5.7 Hz, 1H), 6.23 (s, 2H), 4.59 (s, 2H), 4.34 (s, 2H), 4.10 (q, *J* = 7.1 Hz, 2H), 3.79 (d, *J* = 5.7 Hz, 2H), 2.40 (s, 3H), 1.19 (t, *J* = 7.1 Hz, 3H).

*2-[5-(Acetylhydrazono)-3-[(6-amino-5-chloro-3-pyridyl)methyl]-2-oxo-imidazolidin-1-yl] acetic acid* (**31**). Urea ester **22fd** (210 mg, 0.55 mmol) was reacted according to General Procedure C. The reaction mixture was concentrated *in vacuo* to afford crude hydantoin **31** as an off-white solid (267 mg, 0.450 mmol, 82%). LCMS (Method A) 1.16 min, 355.2, 357.2 [M + H]^+^, halide isotope splitting pattern. ^1^H NMR (400 MHz, DMSO) δ 9.81 (s, 1H), 7.83 (dd, *J* = 4.4, 2.0 Hz, 1H), 7.46 (t, *J* = 2.0 Hz, 1H), 6.31 (s, 2H), 4.30 (s, 2H), 4.00 (s, 2H), 3.80 (s, 2H), 1.80 (s, 3H).

#### *N-[[1-[(6-amino-5-chloro-3-pyridyl)methyl]-3-[2-[(2R)-2-(2-methylsulfanylphenyl) Pyrrolidin-1-yl]-2-oxo-ethyl]-2-oxo-imidazolidin-4-ylidene]amino]acetamide* (**18**)

Crude acid **31** (64 mg, 0.18 mmol) and (2*R*)-2-(2-methylsulfanylphenyl)pyrrolidine (*R*)-(**26**) (70 mg, 0.36 mmol) were reacted according to General Procedure E. Purification by flash column chromatography (silica column, 0% to 5% 2 M methanolic ammonia in DCM gradient) followed by prep. HLPC purification (basic) and concentration *in vacuo* afforded hydantoin **18** (34 mg, 0.064 mmol, 36%). LCMS (Method B) 1.49 min (100% @ 254 nm), 530.2, 532.2 [M + H]^+^, halide isotope splitting pattern. ^1^H NMR (400 MHz, CDCl_3_) δ 9.56 (s, 0.27H), 9.04 (s, 0.38H), 8.50 (s, 0.35H), 7.88–7.79 (m, 1H), 7.47–6.90 (m, 5H), 5.54–5.34 (m, 1H), 4.96–4.93 (m, 2H), 4.53–3.56 (m, 8H), 2.55- 2.30 (m, 5H), 2.11–1.67 (m, 5H).

### Preparation of Compound 19

#### *N-[[1-[(6-amino-5-chloro-3-pyridyl)methyl]-2-oxo-3-[2-oxo-2-[(2R)-2-(2-bromophenyl) Pyrrolidin-1-yl]ethyl]imidazolidin-4-ylidene]amino]acetamide* (**19**)

Crude acid **31** (64 mg, 0.18 mmol) and (2*R*)-2-(2-bromophenyl)pyrrolidine (*R*)-(**27**) (81 mg, 0.36 mmol) were reacted according to General Procedure E. Purification by flash column chromatography (silica column, 0% to 5% 2 M methanolic ammonia in DCM gradient) followed by prep. HLPC (basic) and concentration *in vacuo* afforded the title compound (**19**) (17 mg, 0.030 mmol, 17%). LCMS (Method B) 1.49 min (100% @ 254 nm), 562.0, 564.0 [M + H]^+^, halide isotope splitting pattern. ^1^H NMR (400 MHz, CDCl_3_) δ 9.72 (s, 0.4H), 9.39 (s, 0.3H), 8.95 (s, 0.3H), 7.87–7.74 (m, 1H), 7.64–7.08 (m, 4H), 7.10–6.94 (m, 1H), 5.50–5.18 (m, 1H), 4.98–4.91 (m, 2H), 4.63–3.67 (m, 8H), 2.63–2.33 (m, 1.3H), 2.16–1.51 (m, 5.7H).

## Supporting Information Available

Experimental procedures and characterization data for the preparation of reagents **20b**–**f**, **21a**, (*R*)-**26**, **27** and hydantoin **28**; copies of ^1^H NMR spectra and LCMS for all final compounds (PDF)
Molecular formula strings and biological data (CSV)
Models of compounds **1**–**2** and **5**–**14** bound to Cyp A, Cyp B, and Cyp D in PDB format (ZIP)

## Abbreviations

CRC: Calcium retention capacity assay
CsA: Cyclosporine A
Cyp: Cyclophilin
FEP: Free energy perturbation
HCC: Hepatocellular carcinoma
HepG2: Human hepatocytoma
K_d_: Dissociation constant
Log D: Distribution constant
MASH: Metabolic dysfunction associated steatohepatitis
MASLD: Metabolic dysfunction associated steaotic liver disease
SPR: Surface plasmon resonance

## References

[ref1] Rinella M. E.; Lazarus J. V.; Ratziu V.; Francque S. M.; Sanyal A. J.; Kanwal F.; Romero D.; Abdelmalek M. F.; Anstee Q. M.; Arab J. P.; et al. A multisociety Delphi consensus statement on new fatty liver disease nomenclature. Hepatology 2023, 78 (6), 1966–1986. 10.1097/HEP.0000000000000520. 37363821 PMC10653297

[ref2] Takahashi Y.; Fukusato T. Histopathology of nonalcoholic fatty liver disease/nonalcoholic steatohepatitis. World J. Gastroenterol. 2014, 20 (42), 15539–15548. 10.3748/wjg.v20.i42.15539. 25400438 PMC4229519

[ref3] Llovet J. M.; Kelley R. K.; Villanueva A.; Singal A. G.; Pikarsky E.; Roayaie S.; Lencioni R.; Koike K.; Zucman-Rossi J.; Finn R. S. Hepatocellular carcinoma. Nat. Rev. Dis. Primers 2021, 7 (1), 6 10.1038/s41572-020-00240-3. 33479224 PMC13537433

[ref4] Romero F. A.; Jones C. T.; Xu Y.; Fenaux M.; Halcomb R. L. The Race to Bash NASH: Emerging Targets and Drug Development in a Complex Liver Disease. J. Med. Chem. 2020, 63 (10), 5031–5073. 10.1021/acs.jmedchem.9b01701. 31930920

[ref5] Harrison S. A.; Bedossa P.; Guy C. D.; Schattenberg J. M.; Loomba R.; Taub R.; Labriola D.; Moussa S. E.; Neff G. W.; Rinella M. E.; et al. A Phase 3, Randomized, Controlled Trial of Resmetirom in NASH with Liver Fibrosis. N. Engl. J. Med. 2024, 390 (6), 497–509. 10.1056/NEJMoa2309000. 38324483

[ref6] Wang P.; Heitman J. The cyclophilins. Genome Biol. 2005, 6 (7), 226 10.1186/gb-2005-6-7-226. 15998457 PMC1175980

[ref7] Nigro P.; Pompilio G.; Capogrossi M. C. Cyclophilin A: a key player for human disease. Cell Death Dis. 2013, 4, e888 10.1038/cddis.2013.410. 24176846 PMC3920964

[ref8] Naoumov N. V. Cyclophilin inhibition as potential therapy for liver diseases. J. Hepatol. 2014, 61 (5), 1166–1174. 10.1016/j.jhep.2014.07.008. 25048953

[ref9] Ure D. R.; Trepanier D. J.; Mayo P. R.; Foster R. T. Cyclophilin inhibition as a potential treatment for nonalcoholic steatohepatitis (NASH). Expert Opin. Investig. Drugs 2020, 29 (2), 163–178. 10.1080/13543784.2020.1703948. 31868526

[ref10] Flaxman H. A.; Chrysovergi M.-A.; Han H.; Kabir F.; Lister R. T.; Chang C.-F.; Yvon R.; Black K. E.; Weigert A.; Savai R.; Egea-Zorrilla A.; Pardo-Saganta A.; Lagares D.; Woo C. M. Sanglifehrin A mitigates multiorgan fibrosis by targeting the collagen chaperone cyclophilin B. JCI Insight 2024, 9 (15), e171162 10.1172/jci.insight.171162. 38900587 PMC11383833

[ref11] Angulo P.; Kleiner D. E.; Dam-Larsen S.; Adams L. A.; Bjornsson E. S.; Charatcharoenwitthaya P.; Mills P. R.; Keach J. C.; Lafferty H. D.; Stahler A.; Haflidadottir S.; Bendtsen F.; et al. Liver Fibrosis, but No Other Histologic Features, Is Associated With Long-term Outcomes of Patients With Nonalcoholic Fatty Liver Disease. Gastroenterology 2015, 149 (2), 389 10.1053/j.gastro.2015.04.043. 25935633 PMC4516664

[ref12] Sweeney Z. K.; Fu J.; Wiedmann B. From Chemical Tools to Clinical Medicines: Nonimmunosuppressive Cyclophilin Inhibitors Derived from the Cyclosporin and Sanglifehrin Scaffolds. J. Med. Chem. 2014, 57 (17), 7145–7159. 10.1021/jm500223x. 24831536

[ref13] Wang H.; Zhang Y.; Wang T.; You H.; Jia J. N-methyl-4-isoleucine cyclosporine attenuates CCl_4_-induced liver fibrosis in rats by interacting with cyclophilin B and D. J. Gastroenterol. Hepatol. 2011, 26 (3), 558–567. 10.1111/j.1440-1746.2010.06406.x. 21332552

[ref14] Kuo J.; Bobardt M.; Chatterji U.; Mayo P. R.; Trepanier D. J.; Foster R. T.; Gallay P.; Ure D. R. A Pan-Cyclophilin Inhibitor, CRV431, Decreases Fibrosis and Tumor Development in Chronic Liver Disease Models. J. Pharmacol. Exp. Ther. 2019, 371 (2), 231–241. 10.1124/jpet.119.261099. 31406003 PMC6815936

[ref15] Harrison S. A.; Mayo P. R.; Hobbs T. M.; Canizares C.; Foster E. P.; Zhao C.; Ure D. R.; Trepanier D. J.; Greytok J. A.; Foster R. T. Rencofilstat, a cyclophilin inhibitor: A phase 2a, multicenter, single-blind, placebo-controlled study in F2/F3 NASH. Hepatol. Commun. 2022, 6 (12), 3379–3392. 10.1002/hep4.2100. 36271849 PMC9701462

[ref16] Peterson A. A.; Rangwala A. M.; Thakur M. K.; Ward P. S.; Hung C.; Outhwaite I. R.; Chan A. I.; Usanov D. L.; Mootha V. K.; Seeliger M. A.; Liu D. R.; et al. Discovery and molecular basis of subtype-selective cyclophilin inhibitors. Nat. Chem. Biol. 2022, 18 (11), 1184 10.1038/s41589-022-01116-1. 36163383 PMC9596378

[ref17] Davis T. L.; Walker J. R.; Campagna-Slater V.; Finerty P. J. Jr.; Paramanathan R.; Bernstein G.; MacKenzie F.; Tempel W.; Hui O.; Lee W. H.; et al. Structural and Biochemical Characterization of the Human Cyclophilin Family of Peptidyl-Prolyl Isomerases. PLoS. Biol. 2010, 8 (7), e1000439 10.1371/journal.pbio.1000439. 20676357 PMC2911226

[ref18] Schiene-Fischer C.; Fischer G.; Braun M. Non-Immunosuppressive Cyclophilin Inhibitors. Angew. Chem., Int. Ed. 2022, 61 (39), e202201597 10.1002/anie.202201597. PMC9804594 35290695

[ref19] De Simone A.; Georgiou C.; Ioannidis H.; Gupta A. A.; Juarez-Jimenez J.; Doughty-Shenton D.; Blackburn E. A.; Wear M. A.; Richards J. P.; Barlow P. N.; et al. A computationally designed binding mode flip leads to a novel class of potent tri-vector cyclophilin inhibitors. Chem. Sci. 2019, 10 (2), 542–547. 10.1039/C8SC03831G. 30746096 PMC6335623

[ref20] Stone M. P.; Huang H.; Brown K. L.; Shanmugam G. Chemistry and Structural Biology of DNA Damage and Biological Consequences. Chem. Biodivers. 2011, 8 (9), 1571–1615. 10.1002/cbdv.201100033. 21922653 PMC3714022

[ref21] Ahmed-Belkacem A.; Colliandre L.; Ahnou N.; Nevers Q.; Gelin M.; Bessin Y.; Brillet R.; Cala O.; Douguet D.; Bourguet W.; et al. Fragment-based discovery of a new family of non-peptidic small-molecule cyclophilin inhibitors with potent antiviral activities. Nat. Commun. 2016, 7, 12777 10.1038/ncomms12777. 27652979 PMC5036131

[ref22] Kuhn M.; Firth-Clark S.; Tosco P.; Mey A. S. J. S.; Mackey M.; Michel J. Assessment of Binding Affinity via Alchemical Free-Energy Calculations. J. Chem. Inf. Model. 2020, 60 (6), 3120–3130. 10.1021/acs.jcim.0c00165. 32437145

[ref23] Shamovsky I.; Ripa L.; Borjesson L.; Mee C.; Norden B.; Hansen P.; Hasselgren C.; O’Donovan M.; Sjo P. Explanation for Main Features of Structure-Genotoxicity Relationships of Aromatic Amines by Theoretical Studies of Their Activation Pathways in CYP1A2. J. Am. Chem. Soc. 2011, 133 (40), 16168–16185. 10.1021/ja206427u. 21894985

[ref24] Bentzien J.; Hickey E. R.; Kemper R. A.; Brewer M. L.; Dyekjaer J. D.; East S. P.; Whittaker M. An in Silico Method for Predicting Ames Activities of Primary Aromatic Amines by Calculating the Stabilities of Nitrenium Ions. J. Chem. Inf. Model. 2010, 50 (2), 274–297. 10.1021/ci900378x. 20078034

[ref25] Georgiou C.; McNae L.; Wear M.; Ioannidis H.; Michel J.; Walkinshaw M. Pushing the Limits of Detection of Weak Binding Using Fragment-Based Drug Discovery: Identification of New Cyclophilin Binders. J. Mol. Biol. 2017, 429 (16), 2556–2570. 10.1016/j.jmb.2017.06.016. 28673552

[ref35] Wear M. A.; Nowicki M. W.; Blackburn E. A.; McNae I. W.; Walkinshaw M. D. Thermo-kinetic analysis space expansion for cyclophilinligand interactions - identification of a new nonpeptide inhibitor using Biacore T200. Febs Open Bio 2017, 7 (4), 533–549. 10.1002/2211-5463.12201. PMC5377415 28396838

[ref26] Yadav J.; Reddy B.; Meraj S.; Vishnumurthy P.; Narsimulu K.; Kunwar A. Montmorillonite clay catalyzed synthesis of enantiomerically pure 1,2,3,4-tetrahydroquinolines. Synthesis-Stuttgart 2006, 2006 (17), 2923–2926. 10.1055/s-2006-942528.

[ref27] Cossar P. J.; Hizartzidis L.; Simone M. I.; McCluskey A.; Gordon C. P. The expanding utility of continuous flow hydrogenation. Org. Biomol. Chem. 2015, 13 (26), 7119–7130. 10.1039/C5OB01067E. 26073166

[ref28] Amssoms K.; Oza S. L.; Ravaschino E.; Yamani A.; Lambeir A.-M.; Rajan P.; Bal G.; Rodriguez J. B.; Fairlamb A. H.; Augustyns K.; et al. Glutathione-like tripeptides as inhibitors of glutathionylspermidine synthetase. Part 1: Substitution of the glycine carboxylic acid group. Bioorg. Med. Chem. Lett. 2002, 12 (18), 2553–2556. 10.1016/S0960-894X(02)00489-4. 12182858

[ref29] Kang S. Y.; Lee S.-H.; Seo H. J.; Jung M. E.; Ahn K.; Kim J.; Lee J. Tetrazole-biarylpyrazole derivatives as cannabinoid CB1 receptor antagonists. Bioorg. Med. Chem. Lett. 2008, 18 (7), 2385–2389. 10.1016/j.bmcl.2008.02.061. 18337096

[ref30] Purchase C. F. Ii; White A. D. Alkylation of Tetrazoles Using Mitsunobu Conditions. Synth. Commun. 1996, 26 (14), 2687–2694. 10.1080/00397919608004585.

[ref31] Bélai I. A versatile method for the synthesis of substituted 1-aminohydantoin derivatives. Tetrahedron Lett. 2003, 44 (40), 7475–7477. 10.1016/j.tetlet.2003.08.029.

[ref32] Gianquinto E.; Sodano F.; Rolando B.; Kostrzewa M.; Allarà M.; Mahmoud A. M.; Kumar P.; Spyrakis F.; Ligresti A.; Chegaev K. N-[1,3-Dialkyl(aryl)-2-oxoimidazolidin-4-ylidene]-aryl(alkyl)sulphonamides as Novel Selective Human Cannabinoid Type 2 Receptor (hCB2R) Ligands; Insights into the Mechanism of Receptor Activation/Deactivation. Molecules 2022, 27, 8152 10.3390/molecules27238152. 36500256 PMC9738591

[ref33] Micetich R. G. Lithiation of 5-membered heteroaromatic compounds - methyl substituted 1,2-azoles, oxadiazoles, and thiadiazoles. Can. J. Chem. 1970, 48 (13), 2006 10.1139/v70-334.

[ref34] Katritzky A. R.; Tymoshenko D. O.; Chen K.; Fattah A. A.; Schantl J. Ring and side chain reactivities of 1-(1,3,4 oxadiazol-2-ylmethyl)-1H-benzotriazoles. Arkivoc 2005, 2, 101–108. 10.3998/ark.5550190.0002.212.

[ref36] Graedler U.; Schwarz D.; Blaesse M.; Leuthner B.; Johnson T. L.; Bernard F.; Jiang X.; Marx A.; Gilardone M.; Lemoine H.; et al. Discovery of novel Cyclophilin D inhibitors starting from three dimensional fragments with millimolar potencies. Bioorg. Med. Chem. Lett. 2019, 29 (23), 126717 10.1016/j.bmcl.2019.126717. 31635932 PMC7195332

[ref37] Müller F. A.; Sturla S. J. Human in vitro models of nonalcoholic fatty liver disease. Curr. Opin. Toxicol. 2019, 16 (Sp. Iss. SI), 9–16. 10.1016/j.cotox.2019.03.001.

[ref38] Bonora M.; Giorgi C.; Pinton P. Molecular mechanisms and consequences of mitochondrial permeability transition. Nat. Rev. Mol. Cell Biol. 2022, 23 (4), 266–285. 10.1038/s41580-021-00433-y. 34880425

[ref39] Stauffer W. T.; Goodman A. Z.; Bobardt M.; Ure D. R.; Foster R. T.; Gallay P.; Islam F. Mice lacking cyclophilin B, but not cyclophilin A, are protected from the development of NASH in a diet and chemical-induced model. PLoS One 2024, 19 (3), e0298211 10.1371/journal.pone.0298211. 38427624 PMC10906846

[ref40] Williamson B.; Harlfinger S.; McGinnity D. F. Evaluation of the Disconnect between Hepatocyte and Microsome Intrinsic Clearance and In Vitro In Vivo Extrapolation Performance. Drug Metab. Dispos. 2020, 48 (11), 1137–1146. 10.1124/dmd.120.000131. 32847864

[ref41] Ni S.; Yuan Y.; Huang J.; Mao X.; Lv M.; Zhu J.; Shen X.; Pei J.; Lai L.; Jiang H.; et al. Discovering Potent Small Molecule Inhibitors of Cyclophilin A Using de Novo Drug Design Approach. J. Med. Chem. 2009, 52 (17), 5295–5298. 10.1021/jm9008295. 19691347

[ref42] Daum S.; Schumann M.; Mathea S.; Aumüller T.; Balsley M. A.; Constant S. L.; de Lacroix B. F.; Kruska F.; Braun M.; Schiene-Fischer C. Isoform-Specific Inhibition of Cyclophilins. Biochemistry 2009, 48 (26), 6268–6277. 10.1021/bi9007287. 19480458 PMC2753677

[ref43] Shore E. R.; Awais M.; Kershaw N. M.; Gibson R. R.; Pandalaneni S.; Latawiec D.; Wen L.; Javed M. A.; Criddle D. N.; Berry N.; et al. Small Molecule Inhibitors of Cyclophilin D To Protect Mitochondrial Function as a Potential Treatment for Acute Pancreatitis. J. Med. Chem. 2016, 59 (6), 2596–2611. 10.1021/acs.jmedchem.5b01801. 26950392

