# Supplementary material for: Optimization of Cyclophilin B-Targeted Tri-vector Inhibitors for Novel MASH Treatments
Source: J Med Chem. 2025 Mar 12;68(6):6815–31. doi: 10.1021/acs.jmedchem.5c00301 (PMC11956012; doi:10.1021/acs.jmedchem.5c00301)
Supplement: Supplementary file 1 — jm5c00301_si_001.pdf [file jm5c00301_si_001.pdf]

Optimisation of Cyclophilin B-targeted Tri-vector Inhibitors for Novel MASH Treatments

Maria-Eleni Kouridaki[a], Jonathan Gillespie[b], John Robinson[b], Tanya Mathie[b], Laura Bain[b], Duncan McArthur[b], Angus Morrison[b], Daniel B. Greenslade[a], Michail Papadourakis[a#], Kasia Maj[c], Kate Cameron[c], Darryl Turner[d], Scott P. Webster[e], Martin A. Wear[f], Dahlia Doughty-Shenton[g], Alison N. Hulme[a], and Julien Michel[a*]

[a] EaStCHEM School of Chemistry, David Brewster Road, EH9 3FJ, Scotland, UK

[b] BioAscent Discovery Ltd., Newhouse, Lanarkshire ML1 5UH, Scotland, UK

[c] Cytochroma Ltd., Roslin Innovation Centre, Easter Bush Estate, Edinburgh EH25 9RG, Scotland, UK

[d] Concept Life Science Ltd., Nine, 9 Little France Road, Edinburgh Bioquarter, Edinburgh EH16 4UX, Scotland, UK

[e] Centre for Cardiovascular Science, Queen's Medical Research Institute, 47 Little France Crescent, Edinburgh EH16 4TJ, Scotland, UK

[f] The Edinburgh Protein Production Facility (EPPF), University of Edinburgh, Level 3 Michael Swann Building, King's Buildings, Max Born Crescent, Edinburgh EH9 3FF, Scotland, UK

[g] Centre for Reproductive Health, Institute for Regeneration and Repair, 4-5 Little France Drive, Edinburgh Bioquarter, Edinburgh EH16 4UU, Scotland, UK

Corresponding author email: julien.michel@ed.ac.uk

Table of Contents

Molecular modelling S2

Organic syntheses S8

Biological Experiments S88

Molecular modelling

Cyp A affinity predictions

FlareFEP v5 was used using default protocols to compute relative binding free energies.

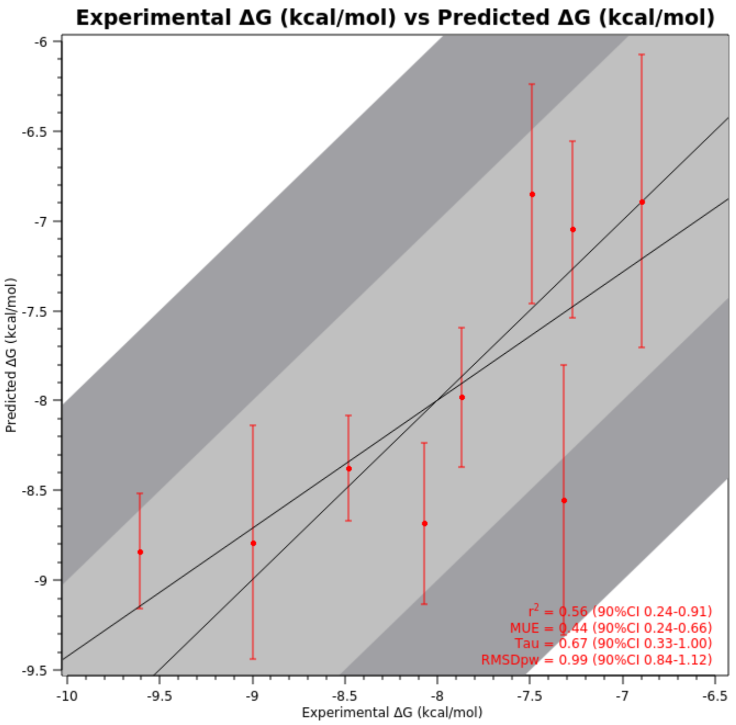

Figure S1. Affinity model for a dataset of nine CypA inhibitors previously reported in the literature.

Binding selectivity analysis

FlareFEP v8 was used in benchmark mode to compute the relative binding free energies of compounds 1,2,5-14. The X-tray structure of 1/Cyp A (PDB 6GJN) was used as starting point to manually model all analogues. Models for Cyp B and Cyp D were constructed by aligning X-ray structures 1CYN (Cyp B) and 4J5B (Cyp D) to the Cyp A models, and performing a round of energy minimisation after deletion of crystallographic water molecules that clashed with the ligands.

For all FEP calculations the ligands were parameterised with OpenFF 2.1.0, and custom torsional parameters were derived using the DFT/GFN2-xTB methodology. The GCNCMC was used to optimise water placement around the ligands during equilibration. Intermediates were automatically added to the planned RBFE network to maximise the similarity of all molecules involved in pairwise transformations. All edges were processed bidirectionally. Noisy edges leading to poor cycle closure were repeated, and additional edges added to the RBFE network to reduce the overall uncertainty of the binding affinity estimates.

The final computed binding affinities used for the selectivity analysis are shown in Table S5. 3D models used as input for the FlareFEP calculations are available as Supporting Information.

Table S1. FEP model for Pro pocket optimisation

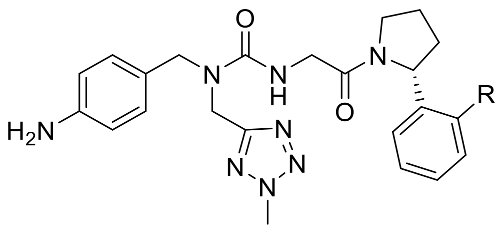

R	FEP ΔG / kcal.mol^{-1}
SMe	-9.2
Br	-8.9
Cl	-8.6
CF_3	-8.6
cyclopropyl	-8.6
OMe	-8.4
Et	-8.3
^{i}Pr	-8.3
^{t}Bu	-7.7
Me	-7.5

Lead compound **1** highlighted in grey. The statistical uncertainties of the binding affinity estimates are approximately ± 0.5 kcal.mol^{-1}.

Table S2. FEP model for Abu pocket optimisation.*

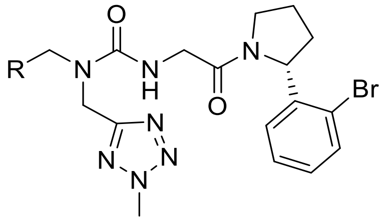

R	FEP ΔG / kcal.mol^{-1}	R	FEP ΔG / kcal.mol^{-1}
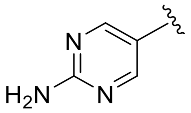	-9.5 (3.5)	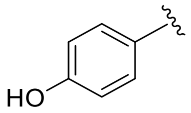	-7.5
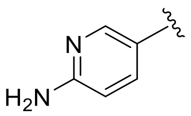	-9.5 (7.2)	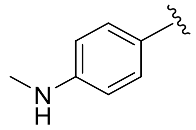	-7.2
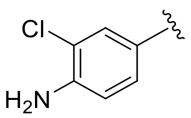	-9.2 (3.2)	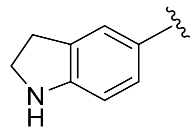	-7.1
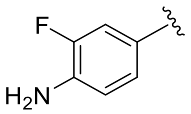	-8.9 (3.4)	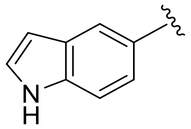	-6.7
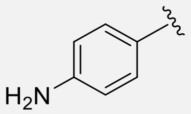	-8.5 (5.0)	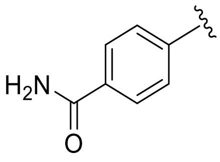	-6.5
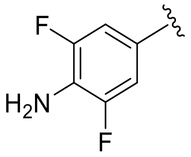	-8.0 (2.3)		

*Values in parentheses are strongest basic pKa estimates for the R group (Chemicalize model).

Lead compound **1** highlighted in grey. The FEP affinity estimate for **1** differs slightly from the data in Table S1 because it was computed from a different set of relative binding free energy estimates. The statistical uncertainties of the binding affinity estimates are approximately ± 0.5 kcal.mol^{-1}.

Table S3. FEP model for 3 o'clock pocket optimisation.*

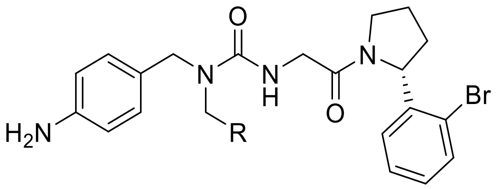

R	FEP ΔG / kcal.mol^{-1}	R	FEP ΔG / kcal.mol^{-1}
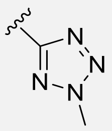	-8.5 (2.7)	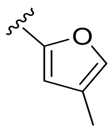	-8.1 (4.5)
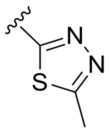	-8.5 (4.3)	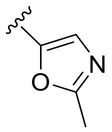	-7.9 (3.9)
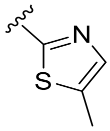	-8.4 (4.9)	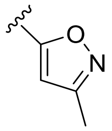	-7.9 (3.9)
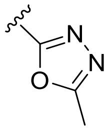	-8.3 (3.3)	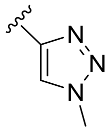	-7.8 (3.3)
		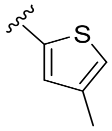	-7.8 (5.6)

*Values in parentheses are calculated cLogP values (Wildman-Crippen model)

Lead compound **1** highlighted in grey. The FEP affinity estimate for **1** differs slightly from the data in Table S1 because it was computed from a different set of relative binding free energy estimates. The statistical uncertainties of the binding affinity estimates are approximately ± 0.5 kcal.mol^{-1}.

Table S4. Compounds with improved FEP and cLogP ~2 – 5 for synthetic prioritization.*

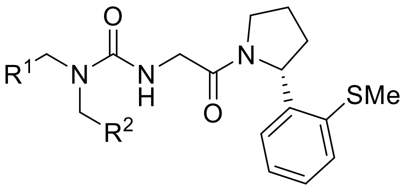

		R^2			
		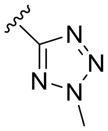	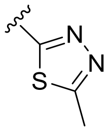	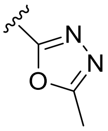	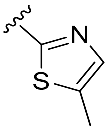
R^1	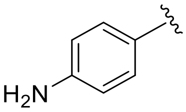	-9.6 2.6	--	--	--
	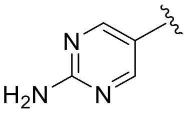	-11.3 1.4	-11.3 3.0	-11.0 2.0	-11.6 3.6
	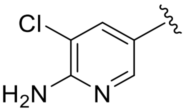	-10.9 2.7	-10.4 4.3	-10.5 3.3	-10.7 4.9
	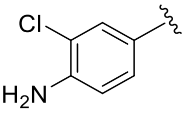	-10.1 3.3	-9.9 4.9	-9.7 3.9	-9.9 5.5

* FEP calculation shown in red (kcal.mol^{-1}), cLogP shown in green. The FEP affinity estimate for the top left compound differs slightly from the data in Table S1 because it was computed from a different set of relative binding free energy estimates. The statistical uncertainties of the binding affinity estimates are approximately ± 0.5 kcal.mol^{-1}.

Table S5. Computed binding free energies for compounds **1**, **2**, **5-14**.

	CypA ΔG	Uncertainty	CypB ΔG	Uncertainty	CypD ΔG	Uncertainty
**1**	-7.1	0.5	-8.4	0.7	-7.4	0.3
**2**	-8.5	0.3	-9.4	0.4	-8.5	0.1
**5**	-9.2	0.3	-10.3	0.3	-9.7	0.1
**6**	-7.7	0.4	-6.7	0.6	-5.8	0.2
**7**	-6.8	0.9	-6.8	1.0	-6.1	0.2
**8**	-8.3	0.5	-8.3	0.6	-8.2	0.1
**9**	-8.3	0.7	-8.3	0.5	-8.8	0.4
**10**	-8.5	0.5	-9.2	0.6	-8.6	0.1
**11**	-9.8	0.3	-11.1	0.4	-10.0	0.4
**12**	-9.5	0.5	-11.3	0.6	-9.6	0.2
**13**	-7.9	0.5	-11.5	0.6	-10.0	0.2
**14**	-9.1	0.4	-10.2	0.5	-9.5	0.1

Binding affinities in kcal.mol^{-1}

Organic syntheses

1.1 Preparation of Abu-pocket 4-amino-benzylamine reagents

***tert*-Butyl *N*-[[(1*S*,9*R*,10*S*)-10-hydroxy-12-oxa-8-azatricyclo[7.3.1.0^{2},^{7}]trideca-2,4,6-trien-4-yl]methyl]carbamate (S1)**

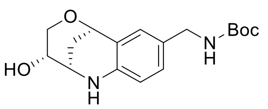

(4*S*,5*R*)-5-(Hydroxymethyl)tetrahydrofuran-2,4-diol (1.00 g, 7.46 mmol), *tert*-butyl *N*-[(4-aminophenyl)methyl]carbamate (2.49 g, 11.2 mmol), and montmorillonite K10 (7.5 g, 7.5 mmol) were placed in a round-bottom flask, and MeCN (30 mL) was added. The reaction mixture was purged with argon and stirred at room temperature for 7 days. The crude reaction mixture was filtered through a celite pad, which was washed with EtOAc (30 mL x 3). The combined organics (2.9 g) were pre-absorbed onto silica and purified by flash column chromatography on the Biotage Isolera (100 g silica, 30 to 70% EtOAc/Heptane) to afford compound **S1** as a light-colored gum (0.710 g, 2.21 mmol, 30%). HRMS (ESI) $[M+H]^+$ calculated for $C_{16}H_{22}N_6O_3Na$ 321.3901; found 321.3905 $[M + H]^+$. ^1H NMR (400 MHz, $CDCl_3$) δ 7.14 – 7.00 (m, 2H), 6.52 (d, *J* = 8.1 Hz, 1H), 4.71 (dd, *J* = 3.5, 1.8 Hz, 1H), 4.38 (d, *J* = 4.4 Hz, 1H), 4.19 (d, *J* = 5.6 Hz, 2H), 3.65 (s, 1H), 3.60 – 3.46 (m, 3H), 2.61 – 2.51 (m, 1H), 2.22 (d, *J* = 7.2 Hz, 1H), 1.58 – 1.51 (m, 1H), 1.46 (s, 9H).

***tert*-Butyl *N*-[[(1*S*,9*R*,10*S*)-10-[*tert*-butyl(dimethyl)silyl]oxy-12-oxa-8-azatricyclo [7.3.1.0^{2},^{7}]trideca-2,4,6-trien-4-yl]methyl]carbamate (S2)**

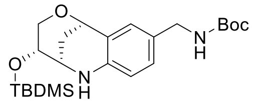

Alcohol **S1** (620 mg, 1.90 mmol) was dissolved in DCM (20 mL). The solution was then cooled in an ice bath. Subsequently, imidazole (0.4 mL, 9.6 mmol) and *tert*-butyldimethylsilyl chloride (870 mg, 5.80 mmol) were added. The mixture was stirred at 0°C for 10 min before being allowed to reach room temperature. The reaction continued to stir overnight. Afterwards, the crude mixture was partitioned between DCM and water. The two phases were mixed and separated. The aqueous phase was extracted with DCM two more times. The combined organic layers were dried using Na_2SO_4, filtered, and concentrated to yield a crude beige solid. This crude product was further purified using flash column chromatography on the Biotage Isolera (40 g silica, 0-30% EtOAc/Heptane gradient) to afford compound **S2** as a colorless solid (680 mg, 1.56 mmol, 82%). LCMS (Method B): 2.157 min, 435.2 $[M+H]^+$. ^1H NMR (400 MHz, $CDCl_3$) δ 7.01 – 6.94 (m, 2H), 6.41 (d, *J* = 8.0 Hz, 1H), 4.60 (s, 2H), 4.23 (d, *J* = 4.1 Hz, 1H), 4.10 (s, 2H), 3.44 – 3.36 (m, 3H), 3.29 (dd, *J* = 13.1, 1.9 Hz, 1H), 2.59 (dt, *J* = 13.1, 2.8 Hz, 1H), 1.37 (s, 10H), 0.85 (s, 9H), 0.01 (s, 3H), 0.00 (s, 3H).

[(1*S*,9*R*,10*S*)-10-[*tert*-Butyl(dimethyl)silyl]oxy-12-oxa-8-azatricyclo[7.3.1.0^{2},^{7}]trideca-2,4,6-trien-4-yl]methanamine (20b)

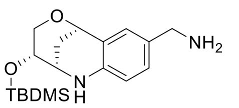

Carbamate **S2** (680 mg, 1.60 mmol) was dissolved in dry DCM (40 mL). The vessel was purged with argon and cooled to approximately -8°C, which further dropped to -10°C overnight using the Julabo chiller unit. Trifluoroacetic acid (2.6 mL, 34 mmol) was added dropwise, and the solution was stirred overnight. The reaction was quenched with a dropwise addition of 2 M NaOH (25 mL), ensuring the temperature remained below 0°C. The mixture was then diluted with water and DCM (25 mL). The phases were mixed and subsequently separated. The aqueous layer underwent two additional extractions with DCM (2 x 25 mL). The combined organics were dried, filtered, and concentrated to yield approximately 650 mg of a yellow gum. The crude product was purified using flash column chromatography on the Biotage Isolera (20 g silica, 0 to 10% 2 M NH_3-MeOH/DCM) to afford amine **20b** as a light-colored gum, which solidified upon standing (340 mg, 1.1 mmol, 67%). HRMS (ESI) $[M+H]^+$ found 335.5510, $C_{18}H_{30}N_2O_2Si$ requires 335.5510. ^1H NMR (400 MHz, $CDCl_3$) δ 7.12 – 7.05 (m, 2H), 6.55 – 6.48 (m, 1H), 4.70 (dd, *J* = 3.3, 1.8 Hz, 1H), 4.30 (s, 1H), 3.74 (s, 2H), 3.53 – 3.44 (m, 3H),

3.39 (dd, J = 13.1, 1.9 Hz, 1H), 2.72 – 2.63 (m, 1H), 1.48 (dddt, J = 12.8, 3.4, 2.1, 1.2 Hz, 1H), 0.93 (s, 9H), 0.09 (d, J = 3.8 Hz, 6H).

4-(Aminomethyl)-2,6-difluoro-aniline (20c)

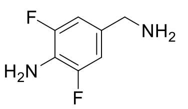

4-Amino-3,5-difluoro-benzonitrile (305 mg, 1.98 mmol) was dissolved in 2% AcOH/MeOH (39.6 mL) and the mixture passed through the H-Cube equipped with a large 10% Pd-C catalyst, 50 bar, 70°C, and a flow of 1 ml/min. The crude reaction was concentrated to dryness and the residue dissolved in DCM/MeOH and passed through an SCX cartridge which was washed successively with DCM then MeOH and eluted with 2 M-NH_3/MeOH to afford amine **20c** as a light-colored gum which crystalised on standing (77 mg, 0.49 mmol, 24%). HRMS (ESI) $[M+H]^+$ found 156.1543, $C_7H_8F_2N_2$ requires 156.1543. ^{1}H NMR (400 MHz, $CDCl_3$) δ 6.85 – 6.70 (m, 2H), 3.74 (m, 4H).

4-(Aminomethyl)-2-chloro-aniline (20d)

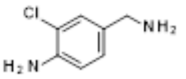

4-Amino-3-chloro-benzonitrile (500 mg, 3.28 mmol) was dissolved in THF (6.5 mL) and the solution was placed under argon and cooled in an ice-bath to 0°C. $LiAlH_4$ (2.4M in THF, 3.41 mL, 8.19 mmol) was added dropwise and the reaction stirred at 0°C for 1 h. The reaction was allowed to warm to room temperature over 1 h and stirred for 72 h. The reaction mixture was cooled to 0°C and water (350 μL) NaOH solution (2 M aq., 350 μL) and water (2 x 1 mL) were added sequentially. EtOAc (10 mL) was added and the mixture stirred at room temperature for 40 min. The afforded suspension was filtered with EtOAc washing and the filtrate concentrated *in vacuo* to afford amine **20d** as a yellow oil (440 mg, 2.82 mmol, 86%). HRMS (ESI) $[M+H]^+$ found 157.0610, $C_7H_9ClN_2$ requires 157.0610; ^{1}H NMR (400 MHz, DMSO) δ 7.14 (d, J = 2.0 Hz, 1H), 6.94 (dd, J = 8.2, 2.0 Hz, 1H), 6.71 (d,J= 8.2 Hz, 1H), 5.10 (s, 2H), 3.53 (s, 2H), 1.75 (s, 2H).

***tert*-Butyl *N*-*tert*-butoxycarbonyl-*N*-(5-cyanopyrimidin-2-yl)carbamate (S3)**

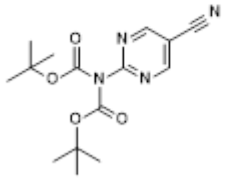

2-Aminopyrimidine-5-carbonitrile (100 mg, 0.830 mmol), triethylamine (0.290 mL, 2.08 mmol) and 4-dimethylaminopyridine (10 mg, 0.080 mmol) were suspended in DMF (1.5 mL). Di-*tert*-butyl dicarbonate (454 mg, 2.08 mmol) was added and the reaction stirred at room temperature for 20 h. The reaction mixture was partitioned between EtOAc (50 mL) and saturated ammonium chloride solution (50 mL). Organics were washed with water (2 x 50 mL) and brine (50 mL), dried over sodium sulfate, filtered, and concentrated *in vacuo*. Purification by flash column chromatography (silica column, 0% to 50% EtOAc in heptane gradient) followed by concentration *in vacuo*, afforded bis-carbamate **S3** as a white solid (146 mg, 0.456 mmol, 55%). LCMS (Method A): 2.69 min, 165.2 $[M\text{-}Boc\text{-}^tBu]^+$. ^{1}H NMR (400 MHz, DMSO) δ 9.32 (s, 2H), 1.44 (s, 18H).

***tert*-Butyl *N*-[5-(aminomethyl)pyrimidin-2-yl]-*N*-*tert*-butoxycarbonyl-carbamate (20e)**

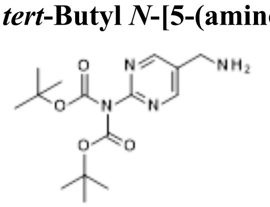

Biscarbamate **S3** (145 mg, 0.450 mmol) was dissolved in ammonia (2 M in MeOH, 10 mL, 20 mmol). The solution was passed through a H-Cube hydrogenation system equipped with a Raney Nickel catalyst cartridge at a flow rate of 1 mL / min, 70°C, under 50 bar of hydrogen pressure. The eluent was concentrated *in vacuo* to afford crude product. Purification by flash column chromatography (silica column, 0% to 10% 2 M methanolic ammonia in DCM gradient) followed by concentration *in vacuo* afforded amine **20e** (64 mg,0.20 mmol, 44%). LCMS (Method A): 1.67 min, 169.2 $[M\text{-}Boc\text{-}^tBu]^+$. Mass due to loss of 1 Boc and carboxylate fragment of the other. ^{1}H NMR (400 MHz, $CDCl_3$) δ 8.74 (s, 2H), 3.97 (s, 2H), 1.46 (s, 18H). Exchangeable NH_2 protons not observed.

5-(Aminomethyl)-3-chloro-pyridin-2-amine (20f)

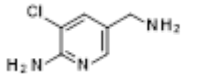

A solution of 6-amino-5-chloro-pyridine-3-carbonitrile (2.0 g, 13 mmol) dissolved in MeOH (130 mL) was passed through a H-Cube hydrogenation system equipped with a Raney Nickel cartridge at a flow rate of 1 ml / min, at 70°C, under 50 bar of hydrogen pressure. The eluent was concentrated *in vacuo* to afford a pale yellow oil (1.98 g). Purification by flash column chromatography (silica column, 0% to 5% 2 M methanolic ammonia in DCM gradient) followed by concentration *in vacuo* afforded amine **20f** (842 mg, 4.81 mmol, 37%). LCMS (Method B) 1.26 min, 157.2, 159.2 $[M+H]^+$, halide isotope splitting pattern. ^1H NMR (400 MHz, DMSO) δ 7.81 (d, *J* = 2.0 Hz, 1H), 7.55 (d, *J* = 2.0 Hz, 1H), 6.03 (s, 2H), 3.53 (s, 2H).

1.2 Preparation of 3 o'clock pocket heteroaryl alkylating reagents

5-(Chloromethyl)-2-methyl-tetrazole (21a)

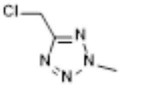

5-(Chloromethyl)-2*H*-tetrazole (120 mg, 1.01 mmol) was suspended in ether (2 mL) and the reaction cooled in an ice bath. Diazomethyl(trimethyl)silane (2 M in hexanes) (0.560 mL, 1.11 mmol) was added dropwise and the solution stirred at 0°C before allowing to come to room temperature and stirring for 3 h. The crude reaction was blown to dryness under a stream of air and pre-absorbed on to silica before purification by flash column chromatography on the Biotage Isolera (12 g silica, 0 to 50% EtOAc/Heptane) to provide 2-methyl tetrazole **21a** as colorless gum (37 mg, 0.28 mmol, 27%) and a slower-eluting compound, identified by nOe as the 1-methyl regioisomeric compound (37 mg, 0.28 mmol, 27%). HRMS (ESI) $[M+H]^+$ calculated for $C_3H_5ClN_4$ 133.0321; found 133.0321 $[M + H]^+$. ^1H NMR (400 MHz, CDCl3) δ 4.77 (s, 2H), 4.36 (s, 3H).

2-(Chloromethyl)-5-methylthiazole hydrochloride **21b**, 2-(chloromethyl)-5-methyl-1,3,4-thiadiazole **21c**, 2-(chloromethyl)-5-methyl-1,3,4-oxadiazole **21d**, propargyl bropmide **21e** and chloroacetonitrile **21f** were all obtained from commercial sources.

1.3 Preparation of Pro-pocket pyrrolidine reagents

***tert*-Butyl *N*-[4-(2-methylsulfanylphenyl)-4-oxo-butyl]carbamate (S4)**

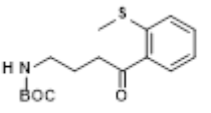

1-Bromo-2-methylsulfanyl-benzene (0.36 mL, 2.7 mmol) was dissolved in THF (5 mL) and the vessel purged with argon and cooled to -78°C in a dry ice/acetone bath. n-Butyllithium (2.7M in hexanes) (1.0 mL, 2.7 mmol) was added dropwise maintaining an internal temperature below -70°C and stirring continued at -78°C for 1 h. *tert*-Butyl 2-oxopyrrolidine-1-carboxylate (0.46 mL, 2.7 mmol) was added dropwise and the cooling bath allowed to decay to room temperature overnight. The reaction was quenched by addition of saturated ammonium chloride and extracted twice with EtOAc. Combined organics were dried (Na_2SO_4), filtered and concentrated to give crude product as a light-colored gum (900 mg) which was purified by flash column chromatography on the Biotage Isolera (40 g silica, 0 to 25% EtOAc/Heptane) to give carbamate **S4** as a colorless solid (454 mg, 1.47 mmol, 54%). LCMS (Method B): 1.842 min, 210.2 $[M+H]^+$. ^1H NMR (400 MHz, CDCl3) δ 7.84 (dd,J= 7.9, 1.5 Hz, 1H), 7.49 (ddd, *J* = 8.1, 7.3, 1.5 Hz, 1H), 7.39 – 7.32 (m, 1H), 7.22 (ddd, *J* = 7.8, 7.2, 1.2 Hz, 1H), 4.66 (s, 1H), 3.24 (q, *J* = 6.6 Hz, 2H), 3.03 (t, *J* = 7.1 Hz, 2H), 2.48 – 2.44 (m, 3H), 1.97 (p, *J* = 7.0 Hz, 2H), 1.45 (s, 9H).

(*R*)-2-(2-Methylsulfanylphenyl)pyrrolidine ((*R*)-26)

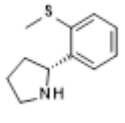

Carbamate **S4** (1.79 g, 5.79 mmol) was dissolved in a mixture of DCM (25 mL) and trifluoroacetic acid (10.0 mL, 131 mmol) and the reaction stirred at room temperature overnight. The crude reaction was basified by addition of 2 N NaOH and extracted into DCM. The organics were separated and dried using a hydrophobic fritted tube before concentration. Crude material was dissolved in 25 mL DCM and sodium triacetoxyborohydride (3.68 mg, 17.4 mmol) added in one portion. Stirring was continued for ~ 5 h after which time a further 2 eq. of sodium triacetoxyborohydride was added and the reaction stirred overnight. The reaction was partitioned between sat $NaHCO_3$ and extracted with DCM (25 mL x 3). The combined organics were dried, filtered and concentrated to give crude product which was purified by flash column chromatography on the Biotage Isolera (40 g silica, 0 to 2.5% 2 M-NH_3-MeOH/DCM) to afford racemic pyrrolidine **26** as an orange/brown gum (580 mg, 3.00 mmol, 47%).

Racemic 2-(2-methylsulfanylphenyl)pyrrolidine **26** was purified using SFC using stacked injections to separate both enantiomers. The purification methods used included analytical SFC with an AD-H Column, operating at a 5 mL/min flowrate, employing a 15% co-solvent (isopropanol + 1% diethylamine) in methanol, with injection volumes of ~10 mg/mL in methanol at 15 µL per injection. Additionally, semi-preparative SFC was performed using the same AD-H Column, but with a higher flow of 15 mL/min and a larger injection volume of 150 µL per injection, maintaining the 15% co-solvent composition. Enantiomeric purity, typically exceeding 98% enantiomeric excess (*ee*), was verified by re-analysis after purification, with the desired enantiomer (*R*)-**26** typically eluting as the second peak. HRMS (ESI) $[M+H]^+$ calculated for $C_{11}H_{15}NS$ 194.1002; found 194.1002 $[M + H]^+$. ^1H NMR (400 MHz, $CDCl_3$) δ 7.56 – 7.49 (m, 1H), 7.25 – 7.21 (m, 2H), 7.20 – 7.13 (m, 1H), 4.60 (t, *J* = 7.6 Hz, 1H), 3.24 (ddd, *J* = 10.1, 7.6, 5.5 Hz, 1H), 3.09 (ddd, *J* = 10.1, 8.1, 6.7 Hz, 1H), 2.47 (s, 3H), 2.29 (dtd, *J* = 12.5, 7.8, 5.2 Hz, 1H), 2.03 – 1.80 (m, 2H), 1.65 (ddt, *J* = 12.5, 8.9, 7.4 Hz, 1H).

2-(2-Bromophenyl)-1-pyrroline (S5)

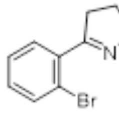

NaH (0.23 g, 9.6 mmol, 2.2 eq) was suspended in anhydrous THF (7.0 mL, 0.6 M) and cooled to 5°C with an ice-water bath. To this cold suspension was added 1-vinyl pyrrolidin-2-one (0.5 mL, 4.8 mmol, 1.1 eq) over approximately 5 min, maintaining an internal temperature of less than 5°C. Then ethyl 2-bromobenzoate (0.7 mL, 4.4 mmol, 1.0 eq) was added. After the addition was complete, the reaction mixture was stirred at 5°C for 10 min (from clear colorless becomes foggy and milky). The suspension was then heated to reflux for 1 h and allowed to cool to room temperature (upon cooling it becomes solid). To the suspension was added HCl (6.0 mL, 5N aq) dropwise, maintaining the reaction temperature between 25°C and 35°C (becomes liquid again). THF was then removed by vacuum distillation at 60°C. The resulting solution was cooled, further HCl (6.0 mL, 5N aq) was added, and the mixture was heated to reflux for 18 h (solution turns from milky white to yellow). The solution was then cooled with a salt-ice bath to 5°C and basified by careful addition of NaOH pellets (~25 g) maintaining an internal temperature between 10 and 15°C. When the reaction mixture was at pH 13, the cooling bath was removed, and temperature was allowed to reach rt. The reaction was then extracted with DCM (3 x 10 mL). The combined DCM extracts were dried with $MgSO_4$, filtered and evaporated to dryness. The crude material was then purified through flash column chromatography (isocratic elution, 2% MeOH in DCM) to afford pyrroline **S5** as a yellow oil (88 mg, 0.40 mmol, 9%). HRMS (ESI) $[M+H]^+$ found 224.0047, $C_{11}H_{11}{}^{79}Br$ requires 224.0044; found 226.0043, $C_{11}H_{11}{}^{81}Br$ requires 226.0046. ^1H NMR (400 MHz, $CDCl_3$) δ 7.60 (1H, dd, *J* = 8.0, 1.2 Hz, Ar*H*), 7.45 (1H, dd, *J* = 7.7, 1.8 Hz, Ar*H*), 7.34 (1H, td, *J* = 7.5, 1.2 Hz, Ar*H*), 7.23 (1H, td, *J* = 7.7, 1.8 Hz, Ar*H*), 4.06 (2H, tt, *J* = 7.4, 2.1 Hz, NCH_2), 3.01 (2H, tt, *J* = 8.3, 2.1 Hz, CCH_2), 2.16 – 1.95 (2H, m, $CH_2CH_2CH_2$).

2-(2-Bromophenyl)pyrrolidine (27)

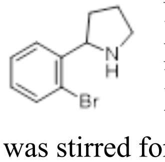

$NaBH_4$ (14.0 mg, 0.360 mmol, 2.00 eq) was added in portions to a mixture of pyrroline **S5** (40.0 mg, 0.18 mmol, 1.00 eq) in anhydrous EtOH (2.00 mL, 0.10 M). The reaction was stirred for 3 h, cooled with an ice bath, and then acidified to pH 1 by addition of HCl (3N aq). The ice bath was removed, and the mixture was stirred for 45 min. The mixture was then basified to pH 11-12 with solution of NaOH (3N aq) and extracted with Et_2O (2 x 15 mL). The organic extracts were combined and washed with H_2O (15 mL) and brine (15 mL). The solution was then dried with $MgSO_4$, filtered and evaporated *in vacuo* to afford racemic pyrrolidine **27** as a pale yellow viscous oil (44.0 mg, 0.18 mmol, 100%). HRMS (ESI) $[M+H]^+$ found 226.0226, $C_{10}H_{13}N^{79}Br$ requires 226.0231. ^1H NMR (400 MHz, $CDCl_3$) δ 7.65 – 7.57 (1H, m, Ar*H*), 7.56 – 7.50 (1H, m, Ar*H*), 7.44 – 7.31 (1H, m, Ar*H*), 7.21 – 7.09 (1H, m, Ar*H*), 5.74 – 5.20 (1H, m, C*H*), 3.61 – 3.46 (1H, m, 0.5CH_2), 3.45 – 3.26 (1H, m, 0.5CH_2), 2.83 – 2.75 (1H, m, 0.5CH_2), 2.41 – 2.30 (1H, m, 0.5CH_2), 2.15 – 1.97 (2H, m, CH_2).

(*R*)-2-(2-Bromophenyl)pyrrolidine **(*R*)-27** was purchased from BLD as a single enantiomer.

1.4 Preparation of Hydantoin 29

2-{[(4-Nitrophenyl)methyl]amino}acetonitrile (22af)

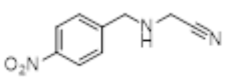

4-Nitrobenzylamine hydrochloride (**20a**) (10.0 g, 53.2 mmol) was reacted with chloroacetonitrile (**21f**) (3.36mL, 53.2 mmol, 1.00 eq) according to General Procedure A to afford the amine **22af** as a brown oil (6.71 g, 35.1 mmol, 66.0%). HRMS (ESI) $[M+H]^+$ found 191.1915, $C_9H_{10}N_3O_2$ requires 191.1903. ^1H NMR (400 MHz, $CDCl_3$) δ 8.21 (2H, d, *J* = 8.7 Hz), 7.55 (2H, d, *J* = 8.7 Hz), 4.05 (2H, s), 3.60 (2H, s), 1.71 (1H, br. s). ^{13}C NMR (126 MHz, $CDCl_3$) δ 147.6, 145.3, 128.9, 123.8, 117.1, 51.5, 36.5.

Ethyl 2-{[(cyanomethyl)[(4-nitrophenyl)methyl]carbamoyl]amino}acetate (23af)

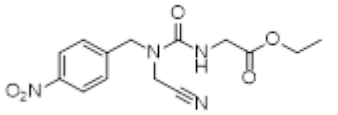

Amine **22af** (0.90 g, 4.8 mmol) was reacted according to General Procedure B to afford urea **23af** as a brown oil (1.3 g, 4.3 mmol, 90%). HRMS (ESI) $[M+H]^+$ found 321.1230, $C_{14}H_{17}N_4O_5$ requires 321.1225. ^1H NMR (400 MHz, $CDCl_3$) δ 8.25 (2H, d, *J* = 8.7 Hz), 7.50 (2H, d, *J* = 8.7 Hz), 5.19 (1H, t, *J* = 5.1 Hz), 4.68 (2H, s), 4.32 (2H, s), 4.27 (2H, q, *J* = 7.2 Hz), 3.99 (2H, d, *J* = 5.1 Hz), 1.31 (3H, t, *J* = 7.2 Hz). ^{13}C NMR (126 MHz, $CDCl_3$) δ 170.4, 156.5, 147.9, 142.4, 127.7, 124.4, 115.4, 61.7, 50.8, 42.9, 35.8, 14.1.

1-(4-nitrobenzyl)-2,4-dioxoimidazolidine-3-acetic acid (29)

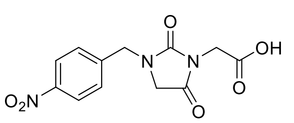

Urea ester **23af** (50.0 mg, 0.16 mmol) was reacted according to General Procedure C. Upon completion of the reaction, the organic solvents were removed *in vacuo*, and the basic aqueous solution was washed with EtOAc to remove any impurities. The aqueous solution was acidified to pH 3 using HCl (3 M aq), washed with EtOAc (×3), then left at rt for 24 h. The aqueous layer was concentrated *in vacuo* to give hydantoin **29** (43 mg, 93 %) as a yellow gum. HRMS (ESI) $[M + H]^+$ found 294.0947 $C_{12}H_{11}N_3O_6$ requires 294.0726. ^1H NMR (600 MHz, MeOD) δ 8.20 (2H, d, *J* = 8.7 Hz), 7.55 (2H, d, *J* = 8.7 Hz), 4.72 (2H, s), 4.22 (2H, s), 4.01 (2H, s). ^{13}C NMR (126 MHz, MeOD) δ 170.2, 169.3, 156.8, 147.6, 143.7, 128.5, 123.6, 49.6, 45.5, 39.2.

Analytical data for compounds 1-19

<table>
<tr><th>#</th><th>Chromatograms at 254 nm / Total absorbance (left) and 220/214 nm (right)</th></tr>
<tr><td>2</td><td>
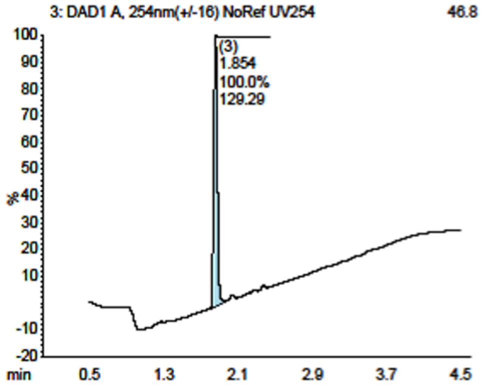

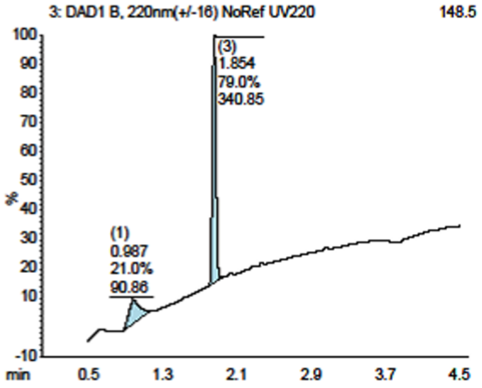

</td></tr>
<tr><td>3</td><td>
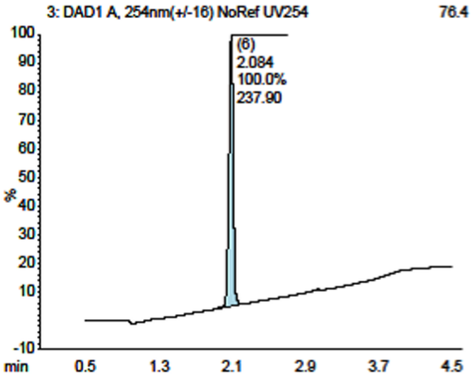

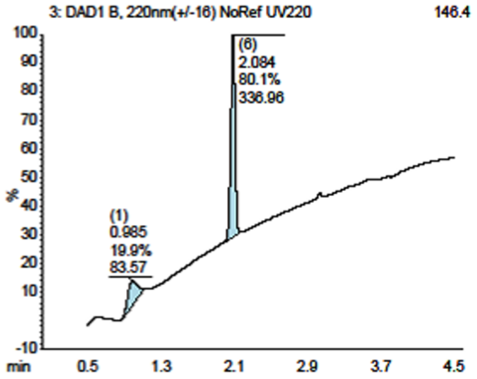

</td></tr>
<tr><td>4</td><td>
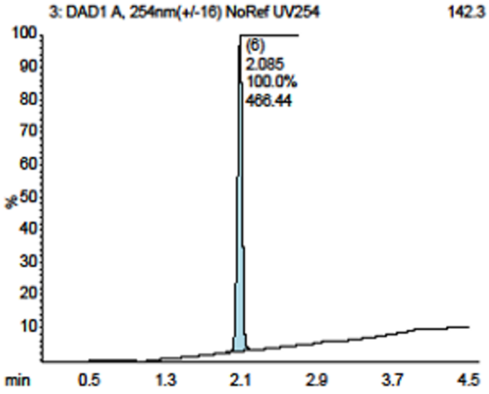

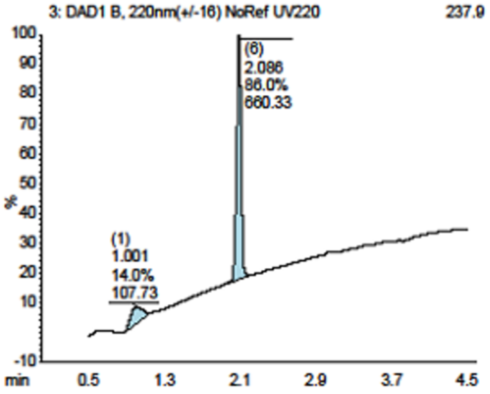

</td></tr>
</table>

**5**	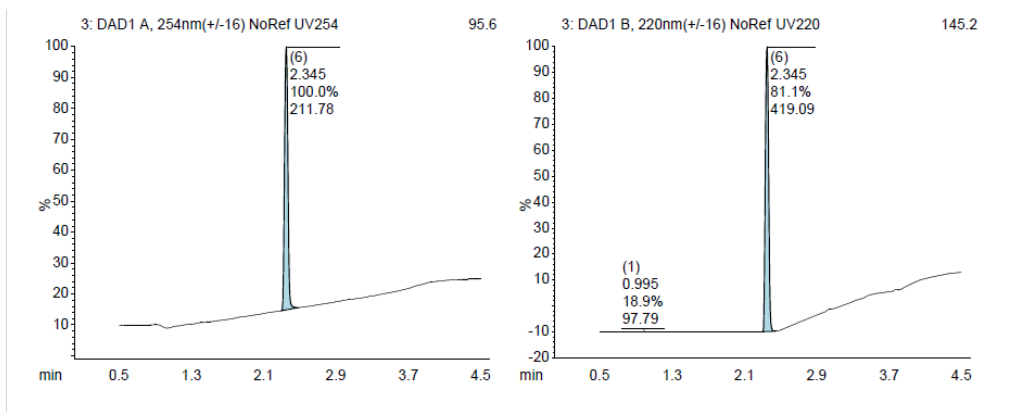3: DAD1 A, 254nm(+/-16) NoRef UV254<br>95.6<br>(6)<br>2.345<br>100.0%<br>211.78<br>3: DAD1 B, 220nm(+/-16) NoRef UV220<br>145.2<br>(6)<br>2.345<br>81.1%<br>419.09<br>(1)<br>0.995<br>18.9%<br>97.79<br>%<br>min
**6**	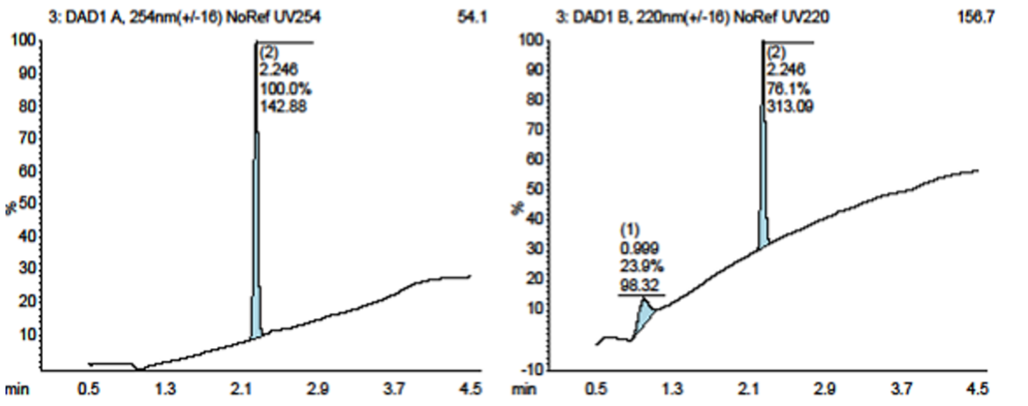3: DAD1 A, 254nm(+/-16) NoRef UV254<br>54.1<br>(2)<br>2.246<br>100.0%<br>142.88<br>3: DAD1 B, 220nm(+/-16) NoRef UV220<br>156.7<br>(2)<br>2.246<br>76.1%<br>313.09<br>(1)<br>0.999<br>23.9%<br>98.32<br>%<br>min
**7**	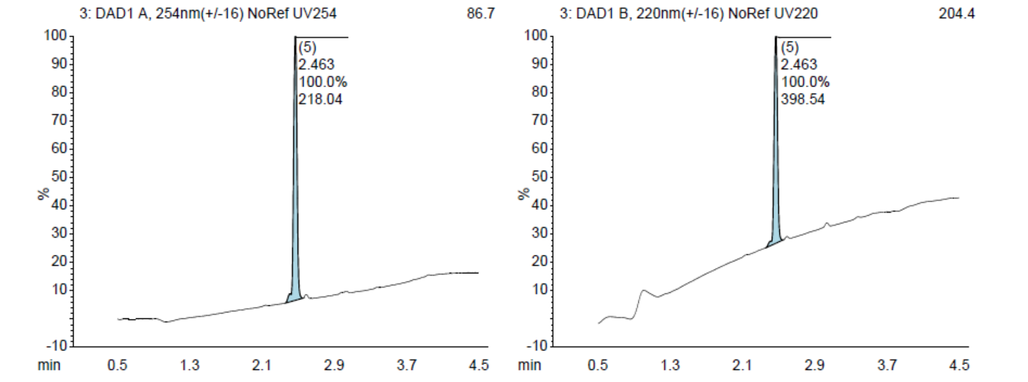3: DAD1 A, 254nm(+/-16) NoRef UV254<br>86.7<br>(5)<br>2.463<br>100.0%<br>218.04<br>3: DAD1 B, 220nm(+/-16) NoRef UV220<br>204.4<br>(5)<br>2.463<br>100.0%<br>398.54<br>%<br>min
**8**	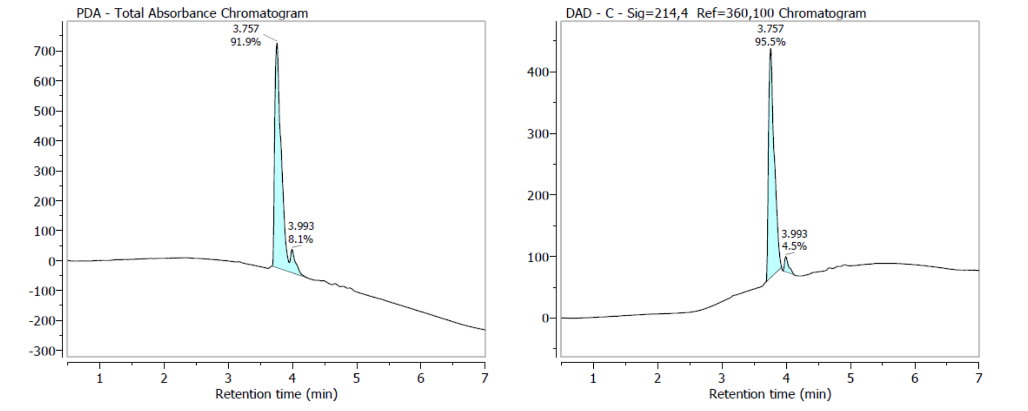PDA - Total Absorbance Chromatogram<br>3.757<br>91.9%<br>3.993<br>8.1%<br>Retention time (min)<br>DAD - C - Sig=214,4 Ref=360,100 Chromatogram<br>3.757<br>95.5%<br>3.993<br>4.5%<br>Retention time (min)

**9**	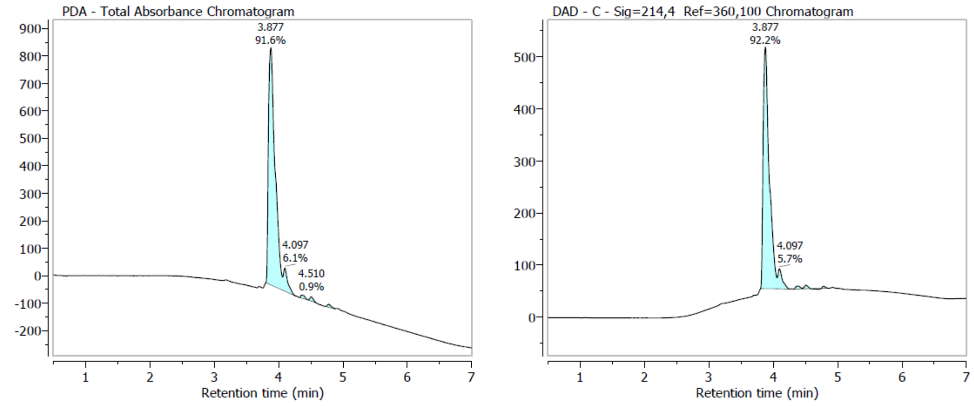
**10**	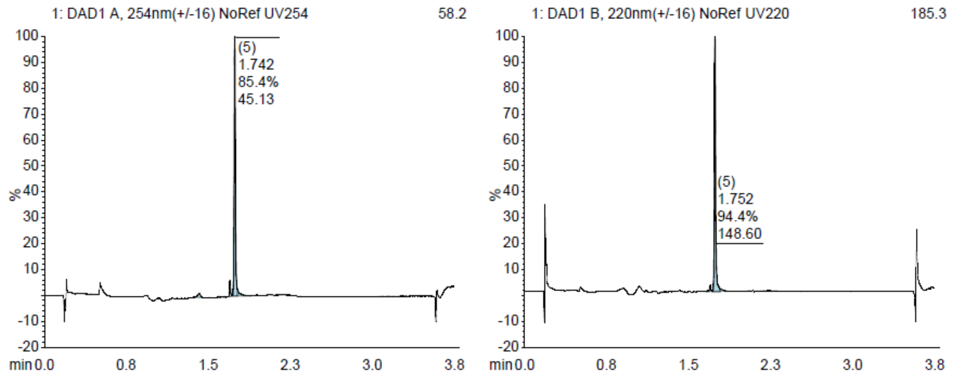
**11**	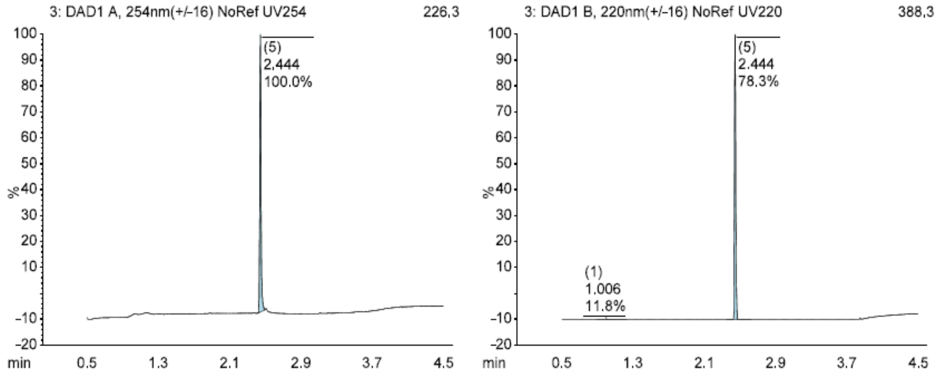
**12**	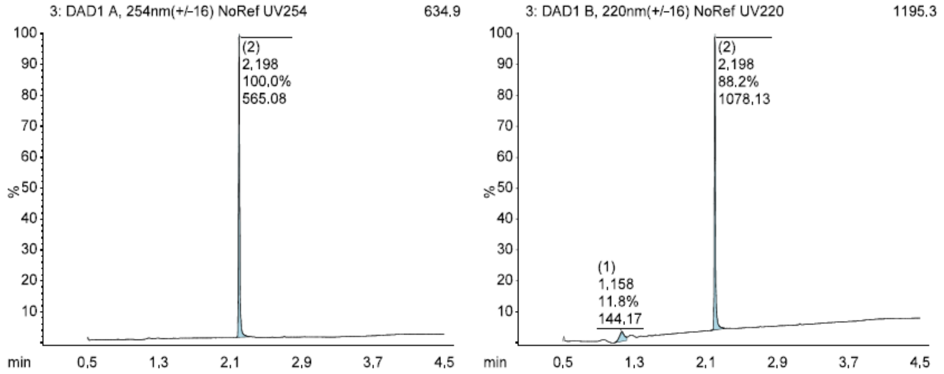

PDA - Total Absorbance Chromatogram
3.877
91.6%
4.097
6.1%
4.510
0.9%
Retention time (min)
DAD - C - Sig=214,4 Ref=360,100 Chromatogram
3.877
92.2%
4.097
5.7%
Retention time (min)

1: DAD1 A, 254nm(+/-16) NoRef UV254
58.2
(5)
1.742
85.4%
45.13
1: DAD1 B, 220nm(+/-16) NoRef UV220
185.3
(5)
1.752
94.4%
148.60
%
min

3: DAD1 A, 254nm(+/-16) NoRef UV254
226,3
(5)
2,444
100.0%
3: DAD1 B, 220nm(+/-16) NoRef UV220
388,3
(1)
1,006
11.8%
(5)
2,444
78.3%
%
min

3: DAD1 A, 254nm(+/-16) NoRef UV254
634,9
(2)
2,198
100,0%
565,08
3: DAD1 B, 220nm(+/-16) NoRef UV220
1195,3
(1)
1,158
11,8%
144,17
(2)
2,198
88,2%
1078,13
%
min

13

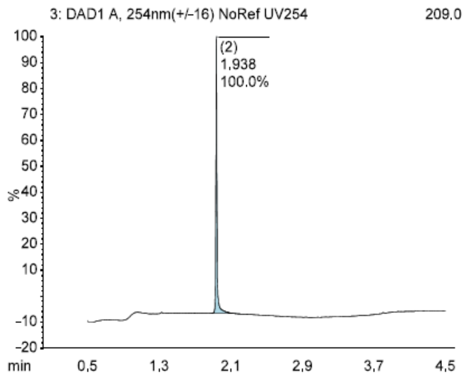
3: DAD1 A, 254nm(+/-16) NoRef UV254
209.0
(2)
1,938
100.0%
%
min

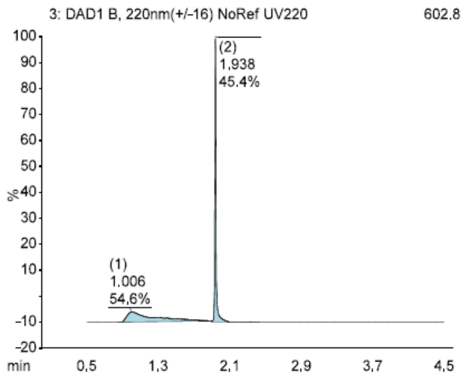
3: DAD1 B, 220nm(+/-16) NoRef UV220
602.8
(2)
1,938
45.4%
(1)
1,006
54,6%
%
min

14

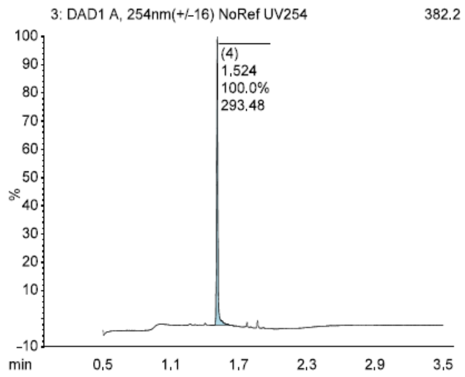
3: DAD1 A, 254nm(+/-16) NoRef UV254
382.2
(4)
1,524
100.0%
293.48
%
min

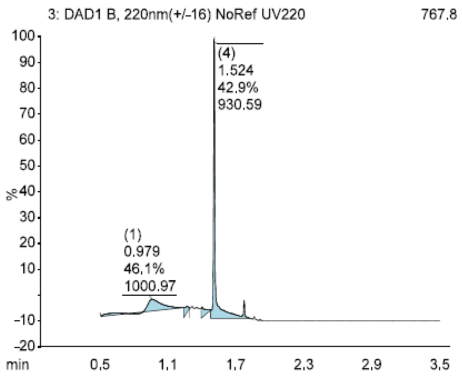
3: DAD1 B, 220nm(+/-16) NoRef UV220
767.8
(4)
1.524
42.9%
930.59
(1)
0,979
46,1%
1000.97
%
min

15

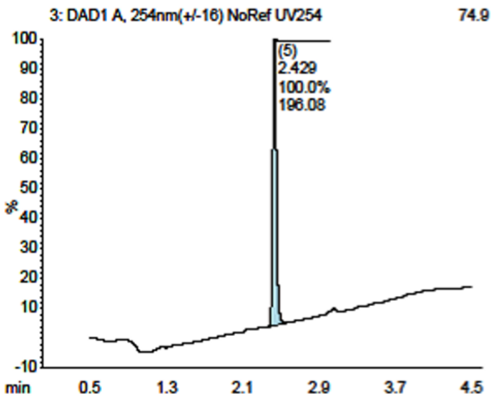
3: DAD1 A, 254nm(+/-16) NoRef UV254
74.9
(5)
2.429
100.0%
196.08
%
min

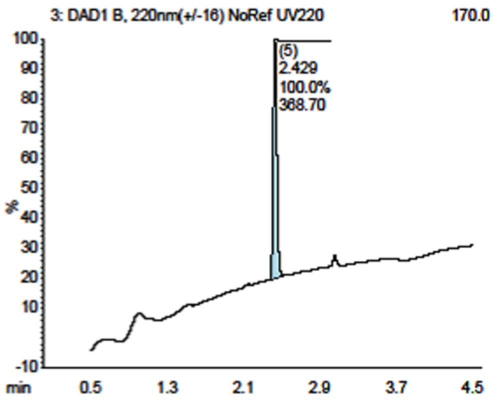
3: DAD1 B, 220nm(+/-16) NoRef UV220
170.0
(5)
2.429
100.0%
368.70
%
min

16

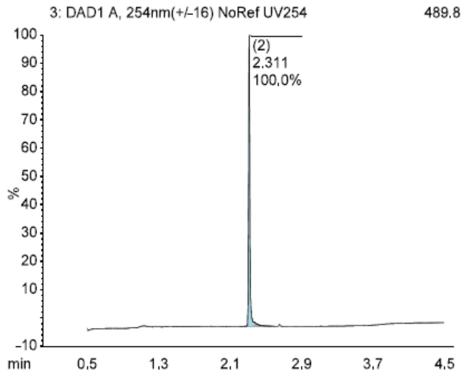
3: DAD1 A, 254nm(+/-16) NoRef UV254
489.8
(2)
2.311
100,0%
%
min

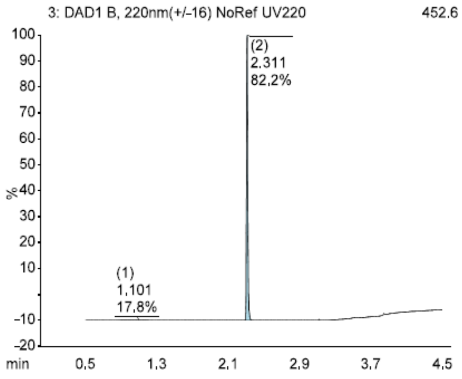
3: DAD1 B, 220nm(+/-16) NoRef UV220
452.6
(2)
2.311
82,2%
(1)
1,101
17,8%
%
min

17

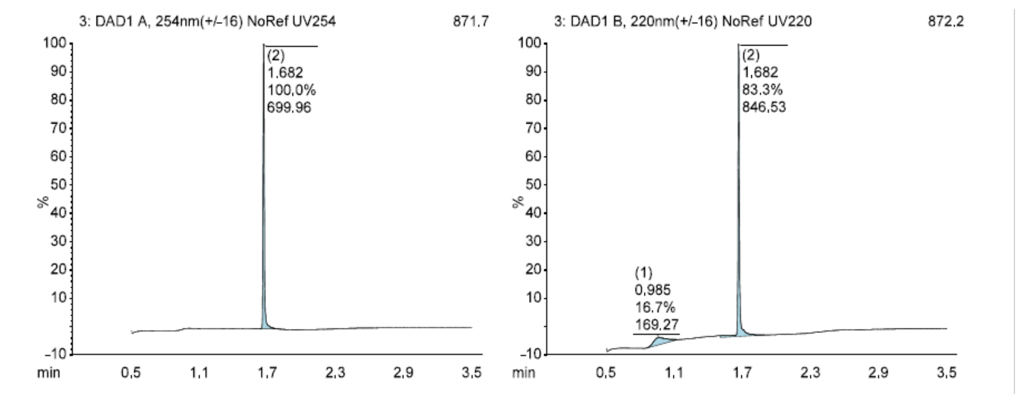
3: DAD1 A, 254nm(+/-16) NoRef UV254
871.7
(2)
1.682
100,0%
699.96
3: DAD1 B, 220nm(+/-16) NoRef UV220
872.2
(1)
0,985
16.7%
169.27
(2)
1,682
83.3%
846,53
%
min
0,5
1,1
1,7
2,3
2,9
3,5

18

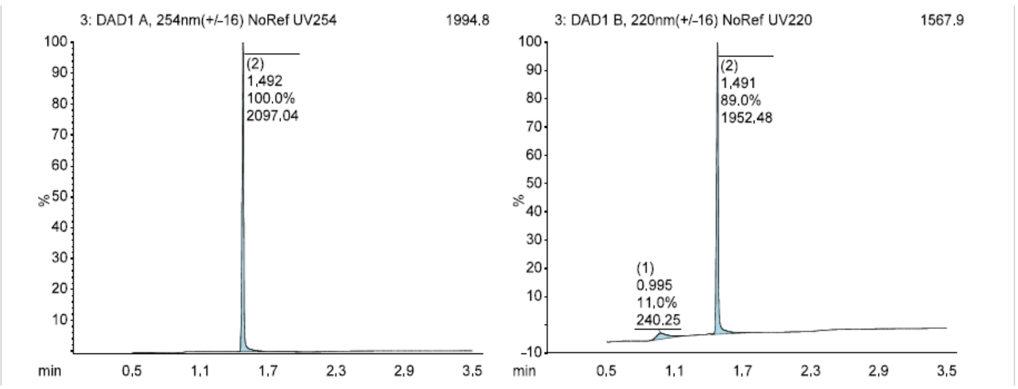
3: DAD1 A, 254nm(+/-16) NoRef UV254
1994.8
(2)
1,492
100.0%
2097,04
3: DAD1 B, 220nm(+/-16) NoRef UV220
1567.9
(1)
0,995
11,0%
240.25
(2)
1,491
89,0%
1952,48
%
min
0,5
1,1
1,7
2,3
2,9
3,5

19

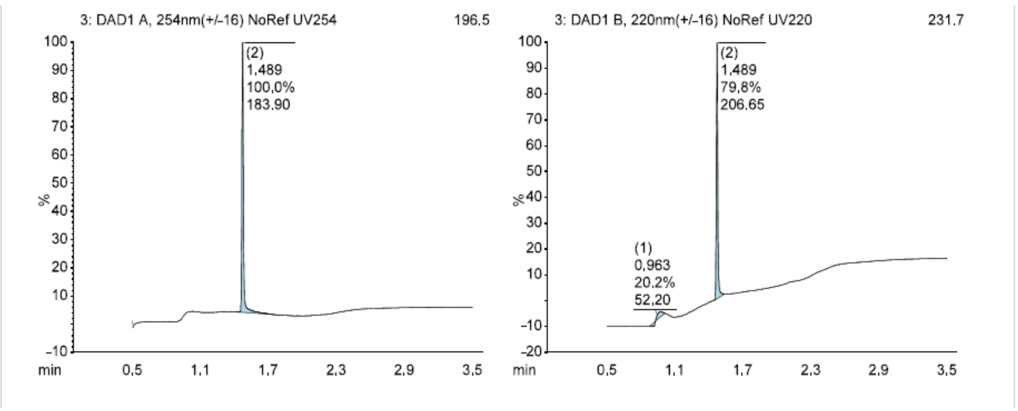
3: DAD1 A, 254nm(+/-16) NoRef UV254
196.5
(2)
1,489
100,0%
183.90
3: DAD1 B, 220nm(+/-16) NoRef UV220
231.7
(1)
0,963
20.2%
52,20
(2)
1,489
79,8%
206.65
%
min
0,5
1,1
1,7
2,3
2,9
3,5

N-[(2-Methyltetrazol-5-yl)methyl]-1-(4-nitrophenyl)methanamine (22aa)

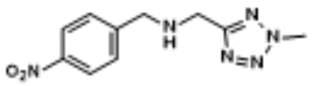

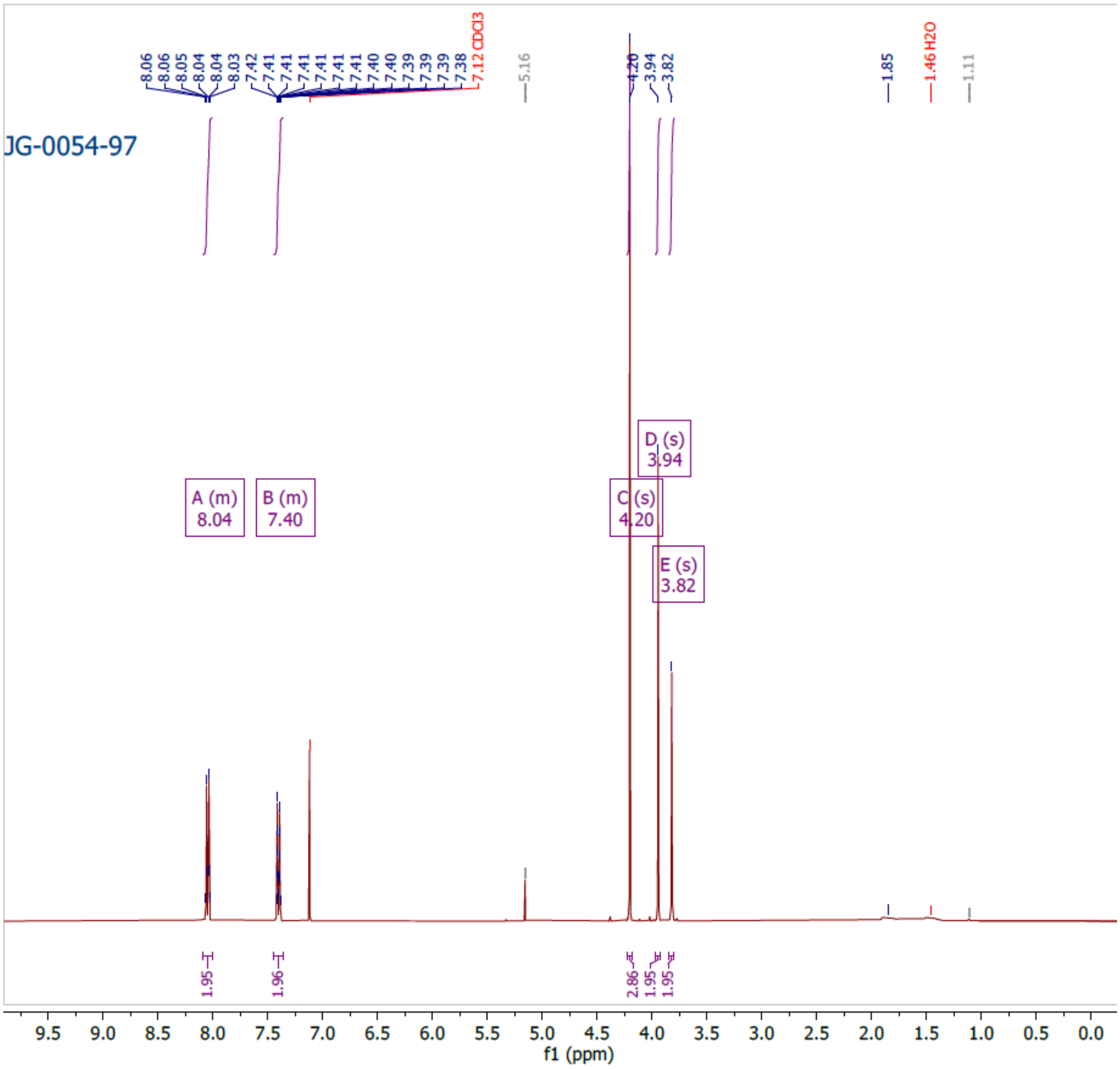

Ethyl 2-({[(2-methyl-2*H*-1,2,3,4-tetrazol-5-yl)methyl][(4-nitrophenyl)methyl] carbamoyl} amino)acetate (23aa)

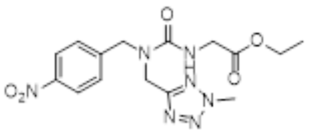

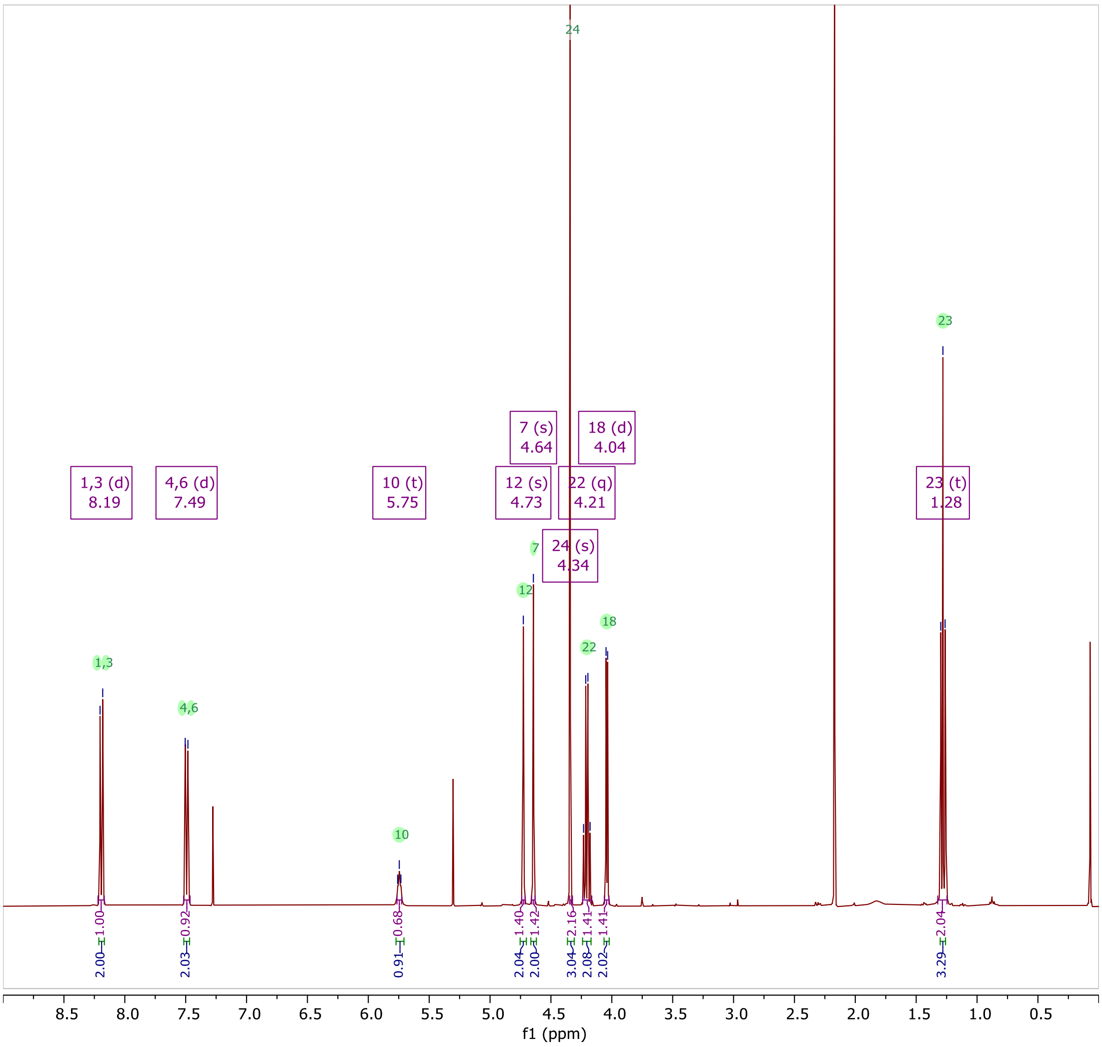

2-({[(2-Methyl-2*H*-1,2,3,4-tetrazol-5-yl)methyl][(4-aminophenyl)methyl]carbamoyl}nitro)acetic acid (24aa)

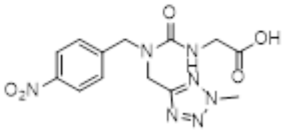

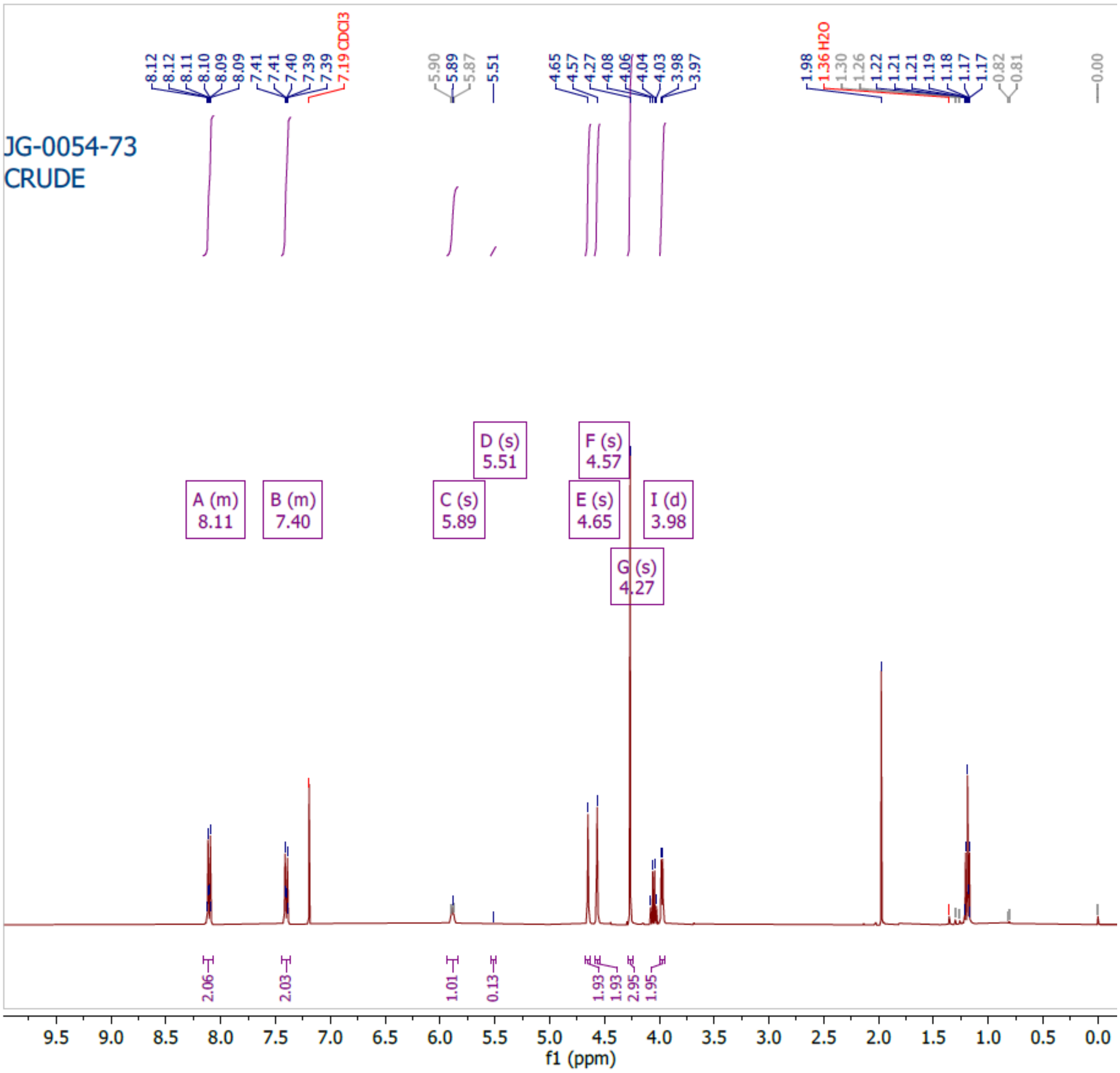

1-[(4-Aminophenyl)methyl]-1-[(2-methyltetrazol-5-yl)methyl]-3-[2-oxo-2-[(2*R*)-2-(2-methylsulfanylphenyl)pyrrolidin-1-yl]ethyl]urea (2)

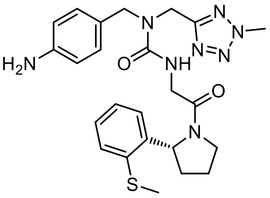

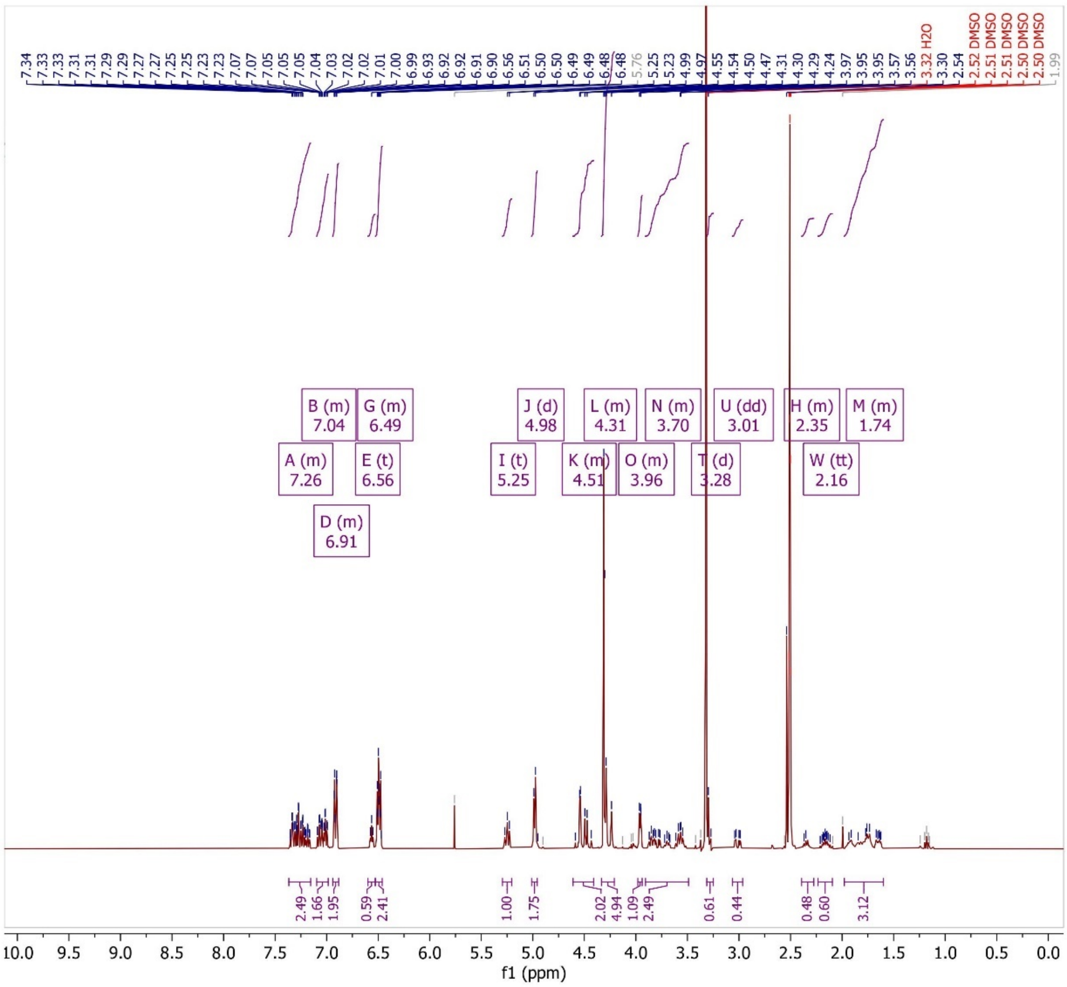

1-[(1*S*,9*R*,10*S*)-10-[*tert*-Butyl(dimethyl)silyl]oxy-12-oxa-8-azatricyclo[7.3.1.0^{2},^{7}]trideca-2,4,6-trien-4-yl]-N-[(2-methyltetrazol-5-yl)methyl]methanamine (22ba)

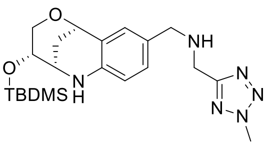

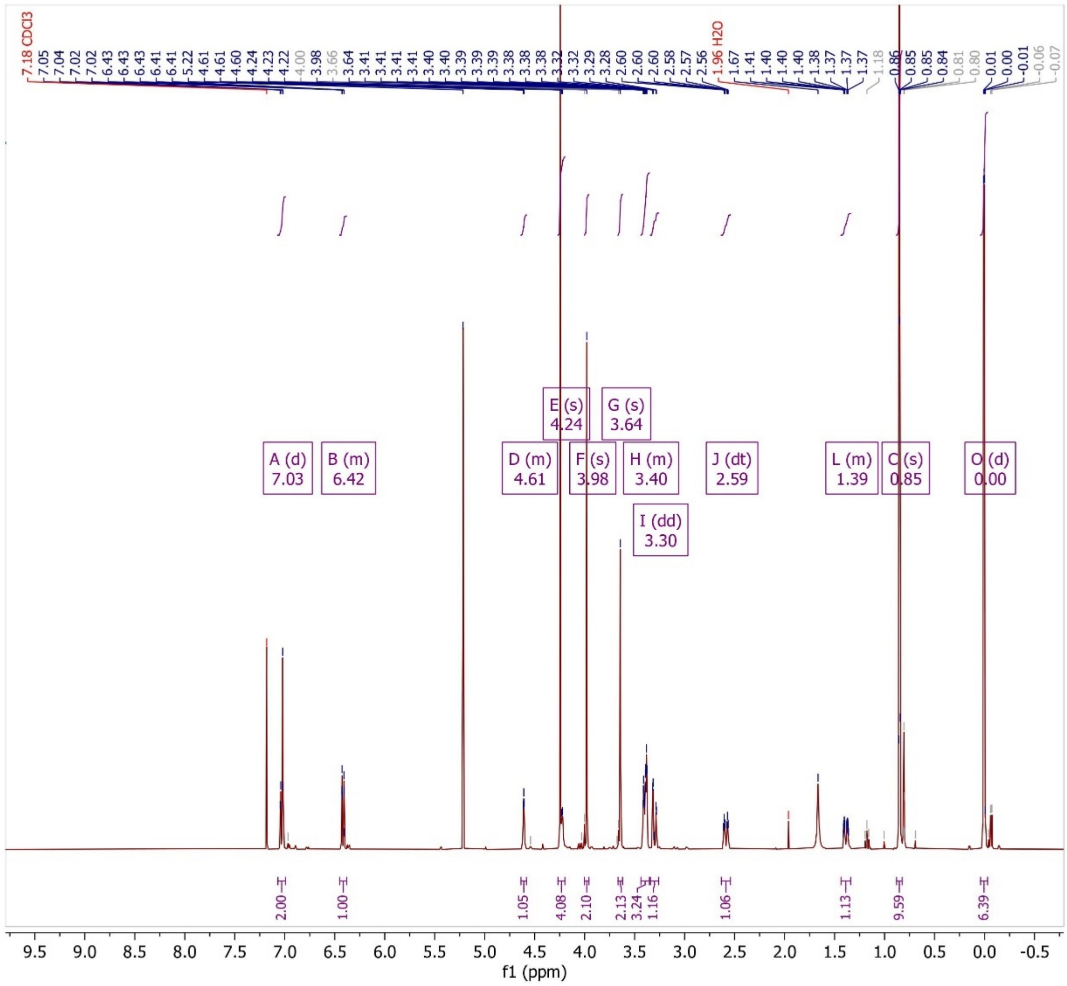

Ethyl 2-[[[(1*S*,9*R*,10*S*)-10-[*tert*-butyl(dimethyl)silyl]oxy-12-oxa-8-azatricyclo[7.3.1.0^{2},^{7}] trideca-2,4,6-trien-4-yl]methyl-[(2-methyltetrazol-5-yl)methyl]carbamoyl]amino]acetate (23ba)

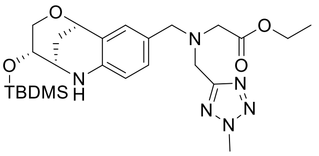

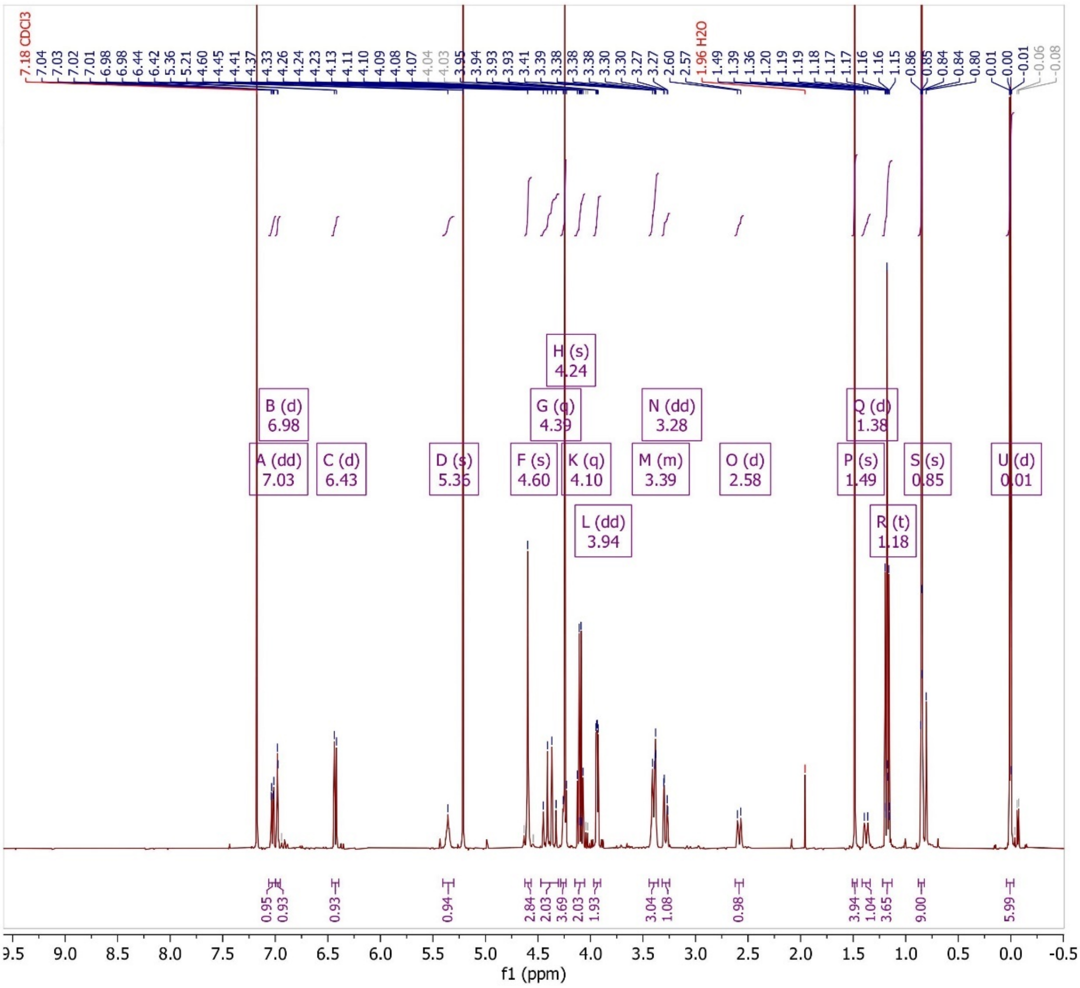

1-[[(1*S*,9*R*,10*S*)-10-[*tert*-Butyl(dimethyl)silyl]oxy-12-oxa-8-azatricyclo[7.3.1.0^{2},^{7}] trideca-2,4,6-trien-4-yl]methyl]-3-[2-[(2*R*)-2-(2-methylsulfanylphenyl) pyrrolidin-1-yl]-2-oxo-ethyl]-1-[(2-methyltetrazol-5-yl)methyl]urea (*R*)-(28ba)

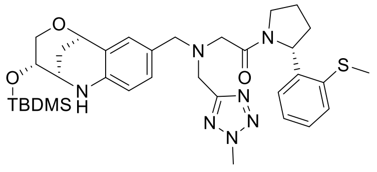

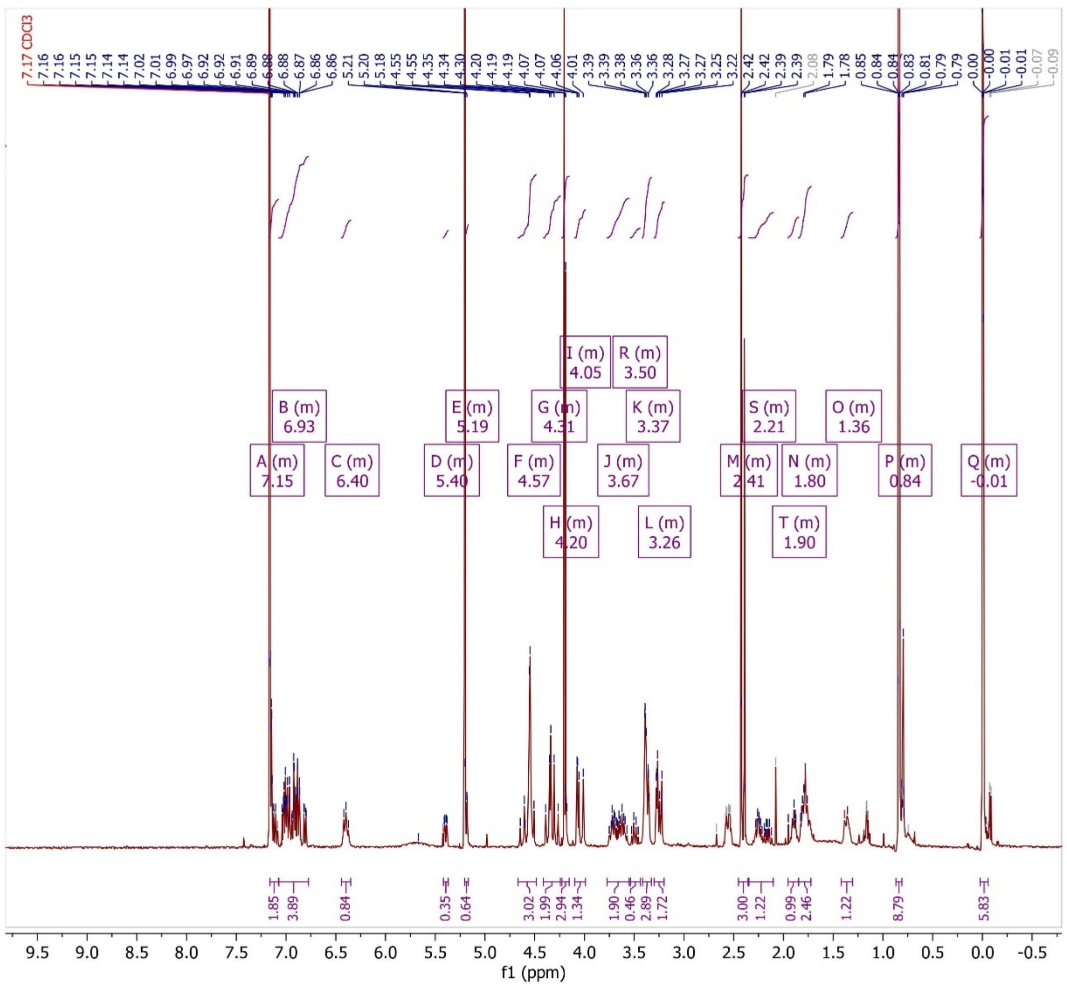

1-[[(1*S*,9*R*,10*S*)-10-Hydroxy-12-oxa-8-azatricyclo[7.3.1.0^{2,7}]trideca-2,4,6-trien-4-yl]methyl]-3-[2-[(2*R*)-2-(2-methylsulfanylphenyl)pyrrolidin-1-yl]-2-oxo-ethyl]-1-[(2-methyltetrazol-5-yl)methyl]urea (3)

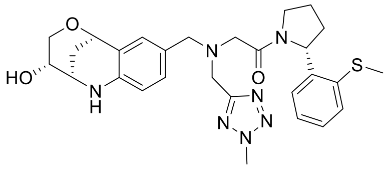

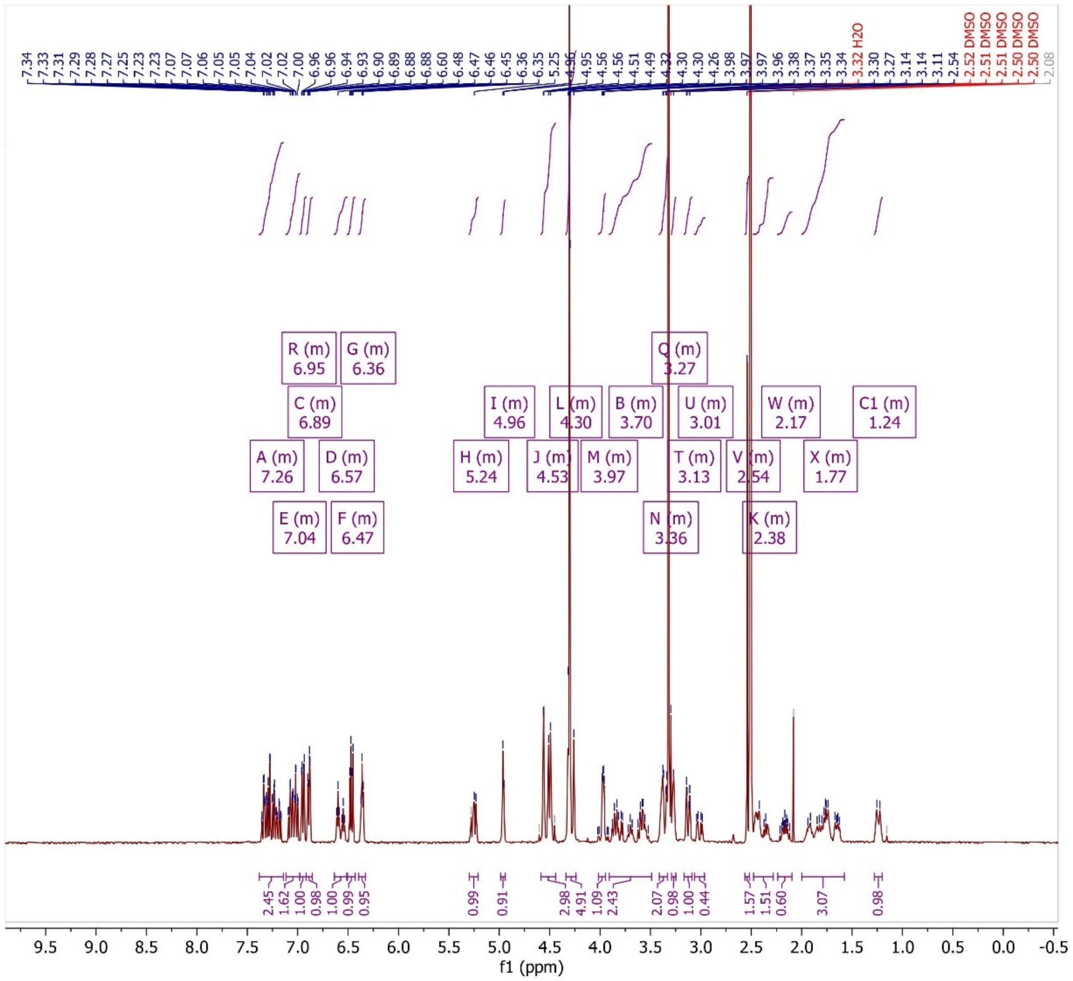

1-[[(1*S*,9*R*,10*S*)-10-Hydroxy-12-oxa-8-azatricyclo[7.3.1.0^{2},^{7}]trideca-2,4,6-trien-4-yl]methyl]-3-[2-[(2*S*)-2-(2-methylsulfanylphenyl)pyrrolidin-1-yl]-2-oxo-ethyl]-1-[(2-methyltetrazol-5-yl)methyl]urea (*S*)-(28ba)

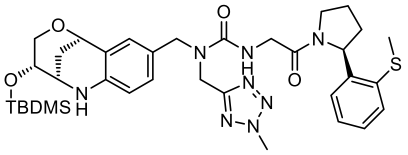

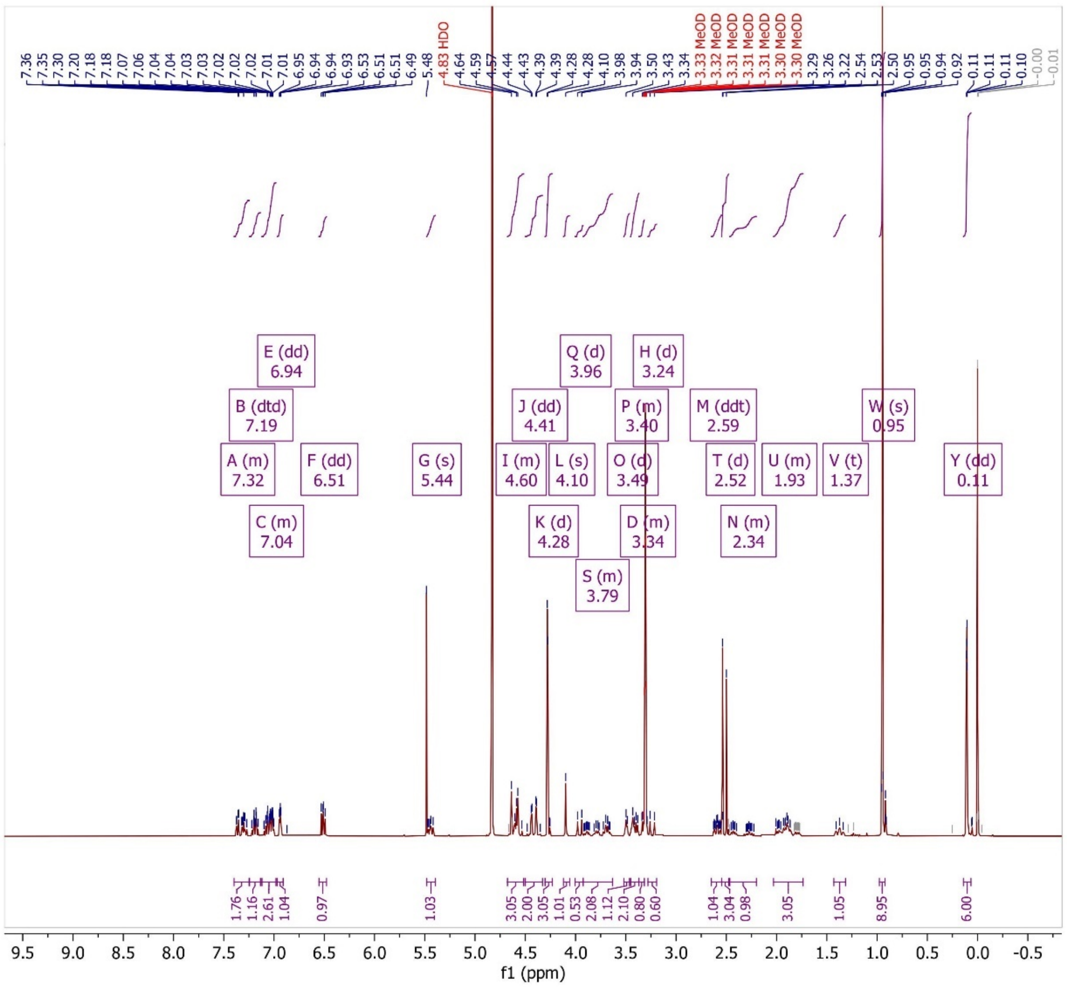

1-[[(1*S*,9*R*,10*S*)-10-Hydroxy-12-oxa-8-azatricyclo[7.3.1.0^{2},^{7}]trideca-2,4,6-trien-4-yl]methyl]-3-[2-[(2*S*)-2-(2-methylsulfanylphenyl)pyrrolidin-1-yl]-2-oxo-ethyl]-1-[(2-methyltetrazol-5-yl)methyl]urea (4)

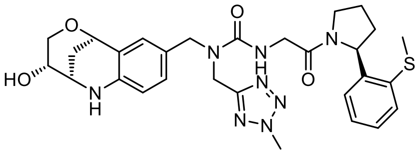

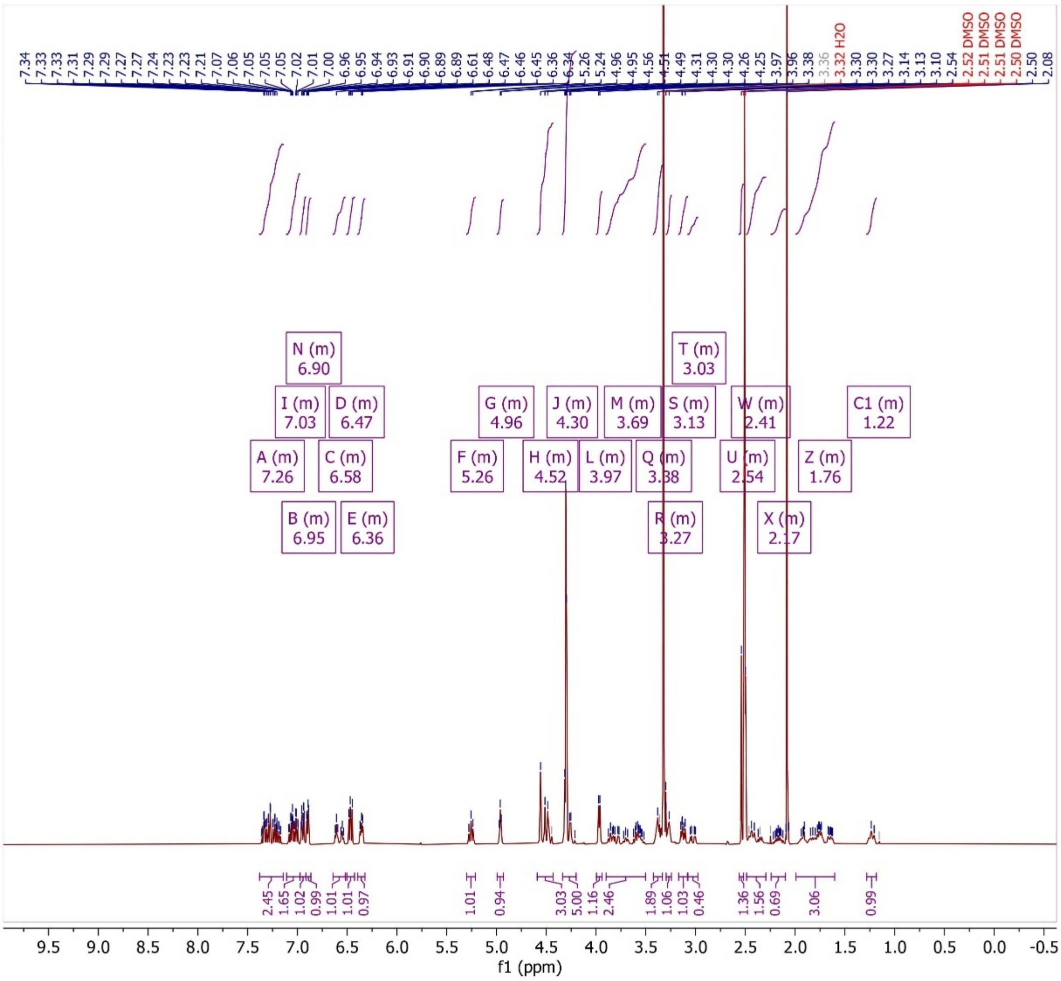

2,6-Difluoro-4-[[(2-methyltetrazol-5-yl)methylamino]methyl]aniline (22ca)

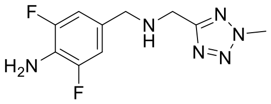

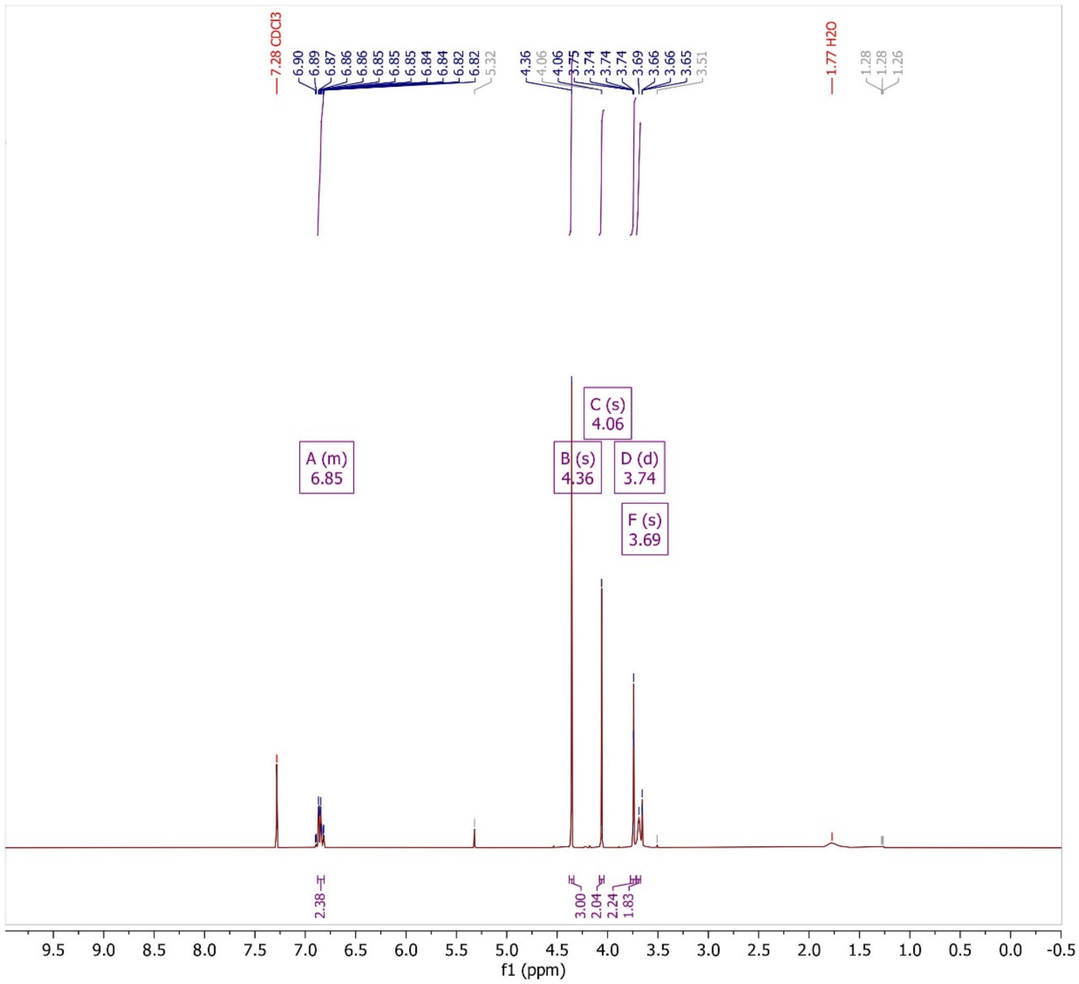

Ethyl 2-[[(4-amino-3,5-difluoro-phenyl)methyl-[(2-methyltetrazol-5-yl)methyl] carbamoyl]amino]acetate (23ca)

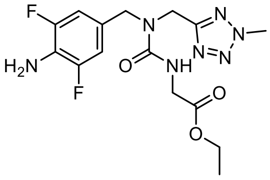

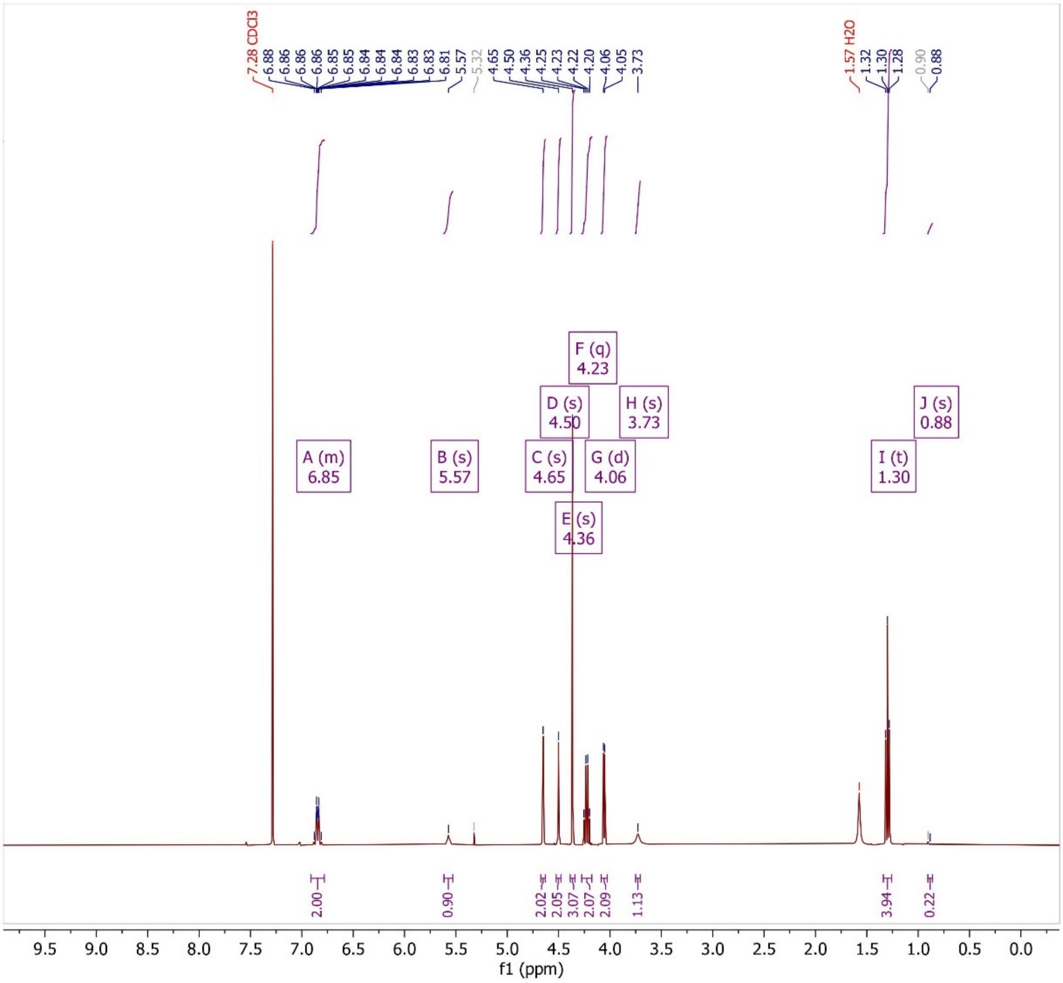

1-[(4-Amino-3,5-difluoro-phenyl)methyl]-1-[(2-methyltetrazol-5-yl)methyl]-3-[2-oxo-2-[(2*R*)-2-(2-methylsulfanylphenyl)pyrrolidin-1-yl]ethyl]urea (5)

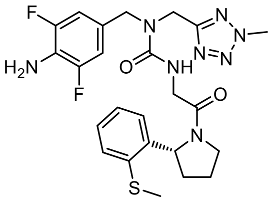

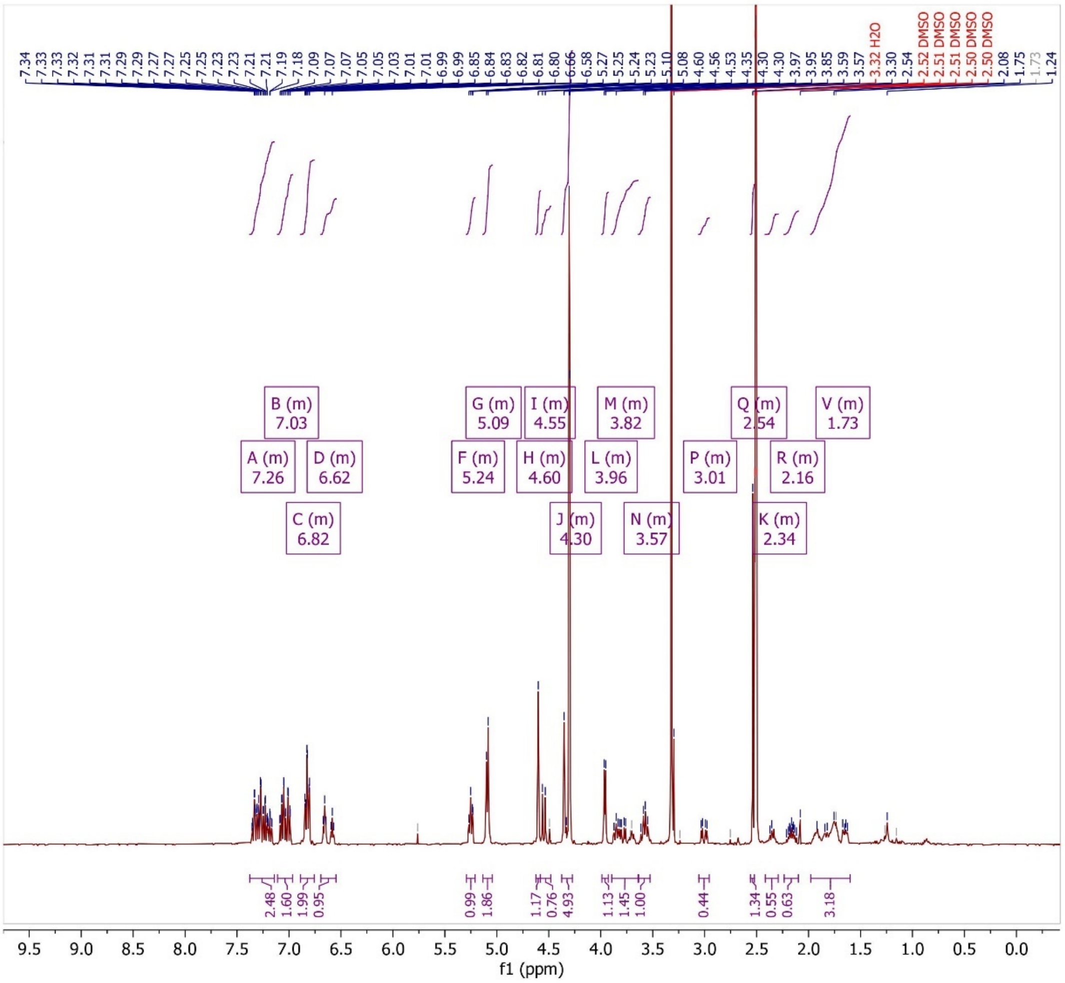

2,6-Difluoro-4-[(prop-2-ynylamino)methyl]aniline (22ce)

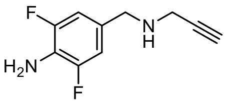

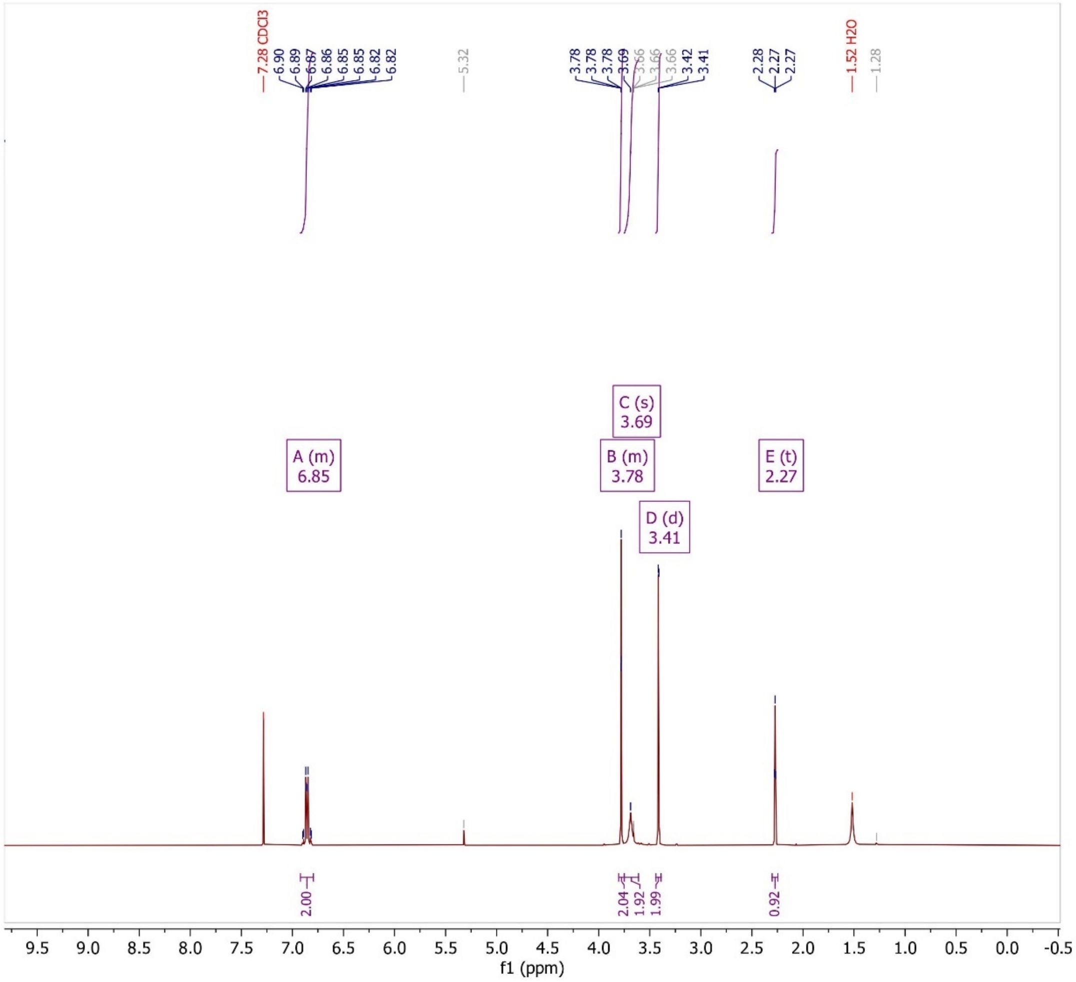

Ethyl 2-[[(4-amino-3,5-difluoro-phenyl)methyl-prop-2-ynyl-carbamoyl]amino] acetate (23ce)

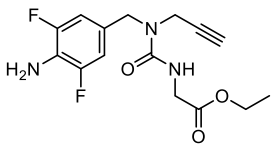

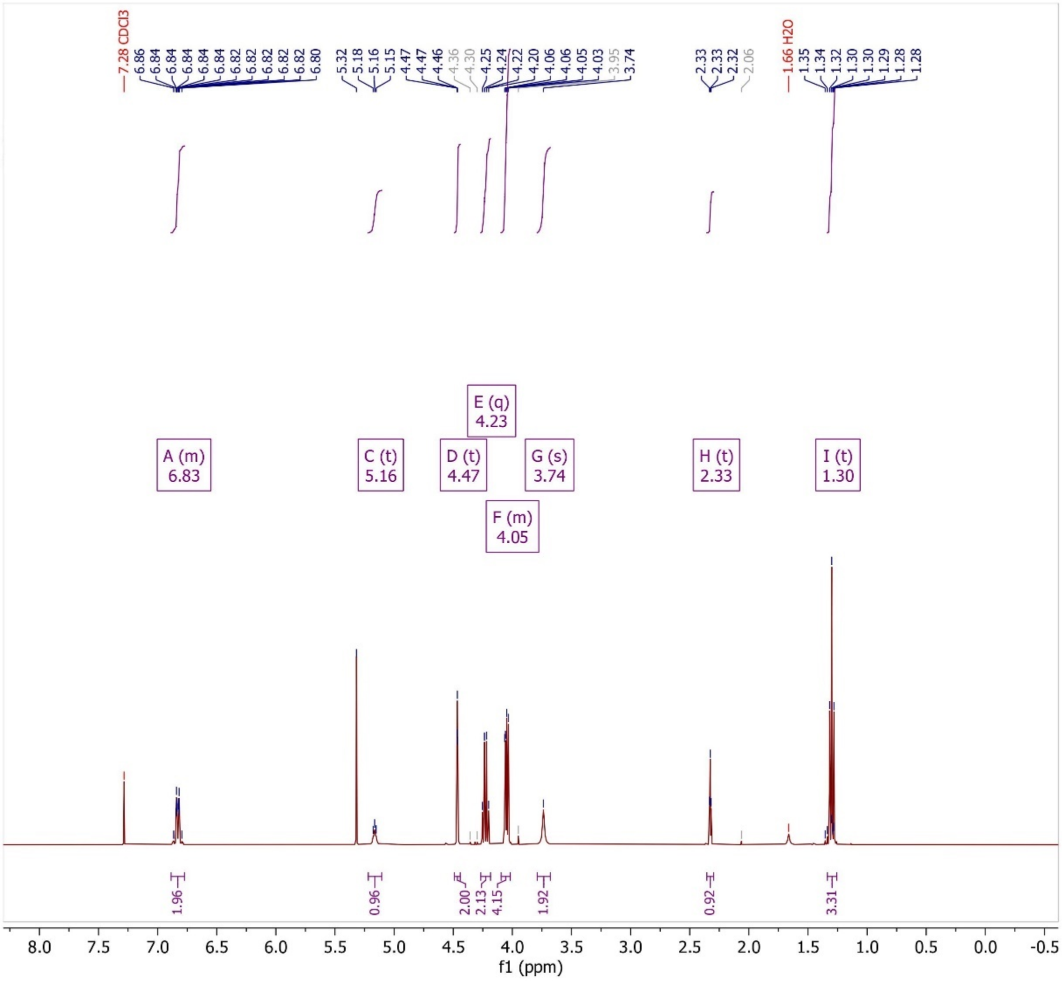

Ethyl 2-[[(4-amino-3,5-difluoro-phenyl)methyl-[(1-methyltriazol-4-yl)methyl]carbamoyl] amino]acetate (23cg)

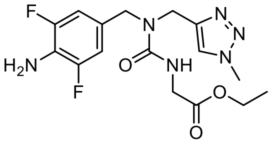

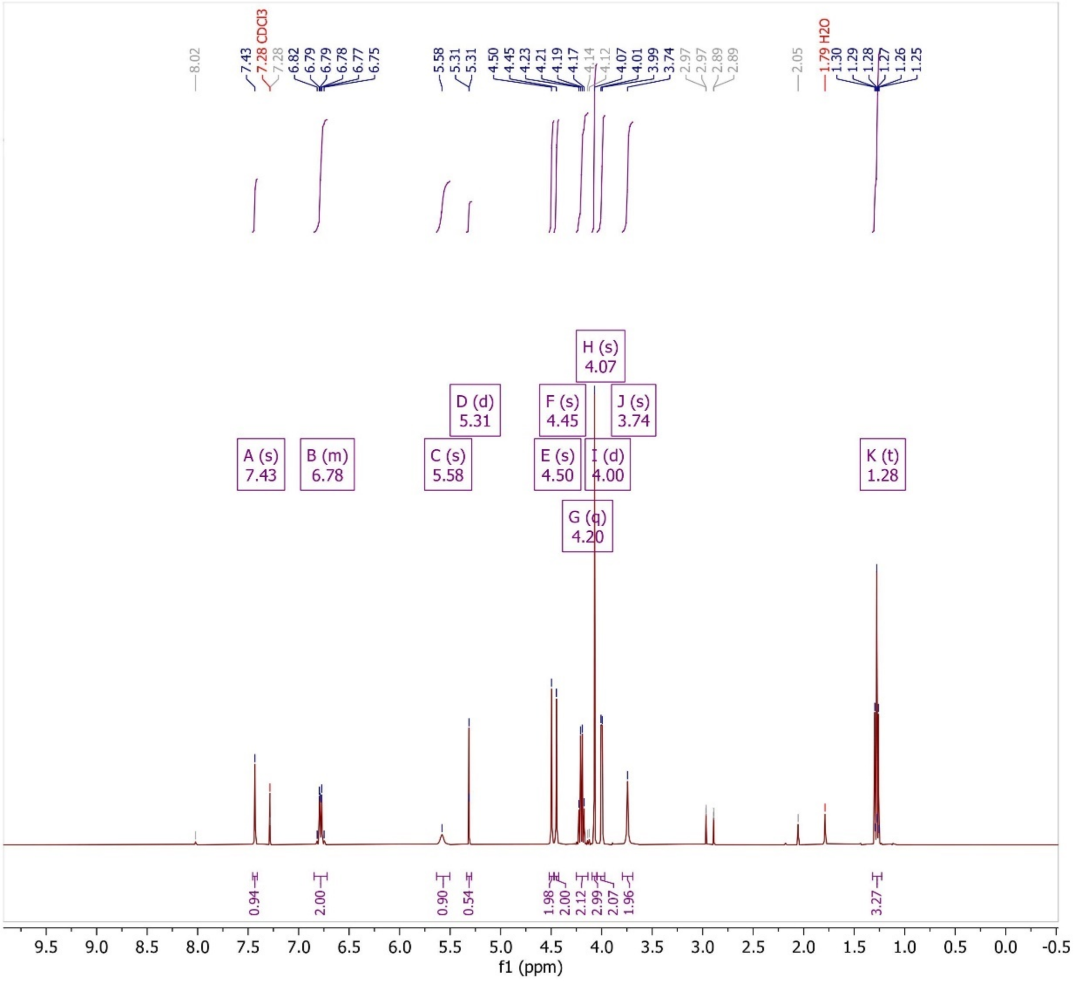

1-[(4-Amino-3,5-difluoro-phenyl)methyl]-1-[(1-methyltriazol-4-yl)methyl]-3-[2-oxo-2-[(2*R*)-2-(2-methylsulfanylphenyl)pyrrolidin-1-yl]ethyl]urea (6)

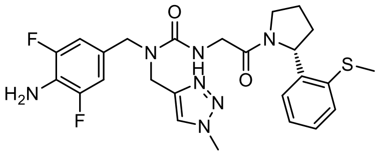

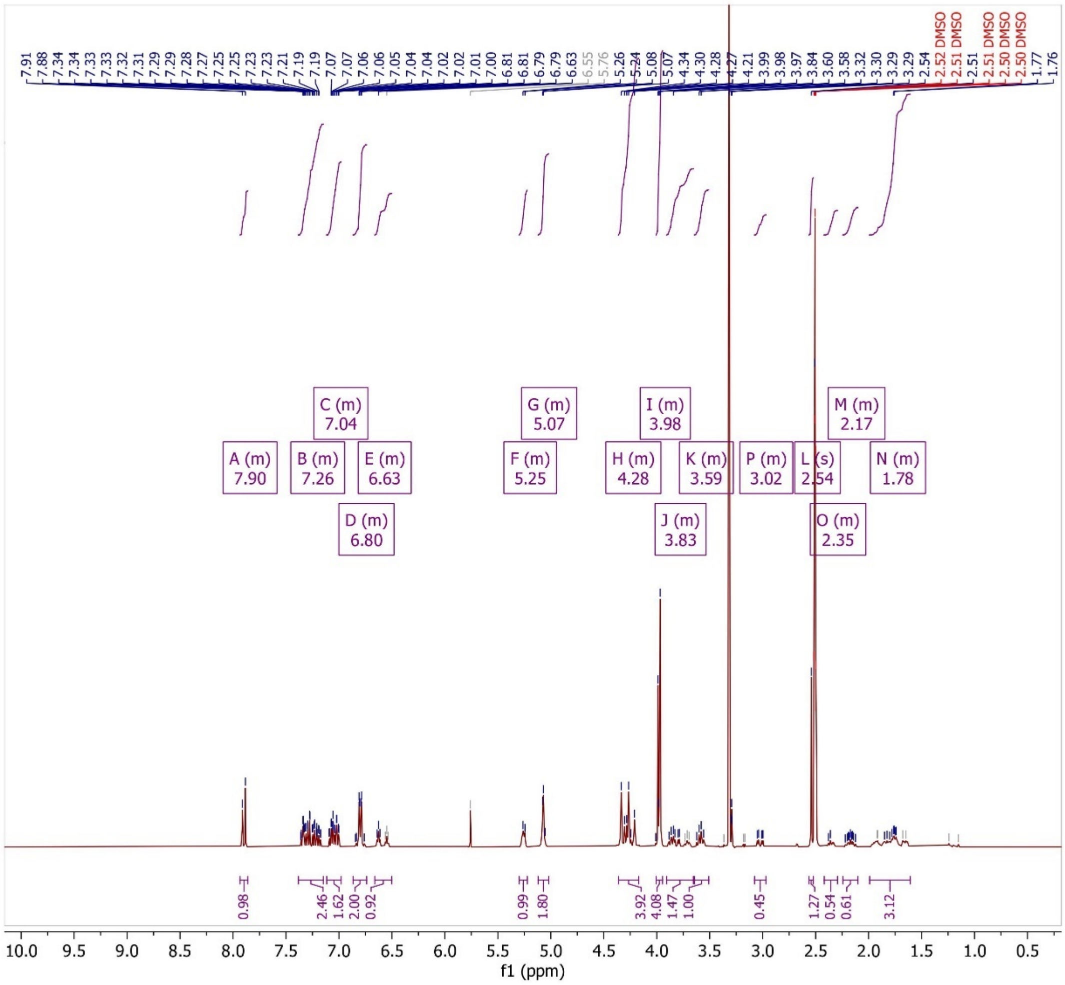

1-[(4-Amino-3,5-difluoro-phenyl)methyl]-3-[2-oxo-2-[(2*R*)-2-(2-methylsulfanyl phenyl)pyrrolidin-1-yl]ethyl]-1-prop-2-ynyl-urea (7)

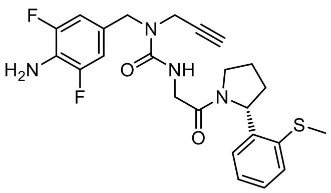

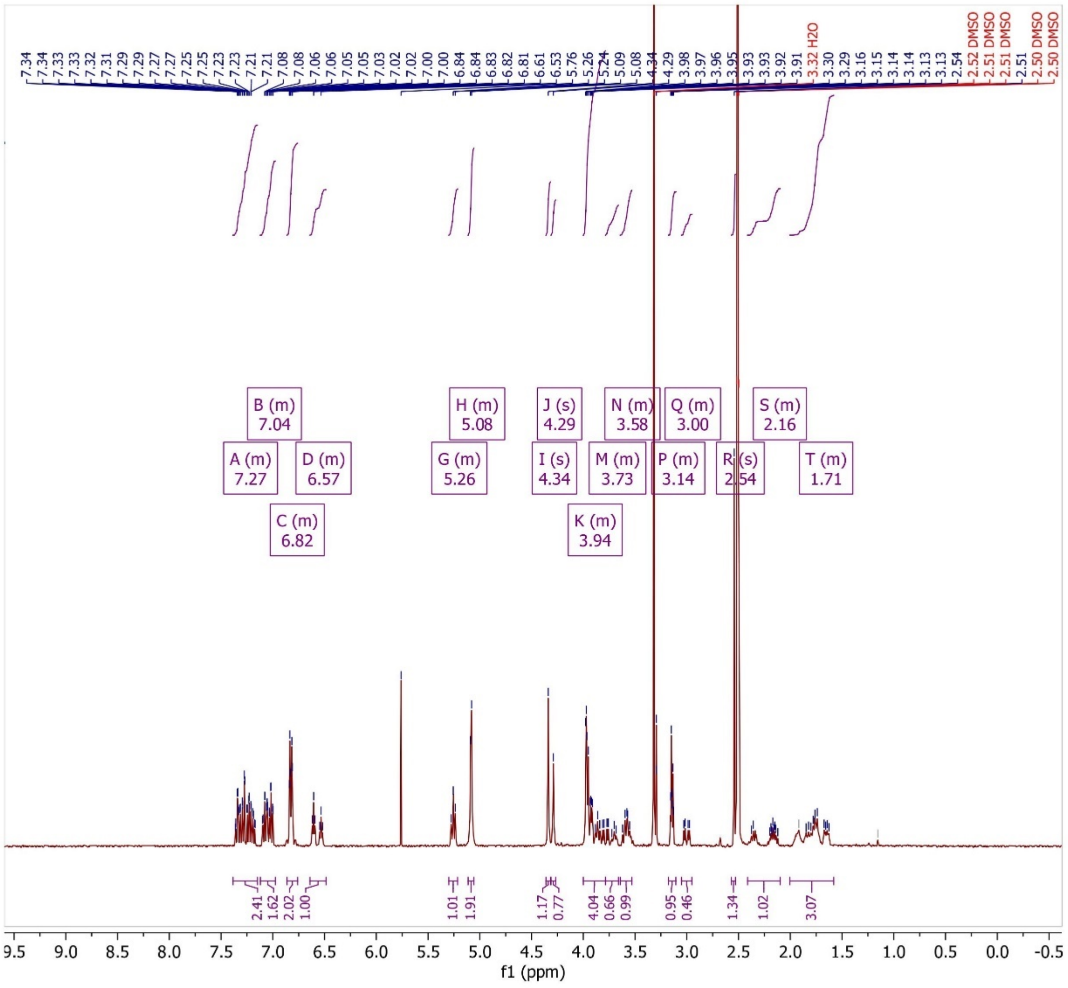

2-{[(4-Nitrophenyl)methyl]amino}acetonitrile (22af)

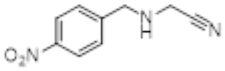

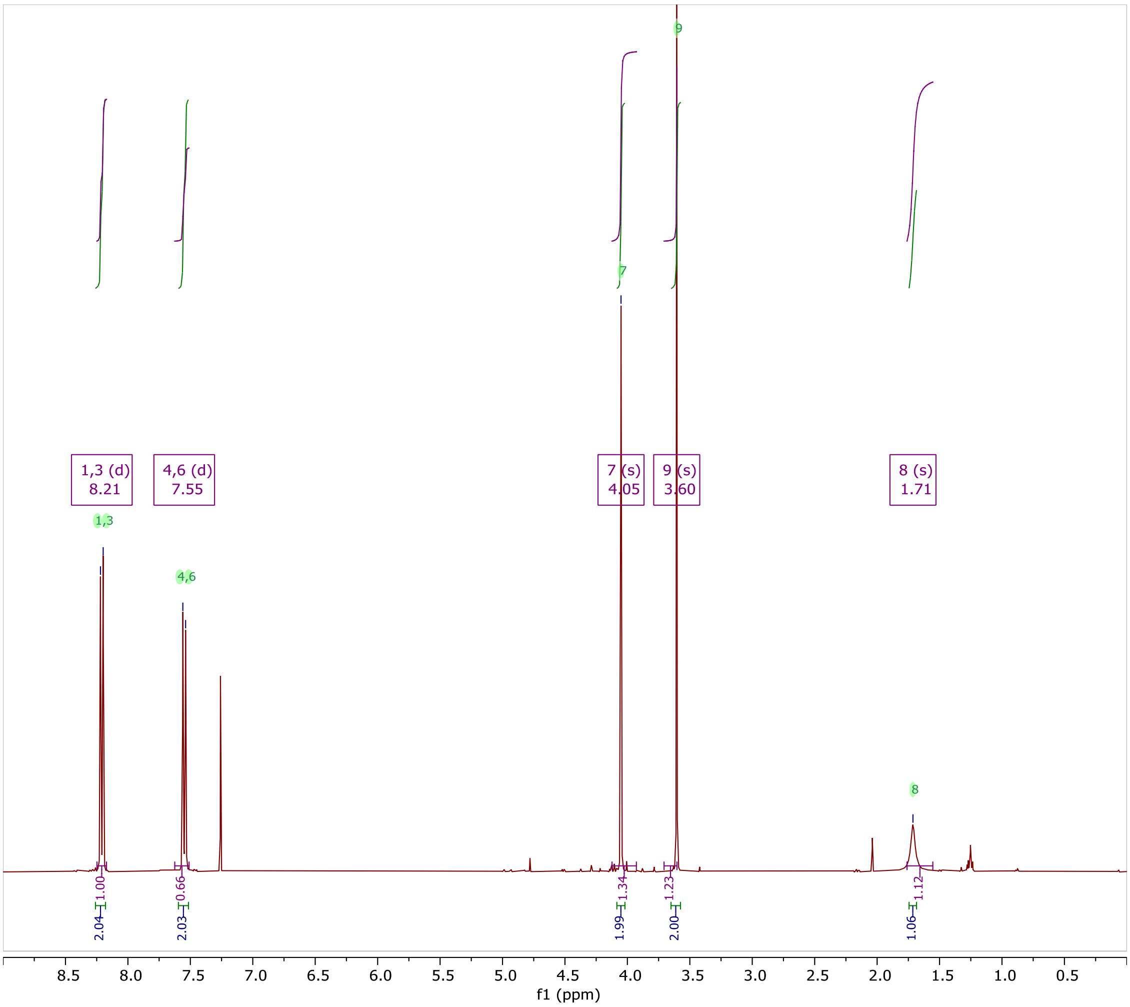

Ethyl 2-{[(cyanomethyl)[(4-nitrophenyl)methyl]carbamoyl]amino}acetate (23af)

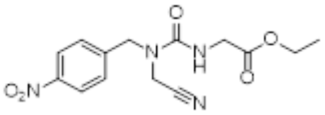

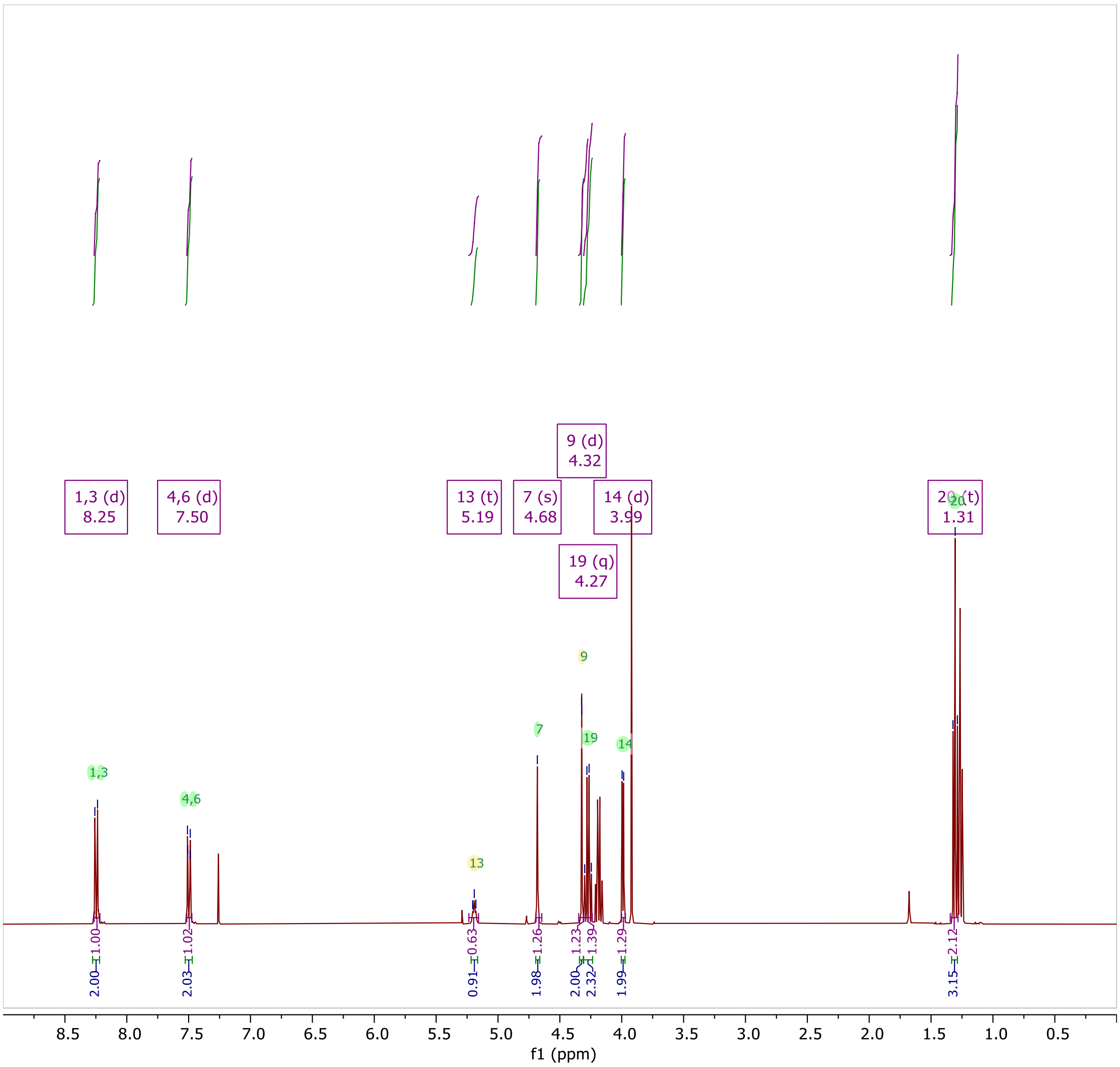

Ethyl 2-({[(4-nitrophenyl)methyl][(1*H*-1,2,3,4-tetrazol-5-yl)methyl]carbamoyl} amino) acetate (23ah)

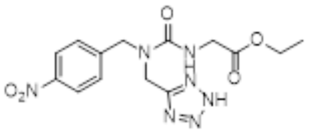

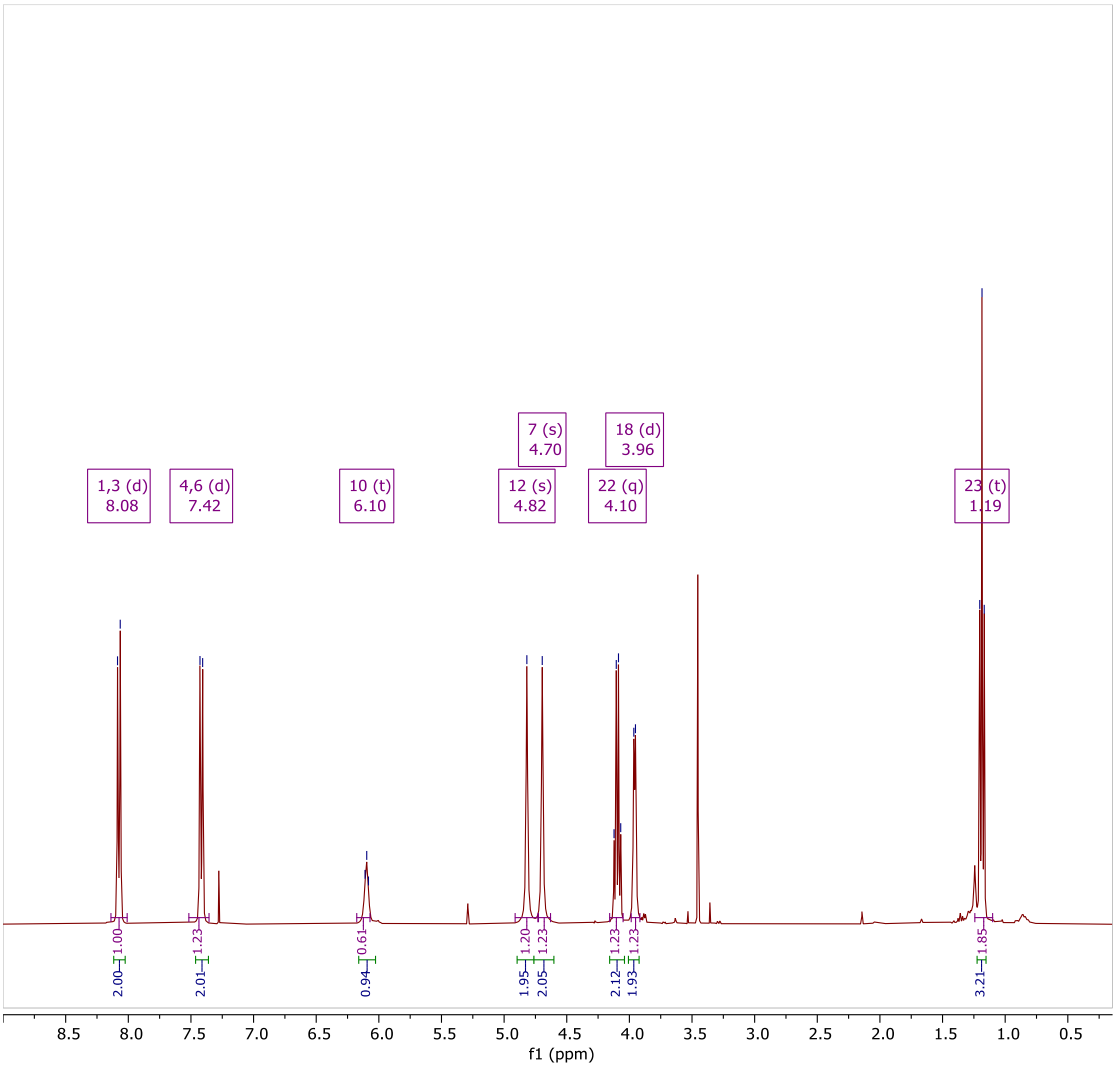

Ethyl 2-({[(2-cyclopentyl-2*H*-1,2,3,4-tetrazol-5-yl)methyl][(4-nitrophenyl)methyl] carbamoyl}amino)acetate (23ai)

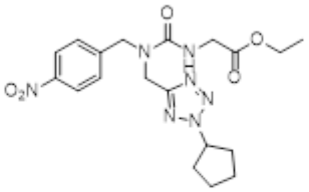

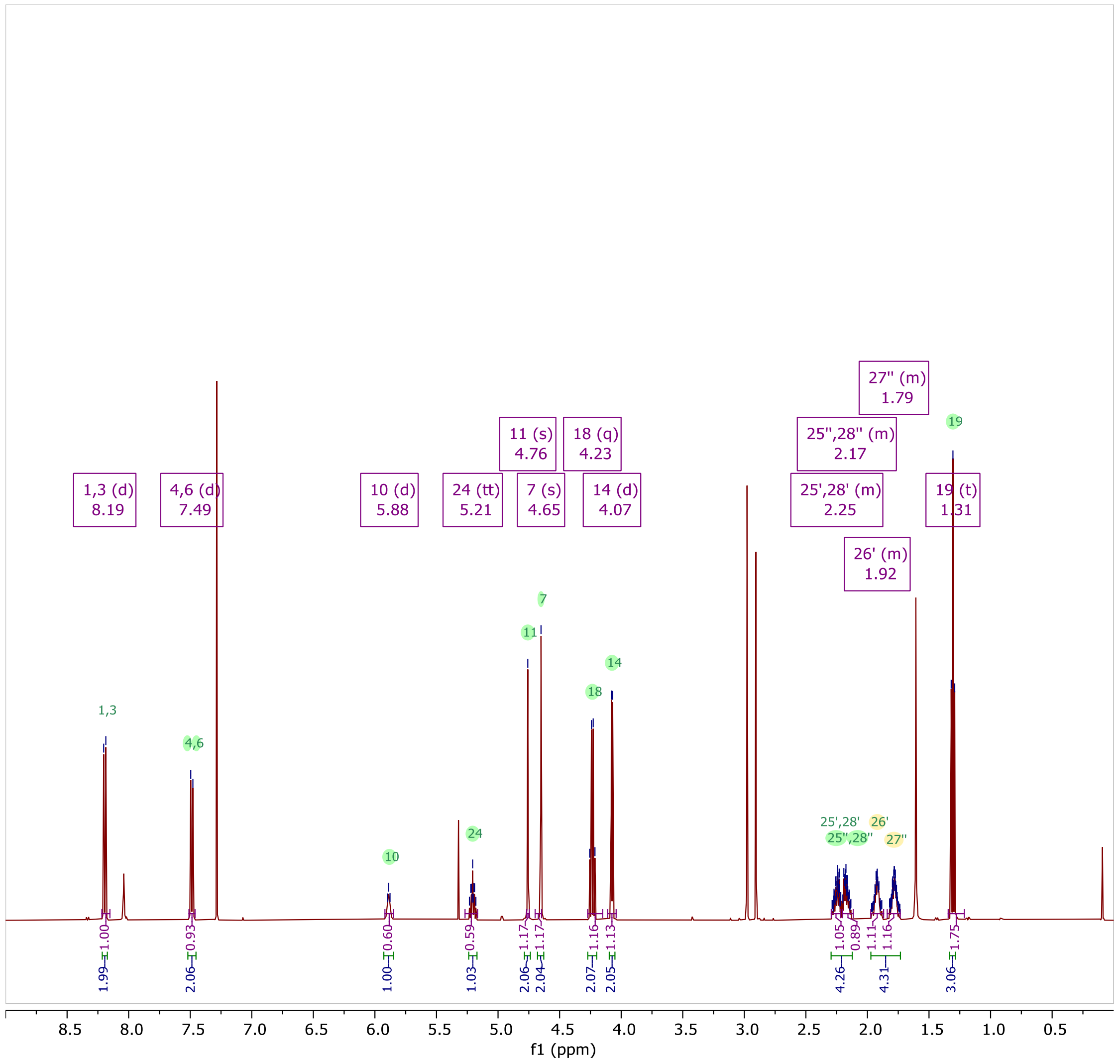

2-({[(2-Methyl-2*H*-1,2,3,4-tetrazol-5-yl)cyclopropyl][(4-anilino)methyl]carbamoyl}amino)acetic acid (25ai)

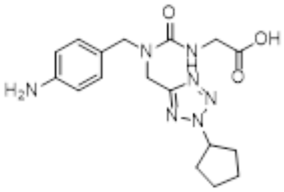

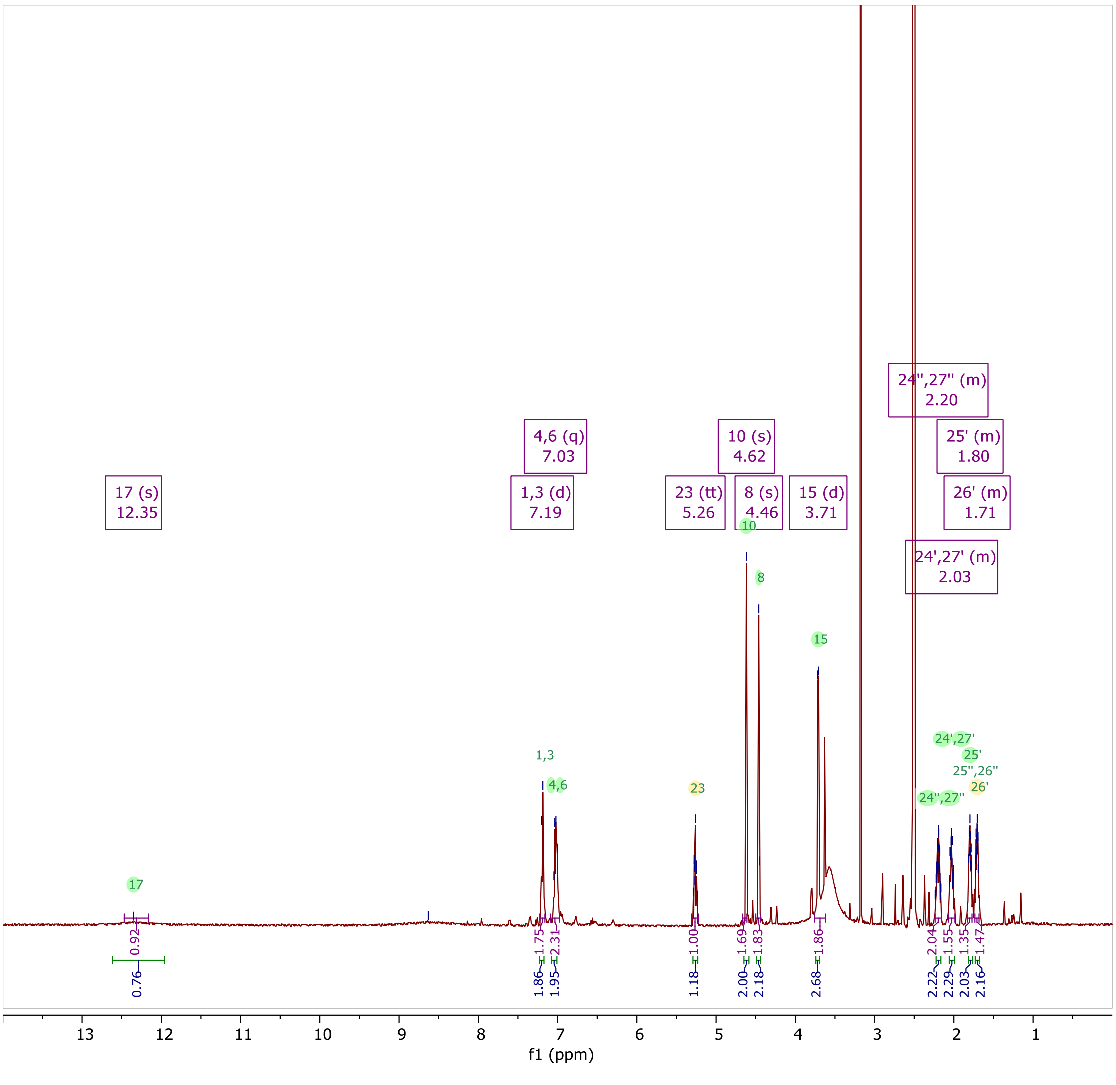

1-[(4-Aminophenyl)methyl]-3-{2-[2-(2-bromophenyl)pyrrolidin-1-yl]-2-oxoethyl}-1-[(2-methyl-2*H*-1,2,3,4-tetrazol-5-yl)cyclopentyl]urea hydrochloride (8)

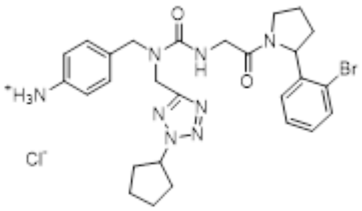

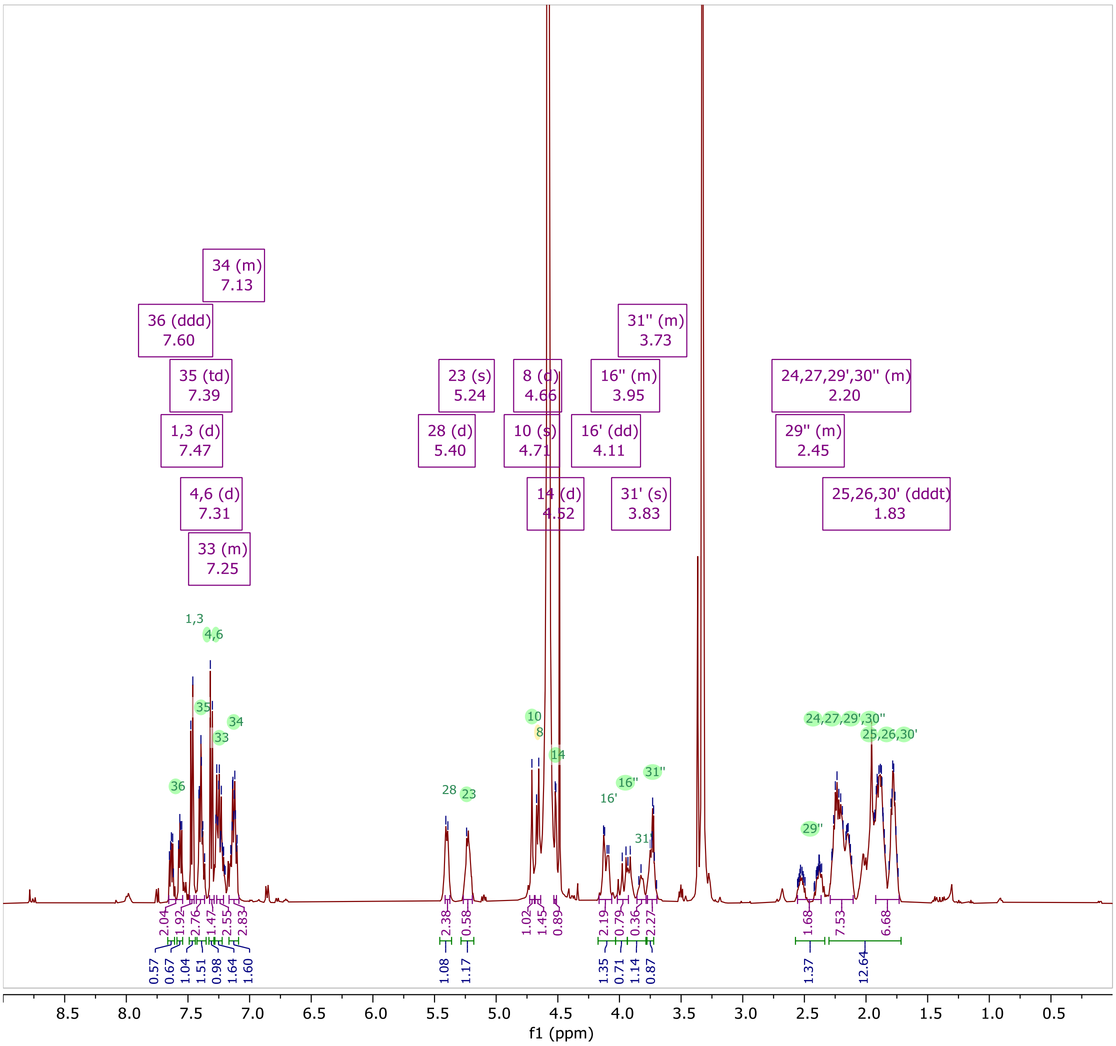

Ethyl 2-({[(2-cyclohexyl-2*H*-1,2,3,4-tetrazol-5-yl)methyl][(4-nitrophenyl)methyl] carbamoyl}amino)acetate (23aj)

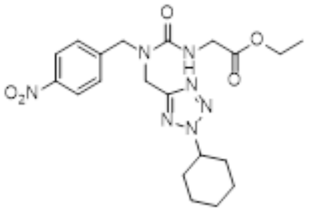

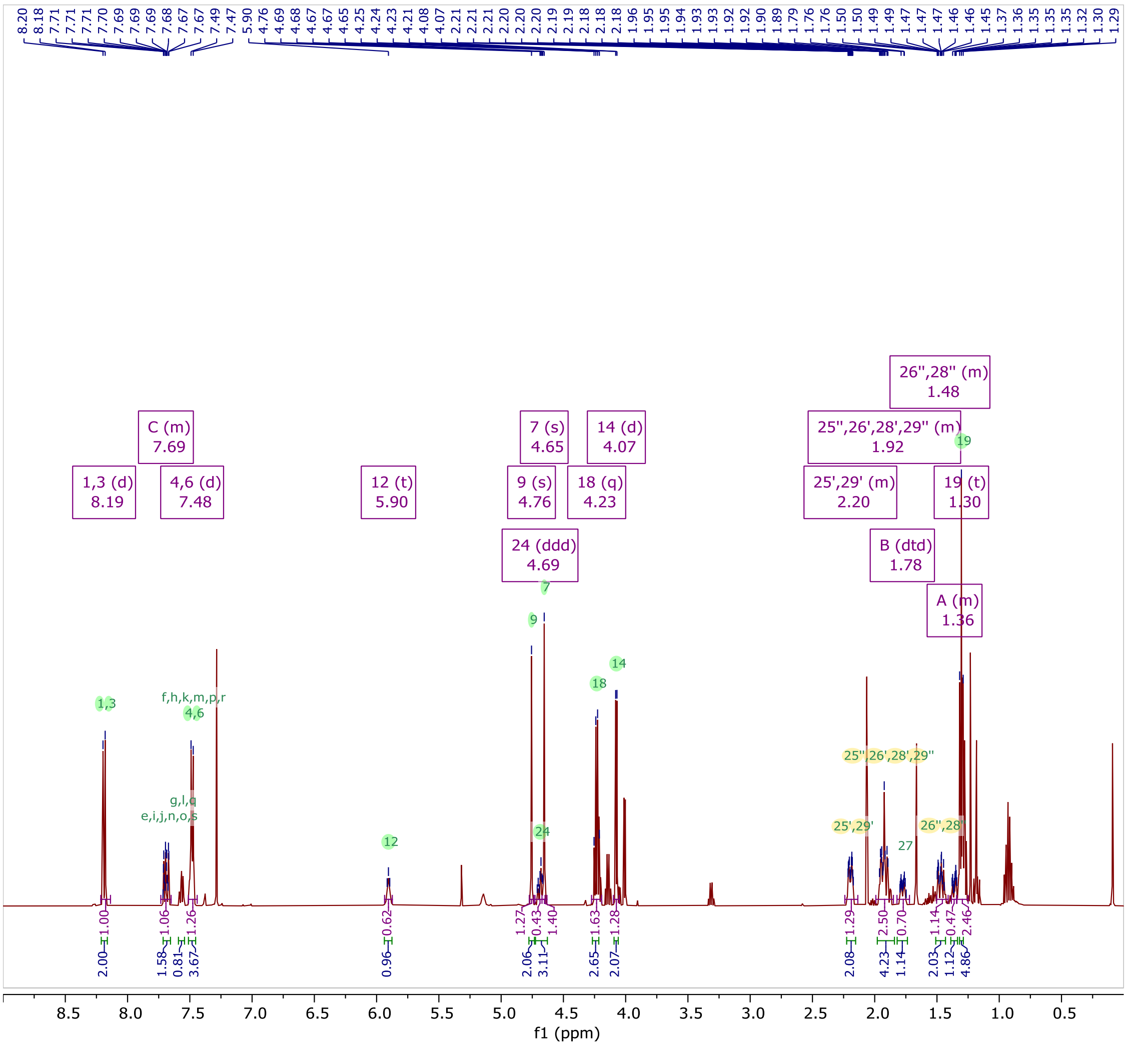

2-({[(2-Methyl-2*H*-1,2,3,4-tetrazol-5-yl)cyclohexyl][(4-aminophenyl)methyl] carbamoyl}amino)acetic acid (25aj)

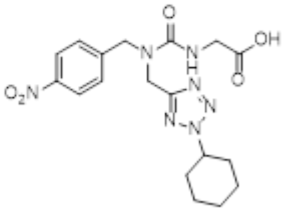

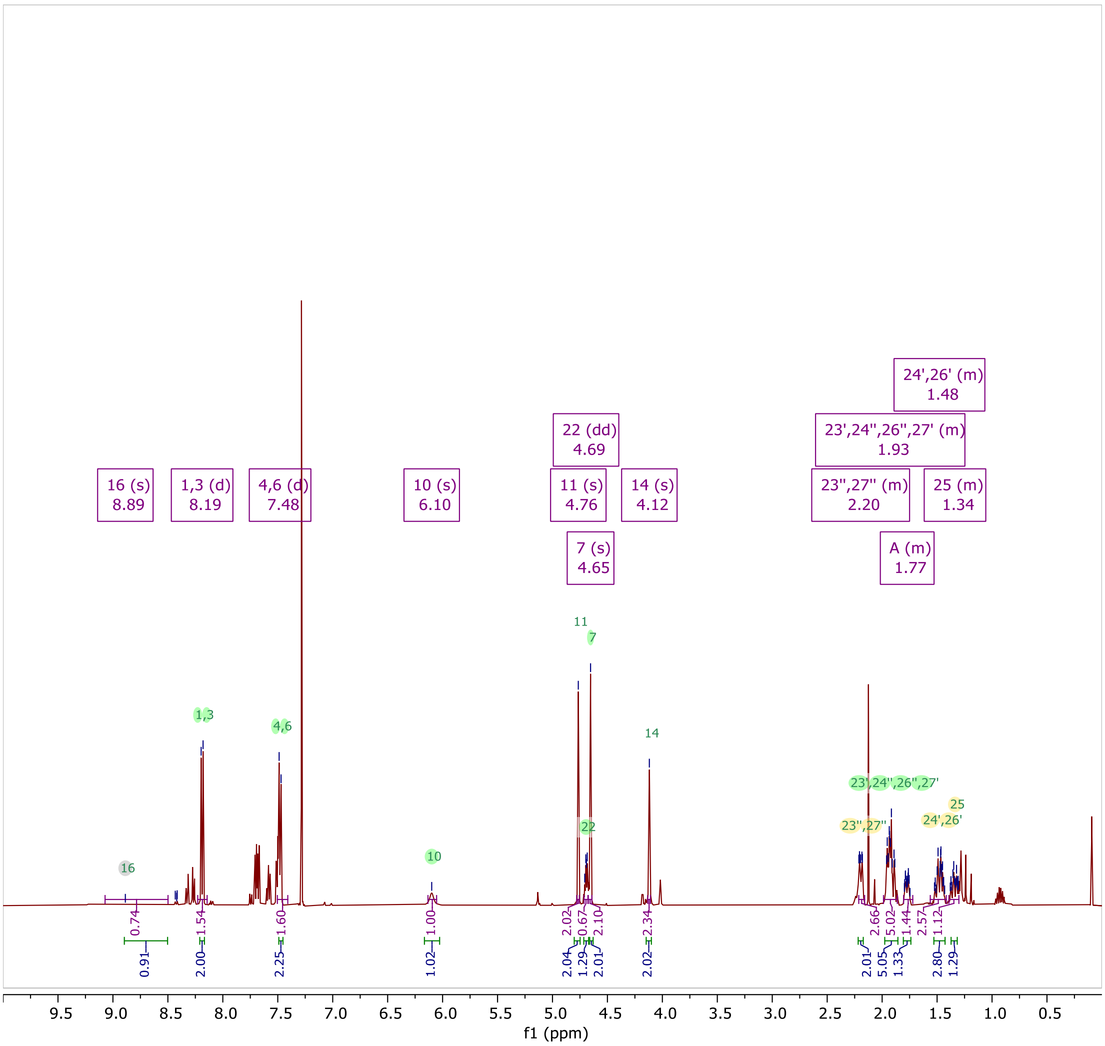

1-[(4-Aminophenyl)methyl]-3-{2-[2-(2-bromophenyl)pyrrolidin-1-yl]-2-oxoethyl}-1-[(2-methyl-2*H*-1,2,3,4-tetrazol-5-yl)cyclohexyl]urea hydrochloride (9)

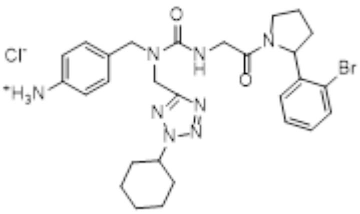

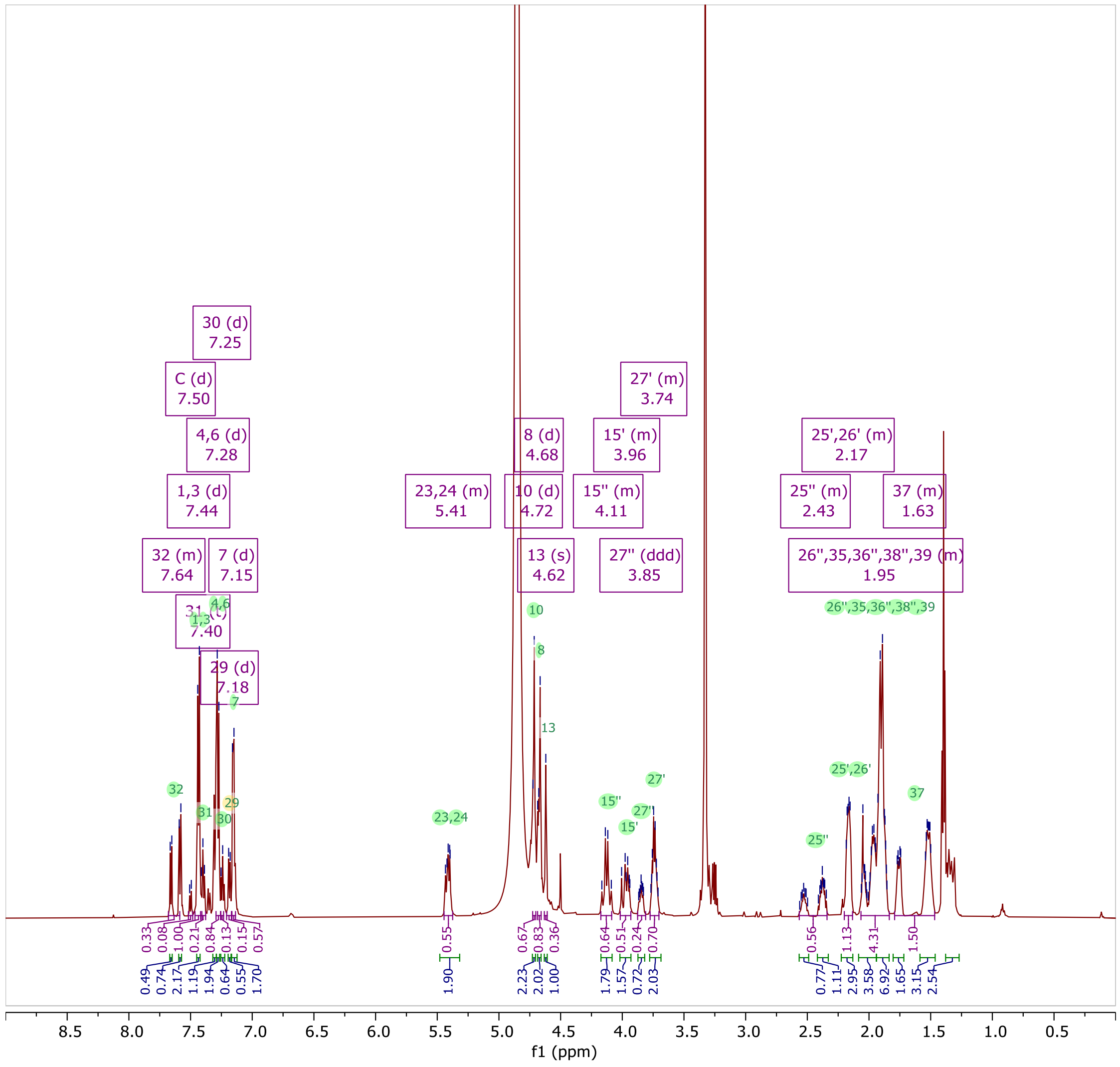

2-Chloro-4-[[(2-methyltetrazol-5-yl)methylamino]methyl]aniline (22da)

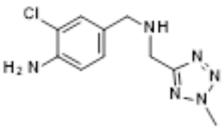

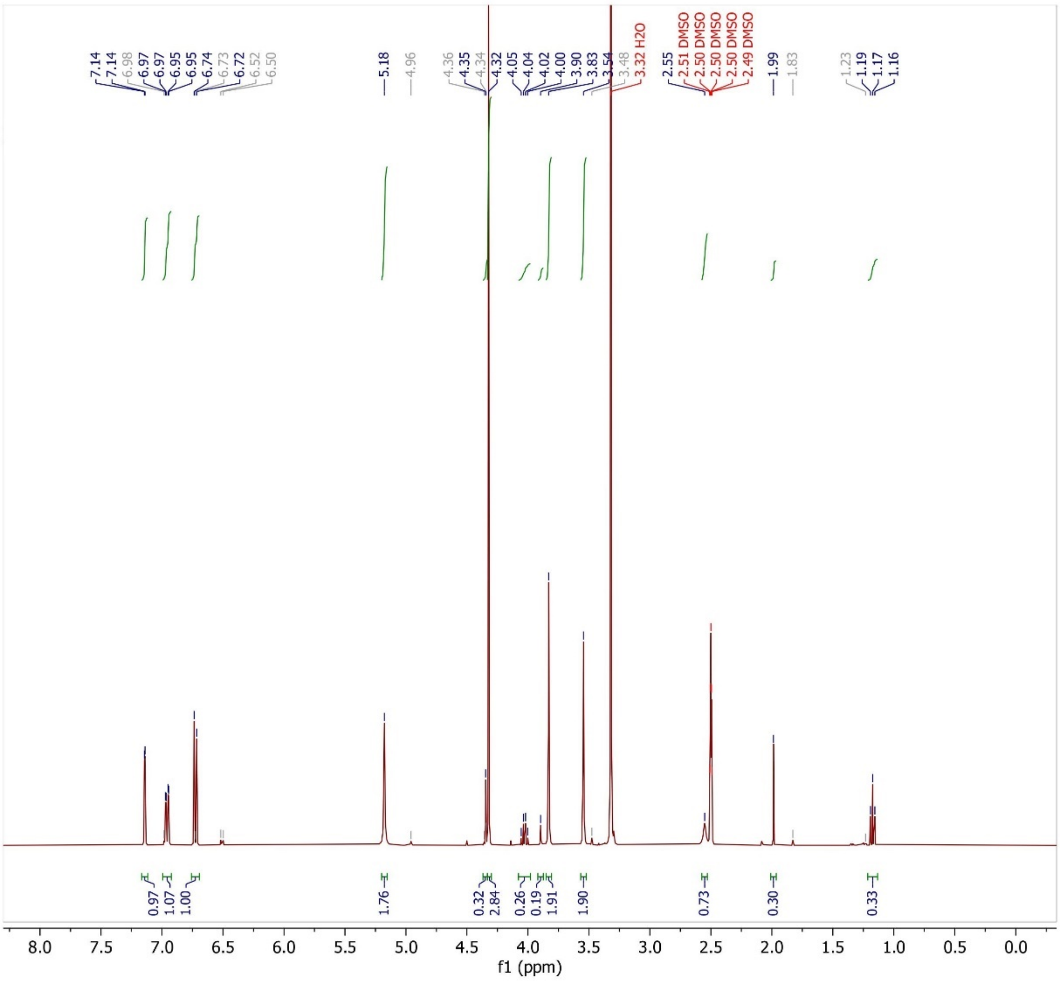

Ethyl 2-[[(4-amino-3-chloro-phenyl)methyl-[(2-methyltetrazol-5-yl)methyl] carbamoyl] amino]acetate (23da)

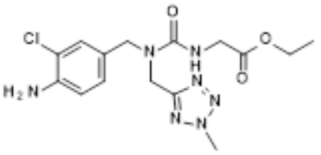

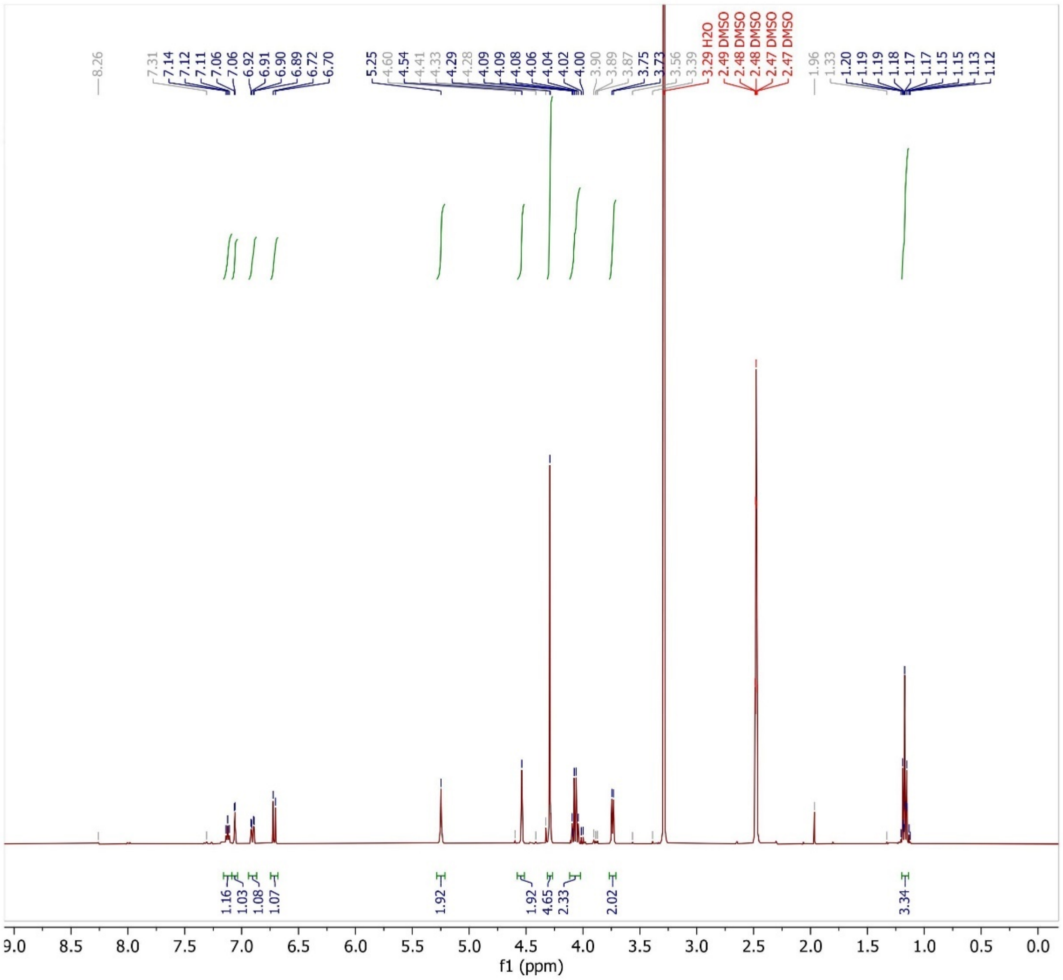

2-[[(4-Amino-3-chloro-phenyl)methyl-[(2-methyltetrazol-5-yl)methyl] carbamoyl]amino] acetic acid (25da)

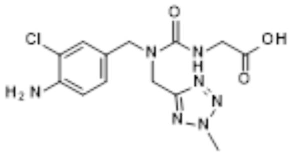

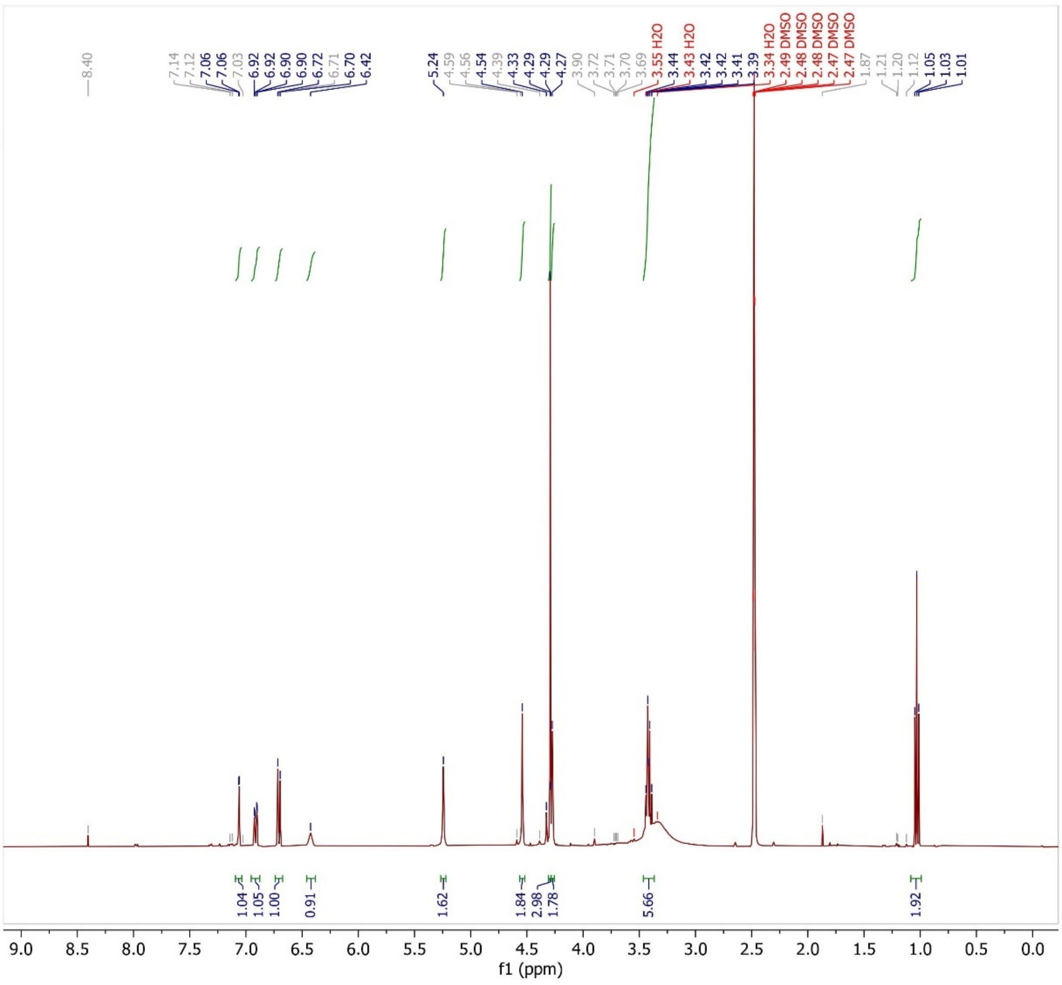

1-[(4-Amino-3-chloro-phenyl)methyl]-1-[(2-methyltetrazol-5-yl)methyl]-3-[2-oxo-2-[(2*R*)-2-(2-bromophenyl)pyrrolidin-1-yl]ethyl]urea (10)

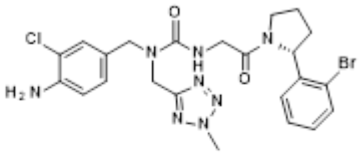

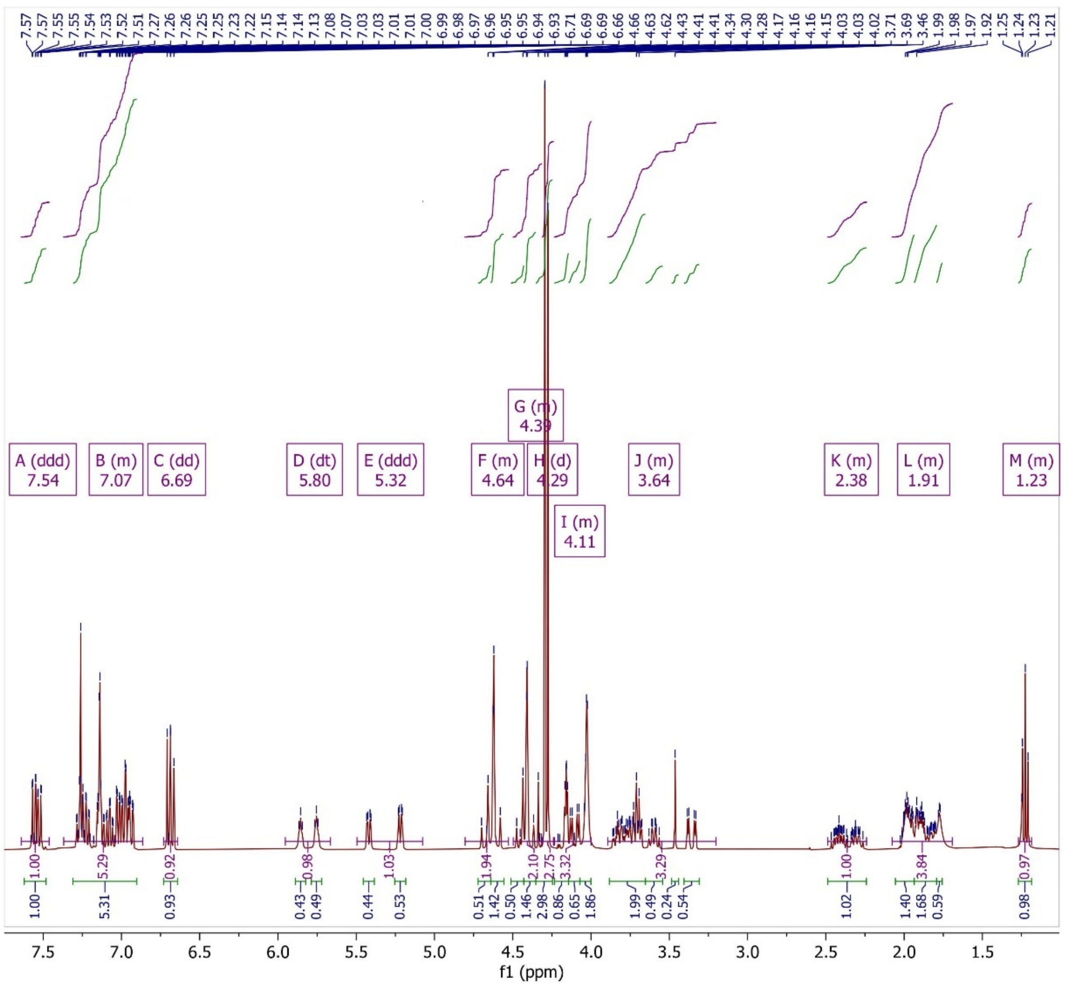

1-[(4-Amino-3-chloro-phenyl)methyl]-1-[(2-methyltetrazol-5-yl)methyl]-3-[2-oxo-2-[(2*R*)-2-(2-methylsulfanylphenyl)pyrrolidin-1-yl]ethyl]urea (11)

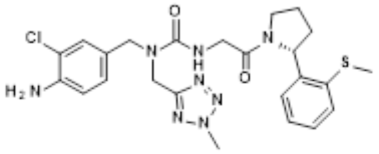

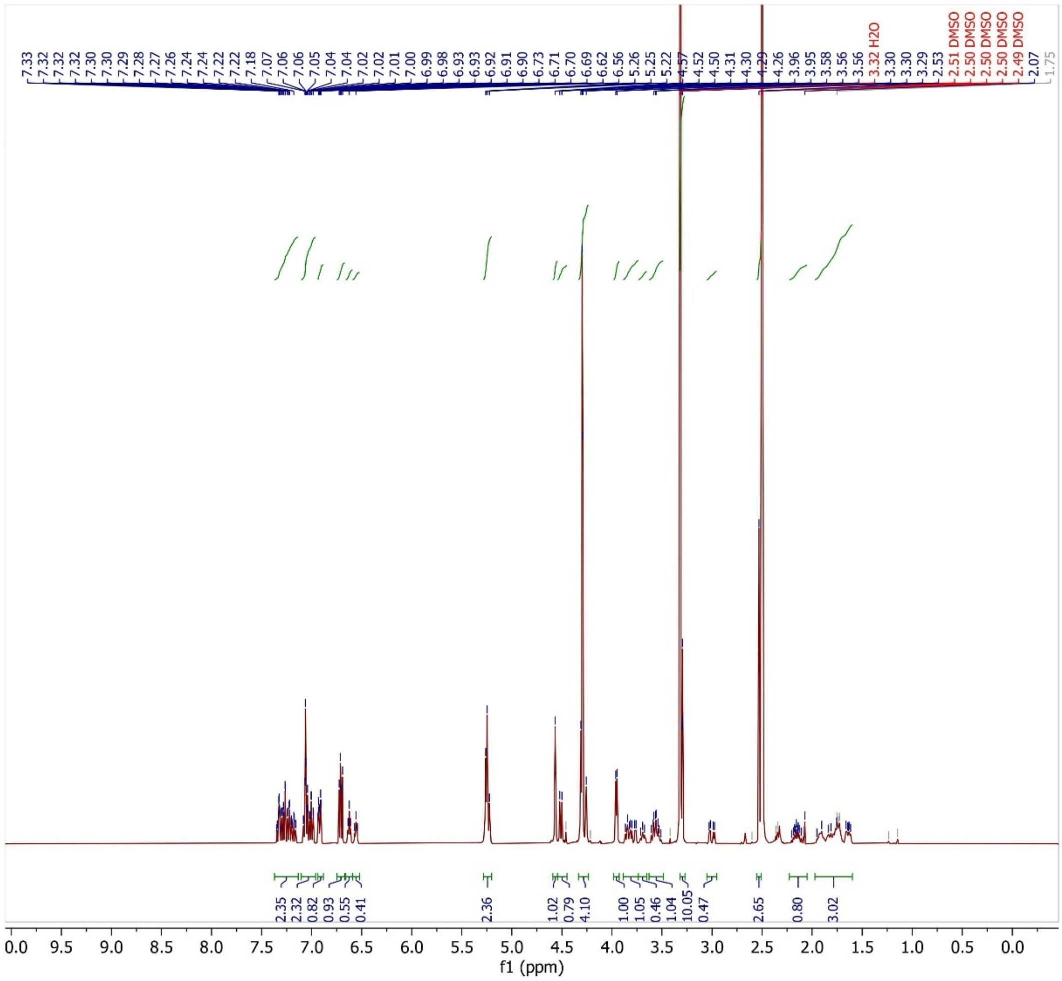

tert-Butyl _N_-_tert_-butoxycarbonyl-_N_-[5-[[(5-methylthiazol-2-yl)methylamino] methyl] pyrimidin-2-yl]carbamate (22eb)

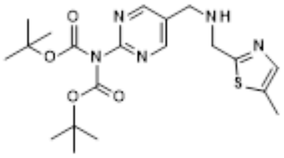

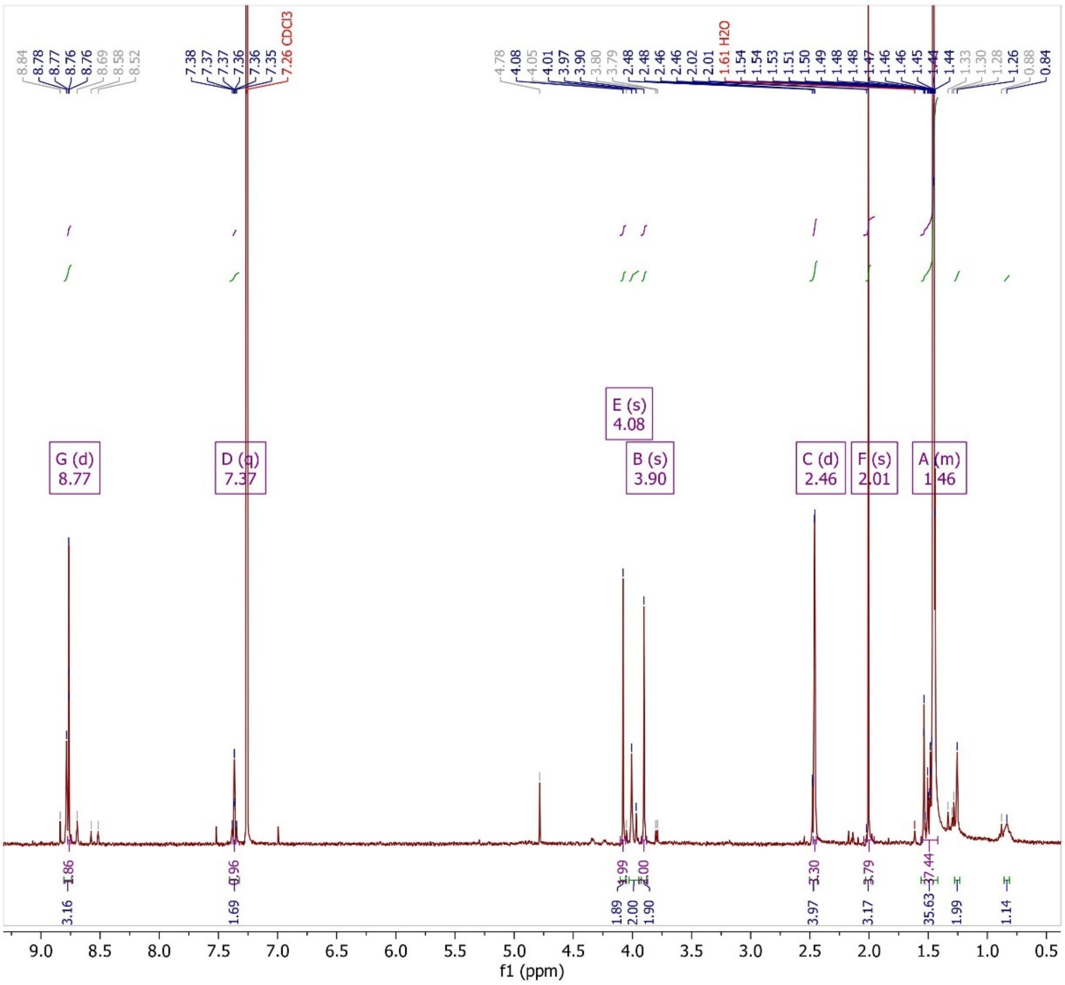

Ethyl 2-[[[2-[bis(*tert*-butoxycarbonyl)amino]pyrimidin-5-yl]methyl-[(5-methylthiazol -2-yl)methyl]carbamoyl]amino]acetate (23eb)

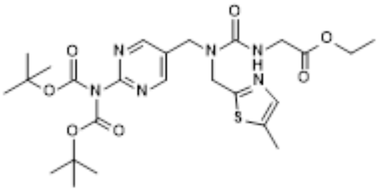

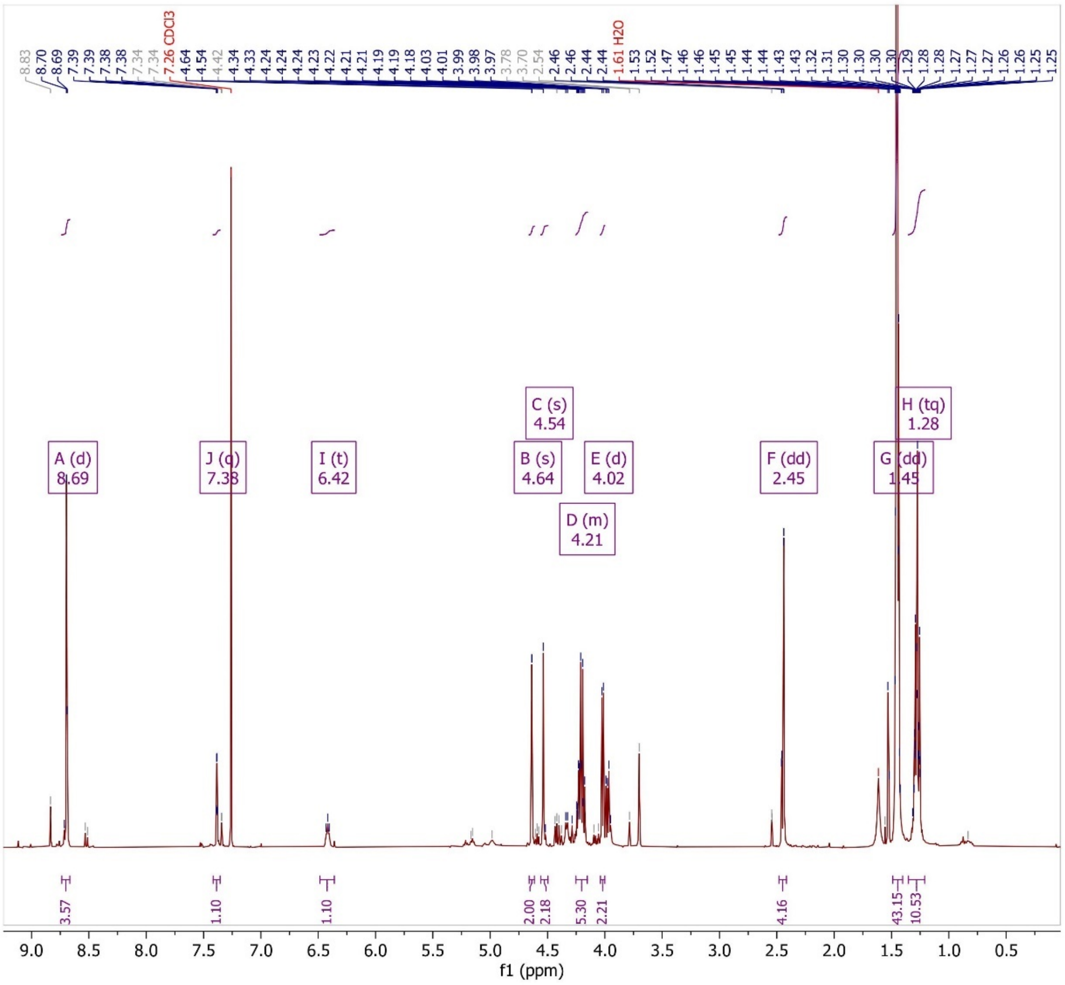

2-[[[2-[Bis(*tert*-butoxycarbonyl)amino]pyrimidin-5-yl]methyl-[(5-methylthiazol-2-yl)methyl]carbamoyl]amino]acetic acid (25eb)

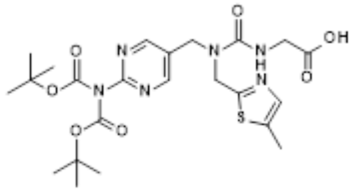

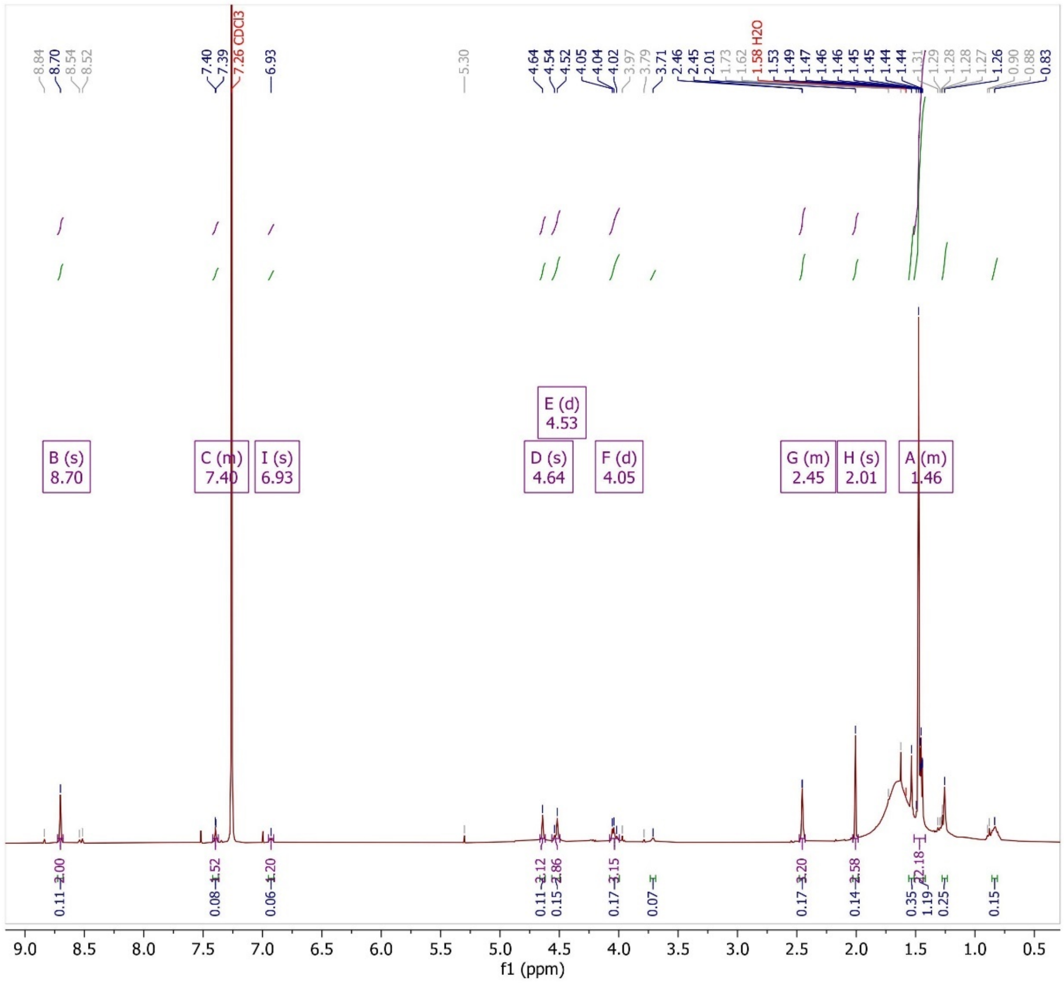

1-[(2-Aminopyrimidin-5-yl)methyl]-1-[(5-methylthiazol-2-yl)methyl]-3-[2-oxo-2-[(2*R*)-2-(2-methylsulfanylphenyl)pyrrolidin-1-yl]ethyl]urea (12)

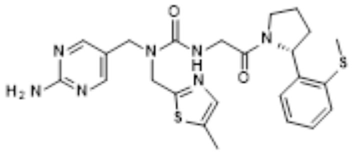

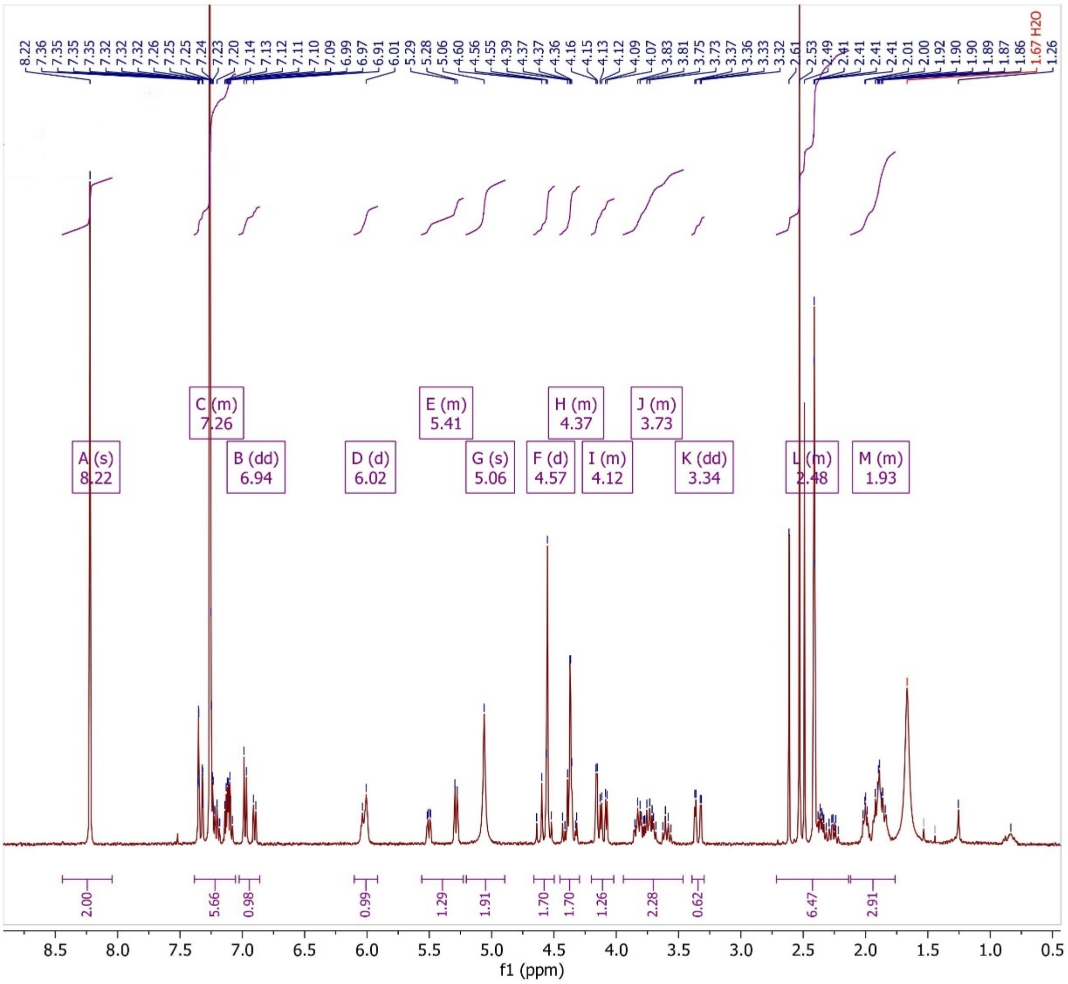

Ethyl 2-[[[2-[bis(*tert*-butoxycarbonyl)amino]pyrimidin-5-yl]methyl-[(5-methyl-1,3,4-thiadiazol-2-yl)methyl]carbamoyl]amino]acetate (23ec)

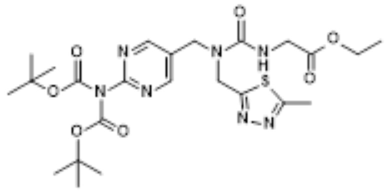

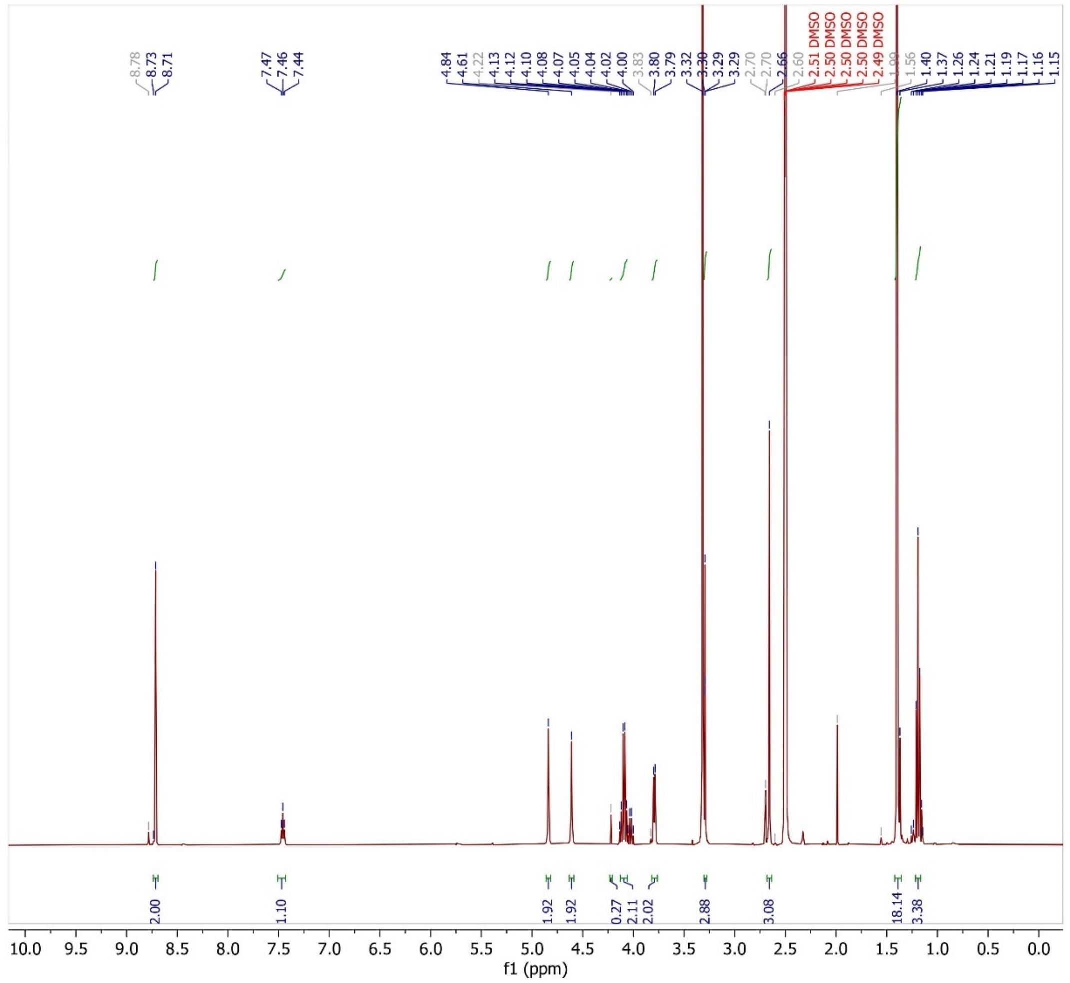

1-[(2-Aminopyrimidin-5-yl)methyl]-1-[(5-methyl-1,3,4-thiadiazol-2-yl)methyl]-3-[2-oxo-2-[(2*R*)-2-(2-methylsulfanylphenyl)pyrrolidin-1-yl]ethyl]urea (13)

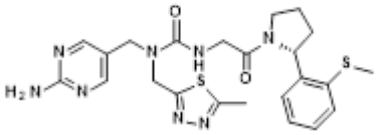

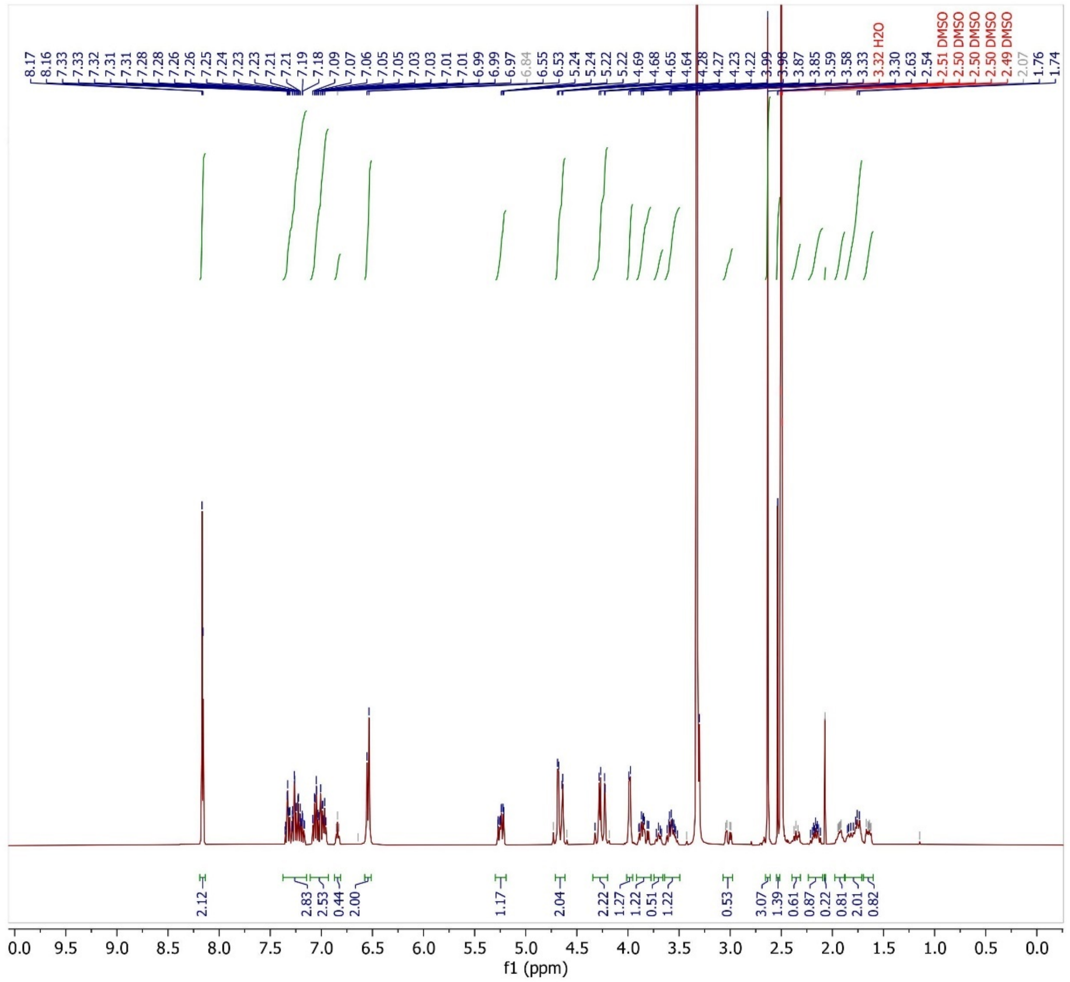

3-Chloro-5-[[(5-methyl-1,3,4-thiadiazol-2-yl)methylamino]methyl]pyridin-2-amine (22fc)

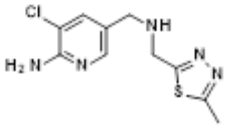

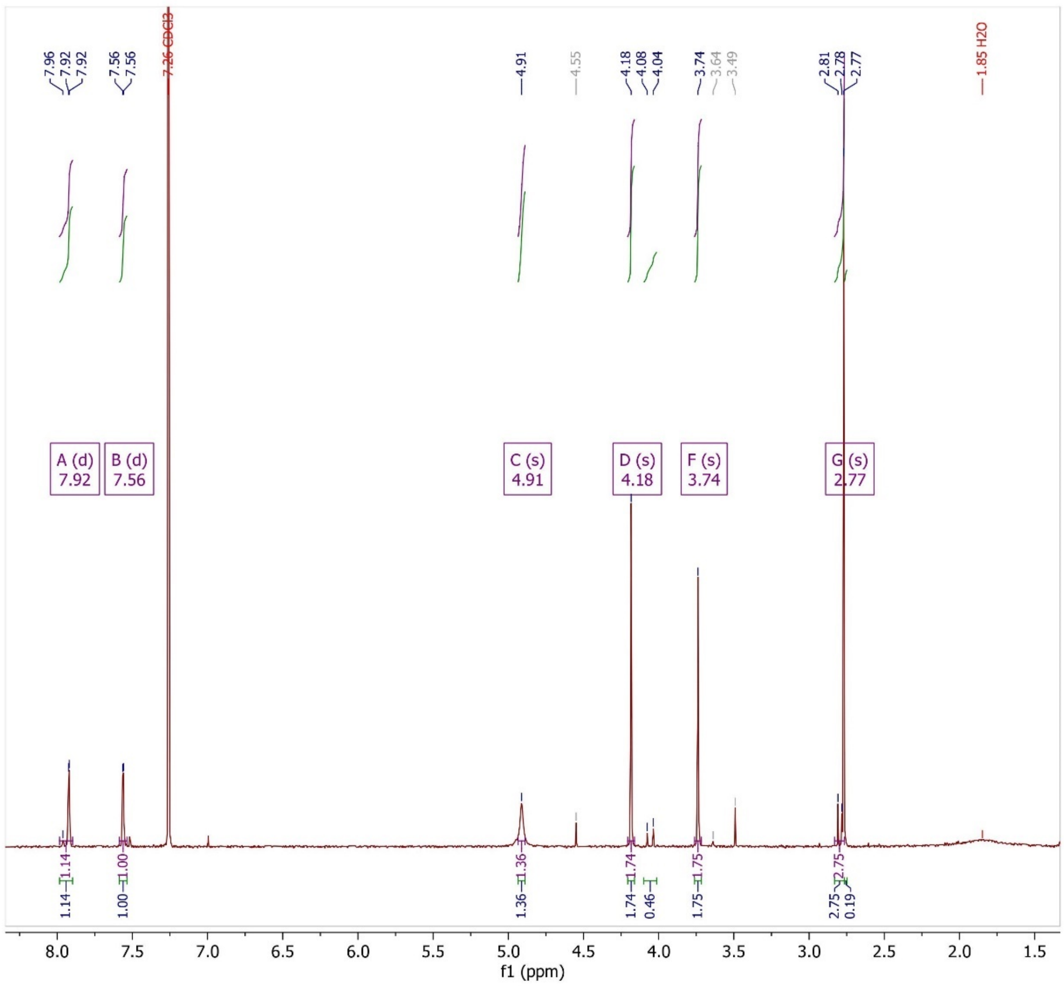

Ethyl 2-[[(6-amino-5-chloro-3-pyridyl)methyl-[(5-methyl-1,3,4-thiadiazol-2-yl) methyl] carbamoyl]amino]acetate (23fc)

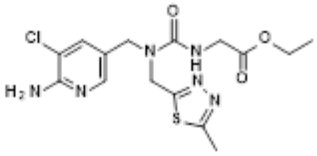

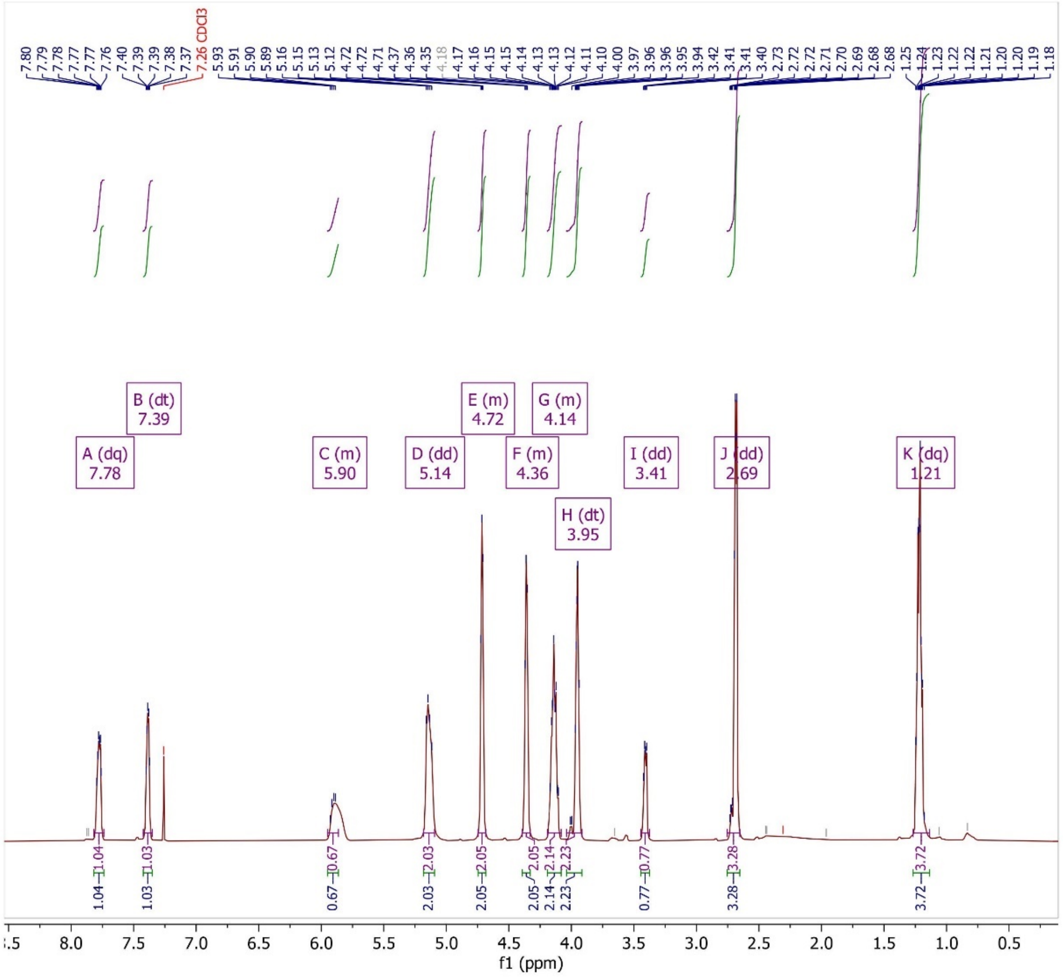

1-[(6-Amino-5-chloro-3-pyridyl)methyl]-1-[(5-methyl-1,3,4-thiadiazol-2-yl)methyl]-3-[2-oxo-2-[(2*R*)-2-(2-methylsulfanylphenyl)pyrrolidin-1-yl]ethyl]urea (14)

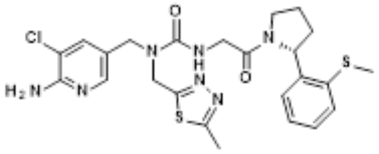

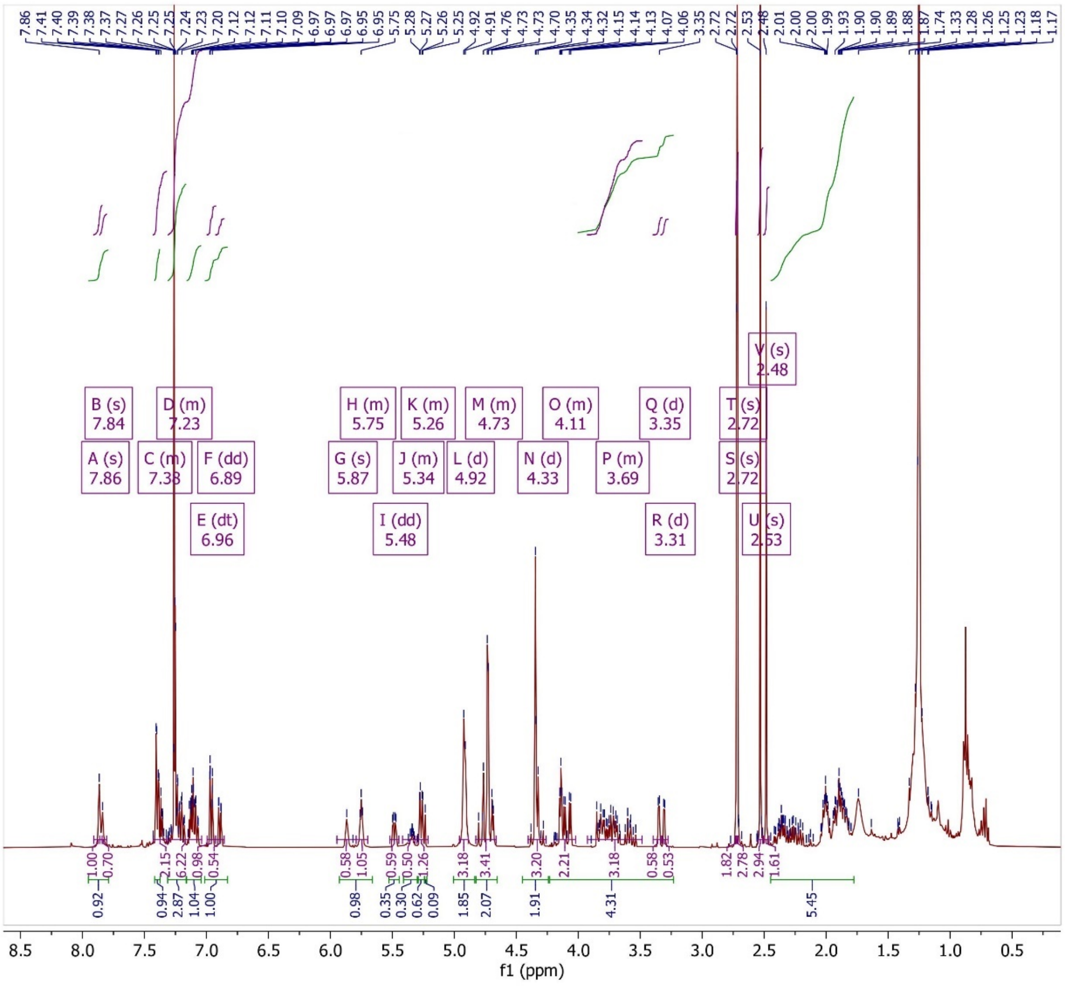

2-[(4-Amino-3,5-difluoro-phenyl)methylamino]acetonitrile (22cf)

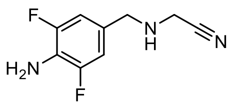

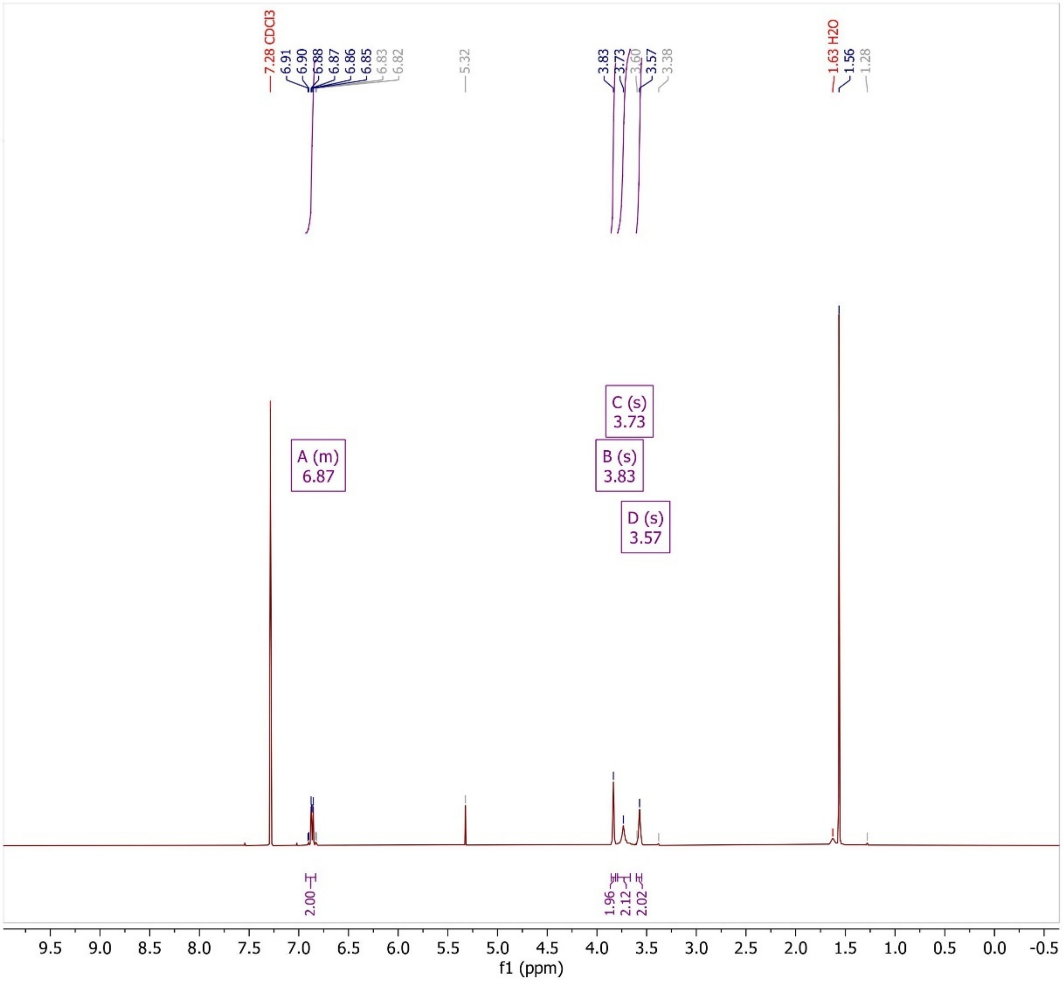

Ethyl 2-[[(4-amino-3,5-difluoro-phenyl)methyl-(cyanomethyl)carbamoyl] amino]acetate (23cf)

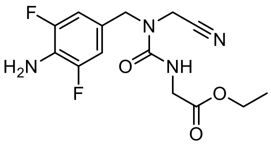

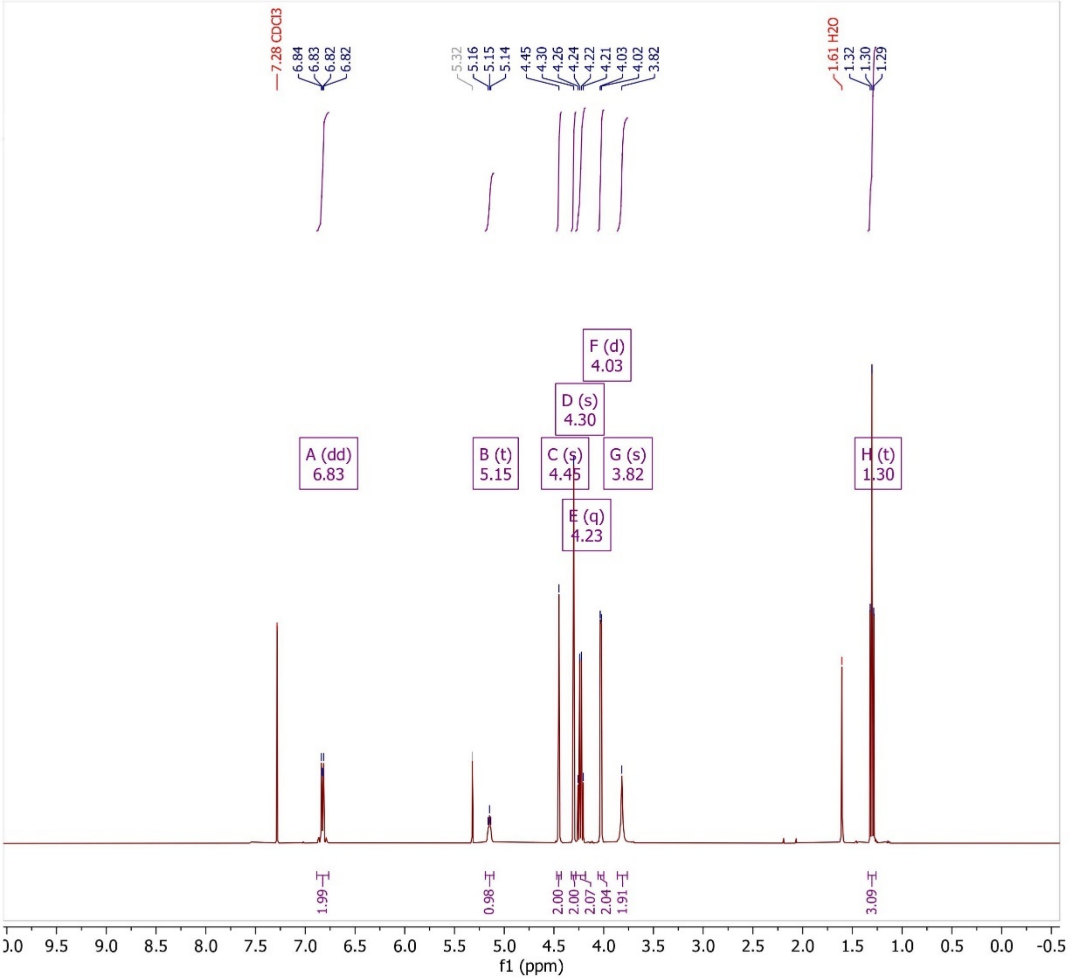

1-[(4-Amino-3,5-difluoro-phenyl)methyl]-3-[2-oxo-2-[(2*R*)-2-(2-methylsulfanylphenyl) pyrrolidine-1-yl]ethyl]imidazolidine-2,4-dione (15)

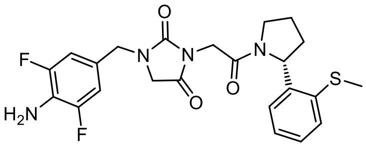

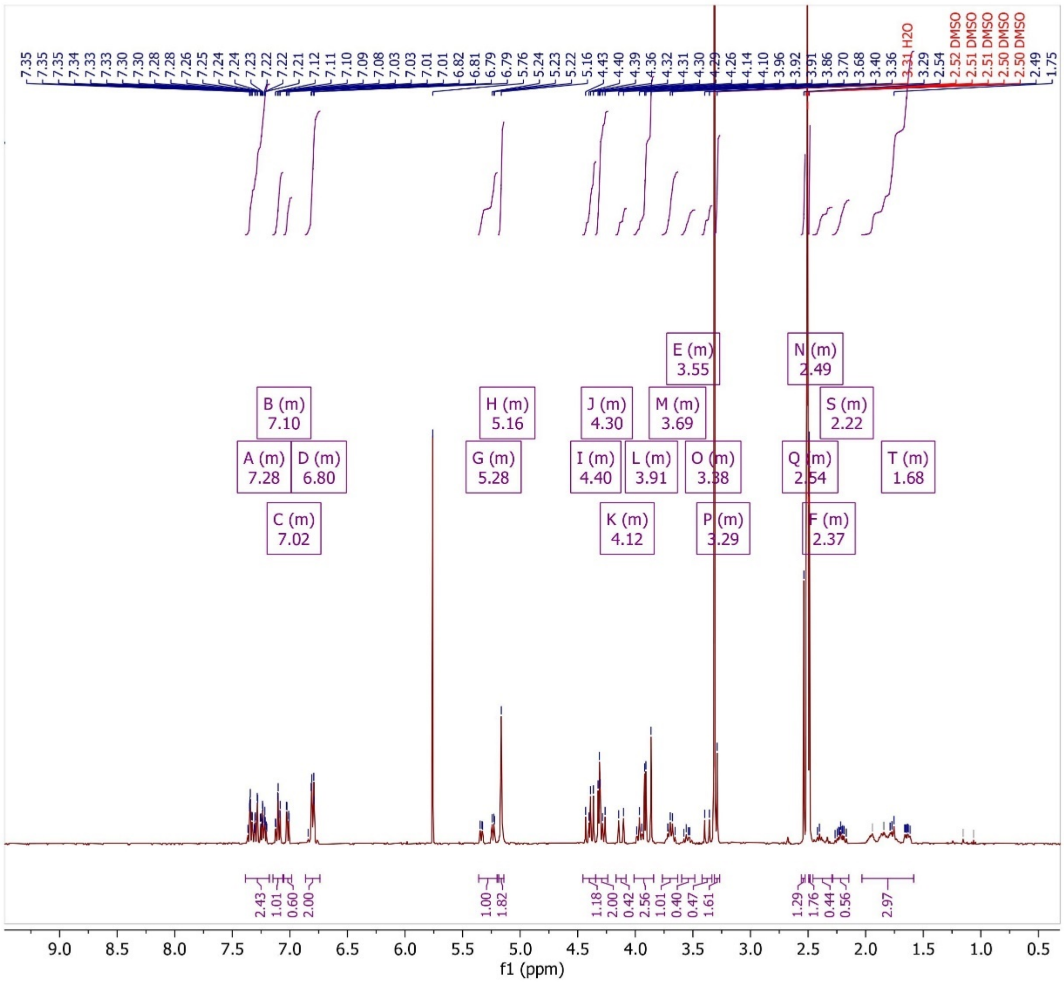

2-Chloro-4-[[(5-methyl-1,3,4-oxadiazol-2-yl)methylamino]methyl]aniline (22dd)

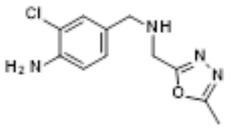

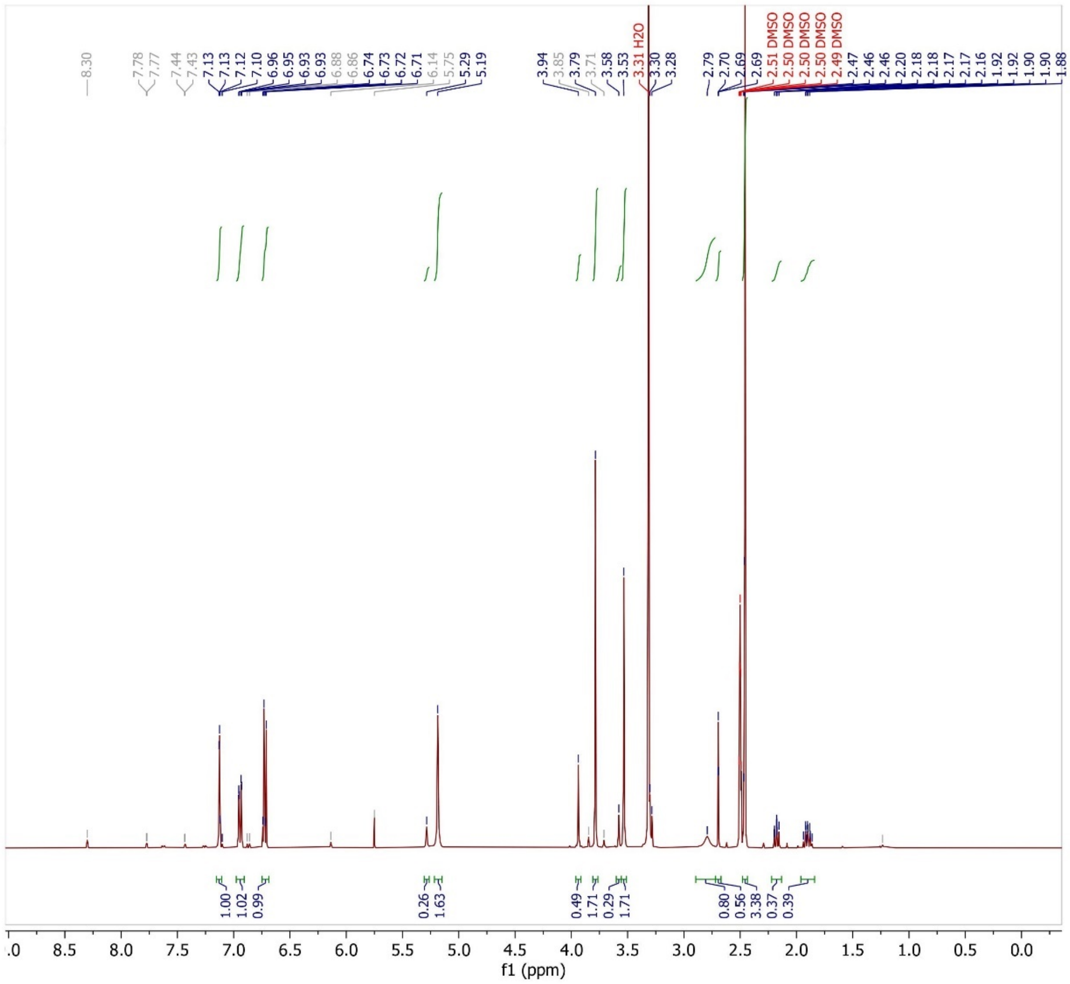

Ethyl 2-[[(4-amino-3-chloro-phenyl)methyl-[(5-methyl-1,3,4-oxadiazol-2-yl)methyl] carbamoyl]amino]acetate (23dd)

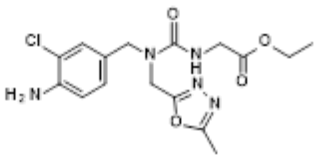

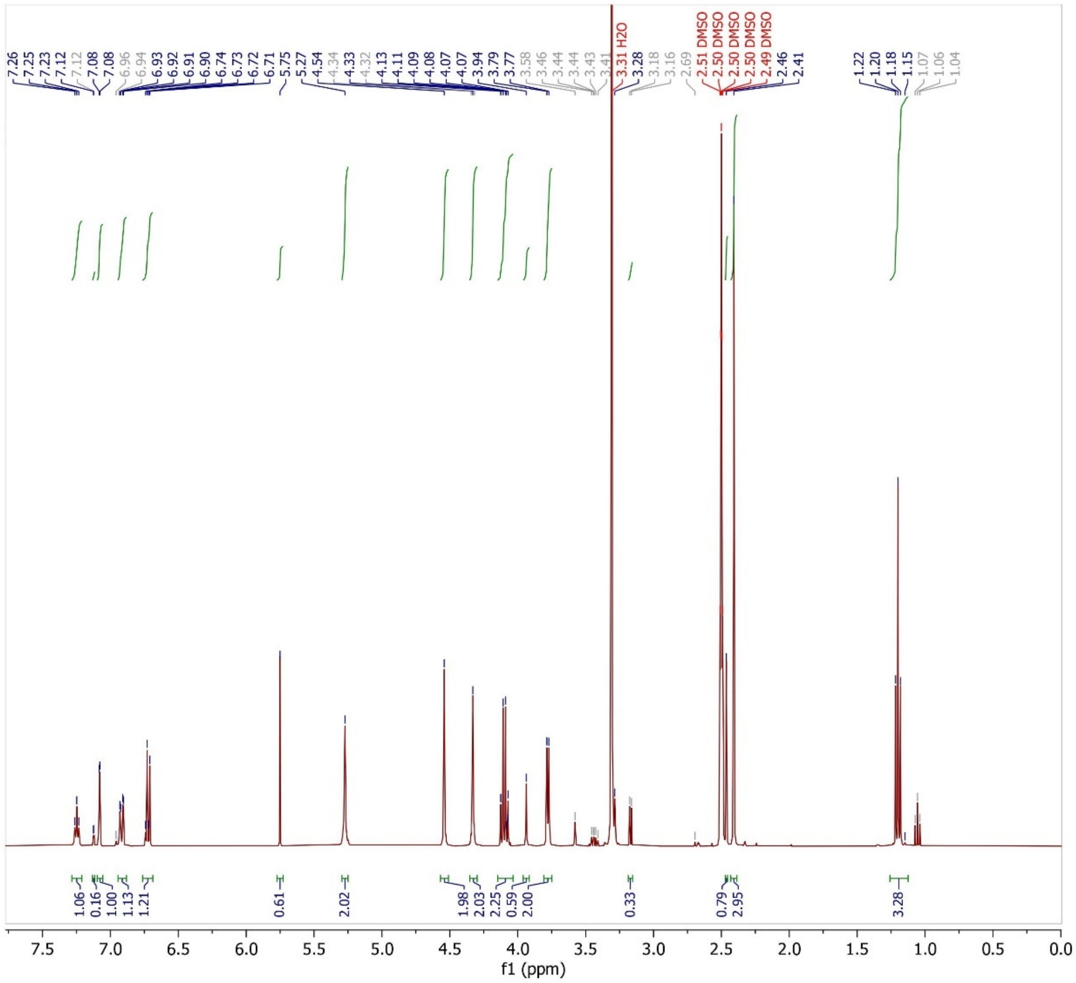

***N*-[[1-[(4-Amino-3-chloro-phenyl)methyl]-2-oxo-3-[2-oxo-2-[(2*R*)-2-(2-methylsulfanyl phenyl)pyrrolidin-1-yl]ethyl]imidazolidin-4-ylidene]amino]acetamide (16)**

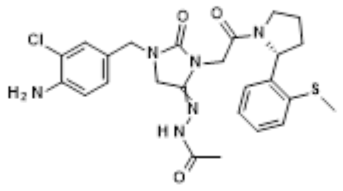

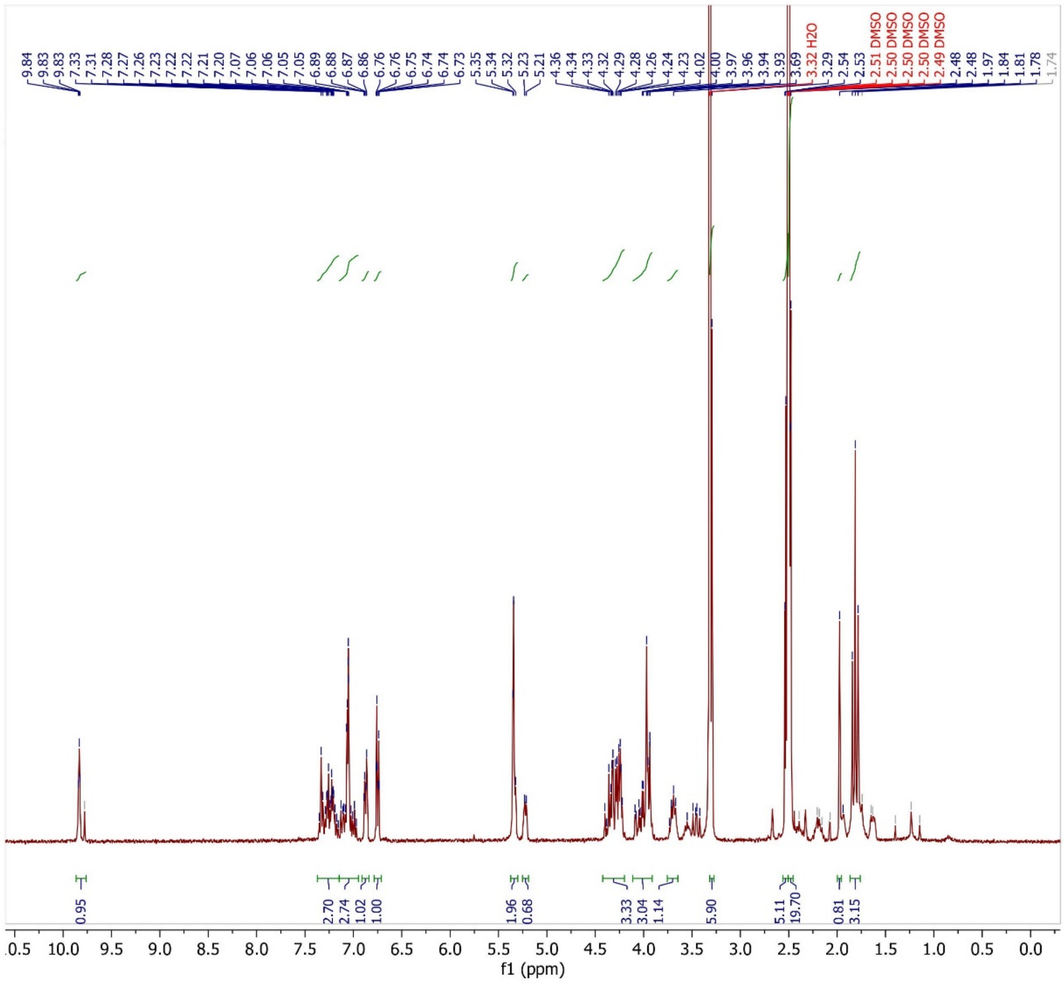

***N*-[[1-[(4-Amino-3-chloro-phenyl)methyl]-3-[2-[(2*R*)-2-(2-bromophenyl)pyrrolidin-1-yl]-2-oxo-ethyl]-2-oxo-imidazolidin-4-ylidene]amino]acetamide (17)**

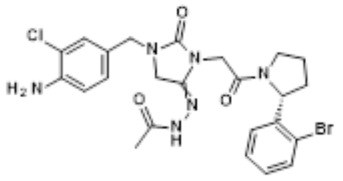

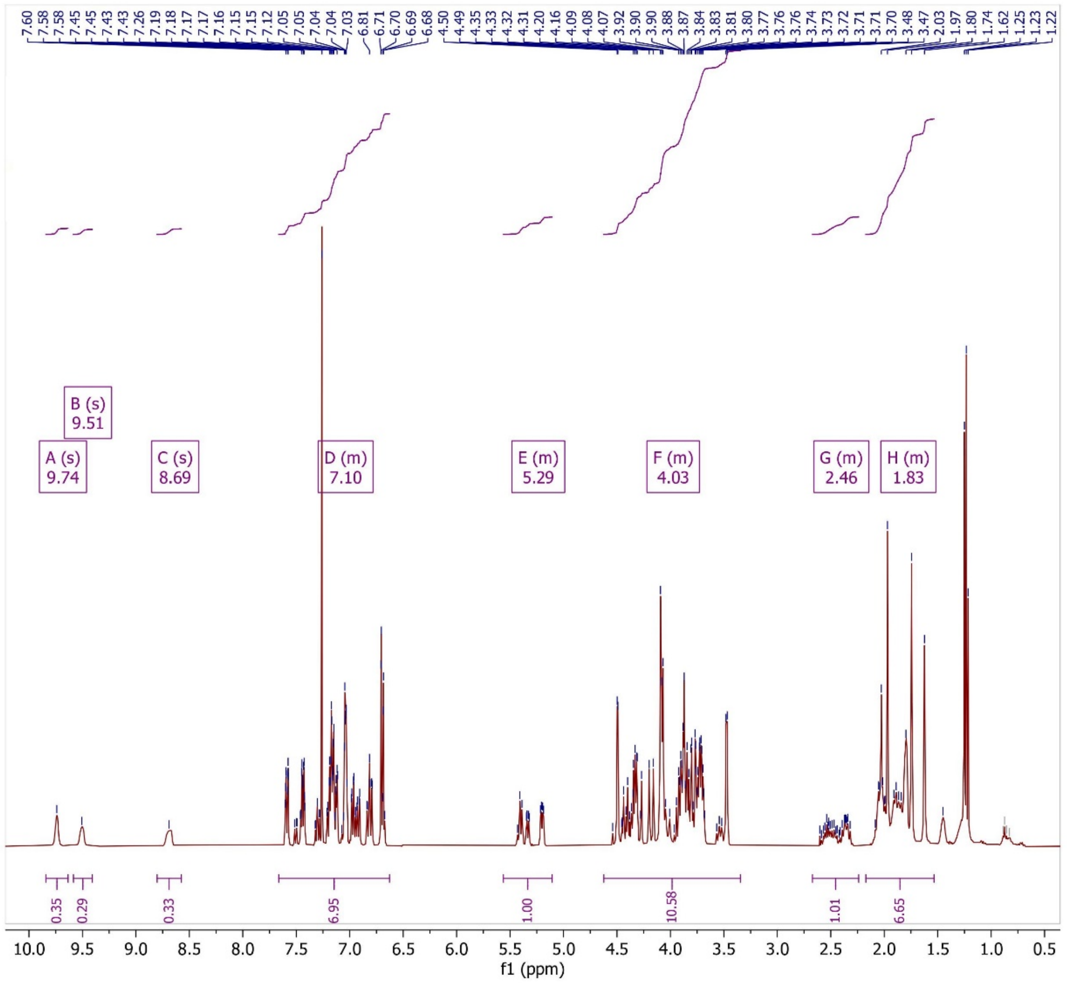

3-Chloro-5-[[(5-methyl-1,3,4-oxadiazol-2-yl)methylamino]methyl]pyridin-2-amine (22fd)

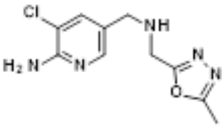

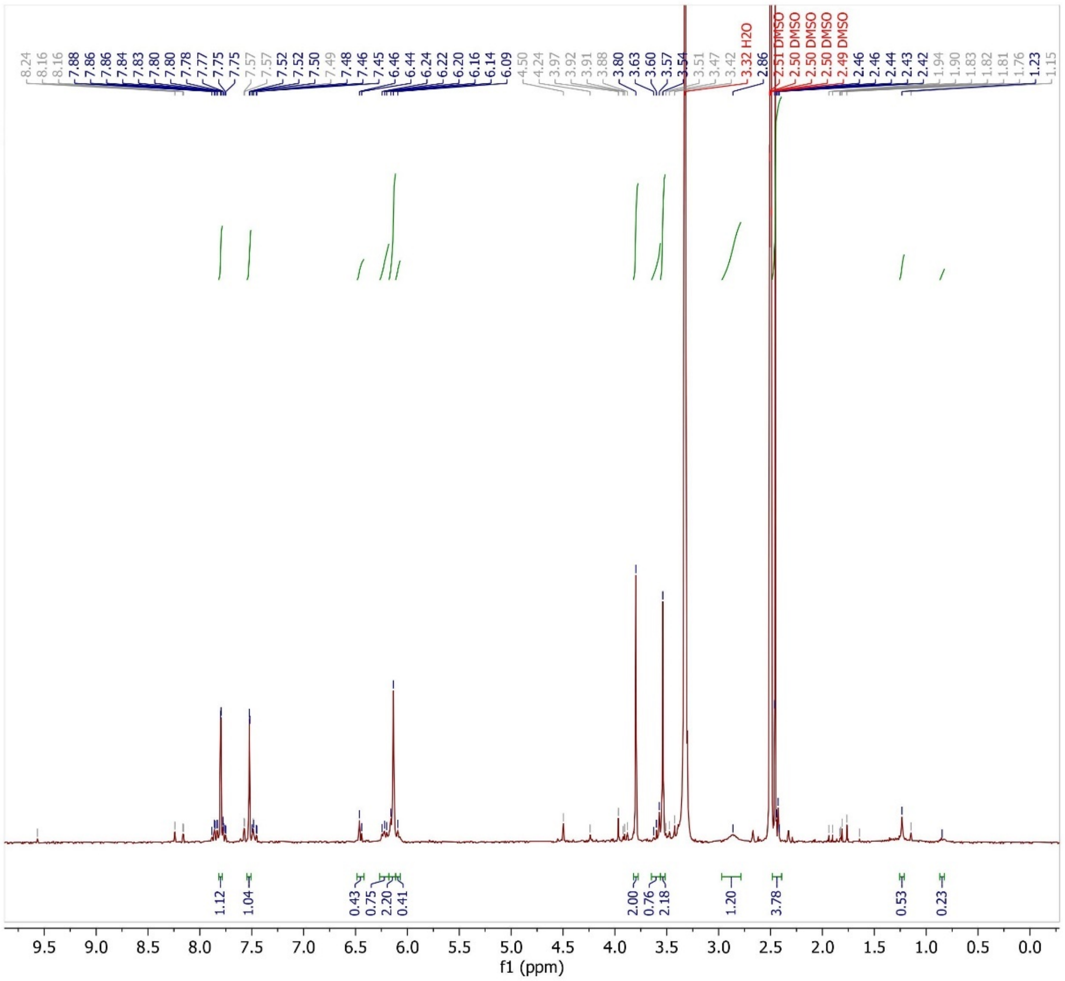

Ethyl 2-[[(6-amino-5-chloro-3-pyridyl)methyl-[(5-methyl-1,3,4-oxadiazol-2-yl)methyl] carbamoyl]amino]acetate (23fd)

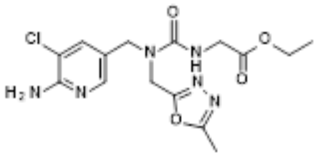

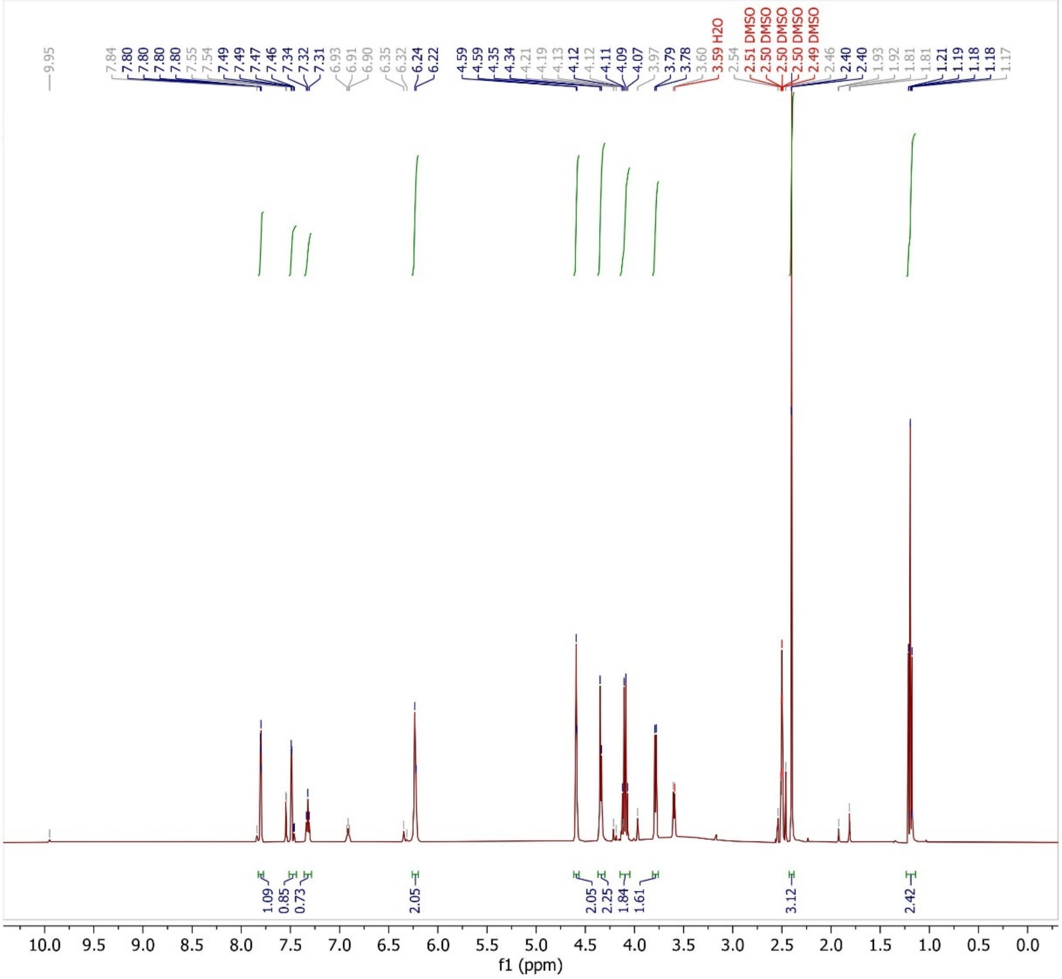

2-[5-(Acetylhydrazono)-3-[(6-amino-5-chloro-3-pyridyl)methyl]-2-oxo-imidazolidin-1-yl] acetic acid (31)

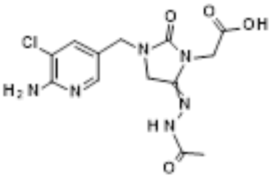

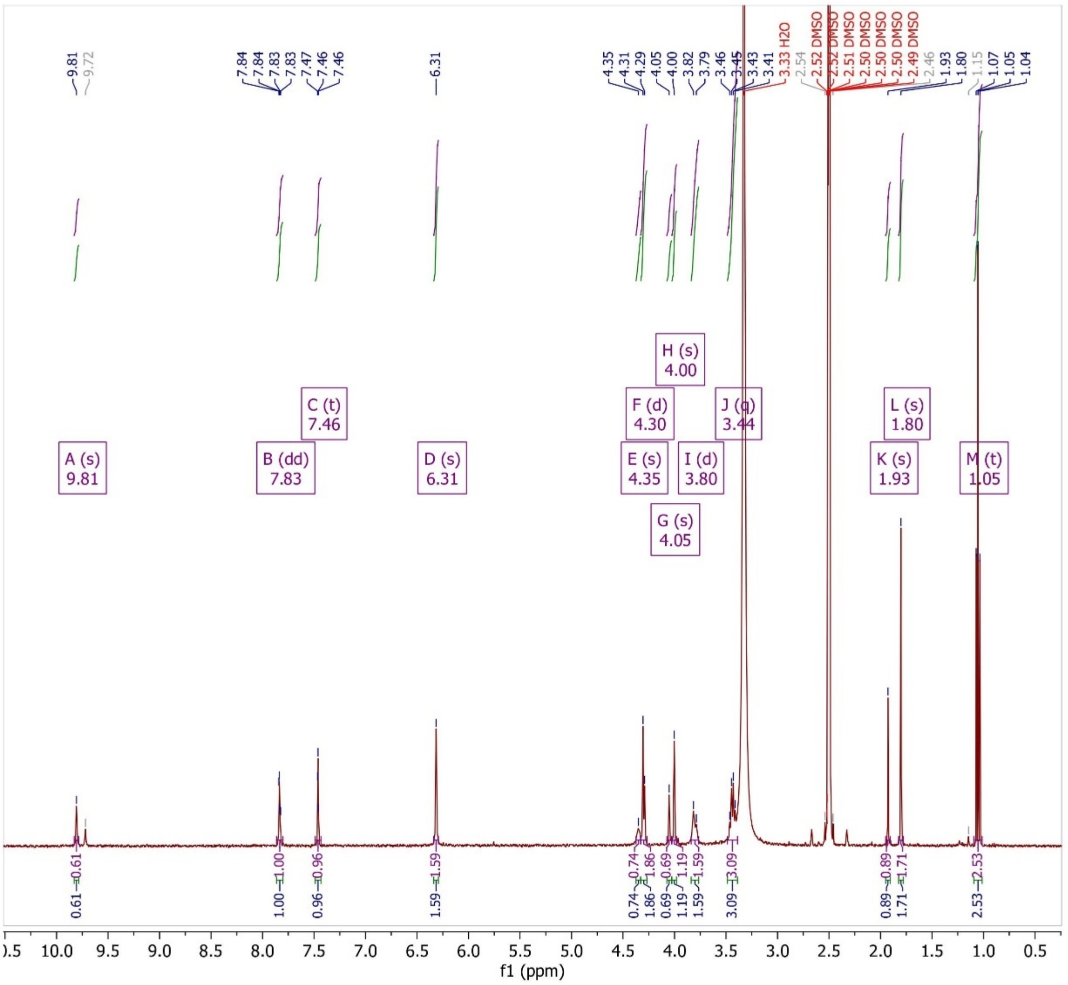

***N*-[[1-[(6-Amino-5-chloro-3-pyridyl)methyl]-3-[2-[(2R)-2-(2-methylsulfanylphenyl) pyrrolidin-1-yl]-2-oxo-ethyl]-2-oxo-imidazolidin-4-ylidene]amino]acetamide (18)**

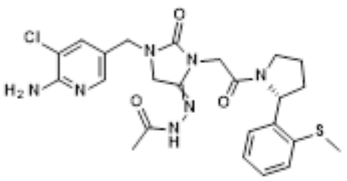

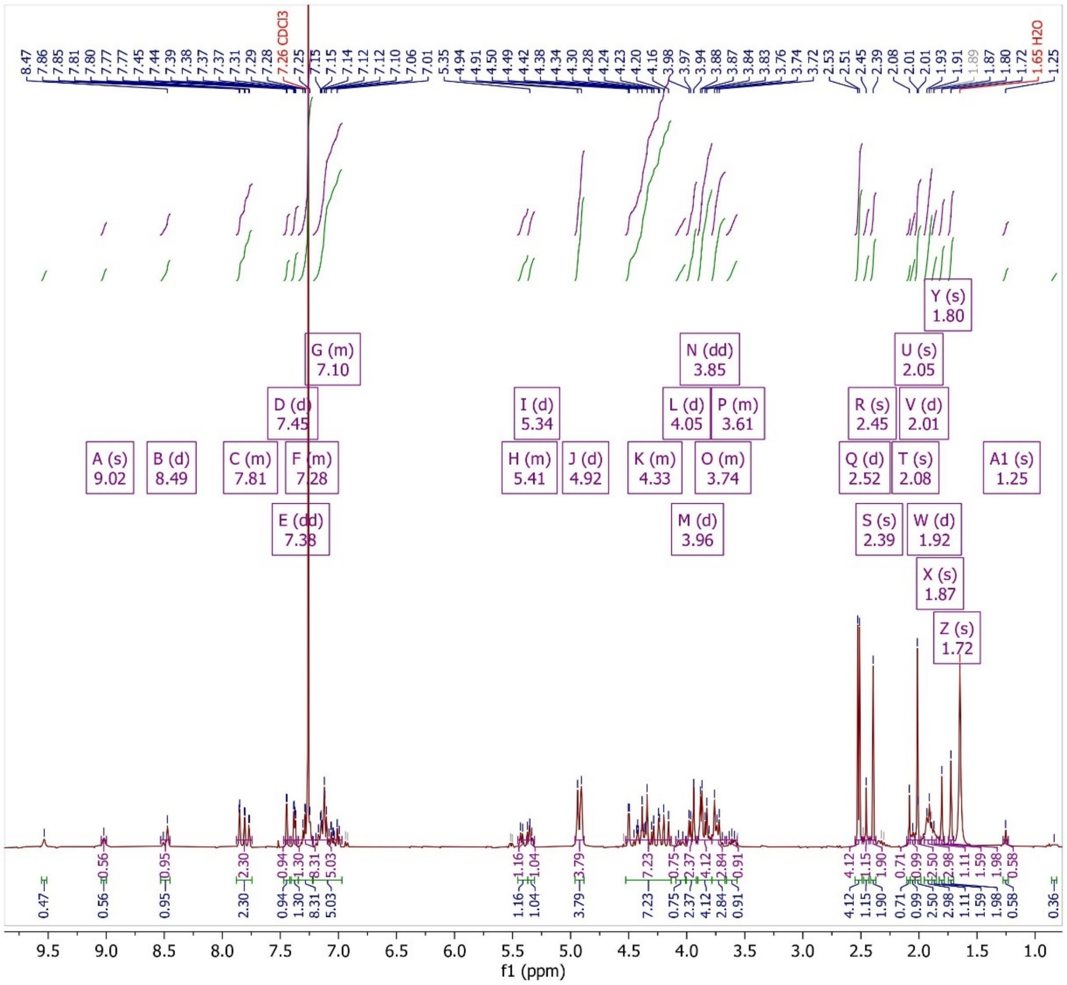

***N*-[[1-[(6-Amino-5-chloro-3-pyridyl)methyl]-2-oxo-3-[2-oxo-2-[(2*R*)-2-(2-bromophenyl) pyrrolidin-1-yl]ethyl]imidazolidin-4-ylidene]amino]acetamide (19)**

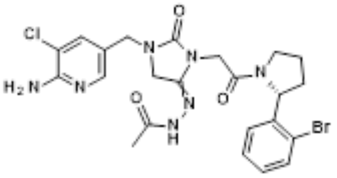

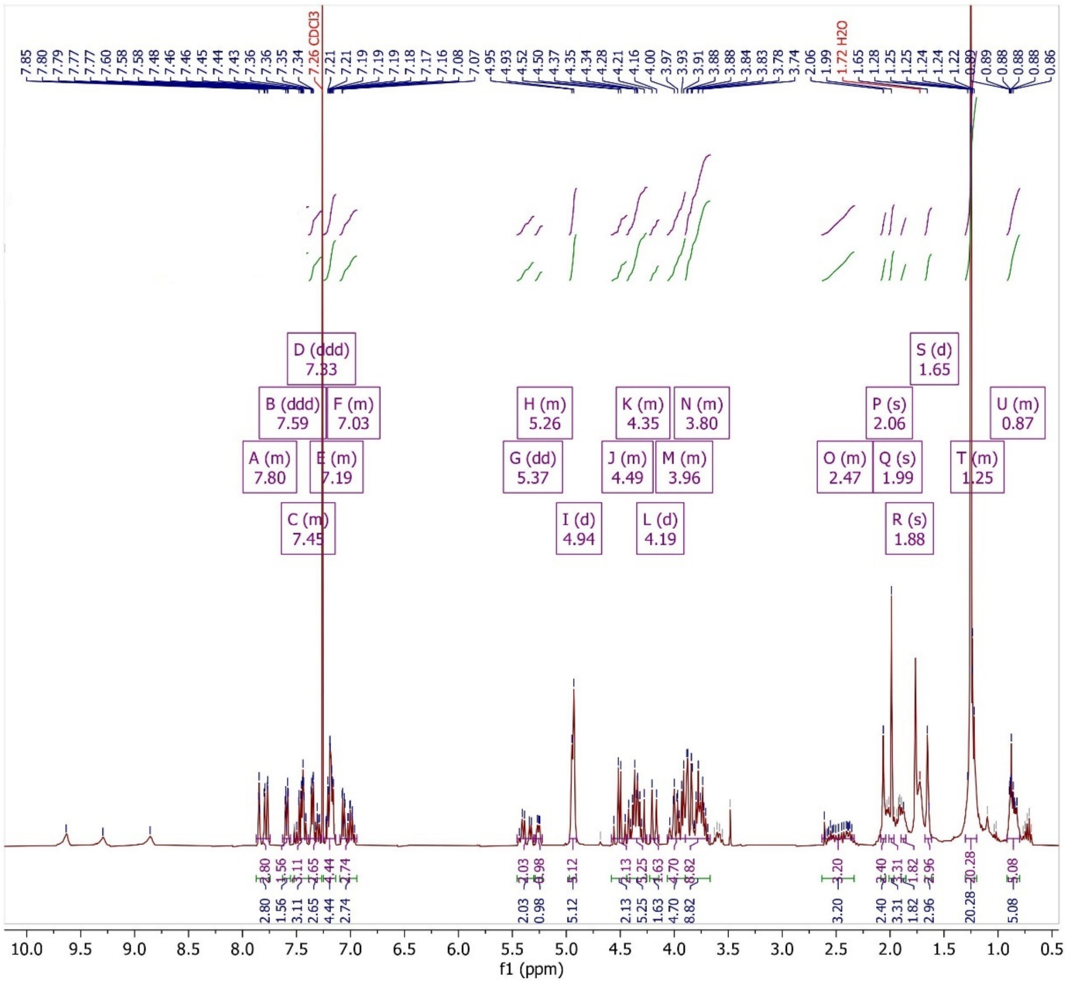

***tert*-Butyl *N*-[[(1*S*,9*R*,10*S*)-10-hydroxy-12-oxa-8-azatricyclo[7.3.1.0^{2},^{7}]trideca-2,4,6-trien-4-yl]methyl]carbamate (S1)**

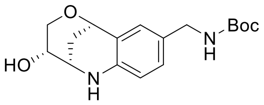

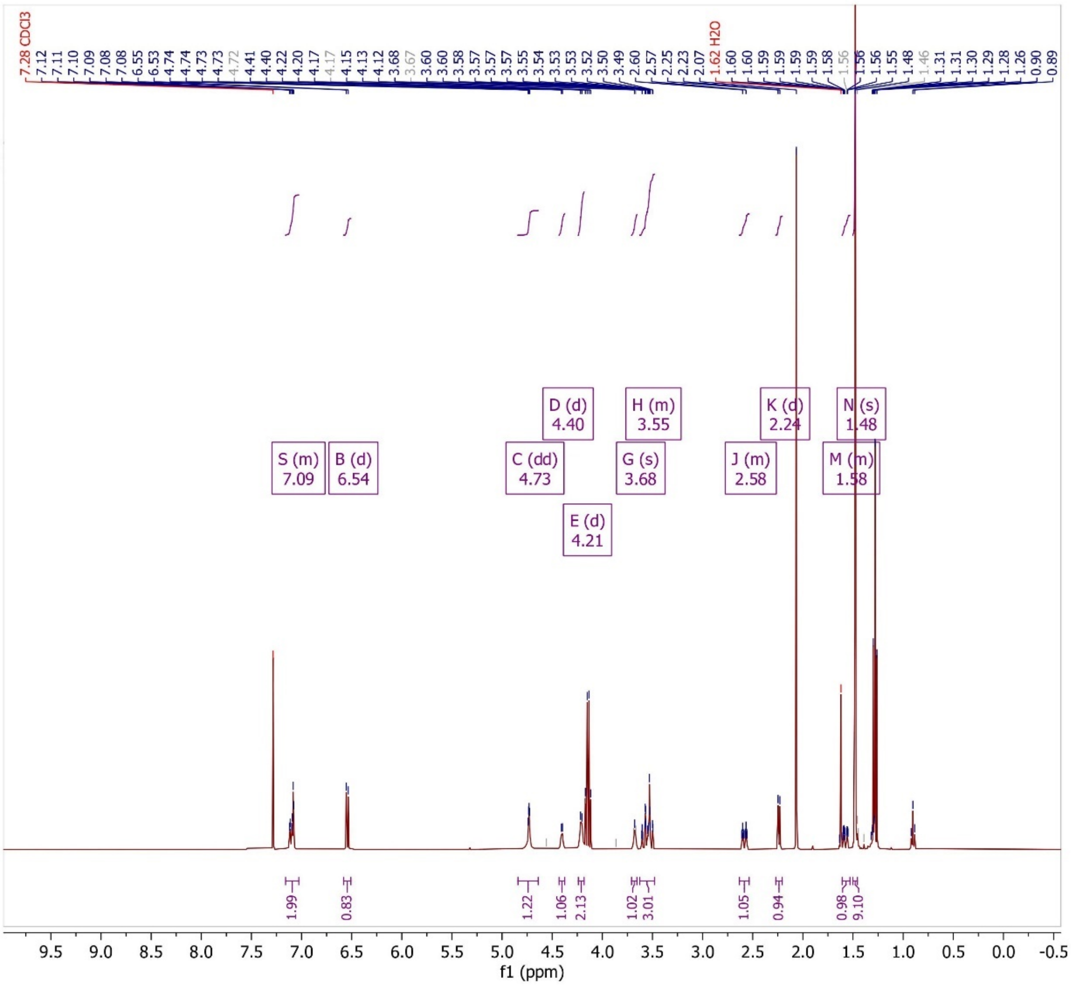

***tert*-Butyl *N*-[[(1*S*,9*R*,10*S*)-10-[*tert*-butyl(dimethyl)silyl]oxy-12-oxa-8-azatricyclo [7.3.1.0^{2,7}]trideca-2,4,6-trien-4-yl]methyl]carbamate (S2)**

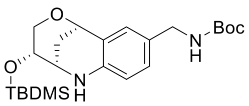

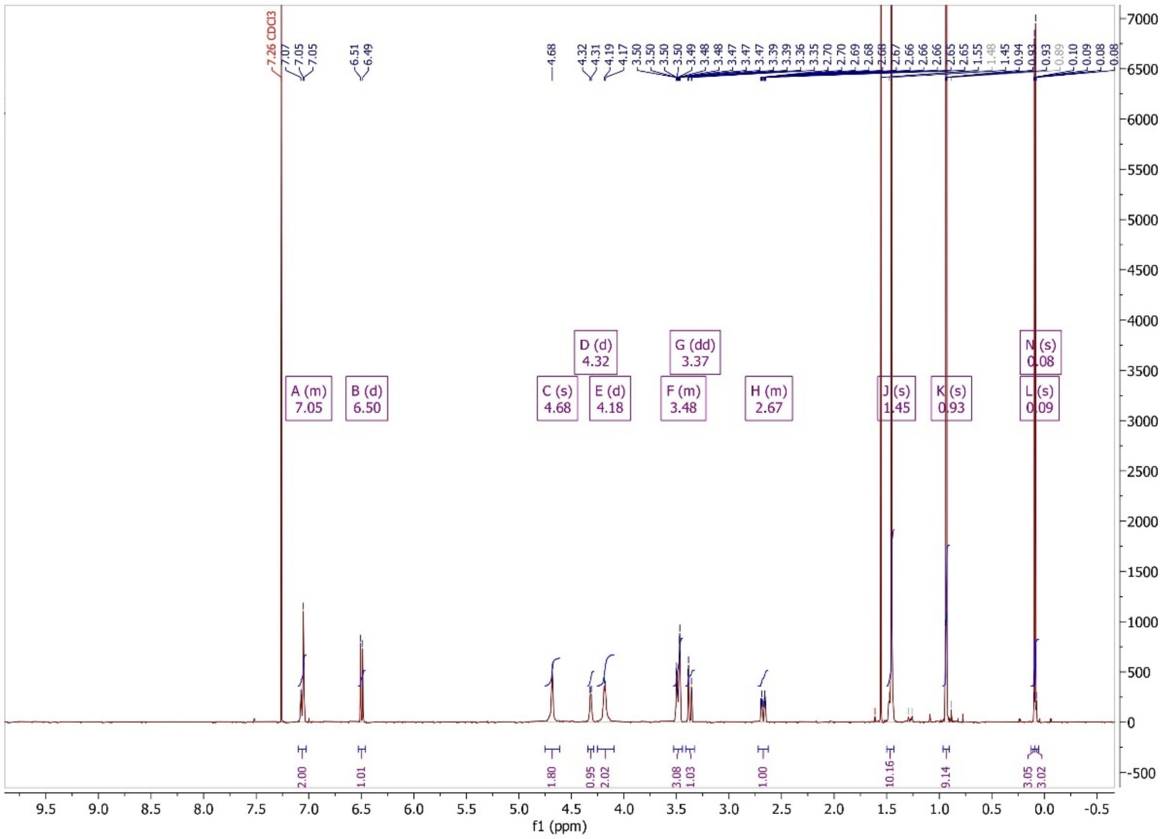

[(1*S*,9*R*,10*S*)-10-[*tert*-Butyl(dimethyl)silyl]oxy-12-oxa-8-azatricyclo[7.3.1.0^{2},^{7}]trideca-2,4,6-trien-4-yl]methanamine (20b)

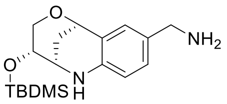

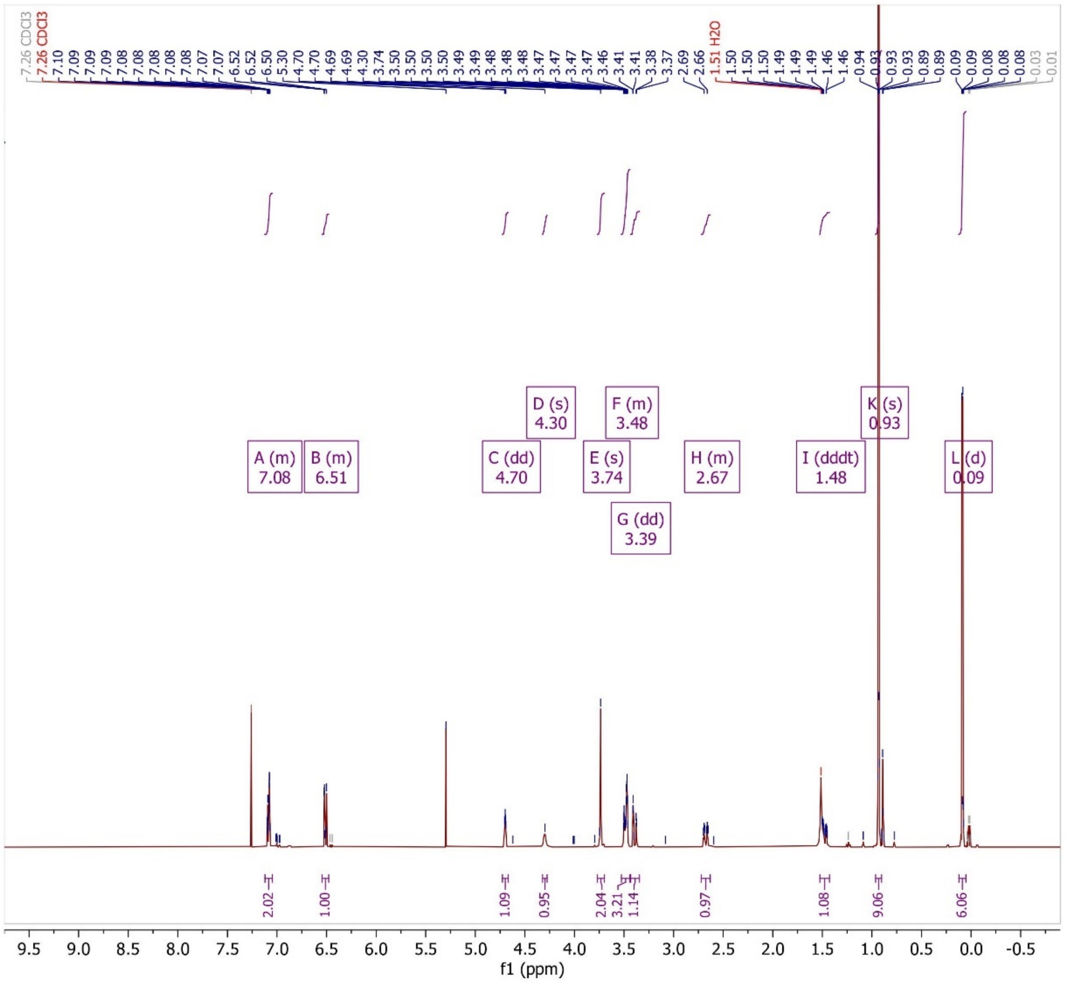

4-(Aminomethyl)-2,6-difluoro-aniline (20c)

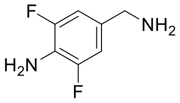

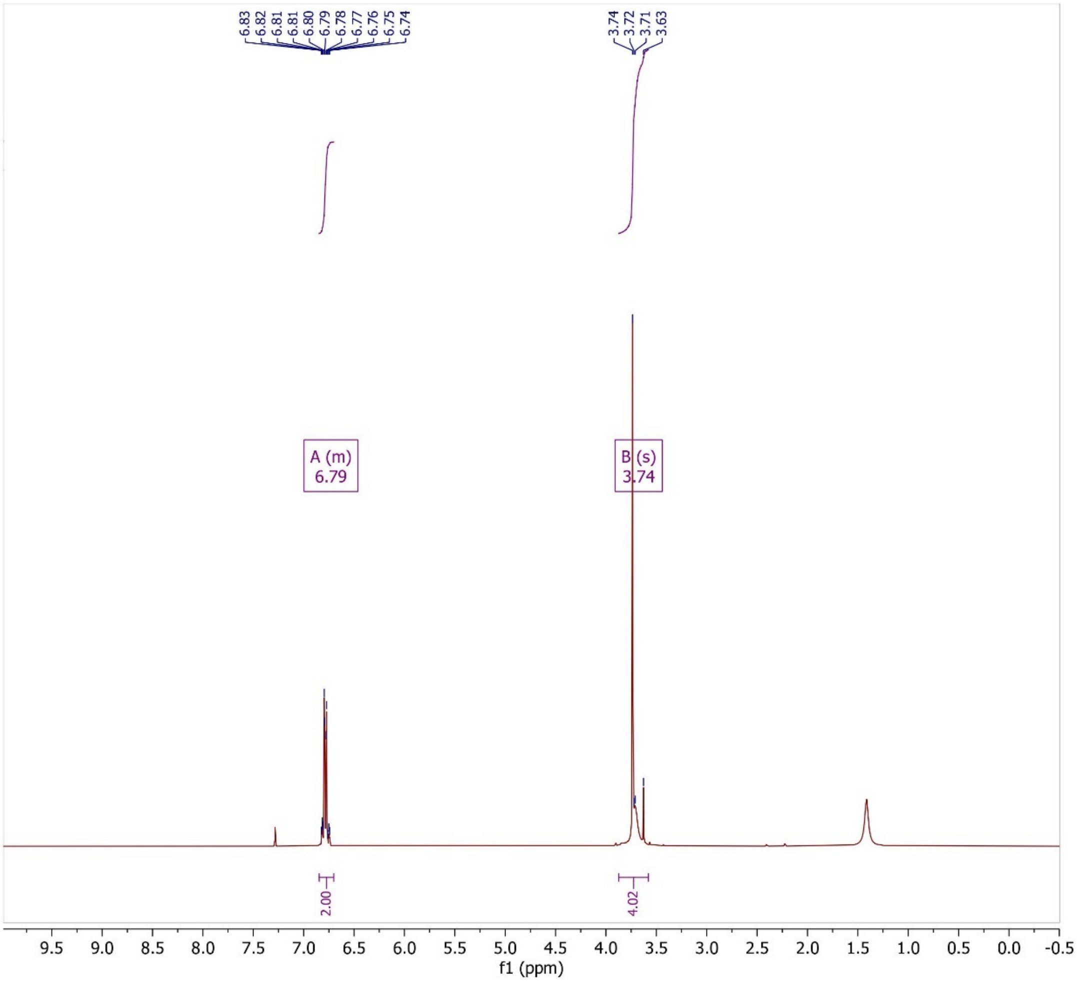

4-(Aminomethyl)-2-chloro-aniline (20d)

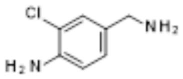

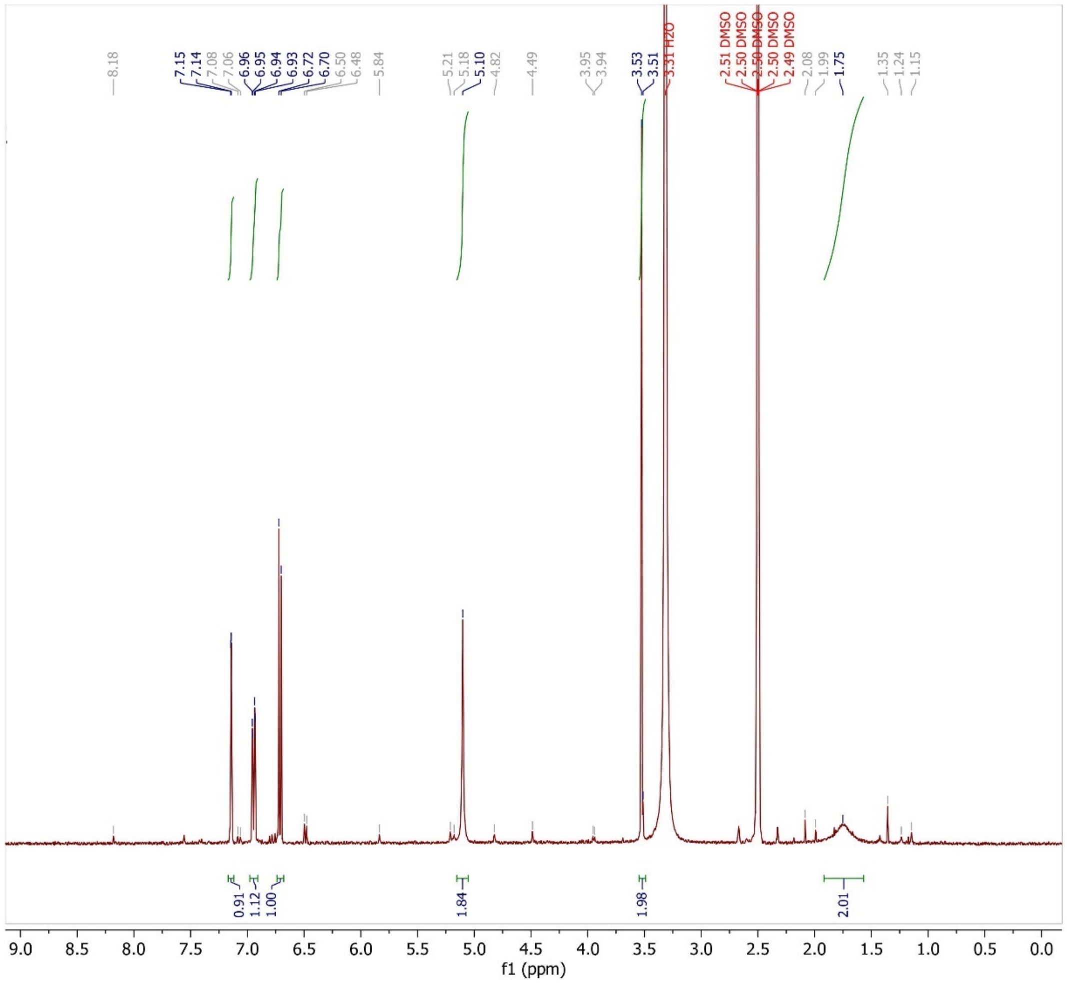

tert-Butyl _N_-_tert_-butoxycarbonyl-_N_-(5-cyanopyrimidin-2-yl)carbamate (S3)

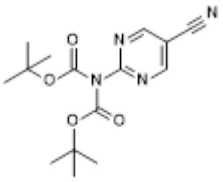

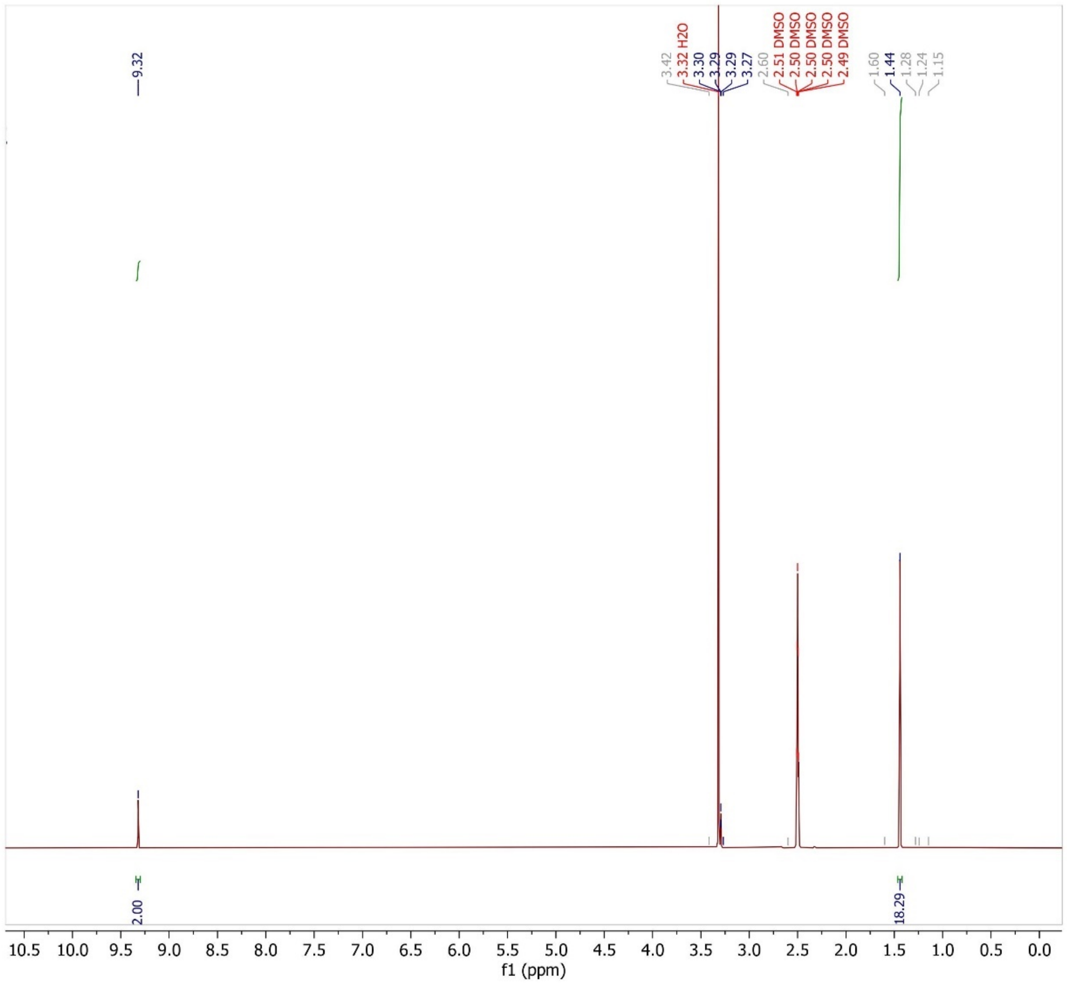

***tert*-Butyl *N*-[5-(aminomethyl)pyrimidin-2-yl]-*N*-*tert*-butoxycarbonyl-carbamate (20e)**

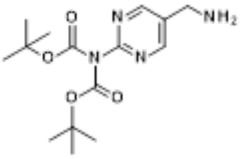

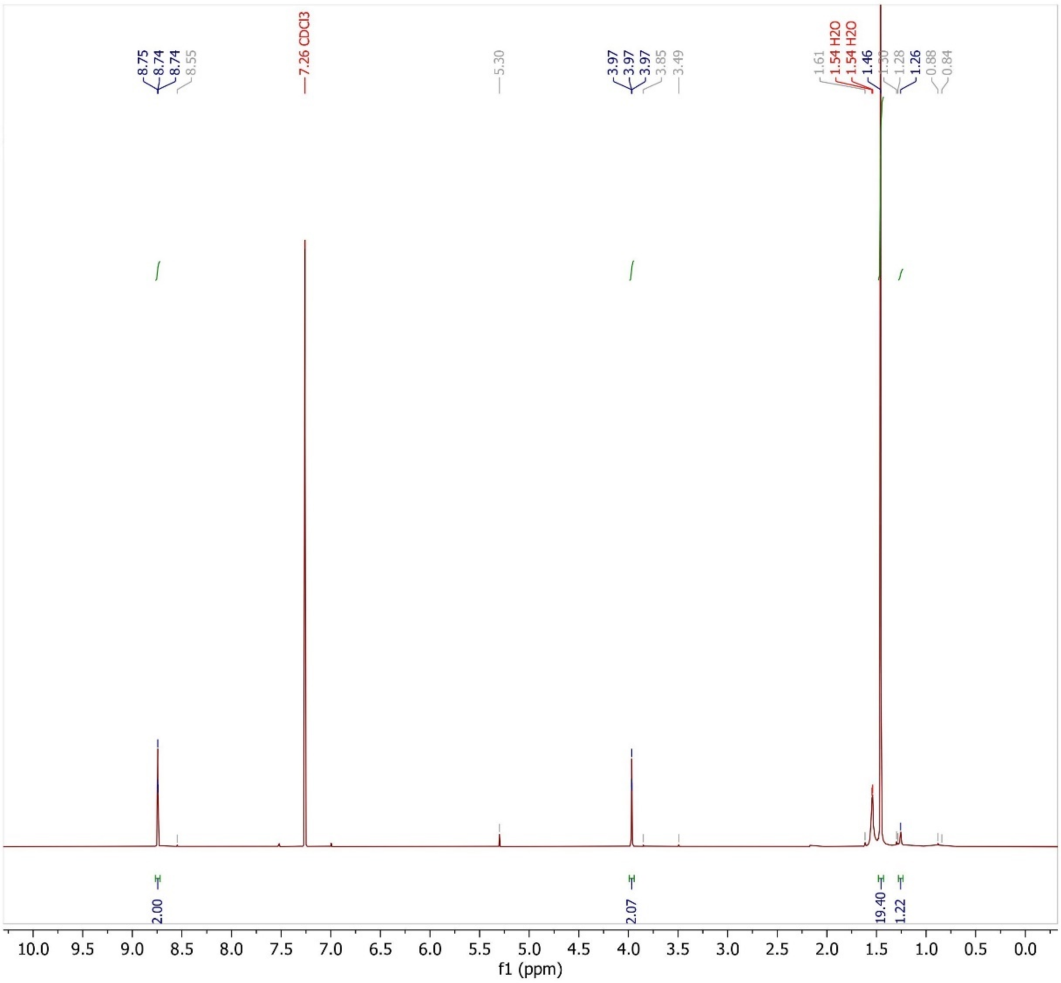

5-(Aminomethyl)-3-chloro-pyridin-2-amine (20f)

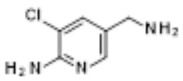

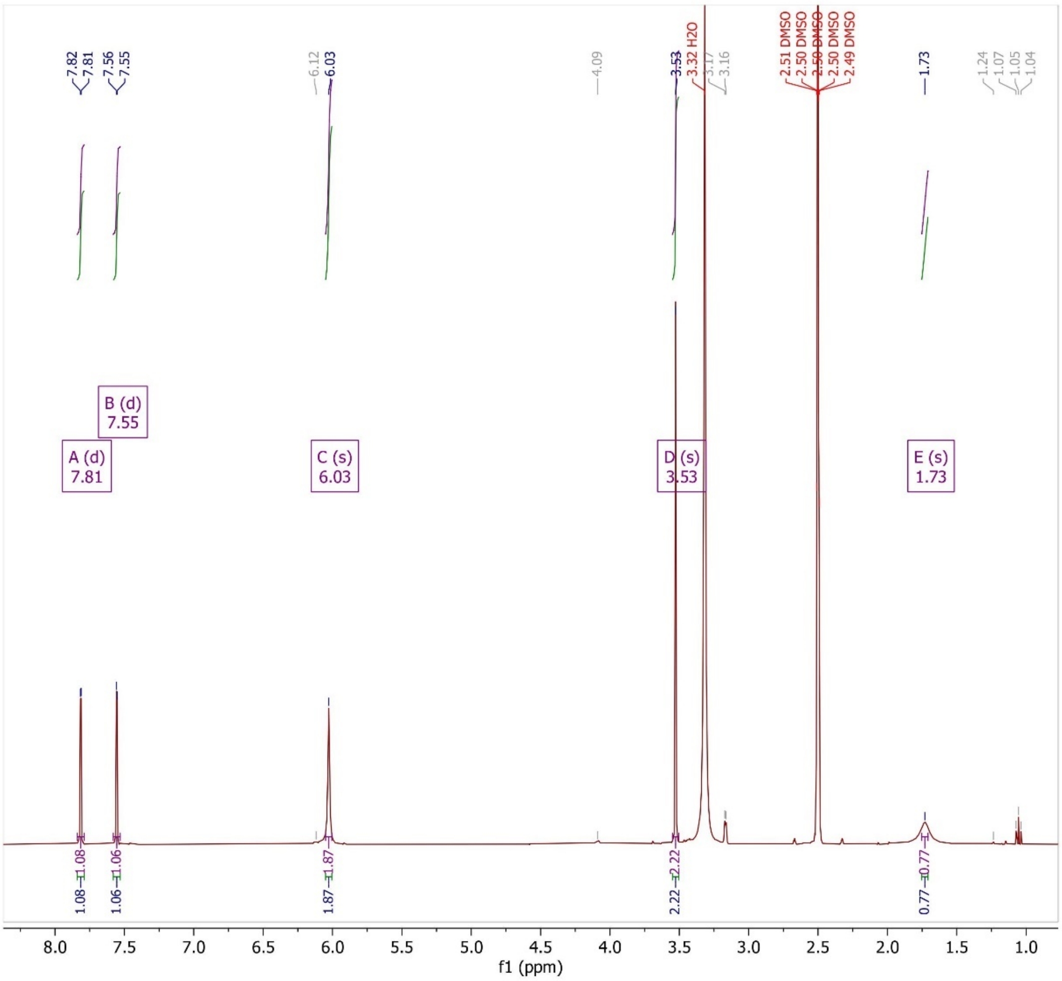

5-(Chloromethyl)-2-methyl-tetrazole (21a)

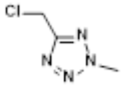

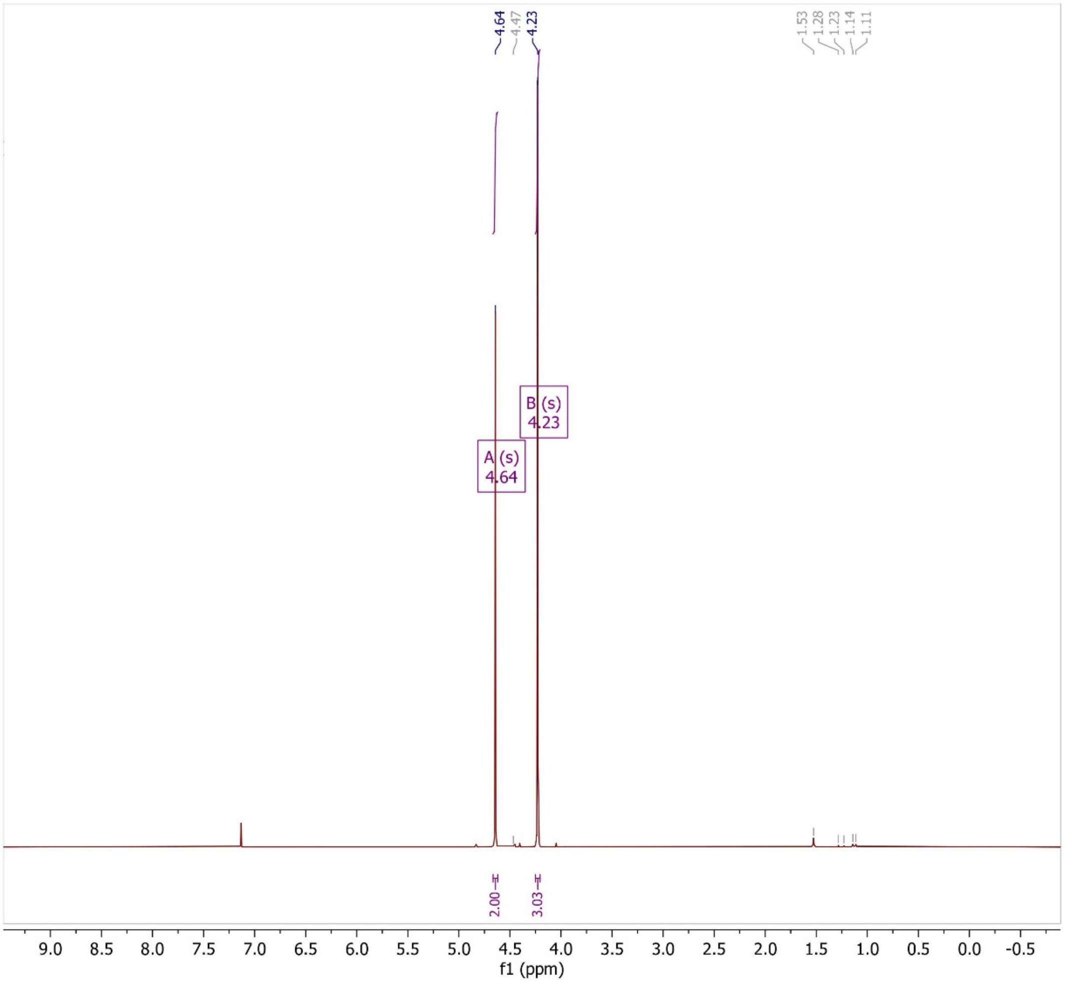

tert-Butyl N-[4-(2-methylsulfanylphenyl)-4-oxo-butyl]carbamate (S4)

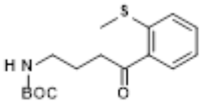

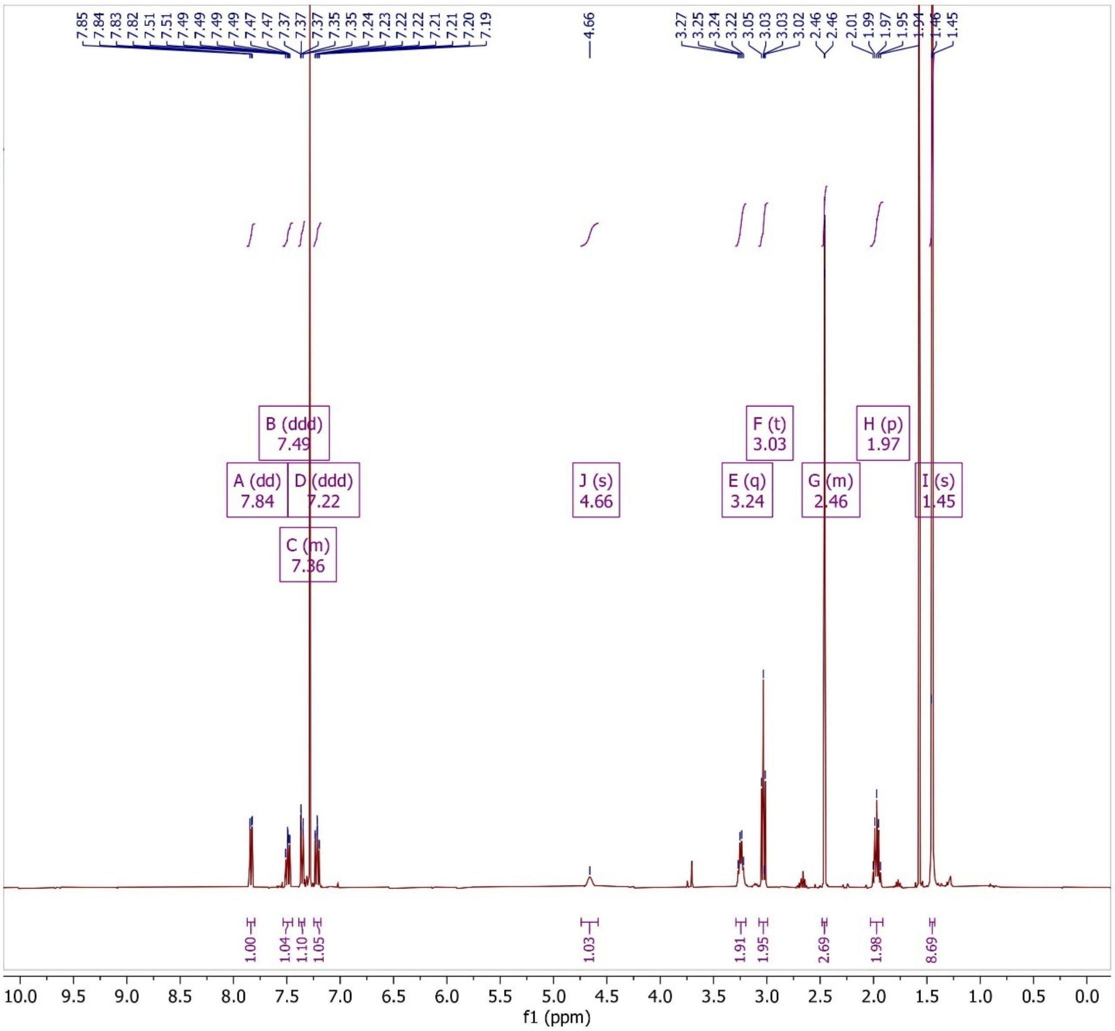

(*R*)-2-(2-Methylsulfanylphenyl)pyrrolidine ((*R*)-26)

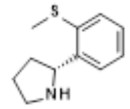

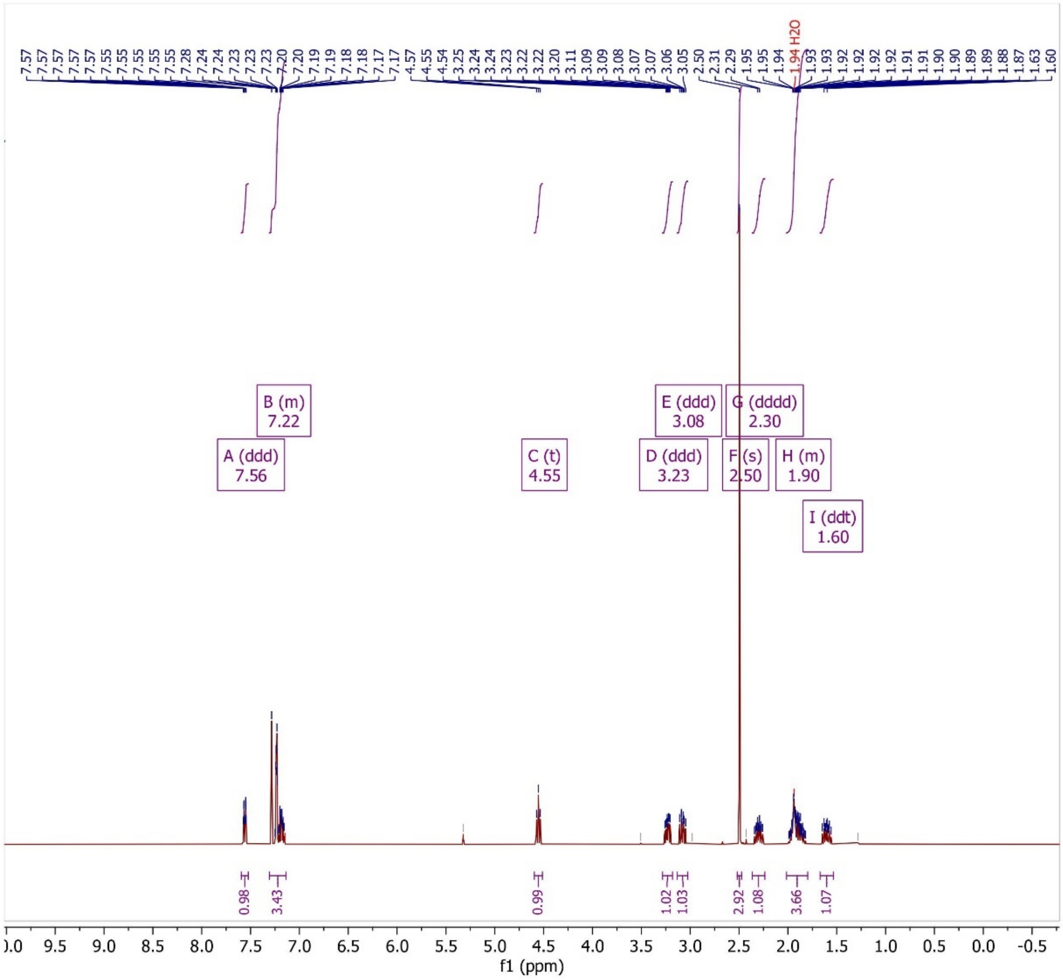

(*R*)-2-(2-Methylsulfanylphenyl)pyrrolidine ((*R*)-26)

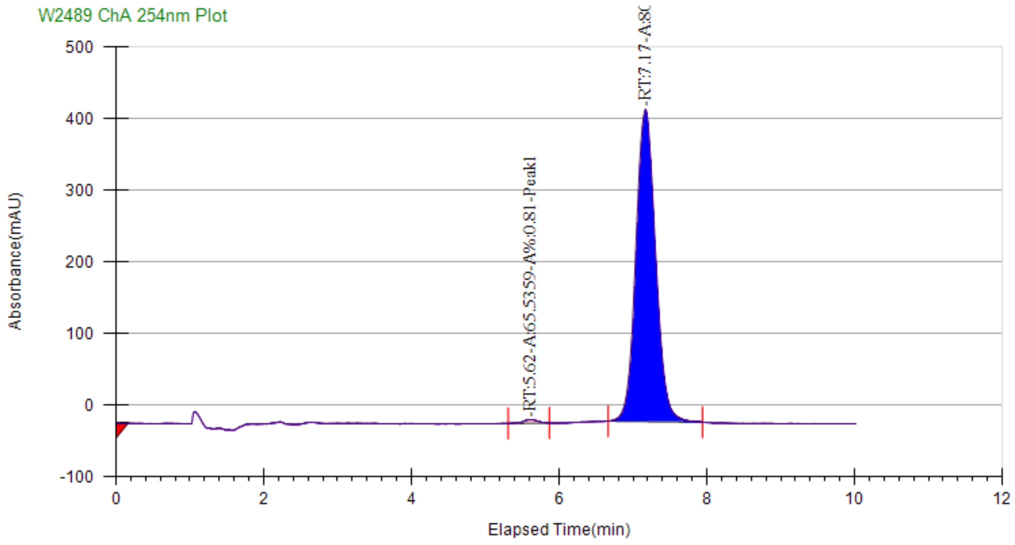

Run Information

Instrument Method	Inj. Vol.	Solvent	Column	Sample	Well Location	Temp	Flow	% Modifier	Pres sure
15_iso15_15min	150	Solvent6	AD-H 10x250mm	JG0054-40-batch1-peak2	13B	40	15	15	120

Peak Information

Peak No	% Area	Area	Ret. Time	Height	Cap. Factor
1	0.81	65.5359	5.62 min	4.9667	0
2	99.19	8025.526	7.17 min	435.8364	0

2-(2-Bromophenyl)-1-pyrroline (S5)

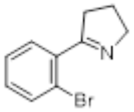

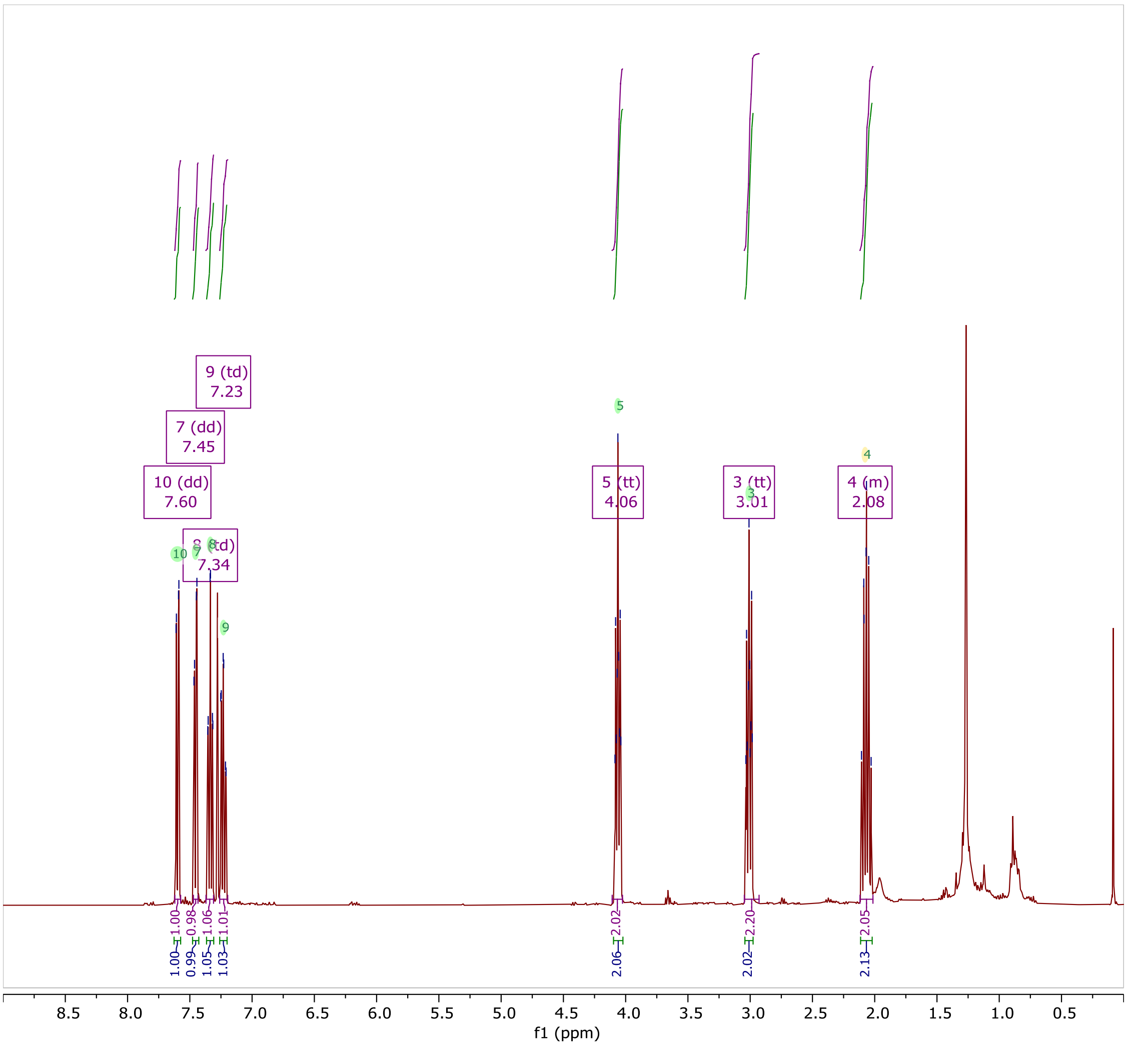

2-(2-Bromophenyl)-1-pyrrolidine (27)

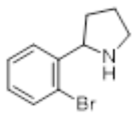

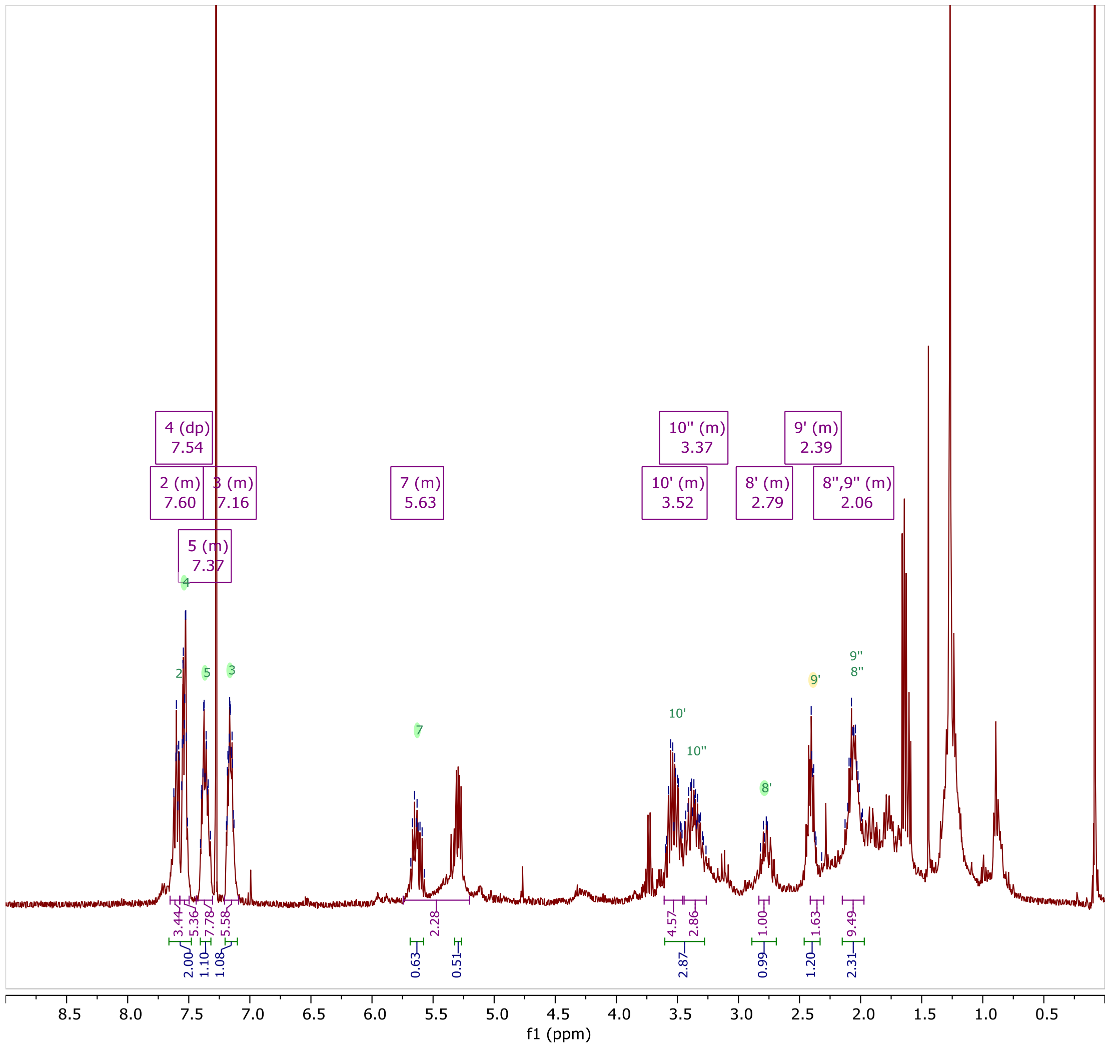

2-{[(4-Nitrophenyl)methyl]amino}acetonitrile (22af)

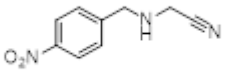

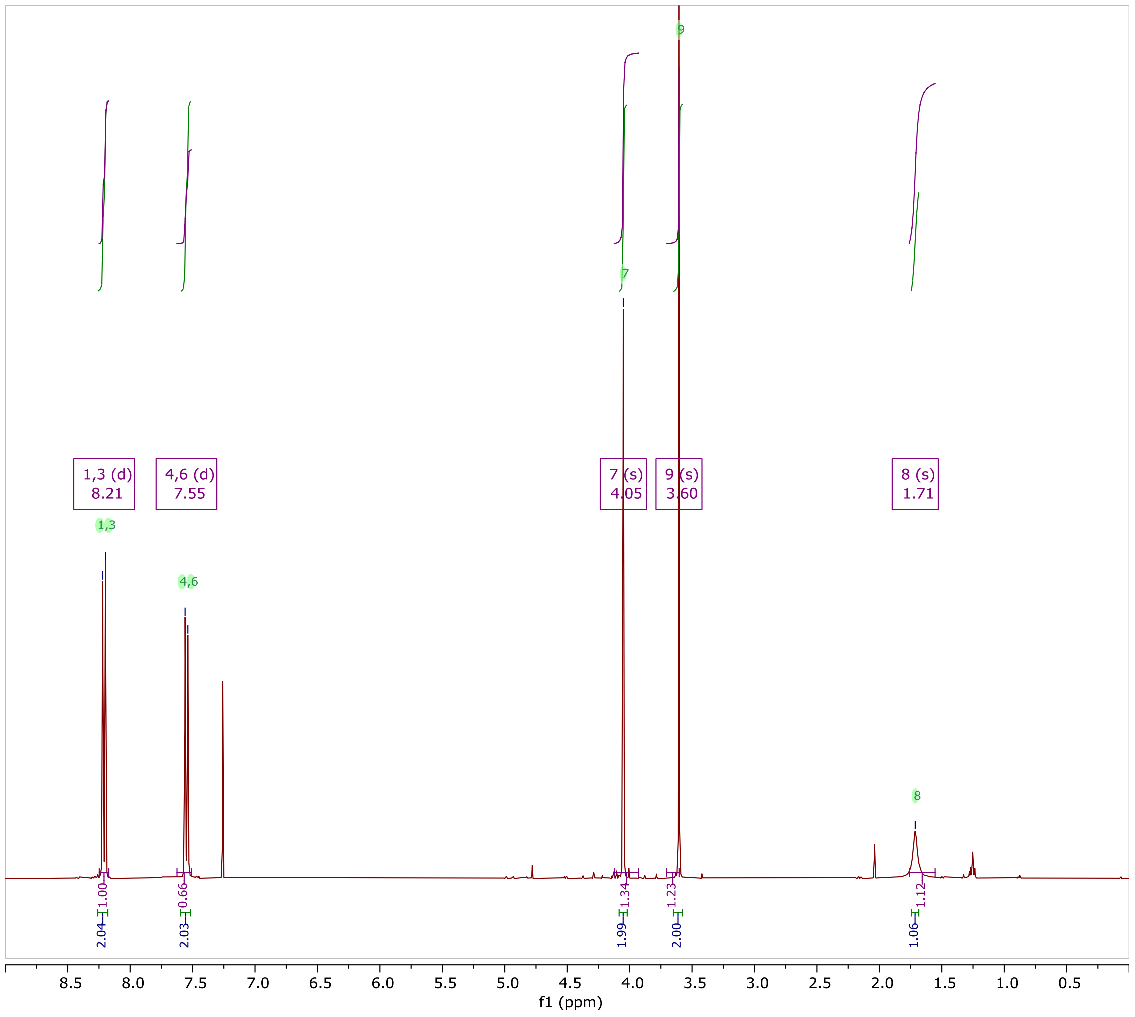

Ethyl 2-{[(cyanomethyl)[(4-nitrophenyl)methyl]carbamoyl]amino}acetate (23af)

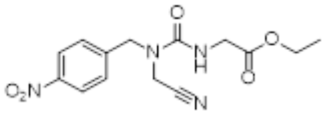

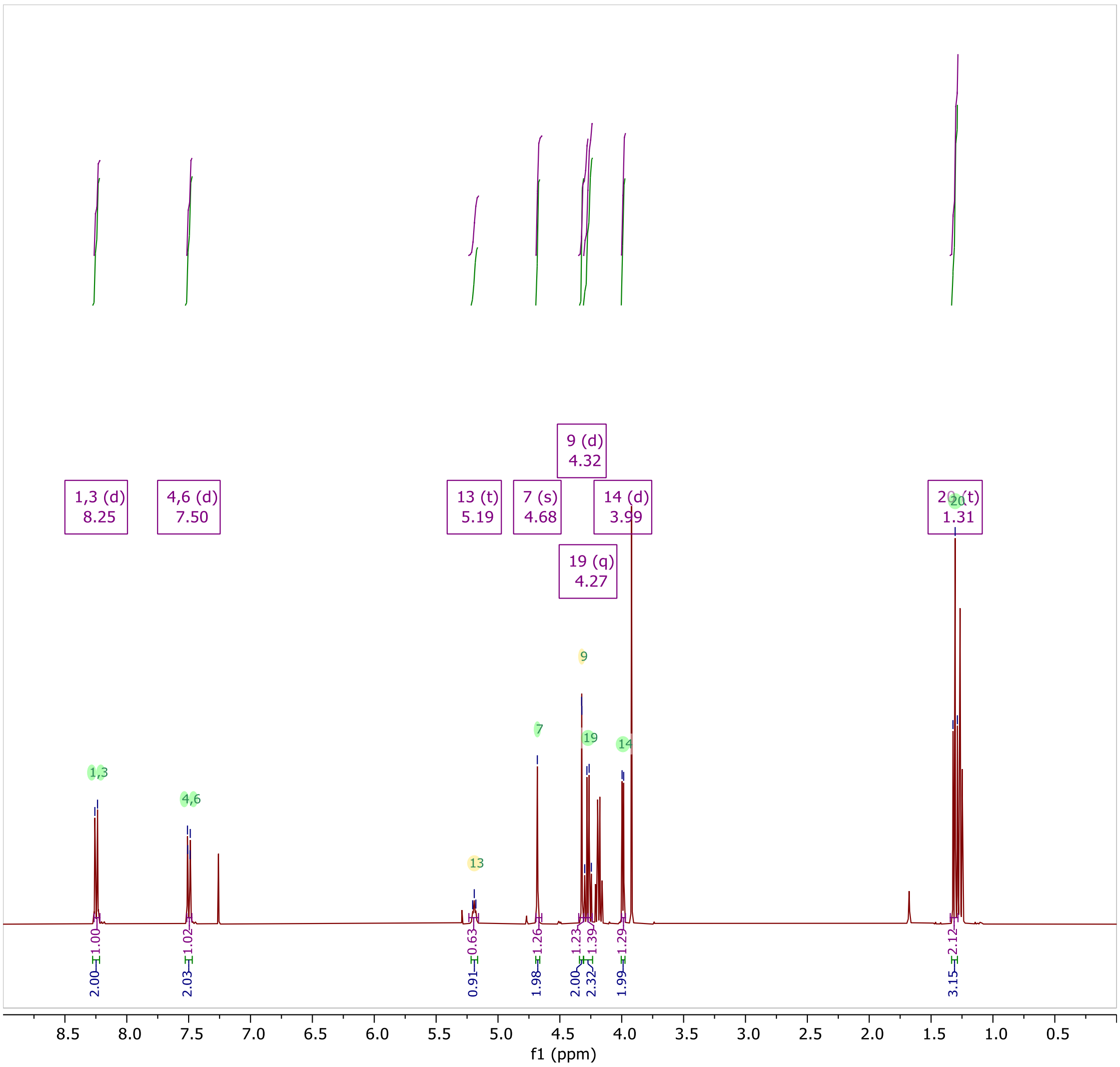

1-(4-nitrobenzyl)-2,4-dioxoimidazolidine-3-acetic acid (29)

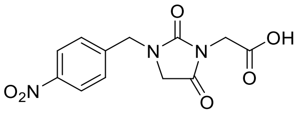

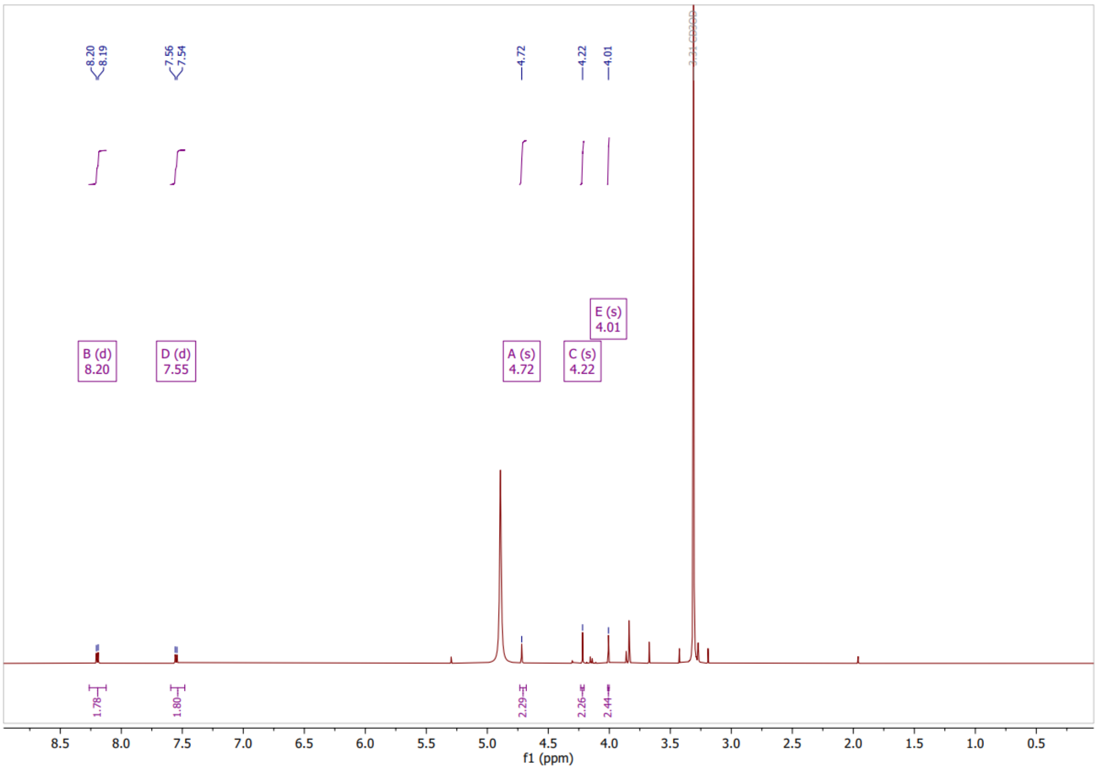

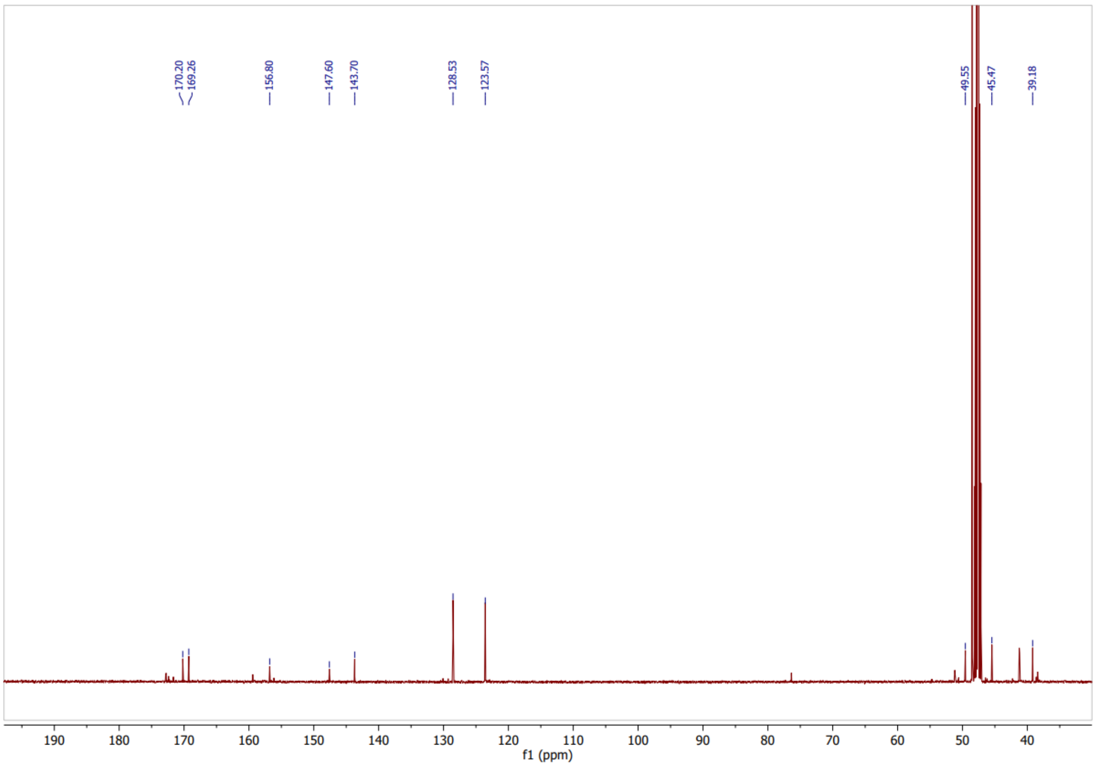

Biological experiments

Protein Production & Purification

All production and purification were performed as described in *Wear et al. **2017**. FEBS openbio. 7, 533-549*. The open reading frame encoding for full-length human CypA (M1 – E165), and CypB (S22-E216) and CypD (S42-S207) minus targeting sequences for ER and Mitochondria were synthesised and codon optimised (GENEART) for expression in Escherichia coli, with a hexa-his tag (underlined), linker and a TEV protease cleavage site (bold, underlined) fused to the N terminus (MSKYHHHHHHDYDIPTTENLYFQ/G-M-CypX). Standard GATEWAY® methodology was used to generate an expression vector in pDEST™14 (ThermoFisher, Waltham, MA, USA). Full open reading frame sequencing was used to verify coding sequence in final the expression vector. The His-CypB(22-216) construct does not have the entire signal sequence removed. This was because it was found that full truncation of this led to a drop in stable expression.

His-CypA(1-165): 21,002 Da. E = 13.200 M-1.cm-1. His-1-165 (ORF)
His-CypB(22-216): 23,552 Da. E = 21,430 M-1.cm-1. His-22-216 (ORF)
His-CypD(42-207): 20,938 Da. E = 14,690 M-1.cm-1. His-42-207 (ORF)

Recombinant proteins were overexpressed and purified to homogeneity from OverExpress C41 BL21(DE3) E. coli (Lucigen, Middleton, WI, USA), grown shaking (260 r.p.m.) at 30°C for 16 h in 50 mL of EnPresso media (BioSilta, St. Ives, Cambridgeshire, UK) containing carbenicillin (100 μg·mL^{-1}). Cell pellets were resuspended in 20 mm NaH_2PO_4, pH 7.4; 500 mm NaCl; 20 mm imidazole; plus protease inhibitors at 10% w/v, and lysed at 6°C by a single passage through a Constant Systems Cell Disruptor (1.1 kW TS Benchtop) set at 22 kpsi, followed by centrifugation at 50 000 g for 1 h at 4°C. The supernatant was filtered (0.22 μm) and then subsequently loaded onto an ÄKTAXpress™ (GE Healthcare, Little Chalfont, Buckinghamshire, UK) system fitted with 5 mL HiTrap Ni^{2+}-IMAC FF (GE Healthcare) and HiPrep S200 26/60 HR (GE Healthcare) columns, with standard configuration and peak-collection settings for a two-step affinity-gel-filtration protocol (with the exception of a 13 mL loop flush from a 10 mL loop, after loading 8 mL from the IMAC elution). A single 10-column volume step to 100% IMAC Elution buffer was used for elution from the IMAC column. Buffers used for the purification were; IMAC Loading buffer: 20 mm NaH_2PO_4, pH 7.4; 500 mm NaCl; 20 mm Imidazole; 100 μmPMSF. IMAC Elution buffer: 20 mm NaH_2PO_4, pH 7.4; 500 mm NaCl; 500 mm Imidazole. Gel-Filtration Buffer: 10 mm NaH_2PO_4, pH 7.5; 150 mm NaCl; 50 μm EDTA. Each isoform was concentrated to 100 μM (using the extinction

coefficient listed above) and stored at 4°C in Gel-Filtration Buffer until use. Representative gels for HisCypA are depicted in **Figure S2**.

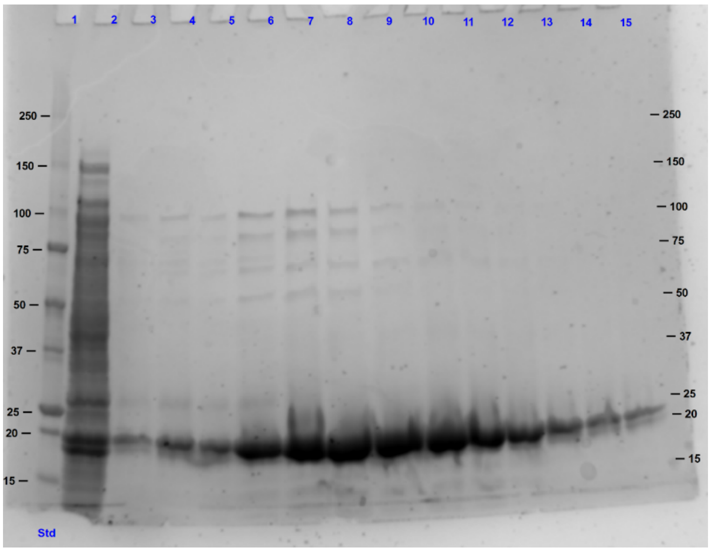

Figure S2. HisCypA purification on polyacrylamide gel

Peptidyl prolyl isomerase assay

This assay determines the rate of the cis to trans conversion of the peptidyl-prolyl amide bond in the substrate N-succinyl-Ala-Ala-Pro-Phe-p-nitroanilide (AAPF-pNA). Selective hydrolysis of AAtransPF-pNA by α-chymotrypsin releases p-ntitroaniline, the accumulation of which is monitored by absorbance at 400 nm. AAPF-pNA, in 470 mm LiCl; 2,2,2-trifluroethanol at 200 mm, was diluted to 4 mm in the same buffer immediately before use. Reactions were conducted at 12°C on a Jasco V550 spectrophotometer with temperature control, in 50 mm HEPES, pH 8.0; 100 mm NaCl; 0.5 mm DTT, in a total volume of 1 mL, essentially as described (Kofron *et al.* Biochemistry *30, 6127–6134,* ***1991*** *;* Wear *et al.* Anal Biochem *345, 214–226,* ***2005*** *;* Husi and Zurini Anal Biochem *222, 251–255,* ***1994)*** with minor modifications. The final concentration of His-CypX and AAPF-pNA were 12.3 nM and 100 µM respectively. The apparent equilibrium dissociation constant, K_{iapp}, for the inhibitor was determined by least squares fit to plots of the initial reaction rate (background thermal isomerisation rate subtracted), V_0 (in $\mu m \cdot s^{-1}$) versus the concentration of CsA in nM. Correction for competition with sAAcisPF-pNA substrate was performed with initial AAcisPF-pNA concentration.

The catalytic activity, inhibition constants by CsA and comparison to binding constants measured by SPR for the isoforms are given below:

Isoform	Seg start/Mr. (Da)	k_{cat}/K_M ($M^{-1}.s^{-1}$)	K_I CsA (nM)	K_{DSPR} (nM)
CypA/PPIA	Tag*-M1-165/PPIA/21,002	8.1×10^6	15±2	21±4
CypB/PPIB	Tag*-S22/PPIB/23,552	1.3×10^7	11±3	10±1
CypD/PPIF	Tag*-S43/PPIF/20,938	9.0×10^6	14±3	14±5

HisTag cleavage

Protein His-Tag on CypA was cleaved for further use of the protein in ITC studies. Proteins were desalted to cleavage buffer (100 mM Tris, 100 mM NaCl, pH 7.5) using a HiPrep 26/10 desalting column prior to the addition of TEV protease (200 ng TEV / 40 µg protein). Samples were left incubating at 30°C for about 4 h and the cleaved His-tag was removed by IMAC. At the end of each purification the purity of the fractions was tested by using precast gels (Biorad®) in Tris/Glycine/SDS, pH 8.3 buffer.

The molecular weights of HisCypA and free CypA are 20.893 and 18.070 kDa, respectively. Protein concentration was determined by measuring the absorbance at 280 nm and the extinction coefficients 14440 and 8480 M^{-1} cm^{-1}, respectively.

Isothermal titration calorimetry (ITC)

All ITC experiments were carried out at 25°C on a MicroCal Auto iTC200 (GE Healthcare) instrument. The buffer used in the titrations of the compounds was 50 mM phosphate buffer, pH 6.5 and the concentration of DMSO was 2% v/v for all the compounds. Final compound solutions were heated to 65°C and/or sonicated prior to the experiment. Each experiment consisted of an initial injection of 0.4 µL followed by repeated 2 µL injections to achieve saturation. Representative thermograms are shown in **Figure S3**. In all cases the first injection was omitted from the data processing. All data were analysed using the MicroCal PEAQ-ITC Analysis software. A fixed stoichiometry to 1 was applied during the non-linear regression of the raw data for fitting the data. Thermodynamic parameters are provided in **Table S6**.

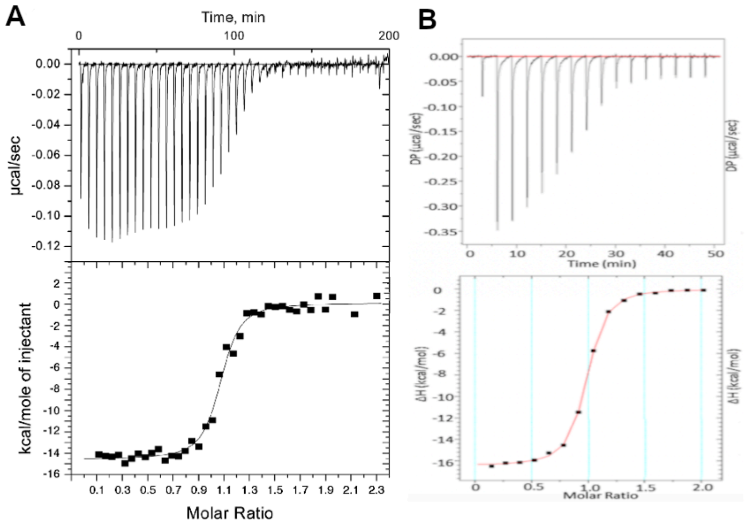

Figure S3. A) Representative ITC thermogram for titration of **CsA** in Cyp A. **B)** Representative ITC thermogram for titration of **11** in Cyp A.

Table S6. Thermodynamic parameters derived from ITC titrations of **CsA** and **11** to Cyp A.

	Kd / nM	ΔG / kcal.mol^{-1}	ΔH / kcal.mol^{-1}	TΔS / kcal.mol^{-1}
**CsA**	32±20	-10.3±0.1	-14.6±0.1	-4.3
**11**	82±6	-9.7±0.1	-16.4±0.1	-6.7

Surface Plasmon Resonance (SPR)

SPR measurements were performed on a Biacore T200 instrument (Cytiva). Ni^{2+} -nitrilotriacetic acid (NTA) sensor chips, 1-ethyl-3-(3-diaminopropyl) carbodiimide hydrochloride (EDC) and N hydroxy succinimide (NHS) were purchased from Cytiva. Immobilization and covalent stabilization of His-Cyps Pure His-cyclophilins on the NTA sensor chip was performed essentially as described. [Wear *et al*., 2017, FEBS OpenBio], using 200 nM concentrations of each protein, in *Running Buffer* (PBS, pH7.4; 0.05 % surfactant P20, 2% v/v ethanol; 50 µM EDTA), at 30 µl min^{-1} with 60 second contact times on the activated NTA surfaces. This gave signals of 1,921 RU for His-CypA, 1932 RU for His-CypB and 1,397 RU for His-CypD. Specific surface protein activity was assayed by passing saturating amounts of CsA (2 µM) in Running Buffer over these surfaces; values of 94.1%, 95.5% and 95.6% activity were obtained for His-CypA, -B and –D, respectively.

Kinetic titration experiments

CsA:

Single cycle kinetic titration binding experiments were performed in triplicate at 25˚C. 3-fold dilution concentration series of CsA, ranging from 2.45 nM to 200 nM, in *Running Buffer*, were injected over the sensor surface, at 100 µl min^{-1} with a 90 s contact time and a 90 s dissociation time. The sensor surface was regenerated between experiments by dissociating any formed complex in running buffer for at least 1,200 seconds. The apparent on-rate (k+) and off-rate (k-) constants and the equilibrium dissociation constant (K_d) were calculated from reference corrected sensorgrams by global fitting of a 1:1 binding model, including a mass transport term, using analysis software (v.2.02, Cytiva) provided with the Biacore T200 instrument.

*Assays of compounds **1–19**:*

Kinetic titration binding experiments were performed in triplicate at 25˚C. 2-fold dilution concentration series of the compounds, ranging from 0.0195 µM to 20 µM, in *Running Buffer*, were injected over the sensor surface, at 100 µl min^{-1} with a 15 s contact time and a 600 s dissociation time. The sensor surface was regenerated between experiments by dissociating any formed complex in running buffer for at least a further 600 seconds.

We verified lack of compound binding to the chip surface by monitoring response on the first flow-cell, a representative sensorgram is shown in **Figure S4**.

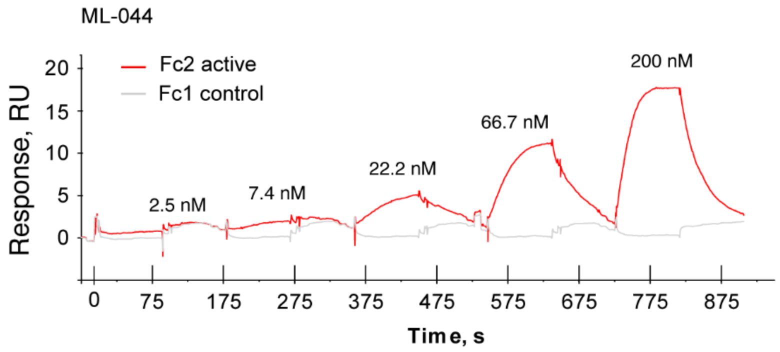

Figure S4. Representative SPR trace of Cyp A/**1** on control flow cell 1 and active flow cell 2.

The apparent on-rate (k+) and off-rate (k-) constants and the equilibrium dissociation constant (K_d) were calculated from reference corrected sensorgrams by global fitting of a 1:1 binding model, including a mass transport term, using analysis software (v.2.02, GE Healthcare) provided with the BIAcore T200 instrument. Typical results are shown for **1** in respective panels in **Figure S5** for His-CypA, -CypB and -CypD.

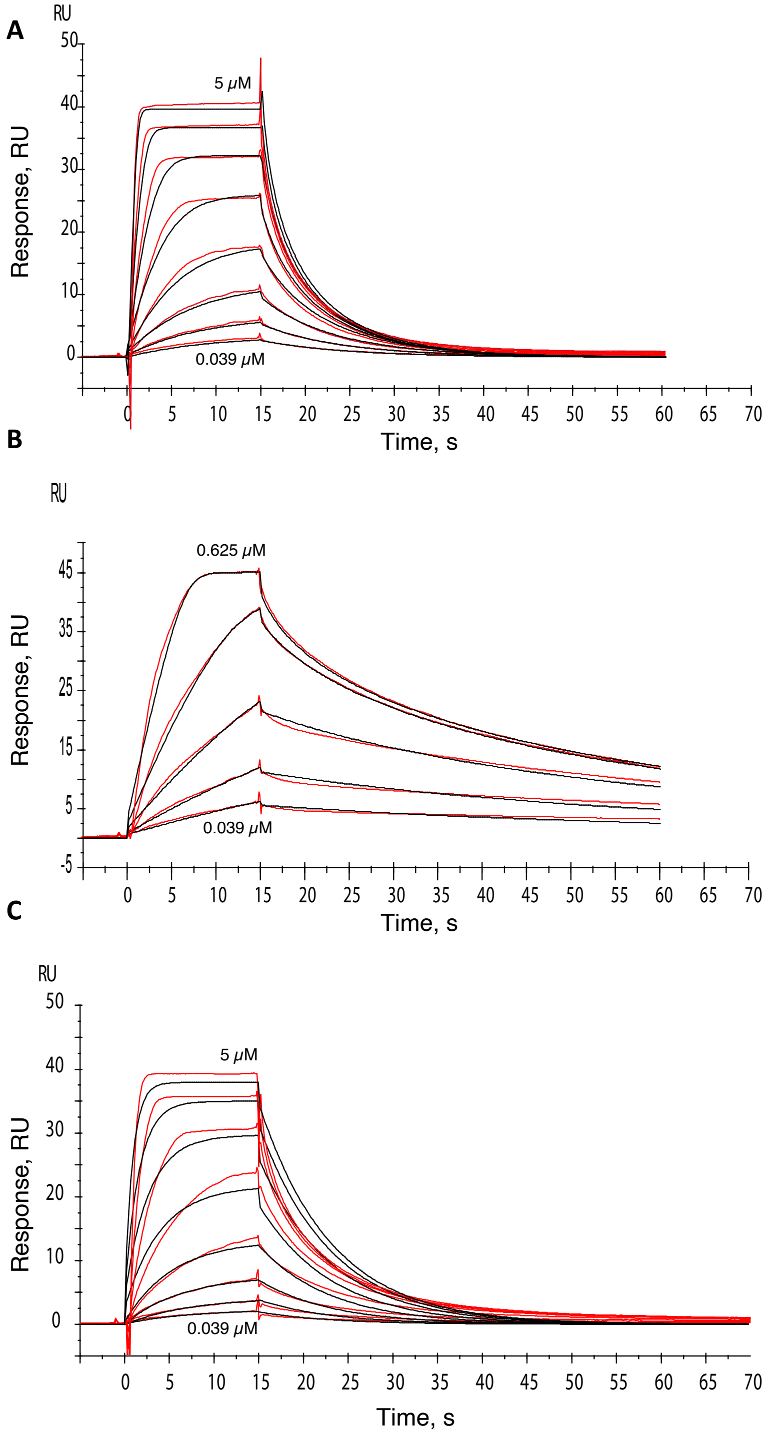

Figure S5. Typical SPR traces for **A)** CypA/**1**, **B)** CypB/**1** and **C)** CypD/**1**.

Calcium Retention Capacity Assay

These experiments were subcontracted to Eurofins. The compounds were prepared as a 10 mM stock solution in 100% DMSO. Subsequent dilutions were made in 100% DMSO and then in assay buffer to 10-fold the final concentration.

HepG2 cells were permeabilized with 100 μM digitonin for 10 min in ice cold buffer containing 1mM EGTA. Following two wash steps to remove the digitonin, the cells were plated into 96 well black and clear plates at 10^6 cell per well in a 180 μL assay buffer containing 0.5 μM Calcium Green 5N. Compounds dilution were prepared in DMSO to 1000 fold the final concentration, diluted 1:100 in assay buffer and added to the assay as 20 μL per well. The assay buffer contained 5 mM glutamate and 2.5 mM malate. The cell plate was immediately run on the FLIPR Tetra^{tm} which added 5 μL of 200 μM (5 μM) calcium chloride every 5 minutes whilst reading the plate every 3 seconds. The area under the curve (AUC) of the raw trace was calculated at each concentration. AUC values were plotted against concentration and EC50 values calculated. Experiments were repeated three times and the EC50 value was determined from a curve fitted to the averaged replicates.

In Vitro Hepatic Steatosis Assay

These experiments were carried out using Cytochroma's proprietary induced pluripotent stem cell (iPSC)-derived hepatocytes which demonstrate genotypic and phenotypic signatures consistent with a metabolic associated steatohepatitis (MASH) phenotype when loaded with free fatty acids.

Hepatocytes were differentiated from IPSCs over 18 days and treated with fatty acid-supplemented medium over 48 hours to induce the MASH phenotype. The MASH induction medium was then replaced with medium supplemented with compounds. The compounds, which had been provided as 10 mM stock solution in DMSO, were serially diluted in 100% DMSO to achieve 1000X the required final concentration, then further diluted 1 in 1000 in culture medium for hepatocyte treatment. After 48 hours treatment, the hepatocytes were fixed in 4% paraformaldehyde and stained for identification of the cell nucleus (DRAQ7, Thermofisher scientific), and neutral cellular lipid (BioTracker 488 Green Lipid Droplet Dye, Merck). Cells were imaged with a 10X objective using the CellInsight CX7 LZR Pro HCS Platform, and Software. Thermo Scientific HCS Studio 5.0 image analysis procedures were applied to identify, measure and quantify nuclei and lipid droplets within the cells. Data were analysed using GraphPad Prism 10.4.1 to produce a concentration response curve (reporting EC_{50} with 95% confidence interval) and column charts (reporting p values following 1-way ANOVA with Dunnett post-hoc).

DMPK Tox experiments

These experiments were subcontracted to Eurofins.

Aqueous solubility

Kinetic solubility measurements were carried out using a shake-flask technique. Compounds were incubated for 24 hours at room temperature. Aqueous solubility (μM) was determined by comparing the peak area of the principal peak in a calibration standard (200 μM) containing organic solvent (methanol/water, 60/40, v/v) with the peak area of the corresponding peak in a buffer sample. In addition, chromatographic purity (%) was defined as the peak area of the principal peak relative to the total integrated peak area in the HPLC chromatogram of the calibration standard. A chromatogram of the calibration standard of each test compound, along with a UV/VIS spectrum with labeled absorbance maxima, was generated.

Partition Coefficient

These were measured using a shake-flask technique. Compounds were incubated for 60 min at room temperature. The total amount of compound was determined as the peak area of the principal peak in a calibration standard (100 μM) containing organic solvent (methanol/water, 60/40, v/v). The amount of compound in buffer was determined as the combined, volume corrected, and weighted areas of the corresponding peaks in the aqueous phases of three organic-aqueous samples of different composition. An automated weighting system was used to ensure the preferred use of raw data from those samples with well quantifiable peak signals. The amount of compound in organic was calculated by subtraction. Subsequently, Log D was calculated as the Log_{10} of the amount of compound in the organic phase divided by the amount of compound in the aqueous phase.

Protein Binding

The peak areas of the test compound in the buffer and test samples were used to calculate percent binding and recovery.

Permeability

The apparent permeability coefficient (Papp) of the test compound was calculated as follows:

$$P_{app}\left(\frac{cm}{s}\right) = \frac{V_R \times C_{R,end}}{\Delta t} \times \frac{1}{A \times \left(C_{D,mid} - C_{R,mid}\right)}$$

where VR is the volume of the receiver chamber. $C_{R,end}$ is the concentration of the test compound in the receiver chamber at the end time point, Δt is the incubation time and A is the surface area of the cell monolayer. $C_{D,mid}$ is the calculated mid-point concentration of the test

compound in the donor side, which is the mean value of the donor concentration at time 0 minute and the donor concentration at the end time point. $C_{R,mid}$ is the mid-point concentration of the test compound in the receiver side, which is one half of the receiver concentration at the end time point. Concentrations of the test compound were expressed as peak areas of the test compound.

Cytochrome P450 Inhibition (HPLC-UV/VIS and HPLC-MS/MS detection)

Peak areas corresponding to the metabolite of each substrate were recorded. The percent of control activity was then calculated by comparing the peak area obtained in the presence of the test compound to that obtained in the absence of the test compound. Subsequently, the percent inhibition was calculated by subtracting the percent control activity from 100 for each compound. IC_{50} values (concentration causing a half-maximal inhibition of control values) were determined by non-linear regression analysis of the concentration-response curve using Hill equation curve fitting.

Intrinsic Clearance (microsomes, S9, cryopreserved hepatocytes)

Metabolic stability, expressed as percent of the parent compound remaining, was calculated by comparing the peak area of the compound at the time point relative to that at time-0. The half-life ($T_{1/2}$) was estimated from the slope of the initial linear range of the logarithmic curve of compound remaining (%) vs. time, assuming the first-order kinetics. The apparent intrinsic clearance (CL_{int}, in µL/min/pmol, µL/min/mg or µL/min/Mcell) was calculated according to the following formula:

$$CL_{int} = \frac{0.693}{T_{1/2} * (mg\ protein/\mu L\ or\ million\ cells/\mu L)}$$

Bacterial Cytotoxicity

Bacterial cytotoxicity tests were carried out using Reverted Salmonella typhimurium strains (TA98, TA100, TA1535, TA1537) that were incubated 96 hours at 37 Celsius. The results for cytotoxicity are expressed as percent of control growth (OD_{650}). Compounds with growth of less than 60 % of control are flagged and considered cytotoxic. None of the compounds tested showed evidence of cytotoxicity.

Ames Tests

Compounds were incubated for 96 hours at 37°C at 4 different concentrations (5 mM, 10 mM, 50 mM, 100 mM) using *Salmonella typhimurium* strains (TA98, TA100, TA1535, TA1537) in the absence and presence of rat liver S9 microsomes. Wells that displayed bacteria growth due

to the reversion of the histidine mutation (as judged by the ratio of OD_{430}/OD_{570} being greater than 1.0) are counted and recorded as positive counts. The significance of the positive counts between the treatment (in the presence of test compound) and the control (in the absence of test compound) are calculated using the one-tailed Fisher's exact test. Three significance levels are reported as follows:

Weak positive, if $0.01 \leq p < 0.05$, denoted as "+"
Strong positive, if $0.001 \leq p < 0.01$, denoted as "++"
Very strong positive, if $p < 0.001$, denoted as "+++"

Control experiments were carried out with 2-aminoanthracene (10 mM), 9-aminoacridine (10 mM), quercetin (30 mM) and streptozocitin (2.5 mM). None of the tested cyclophilin inhibitors showed significant differences from background rate.
